# Fluorinated tetrapodal anion transporters

**DOI:** 10.1016/j.isci.2023.105988

**Published:** 2023-01-23

**Authors:** Alexander M. Gilchrist, Xin Wu, Bryson A. Hawkins, David E. Hibbs, Philip A. Gale

**Affiliations:** 1 School of Chemistry, The University of Sydney, Sydney, NSW 2006, Australia; 2 School of Pharmacy, The University of Sydney, Sydney, NSW 2006, Australia; 3 The University of Sydney, The University of Sydney Nano Institute (Sydney Nano), Sydney, NSW 2006, Australia

**Keywords:** Chemistry, Organic chemistry, Organic synthesis

## Abstract

Synthetic anion transporters show potential in treating life-threatening diseases like cystic fibrosis and cancer. However, with increasingly complex transporter architectures designed to control anion binding and transport, it is important to consider solubility and deliverability during transporter design. The fluorination of synthetic anion transporters has been shown to tune the transporter lipophilicity, transport rates, and binding strength. In this work, we expand on our previously reported tetrapodal (thio)urea transporters with a series of fluorinated tetrapodal anion transporters. The effects of fluorination on tuning the lipophilicity, solubility, deliverability, and anion transport selectivity of the tetrapodal scaffold were investigated using anion-binding and transport assays. The primary mode of anion transport was H^+^/X^−^ cotransport, with the most fluorinated tetrathiourea (**8**) displaying the highest transport activity in the 8-hydroxypyrene-1,3,6-trisulfonic acid (HPTS) assay. Intriguingly, inversion of the transmembrane Cl^−^ vs NO_3_^−^ transport selectivity compared with previously reported tripodal (thio)urea transporters was observed under a modified HPTS assay.

## Highlights

• Eight fluorinated tetrapodal anion transporters were synthesized and characterized
• Nonselective H^+^/X^−^ cotransport was the primary mode of anion transport
• The expected anion transport selectivity of Cl^−^ vs NO_3_^−^ was inverted

## Graphical abstract

**Figure:**
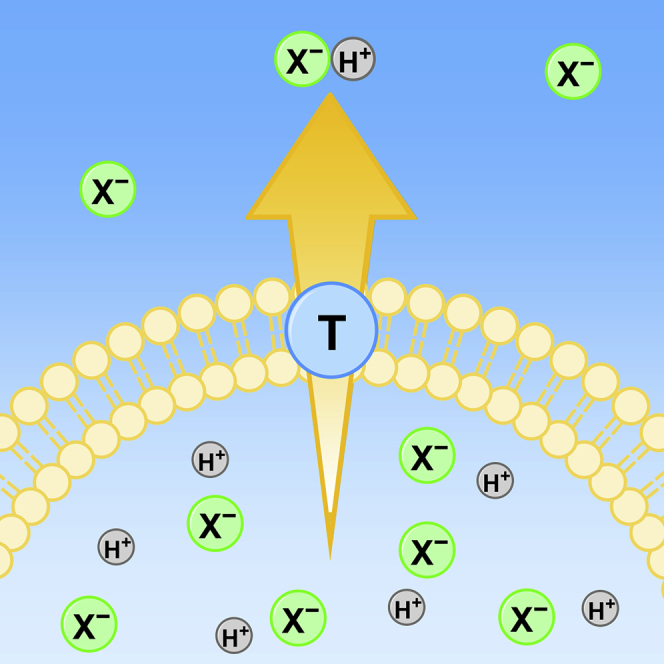

## Introduction

The transport of anionic species across the lipid bilayers of cells, often mediated by membrane-bound proteins or ion channels, is crucial for correct biological function.^1^ The malfunction of ion channels, and the subsequent loss of the anion permeability, can have severe physiological impacts.^2^ The resultant diseases are collectively known as channelopathies. Currently, no cures exist for channelopathies like cystic fibrosis, with only treatments available to improve a patient’s quality of life.^3^ Small-molecule anion transporters have been proposed as a potential therapeutic avenue for such diseases.^4^ By facilitating anion uniport, preferably without proton transport that leads to toxicity, anion transporters are proposed to replace the function of debilitated ion channels.^4^

Researchers have also explored the use of anionophores in anticancer therapies as inspired by the potent anticancer activity of the natural anionophore prodigiosin which functions as an H^+^/Cl^−^ symporter.^5^ Disruption of the pH gradient across plasma or organelle membranes through H^+^/X^−^ symport or OH^−^/X^−^ antiport ultimately triggers programmed cell death via apoptosis and autophagy.^5^ While this approach has potential use in cancer therapies, the challenge of minimizing off-target transport function, such as in anion channel-replacement therapies, remains. Mitochondrial uncoupling and resulting inhibition of the oxidative phosphorylation mechanism involved in adenosine triphosphate (ATP) synthesis within mitochondria is another avenue for anticancer therapies.^6^ Small-molecule transporters have been shown to increase proton permeability via a fatty acid-coupled transport process.^6^ Reduced cancer cell viability was observed as a result of depolarization of the mitochondrial membrane from enhanced proton transport.^6^ As such, anion transporters have a variety of potential biomedical applications, including channelopathy disease treatments and enhancing proton permeability through mitochondrial uncoupling, each of which requires a different anion transport mechanism.^7^

Highly efficient binding and transport of anions across a phospholipid bilayer remains a significant challenge, despite previous work exploring the use of many structurally diverse scaffolds.^5^ One feature which has been investigated is the effect of varying the degree of fluorination of a series of anionophores which was found to fine-tune their lipophilicity and anion-binding affinity. Lipophilicity is an important property when designing anion transporters as a careful balance must be kept for a transporter to partition into the lipid bilayer membrane without precipitating from the aqueous phase. We have previously investigated a series of fluorinated tris-tren tripodal transporters.^6^ It was shown that increasing the degree of fluorination resulted in more lipophilic and stronger hydrogen bond-donating transporters with improved membrane permeability, anion-binding affinity, and transport activity.^6^ In addition, a series of fluorinated bambus[6]uril macrocyclic antiporters were shown to be highly selective for Cl^−^/HCO_3_^−^ and were capable of efficient anion exchange across phospholipid bilayers.^8^ Efficient Cl^−^/HCO_3_^−^ transport has been previously observed in a series of structurally simple fluorinated and non-fluorinated *o*-phenylenediamine-based bis urea transporters.^9^ It was found that increasing the degree of fluorination can effectively enhance transport activity.^9^

**Figure 1:**
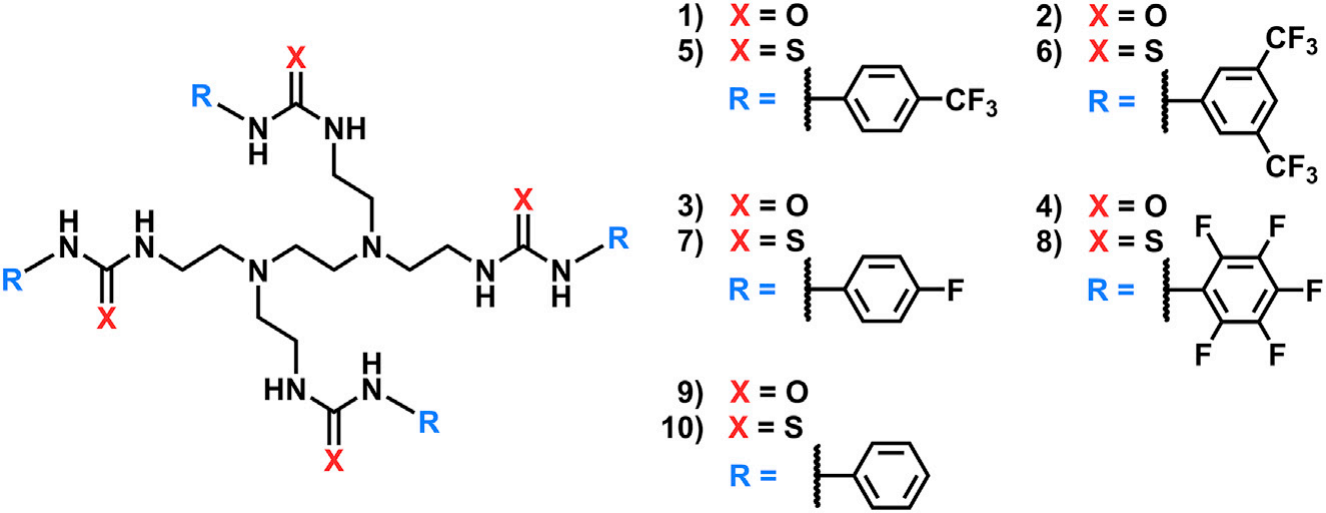
The fluorinated tetraurea (**1**–**4**), tetrathiourea (**5**–**8**) transporters, and non-fluorinated controls **9**–**10**

We have previously developed non-fluorinated tetrapodal anion transporters (Figure 1, compounds **9** and **10**), which were more selective for Cl^−^ uniport against H^+^ transport than the structurally similar tripodal transporters and, therefore, potentially more applicable as a future ion channel disease treatment.^10^^,^^11^^,^^12^ Previously, we have shown that the anionophore-faciliated fatty acid flip-flop pathway is a prominent mechanism of pH gradient dissipation by anion transporters.^7^^,^^10^ It was shown that the tetrapodal anion transporters exhibited Cl^−^ uniport selectivity in the presence of fatty acids due to the enhanced binding site encapsulation offered by the tetrapodal scaffold making fatty acid carboxylate head group binding unfavorable compared with Cl^−^ binding.^10^^,^^13^^,^^14^

To further investigate the effects of both binding site encapsulation and the addition of fluorinated substituents on anion transporters, we report here the design and synthesis of a novel fluorinated tetra(thio)urea transporter series (Figure 1, compounds **1**–**8**). In contradistinction to the analogous tripodal transporters, we show that fluorination of the aryl substituents results, in most cases in decreased Cl^−^ transport activity compared to the non-fluorinated analogues in the Cl^−^/NO_3_^−^ exchange assay, presumably due to poor solubility and low membrane partitioning of fluorinated transporters. The benefit of enhanced lipophilicity via fluorination, however, was observed in an 8-hydroxypyrene-1,3,6-trisulfonic acid (HPTS) assay which is more sensitive and allows lower transporter loadings to be used. HPTS assays performed after sequestering fatty acids from vesicles revealed that the tetrapodal transporters facilitated H^+^/Cl^−^ symport predominantly via a fatty acid-dependent mechanism. We also demonstrate that the tetrapodal scaffold led to inverted NO_3_^−^ vs Cl^−^ selectivity compared with the tripodal compounds.

## Results and discussion

### Synthesis

**Figure 2:**
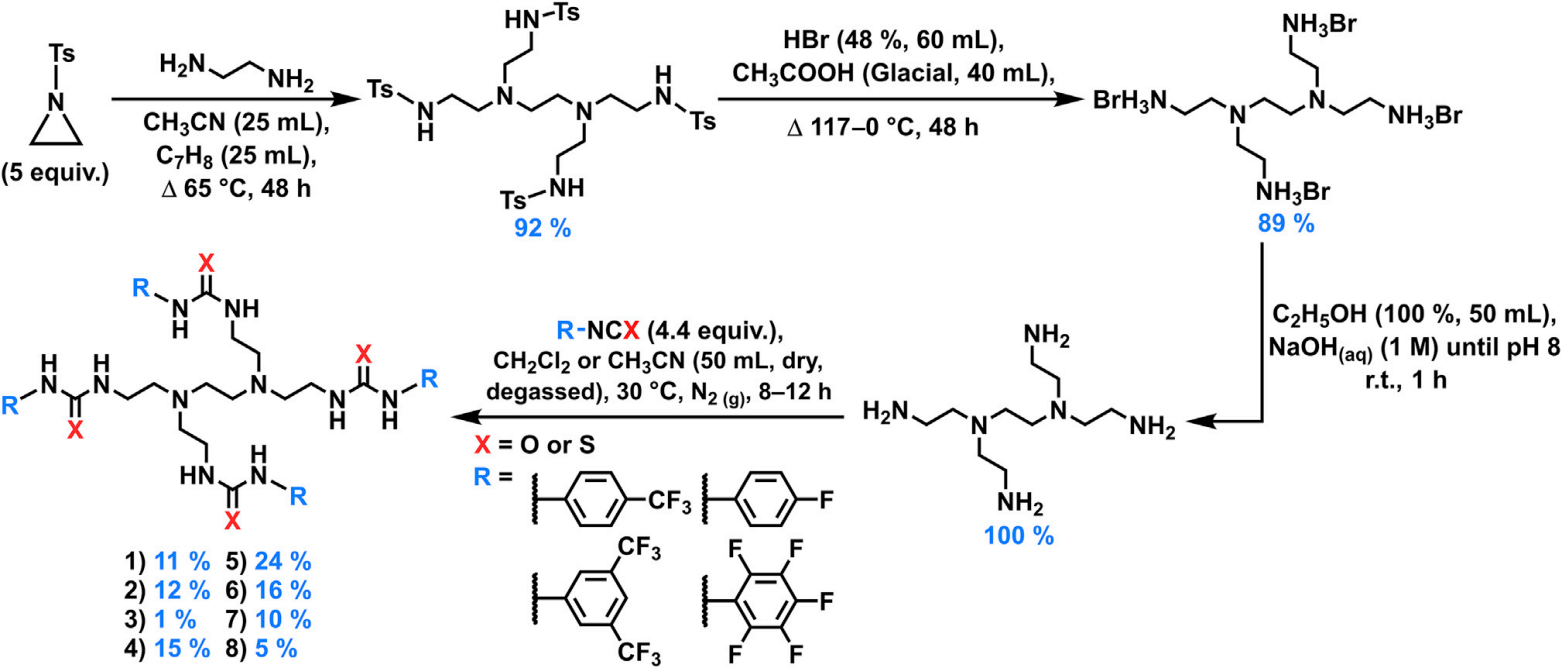
The four-step synthetic scheme of the fluorinated tetrapodal receptors (**1**–**8**), where X is representative of a urea or thiourea moiety and R denotes the fluorinated aryl substituents

A four-step reaction pathway was used to synthesize the fluorinated tetrapodal receptors (Figure 2, compounds **1**–**8**). Firstly, a reaction of *N*-tosylaziridine (5.0 equiv) and ethylenediamine resulted in the synthesis of the previously reported tetrakis tosyl sulfonamide.^15^^,^^16^ The tosyl groups were removed via deprotection to yield the bromine salt.^16^^,^^17^ The free tetrakis amine was afforded through neutralization of the bromide salt, which was further reacted in CH_2_Cl_2_ or CH_3_CN with the appropriate iso(thio)cyanate (4.4 equiv).^10^^,^^18^ The crude products were purified by solvent washes and preparative thin-layer chromatography to afford receptors **1**–**8**. For further synthetic and characterization details, refer to the STAR Methods section, and for all characterization spectra, refer to Figures S1–S24 in the supplemental information.

To further characterize the structures of **1**–**8**, multiple attempts were made to grow single crystals that would diffract well enough to obtain X-ray crystal structures. Multiple crystallization systems were tested, varying by the solvent system, receptor concentration, guest concentration, temperature, evaporation rate, and water diffusion rate. However, due to the high degree of freedom in the scaffold, the crystallization process of these compounds was slow. However, it was important to obtain solid-state structures to provide further evidence of intramolecular bonding and host-guest complexation.

### Crystallography

**Figure 3:**
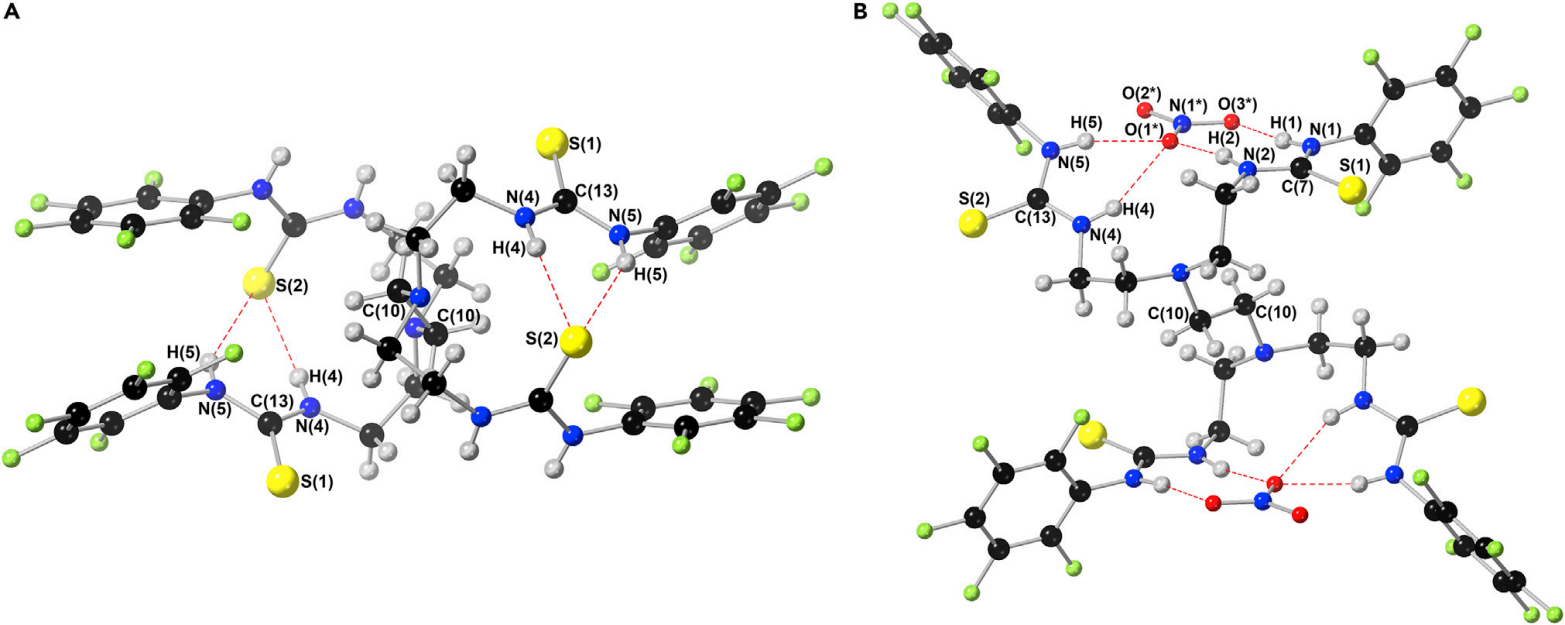
The single crystal X-ray structures of receptor **8** and nitrate complex of **8** (**8**·2NO_3_) (A) The single crystal X-ray structure of receptor **8**. (B) The single crystal X-ray structure of the 1:2 host-guest nitrate complex of **8** (**8**·2NO_3_). Hydrogen, carbon, nitrogen, oxygen, fluorine, and sulfur atoms are depicted as gray, black, blue, red, lime-green, and yellow, respectively. Hydrogen-bonding interactions are shown as broken red bonds; atoms which are referenced in text are labeled, while solvent and TBA countercations have been removed for clarity.

Single-crystal X-ray diffraction was performed on single crystals of the free receptor (**8**) and the host:guest nitrate complex of **8** (**8**·2NO_3_, Figures 3A and 3B). Single crystals of **8** were grown from slow evaporation of a saturated solution of **8** in acetone, while **8**·2NO_3_ was grown in the presence of nitrate (added as the tetrabutylammonium (TBA) salt) through slow diffusion of water into an initial DMSO-*d*_6_/0.5% H_2_O solvent system.

For **8**, the asymmetric unit contains half the full molecule of **8** with C(10), giving localized symmetry, and extending outside of the asymmetric unit. The symmetric unit is tightly closed by 2 intramolecular hydrogen bonds N(4)–H(4) ⋯ S(2), 2.401 Å, 165.03° and N(5)–H(5) ⋯ S(2), 2.720 Å, 148.09°. The remaining crystal system is maintained by 5 intermolecular bonds extending outside the asymmetric unit, denoted in Table 1.

**Table 1:** The hydrogen bonds in the solid-state structure of receptor **8**

D–H	d(D–H)	d(H⋯A)	<DHA	A
N(4)–H(4)	0.880	2.401	165.06	S(2)
N(5)–H(5)	0.880	2.720	148.09	S(2)
N(2)–H(2)	0.880	2.701	151.11	S(1) [x+1, y, z]
N(1)–H(1)	0.880	2.441	157.90	S(1) [x+1, y, z]
N(5)–H(5)	0.880	3.029	128.46	S(2) [−x+1, −y+1, −z+1]
C(9)–H(9A)	0.990	2.634	164.96	F(4) [x, y+1, z]
C(12)–H(12B)	0.990	2.822	148.48	S(1) [−x, −y+1, −z]

D is a hydrogen bond donor, H is a hydrogen atom, d is distance, A is the hydrogen bond acceptor, and <DHA is the angle of bond formation.

For **8·**2NO_3_, the asymmetric unit contains, like **8**, half a full molecule with the central (C(10)) carbon atoms providing localized symmetry. Evidently, from Figures 3A and 3B, the intramolecular bonds maintaining **8** are displaced within **8·**2NO__3__, opening the pentafluoro-aromatic sandwich system, and rotating the sulfur atoms away from the cavity. This allows the nitrate anion to include in the system maintained by 6 hydrogen bonds. Within this structure, O(1^∗^) is tightly held with a trifurcated bonding arrangement created by N(4)–H(4), N(2)–H(2), and N(5)–H(5) (Table 2).

**Table 2:** The hydrogen bonds in the solid-state structure of **8**·2NO_3_

D–H	d(D–H)	d(H⋯A)	<DHA	A
N(4)–H(4)	0.880	2.352	149.02	O(1^∗^)
N(2)–H(2)	0.880	2.013	166.97	O(1^∗^)
N(5)–H(5)	0.880	2.051	162.29	O(1^∗^)
N(5)–H(5)	0.880	2.574	161.74	N(1^∗^)
N(1)–H(1)	0.880	1.990	168.07	O(3^∗^)
C(9′)–H(9′A)	0.990	2.776	151.04	S(2)
C(14′)–H(15′A)	0.990	2.92	167.07	S(2)
C(5′)–H(5′B)	0.990	2.465	137.89	O(2^∗^) [x−1, y, z]
C(13′)–H(13′A)	0.990	2.576	155.80	O(3^∗^) [x−1, y, z]
C(13′)–H(13′B)	0.990	2.5613	135.59	F(1) [−x+1, −y+1, −z+2]
C(2′)–H(2′A)	0.990	2.966	133.57	S(1) [x−1, y−1, z]
C(11)–H(11C)	0.990	2.600	148.00	F(5) [−x+1, −y+1, −z+1]
C(9′)–H(9′B)	0.990	3.004	166.84	S(2) [−x, −y, −z+1]

D is a hydrogen bond donor, H is a hydrogen atom, d is distance, A is the hydrogen bond acceptor, and <DHA is the angle of bond formation.

In addition, N(5)–H(5) of **8·**2NO_3_ also provides two further hydrogen bonds with N(1^∗^) and O(2^∗^). The two NO_3_^−^ anions are held in place by a hydrogen bond between N(1)–H(1) (Table 2). These bonds are of a classical nature and provide a more tightly bound system in this configuration compared to the intermolecular bonding of **8**, allowing for anion binding and transport. All these bonds can be found in Table 2. In **8·**2NO_3_, there are also two weak intermolecular hydrogen bonds between the C(9′)–H(9′A) and C(14′)–H(14′A) atoms of a neighboring molecule, which was removed for clarity, to S(2) to maintain the open cage. Like **8**, there are also six further hydrogen bonds extending outside the asymmetric unit, which can be found in Table 2. Further crystallographic information can be found in Table S2.

### Anion-binding studies

The ability of the receptors to bind to chloride (Cl^−^), bicarbonate (HCO_3_^−^), nitrate (NO_3_^−^), dihydrogen phosphate (H_2_PO_4_^−^), and pyrophosphate (HP_2_O_7_^3−^) anions was investigated using proton-NMR titrations. Using a host solution of a constant concentration (1 mM) in DMSO-*d*_6_/0.5% H_2_O, the respective anions, as the TBA or tetraethylammonium (TEA, for HCO_3_^−^) salts, were incrementally titrated into the host receptor solution. The chemical shifts (ppm) of the proton peaks involved in binding were recorded from stacked spectra. Anion binding constants (*K*_a_) of the host:guest complexes that formed were derived using the BindFit web applet, and the experimental, fitted data and residual error were plotted (Table 3, Figures S69–S108).^19^^,^^20^^,^^21^

**Table 3:** The Host:Guest binding constants of the fluorinated tetrapodal anion transporters (1 mM)

Receptor	Host:Guest Binding Constants (*K*_a_, M^−1^)	
	Cl^−^	NO_3_^−^
1	*K*_11_: 371	No Binding
	*K*_12_: 33.4	
2	*K*_11_: 271	No Binding
	*K*_12_: 3.84	
3	*K*_11_: 507	*K*_a_: 1.33
	*K*_12_: 44.5	
4	*K*_11_: 135	*K*_a_: 2.40
	*K*_12_: 14.1	
5	*K*_11_: 352	*K*_a_: 1.51
	*K*_12_: 1.96	
6	*K*_11_: 170	*K*_a_: 1.64
	*K*_12_: 4.18	
7	*K*_11_: 160	No Binding
	*K*_12_: 13.4	
8	*K*_11_: 155	No Binding
	*K*_12_: 9.20	

All binding studies were performed in DMSO-*d*_6_/0.5% H_2_O. All reported fitting error is under 5.4 %.

The competitive nature of the DMSO-*d*_6_/0.5% H_2_O solvent system, and the host-cavity complementarity toward the complex geometry of the NO_3_^−^ ion, resulted in low but observable NO_3_^−^ binding affinities for **3**–**6**, which fit a 1:1 (host:guest) binding model (Figures S81, S86, S90, and S97). However, comparing the crystallographic studies of **8·**2NO_3_ in the same solvent system also containing an excess of NO_3_^−^ ions shows that while binding data could only be fit accurately to a 1:1 binding model, it is possible for NO_3_^−^ to occupy two separate binding sites (Figure 3B). The urea (**4**) and thiourea (**6**) with high levels of fluorination had the highest binding affinities, followed by **3** and **5**, while no binding was observed for receptors **1**, **2**, **7**, and **8** (Figures S71, S76, S102, and S106). No binding constant could be obtained for the HP_2_O_7_^3−^ due to receptor deprotonation or complex binding during the experiments. Proton-NMR titrations studying H_2_PO_4_^−^ and HCO_3_^−^ binding were also attempted. However, the receptors likely complexed with HPO_4_^2−^ and CO_3_^2−^ anions as indicated by the magnitude of chemical shift changes being greater than those observed for binding of two Cl^−^ anions, and therefore binding constants were not quantified (Figures S70, S72, S75, S77, S80, S82, S85, S87, S90, S92, S95, S98, S100, S101, S105, and S107).^22^ Most of the receptors were seen to either immediately deprotonate or exhibit complex binding upon the addition of the guest on the NMR timescale.

The data obtained from the Cl^−^-binding studies of each receptor was fit to a 1:2 host:guest binding model to account for the binding of a second chloride anion with all receptors displaying a Fcov_fit_ value > 1 (Table S1). The urea receptors (**1**–**3**) displayed stronger binding affinities to Cl^−^ than the thiourea receptors (**5**–**7**), with the only exception being the perfluorinated receptors **4** and **8** (Figures S69, S74, S79, S84, S89, S94, S99, and S104). The overall weaker Cl^−^ binding of the pentafluorinated receptors (**4** and **8**) is due to steric hindrance imposed by the ortho-fluorine substituents. The Cl^−^ binding constants of thiourea receptors **6**, **7**, and **8** were weaker than those of the unsubstituted controls **9** and **10** (*K*_11_ = 447, *K*_12_ = 28 M^−1^ and *K*_11_ = 458, *K*_12_ = 30 M^−1^, respectively, Table 3).^10^ This reversal in expected binding affinities, where the thiourea receptors produce binding constants lower than the analogous urea receptors despite being more acidic, is also observed in previously reported tetrapodal and tripodal scaffolds.^10^^,^^11^ Furthermore, the rearrangement of thiourea moieties from the lowest energy state with the N–H protons pointing away from each other, *trans*-*cis*, to the ideal binding confirmation where both N–H protons point in the same direction, *trans*-*trans*, incurs an energetic penalty.^11^^,^^23^ The reversal in (thio)urea binding affinity also agrees with the previously reported fluorinated tris-tren tripodal anion transporters.^11^ Like the fluorinated tetrapods, the fluorinated tripodal thioureas exhibited lower Cl^−^ binding constants than both the fluorinated ureas and non-fluorinated (thio)ureas.^11^

### Transport studies

#### The chloride/nitrate exchange assay

The ability of receptors **1**–**8** to facilitate the transmembrane exchange of Cl^−^ and NO_3_^−^ anions was investigated using the chloride/nitrate exchange assay. Large unilamellar vesicles (200 nm) of 1-palmitoyl-2-oleoyl-*sn*-glycero-3-phosphocholine (POPC) were prepared following the procedure previously reported.^24^ Using a sodium phosphate salt buffer (5 mM), both the internal and external solutions of the vesicles, NaCl (489 mM) and NaNO_3_ (489 mM), respectively, were buffered to pH 7.2.^24^ A chloride ion-sensitive electrode (ISE) was used to monitor the transporter-facilitated Cl^−^ efflux when added to a system containing the synthetic vesicles, and initial rate constants (*k*_ini_) were calculated (Table 4).

**Table 4:** The Initial Rate Constants (*k*_ini_) From the Chloride/Nitrate Exchange Assay and the Calculated LogP Values of Transporters **1**–**10** (1 mol % with respect to the lipid concentration)

Transporter (1 mol %)	Initial Rate Constants *k*_ini_ (s^−1^)	LogP^a^
1	0.068	5.3
2	b	6.4
3	0.018	3.2
4	b	4.8
5	b	6.0
6	b	7.0
7	0.049	4.5
8	0.036	6.6
9	0.020^c^	3.1^a^^,^^c^
10	0.074^c^	4.1^a^^,^^c^

a Calculated using the online LogP prediction software ALOGPS2.1 by VCCLAB.^25^^,^^26^

b Initial rate constants could not be derived due to solubility issues eliciting less than 10 % chloride efflux.

c Initial rate constants and calculated LogP values, which have been previously reported.^10^

The low solubility of the transporters **1**–**8** prevented an accurate EC_50_ value (the concentration of each transporter required to facilitate half of the maximum recorded Cl^−^ efflux at *t* = 270 s) from being obtained. Therefore, the initial transport rates (*k*_ini_) of **1**–**8** at 1 mol % (with respect to lipid concentration) were calculated using Equations 7 and 8. Any attempt to increase the loading of **1**–**8** at 10 mol % resulted in precipitation and interference with the ISE probe readings. In addition, the low chloride efflux (>10 %) prevented the accurate derivation of *k*_ini_ values for **2** and **4**–**6** (Figures S52 and S54–S56). Among the urea-based transporters, the highest *k*_ini_ rate was observed for *p*-(trifluoromethyl)phenyl-substituted tetraurea (**1**), which displays a ∼3-fold improved activity compared to the unsubstituted (**9**), and the less fluorinated (**3**) analogues. Further increases to the degree of fluorination in the ureas (**2** and **4**) resulted in transporter precipitation and, thus, the attenuated initial rates. Among the thioureas, the transport rates decreased with increased fluorination of the transporters due to transporter solubility issues. The trend observed in the tetrapodal series of transporters contrasted with the tripodal transporters, where increased fluorination was found to benefit transport activity due to enhanced lipophilicity. These results indicate that the fluorinated tetrapodal transporters were generally too insoluble for efficient partitioning into the lipid membranes for the ISE anion exchange assay that requires high transport loadings.

#### The HPTS transport selectivity assay

**Figure 4:**
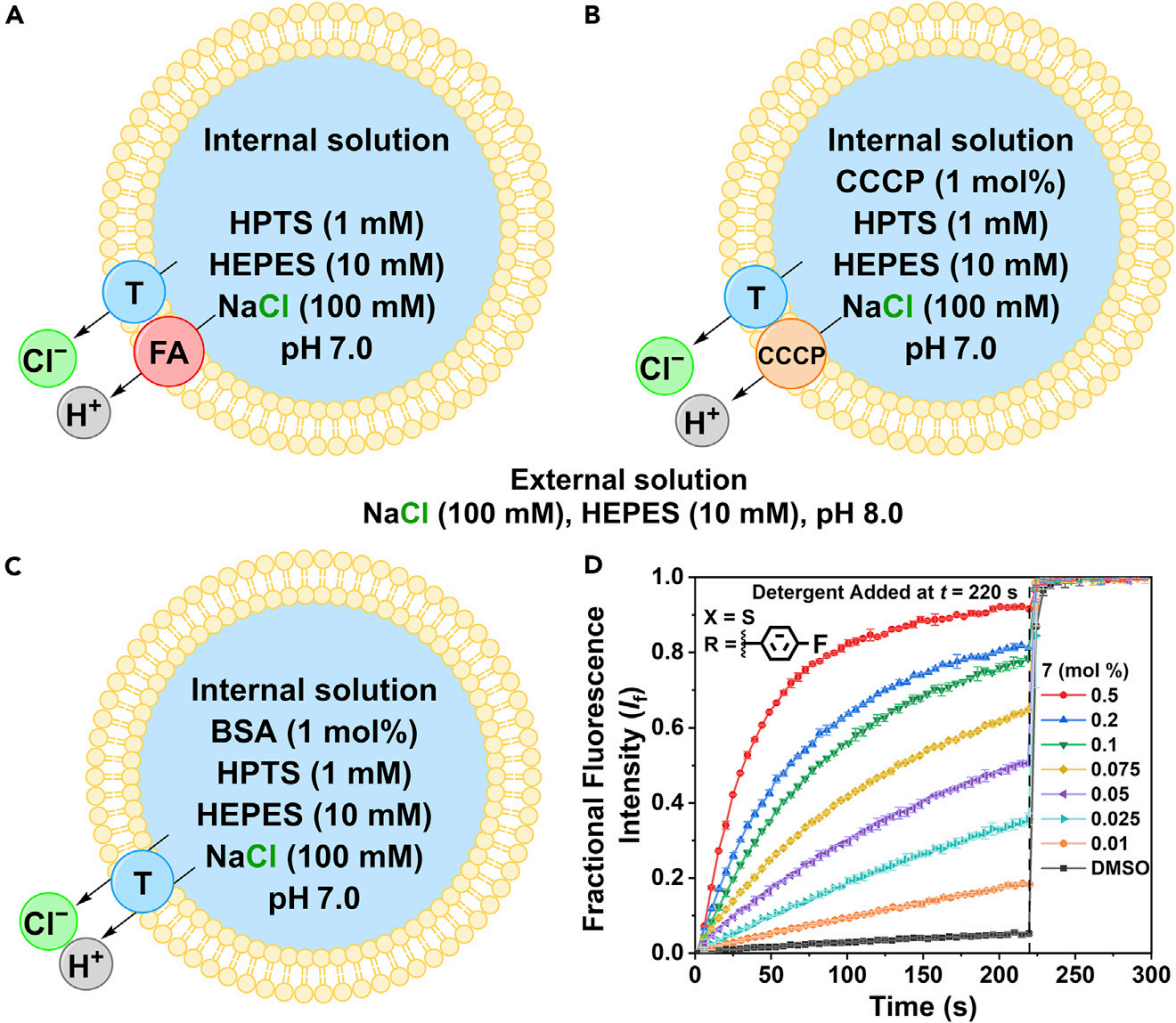
The conditions of the HPTS transport assay and the recorded fluorescent response of **7** (A) Fatty acid-assisted H^+^/Cl^−^ (H^+^, gray and Cl^−^, green) transport was tested using synthetic vesicles (200 nm, yellow) containing membrane-bound fatty acids (FA, red). (B) Transporter (T, blue)-mediated selective transport was tested using vesicles treated with CCCP (1 mol %, orange). (C) Transporter-mediated anion symport was tested using vesicles treated with BSA (1 mol %). (D) The change in fractional fluorescence intensity (*I*_f_) of HPTS when **7** was added to vesicles containing fatty acids at different concentrations, where X and R denote the thiourea and fluorinated aryl substituent, respectively. All BSA, CCCP, and transporter concentrations are shown as mol % values with respect to the lipid concentration, with error bars showing the SD of two repeats.

The HPTS transport selectivity assay was used to assess the ability of transporters **1**–**10** to selectively facilitate Cl^−^ uniport over H^+^ transport and the influence of fatty acids (FA) on H^+^ transport (Figure 4D). Unilamellar vesicles (200 nm) were prepared following a previously reported method*,* containing an internal solution of HPTS (1 mM) and NaCl (100 mM), buffered to pH 7.0 with 4-(2-hydroxyethyl)-1-piperazineethanesulfonic acid (HEPES, 10 mM, Figure 4A).^27^ The vesicles were suspended in an external solution of NaCl (100 mM) and HEPES (10 mM), which was also buffered to pH 7.0. The transporters were added to systems of vesicles at varying concentrations as DMSO solutions so that Hill analysis, using Equation 6, could be performed (Table 5, Figures S33–S42). Using *k*_ini_ constants, transporters **1**–**10** (1 mol %) were also screened for the ability to facilitate selective anion transport by removing the rate-limiting proton transport step and adding the protonophore carbonyl cyanide-*m*-chlorophenylhydrazone (CCCP, 1 mol %, Table 5, Figures 4B and S59–S68). Similarly, the ability of **1**–**10** (1 mol %) to mediate H^+^/Cl^−^ cotransport without the aid of membrane-embedded fatty acids was studied when vesicles underwent pre-treatment with bovine serum albumin (BSA) (1 mol %, Table 5, Figures 4C and S59–S68).^7^^,^^28^

**Table 5:** The EC_50_ (mol %) Values, Hill Coefficients (*n*), and Initial Rate Constants at 1 mol % (*k*_ini_, s^−1^) of both the Fluorinated (**1**–**8**) and Non-Fluorinated (**9** and **10**) Transporters in the HPTS Transport Selectivity Assay

Transporter	EC_50_ (mol %)	Hill Coefficient (*n*)	Initial Rate Constants (*k*_ini_, s^−1^)		
			FA	CCCP	BSA
1	0.1 ± 0.007	1.7	0.005	0.002	0.001
2	0.52 ± 0.04	2.5	0.001	0.001	0.001
3	0.53 ± 0.07	1.0	0.029	0.024	0.024
4	0.25 ± 0.04	1.6	0.014	0.009	0.009
5	0.68 ± 0.16	1.1	0.002	0.005	0.000
6	0.26 ± 0.11	0.5	0.004	0.002	0.001
7	0.056 ± 0.006	1.3	0.030	0.028	0.015
8	0.047 ± 0.007	1.1	0.061	0.042	0.033
9	1.30 ± 0.06	1.5	0.049	0.121	0.013
10	0.18 ± 0.02	1.2	0.015	0.009	0.010

Among the library of aryl (thio)urea-based tetrapodal transporters, only compound **5** and **9** displayed a modestly enhanced transport rate in the presence of CCCP, which indicates selective Cl^−^ uniport over H^+^/Cl^−^ symport. Previously, we have observed a much more pronounced Cl^−^ uniport > H^+^/Cl^−^ symport selectivity for alkyl thiourea-based tetrapodal transporters.^10^ This suggests that alkyl substituents are optimal for Cl^−^ uniport selectivity, likely due to reduced acidity of the (thio)urea motifs which minimize interactions with the carboxylate head group of deprotonated fatty acids. The binding of deprotonated fatty acids and subsequent facilitation of their flip-flop are the dominant mechanism for H^+^ transport by hydrogen bond-based anion transporters, as reported by Wu and Gale^7^ unless the transporters are sufficiently acidic to undergo deprotonation and function as protonophores themselves under the tested pH conditions.^7^

Decreased *k*_ini_ rates calculated for BSA-treated systems compared to the *k*_ini_ rates in untreated vesicles that contain fatty acid impurities show that **1**–**8** primarily facilitate H^+^/Cl^−^ symport aided by the fatty acid flip-flop mechanism. To further analyze the H^+^/Cl^−^ symport mechanism, the transport activities of transporters **1**–**8** were determined by calculating the EC_50_ values under the HPTS assay using untreated vesicles. Compared to the chloride/nitrate exchange assay, compound **8** is the most active transporter. Considering that the HPTS transport assay is more sensitive, only 5 mM of H^+^ and Cl^**−**^ influx is required for complete pH dissipation, compared with the ∼500 mM of Cl^−^/NO_3_^−^ exchange required for 100% transport under the ISE assay. Therefore, the transport activity at lower concentrations under the HPTS assay was not comparable, where the most lipophilic transporter **8** was indeed the best transporter which is consistent with previous findings for the tripodal compounds.^11^

#### Selectivity among anions

**Figure 5:**
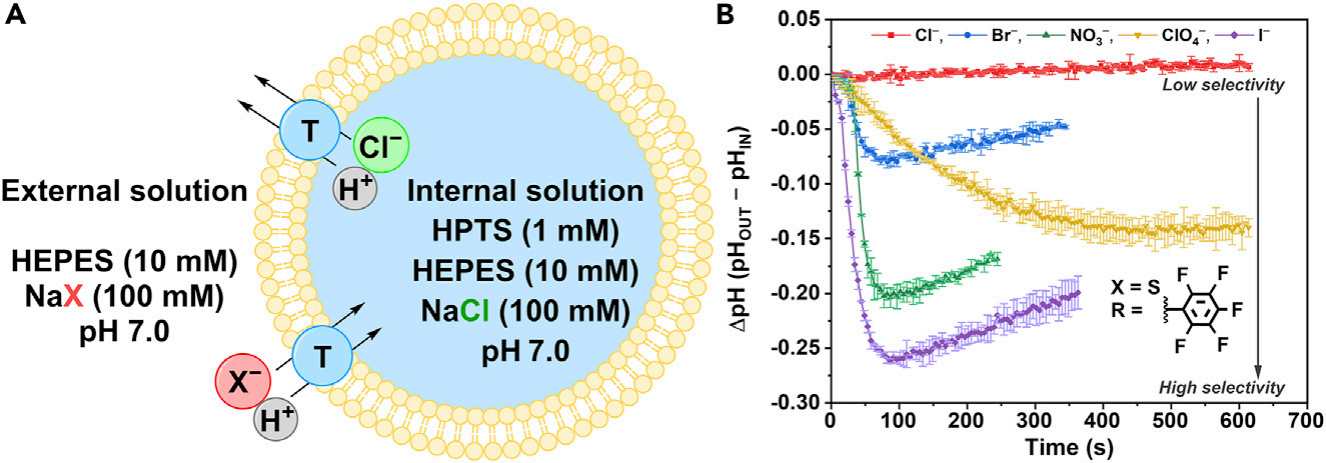
The modified HPTS anion transport selectivity assay (A) The conditions of the modified HPTS anion transport selectivity assay. The synthetic vesicles contain an internal solution of HPTS (1 mM), HEPES (10 mM), and NaCl (100 mM) buffered to pH 7.0. The transporters (T, blue) are added to the vesicles (200 nm, yellow) suspended in a pH 7.0-buffered HEPES (10 mM) and NaX (100 mM, where X = Cl^−^, Br^−^, NO_3_^−^, ClO_4_^−^, and I^−^) external solution. (B) The normalized anion transport selectivity of transporter **8** (0.5 mol %, X and R denote the thiourea and fluorinated aryl substituent, respectively) with error bars showing the SD of two repeats, and a gray arrow indicates the direction of anion selectivity. Transporter concentrations are shown as mol % values with respect to the lipid concentration.

Using a recently reported modified HPTS transport assay, the selectivity of the anion transport facilitated by the transporters was studied (Figures 5A, and S43–S50).^29^ Transporters **1**–**5** and **7** (1 mol %), as well as **6** and **8** (0.5 mol %), were added to systems of various external buffer solutions of Cl^−^, Br^−^, NO_3_^−^, ClO_4_^−^, and I^−^ (100 mM) with suspended vesicles containing an internal solution of HPTS (1 mM), HEPES (10 mM), and NaCl (100 mM) buffered to pH 7.0.

Over the series, none of the transporters displayed Cl^−^ selectivity. Instead, transporters **1**–**5** and **7** displayed I^−^ selectivity followed by ClO_4_^−^ > NO_3_^−^ > Br^−^ > Cl^−^, with a slight perturbation from the Hofmeister series (Figures S43–S47 and S49). Considering the higher hydrophobicity of the ClO_4_^−^, I^−^, and NO_3_^−^ anions, it is expected that the transport of these larger anions is easier to facilitate than the smaller spherical anions like Br^−^ and Cl^−^.^30^ However, it is likely that as a result of the structural flexibility of the tetrapodal scaffold, binding site preorganization is also decreased, which could account for the lower selectivity for the smaller spherical Cl^−^ and Br^−^ anions.^31^

This is in good agreement with the ability of **1**–**8** to bind larger anions with complex geometries during ^1^H-NMR anion-binding studies due to more favorable host-cavity complementarity. This is evidenced by weak but observable NO_3_^−^ binding in the competitive DMSO-*d*_6_/0.5% H_2_O solvent system during binding studies. When taken with the poor chloride transport observed for compounds **1**–**8**, the NO_3_^−^ > Cl^−^ transport selectivity trend suggests that the rate-limiting step is Cl^−^ transport rather than NO_3_^−^ transport. This contrasts with the tripodal transporters, which displayed transport selectivity for Cl^−^ > NO_3_^−^ as they contain fewer degrees of freedom, a smaller binding cavity, and a resultingly higher level of binding-site preorganization optimal for binding the spherical Cl^−^ anion.^11^^,^^29^ Interestingly, for the most acidic transporters of the series, tetrathioureas **6** and **8** were most selective for I^−^, and the Hofmeister selectivity order between the more hydrophobic ClO_4_^−^ anion and NO_3_^−^ was inverted, revealing the I^−^ > NO_3_^−^ > ClO_4_^−^ > Br^−^ >Cl^−^ transport selectivity trend (Figures 5B and S50).

### Conclusions

We have successfully synthesized a series of fluorinated tetrapodal anion transporters and investigated their binding and transport properties. Through binding studies, the transporters were capable of binding chloride anions as a 1:2 host:guest complex as well as anions of more complex geometries. Transport studies showed that **1**–**8** primarily operate via an H^+^/Cl^−^ symport pathway, aided by fatty acid flip-flop. Across the series, increasing fluorination resulted in lower transport activities in assays, like the ISE assays, which require a higher transporter loading. The decrease in transport activities is due to the issue of transporter precipitation showing that enhanced lipophilicity does not benefit transmembrane transport under these conditions. However, in the HPTS transport assay, which requires a lower transporter loading, we can indeed observe the benefit of enhanced lipophilicity from transporter fluorination, which is consistent with our previously reported tripodal compounds.^11^^,^^12^ Therefore, through further investigation of the tetrapodal scaffold by appending fluorinated substituents, we have gained further insight into the effects that receptor fluorination has on anion binding and transport. We show that it is important to consider transporter solubility and deliverability to cell membranes when designing transporters with increasingly complex architectures and enhanced lipophilicity. Interestingly, the larger, more flexible tetrapodal binding sites led to an inverted NO_3_^−^ > Cl^−^ selectivity when compared with the Cl^−^ > NO_3_^−^ transport selectivity reported for the analogous tripodal transporters.^11^^,^^12^ The inversion in NO_3_^−^ vs Cl^−^ transport suggests that transmembrane anion selectivity is tuneable according to the size and flexibility of the binding sites.

### Limitations of the study

The work presented here shows the ability of the fluorinated tetrapodal anion transporters to perform H^+^/Cl^−^ cotransport across lipid bilayers compared to the ability of the non-fluorinated precursor to undergo anion-selective transport. However, the insolubility of these compounds prevented further mechanistic transport studies from being carried out, and further scaffold optimization must be carried out to increase solubility.

## STAR★Methods

### Key resources table

**Table:** 

REAGENT or RESOURCE	SOURCE	IDENTIFIER
**Chemicals, peptides, and recombinant proteins**		

Acetone	Merck – Sigma-Aldrich	CAS №: 67-64-1
Acetonitrile	GENERAL REAGENT	CAS №: 75-05-8
BSA	Merck – Sigma-Aldrich	CAS №: 9048-46-8
Carbonyl cyanide-*m*-chlorophenylhydrazone	Alfa Aesar	CAS №: 555-60-2
Chloroform	Thermo Fisher Scientific	CAS №: 67-66-3
Dichloromethane	GENERAL REAGENT	CAS №: 75-09-2
Diethyl ether	Merck – Sigma-Aldrich	CAS №: 60-29-7
Dimethyl sulfoxide	Merck – Sigma-Aldrich	CAS №: 67-68-5
Dimethyl sulfoxide-*d*_6_	Cambridge Isotopes Ltd. (CIL) via Novachem	CAS №: 2206-27-1
Ethanol (100%)	Merck – Sigma-Aldrich	CAS №: 64-17-5
Ethylenediamine	Merck – Sigma-Aldrich	CAS №: 107-15-3
Glacial acetic acid	Thermo Fisher Scientific	CAS №: 64-19-7
Hexafluorobenzene	Merck – Sigma-Aldrich	CAS №: 392-56-3
Hydrobromic acid (48 %)	Merck – Sigma-Aldrich	CAS №: 10035-10-6
Methanol	Merck – Sigma-Aldrich	CAS №: 67-56-1
Monensin sodium salt	Cayman Chemical	CAS №: 22373-78-0
n-Hexane	Merck – Sigma-Aldrich	CAS №: 110-54-3
n-Pentane	Merck – Sigma-Aldrich	CAS №: 109-66-0
*N*-tosylaziridine	Combi-Blocks	CAS №: 3634-89-7
Paratone®-N (Parabar 10312)	Hampton Research	HR2-643
Pentafluorophenyl isocyanate	Oakwood Chemical	CAS №: 1591-95-3
Pentafluorophenyl isothiocyanate	Oakwood Chemical	CAS №: 35923-79-6
Potassium chloride	Merck – Sigma-Aldrich	CAS №: 7447-40-7
Potassium gluconate	Thermo Fisher Scientific	CAS №: 299-27-4
Sephadex® G-25 (Medium)	Merck – Sigma-Aldrich	CAS №: 9041-35-4
Sodium bromide	Merck – Sigma-Aldrich	CAS №: 7647-15-6
Sodium chloride	Merck – Sigma-Aldrich	CAS №: 7647-14-5
Sodium phosphate monobasic dihydrate	Merck – Sigma-Aldrich	CAS №: 13472-35-0
Sodium hydroxide	Thermo Fisher Scientific	CAS №: 1310-73-2
Sodium iodide	Merck – Sigma-Aldrich	CAS №: 7681-82-5
Sodium nitrate	Thermo Fisher Scientific	CAS №: 7631-99-4
Sodium perchlorate monohydrate	Merck – Sigma-Aldrich	CAS №: 7791-07-3
Sodium phosphate dibasic	Merck – Sigma-Aldrich	CAS №: 7558-79-4
Tetrabutylammonium chloride	Merck – Sigma-Aldrich	CAS №: 1112-67-0
Tetrabutylammonium nitrate	Acros Organics	CAS №: 1941-27-1
Tetrabutylammonium phosphate monobasic	Merck – Sigma-Aldrich	CAS №: 5574-97-0
Tetrabutylammonium pyrophosphate	Merck – Sigma-Aldrich	CAS №: 76947-02-9
Tetraethylammonium bicarbonate	Merck – Sigma-Aldrich	CAS №: 17351-61-0
Toluene	GENERAL REAGENT	CAS №: 108-88-3
Triton™ X-100	Merck – Sigma-Aldrich	CAS №: 9036-19-5
Valinomycin (Potassium ionophore I)	Merck – Sigma-Aldrich	CAS №: 2001-95-8
1-palmitoyl-2-oleoyl-*sn*-glycero-3-phosphocholine (POPC)	Avanti Polar Lipids	CAS №: 26853-31-6
3,5-Bis(trifluoromethyl)phenyl isocyanate	Oakwood Chemical	CAS №: 16588-74-2
3,5-Bis(trifluoromethyl)phenyl isothiocyanate	Oakwood Chemical	CAS №: 23165-29-9
4-(2-hydroxyethyl)-1-piperazineethanesulfonic acid (HEPES)	Merck – Sigma-Aldrich	CAS №: 7365-45-9
4-(Trifluoromethyl)phenyl isocyanate	Oakwood Chemical	CAS №: 1548-13-6
4-(Trifluoromethyl)phenyl isothiocyanate	Oakwood Chemical	CAS №: 1645-65-4
4-Fluorophenyl isocyanate	Oakwood Chemical	CAS №: 1195-45-5
4-Fluorophenyl isothiocyanate	Oakwood Chemical	CAS №: 1544-68-9
8-Hydroxypyrene-1,3,6-Trisulfonic Acid (HPTS)	Merck – Sigma-Aldrich	CAS №: 6358-69-6
1,1′,1″,1‴-((ethane-1,2-diylbis(azanetriyl))tetrakis(ethane-2,1-diyl))tetrakis(3-(4-(trifluoromethyl)phenyl)urea) (**1**)	This Manuscript	
1,1′,1″,1‴-((ethane-1,2-diylbis(azanetriyl))tetrakis(ethane-2,1-diyl))tetrakis(3-(3,5-bis(trifluoromethyl)phenyl)urea) (**2**)	This Manuscript	
1,1′,1″,1‴-((ethane-1,2-diylbis(azanetriyl))tetrakis(ethane-2,1-diyl))tetrakis(3-(4-fluorophenyl)urea) (**3**)	This Manuscript	
1,1′,1″,1‴-((ethane-1,2-diylbis(azanetriyl))tetrakis(ethane-2,1-diyl))tetrakis(3-(perfluorophenyl)urea) (**4**)	This Manuscript	
1,1′,1″,1‴-((ethane-1,2-diylbis(azanetriyl))tetrakis(ethane-2,1-diyl))tetrakis(3-(4-(trifluoromethyl)phenyl)thiourea) (**5**)	This Manuscript	
1,1′,1″,1‴-((ethane-1,2-diylbis(azanetriyl))tetrakis(ethane-2,1-diyl))tetrakis(3-(3,5-bis(trifluoromethyl)phenyl)thiourea) (**6**)	This Manuscript	
1,1′,1″,1‴-((ethane-1,2-diylbis(azanetriyl))tetrakis(ethane-2,1-diyl))tetrakis(3-(4-fluorophenyl)thiourea) (**7**)	This Manuscript	
1,1′,1″,1‴-((ethane-1,2-diylbis(azanetriyl))tetrakis(ethane-2,1-diyl))tetrakis(3-(perfluorophenyl)thiourea) (**8**)	This Manuscript	
1,1′,1″,1‴-((Ethane-1,2-diylbis(azanetriyl))tetrakis(ethane-2,1-diyl))tetrakis(3-phenylourea) (**9**)	School of Chemistry, The University of Sydney	
1,1′,1″,1‴-((Ethane-1,2-diylbis(azanetriyl))tetrakis(ethane-2,1-diyl))-tetrakis(3-phenylthiourea) (**10**)	School of Chemistry, The University of Sydney	
*N,N′,N″,N‴*-((ethane-1,2-diylbis(azanetriyl))tetrakis(ethane-2,1-diyl))tetrakis(4-methylbenzenesulfonamide)	School of Chemistry, The University of Sydney	CAS №: 103635-08-1
*N*^1^,*N*^1^,*N*^2^,*N*^2^-tetrakis(2-(bromo-λ^5^-azaneyl)ethyl)ethane-1,2-diamine	School of Chemistry, The University of Sydney	CAS №: 591732-97-7
*N*^1^,*N*^1^,*N*^2^,*N*^2^-tetrakis(2-aminoethyl)-1,2-ethanediamine	School of Chemistry, The University of Sydney	CAS №: 4097-90-9

**Software and algorithms**		

ChemDraw Professional 21	PerkinElmer	https://perkinelmerinformatics.com/
Bruker TopSpin 4.1.4	Bruker	https://www.bruker.com/
MestReNova	Mestrelab	https://mestrelab.com/
OriginPro 2022 (Academic)	OriginLab®	https://www.originlab.com/
CrysAlis^Pro^	Rigaku Oxford Diffraction	https://www.rigaku.com/
SHELX-T	SHELX Website	https://shelx.uni-goettingen.de/
SHELXL-2015	SHELX Website	https://shelx.uni-goettingen.de/
CrystalMaker® X	CrystalMaker Software Ltd.	http://crystalmaker.com/

### Resource availability

#### Lead contact

Requests for any of the listed chemical reagents and resources should be addressed to the lead contact, Philip A. Gale (philip.gale@uts.edu.au).

#### Materials availability

The syntheses of all reported compounds are described in the synthesis and characterization of compounds. Further information on synthesis and characterization is available in the supplemental information. If available, compounds 1–8 can be requested from the lead contact. All other chemical reagents in the key resources table were either available from the School of Chemistry or the School of Pharmacy at The University of Sydney or bought from commercial suppliers.

### Method details

#### Materials and instrumentation

All chemical syntheses were performed in the School of Chemistry at The University of Sydney under dry N_2 (g)_ at room temperature 25 °C (r.t., 298 K) using anhydrous and degassed solvents unless stated otherwise. The solvents used during the synthesis of compounds **1**–**8** were anhydrous and obtained at The University of Sydney via the Innovative Technology PureSolv7 solvent purification system. The chemicals used in synthesis, characterization, and analytical assays were obtained either internally or commercially from the sources listed in the key resources table. The compounds which required further purification using preparative thin-layer chromatography (Prep-TLC) were performed on Sigma-Aldrich Silica Gel 60 F_245_ (1.0 mm) glass sheets (200 × 200 mm), and all eluent solvent systems are reported as *v/v* ratios as percentages.

Proton-NMR (400 MHz), ^13^C-NMR (126 MHz), and ^19^F-NMR (356 MHz) spectroscopic datasets were collected on either a Bruker Avance AVIII 400 or a Bruker AVIII 500 MHz NMR spectrometer. While ^1^H-NMR (600 MHz) and ^13^C-NMR (151 MHz) data was collected at r.t. on Bruker AVIII 600 MHz NMR spectrometer equipped with a triple nucleus (^1^H, ^13^C, ^15^N) cryogenic probehead. All chemical shifts (*δ*, ppm) are reported relative to the DMSO-*d*_6_^1^H-NMR (2.50 ppm) and ^13^C-NMR (39.7 ppm) solvent peaks, while ^19^F-NMR chemical shifts in DMSO-*d*_6_ are reported relative to the C_6_F_6_ peaks at −164.9 ppm, used as an internal standard. All NMR J-coupling constants (*J*) are reported as Hertz (Hz), with the peak multiplicities reported as singlets (s), doublets (d), triplets (t), quartets (q), doublet of doublets (dd) or multiplets (m).

A Bruker amazon SL mass spectrometer which was equipped with a quadrupole analyzer was used to acquire low-resolution mass spectrometry (LS-MS) data via both positive and negative electrospray ionization (ESI^+^ or ESI^−^, respectively). Using the same technique, a Bruker Solarix 2XR mass spectrometer was used to acquire high-resolution mass spectrometry (HR-MS) data. All relative intensity mass spectrometry data is expressed as mass-to-charge (*m/z*) ratios. Melting points (M.P.) were determined using a Stanford Research System (SRS) OptiMelt Automated Melting Point System and reported as a range in °C.

Anion transport studies were performed using 16:0–18:1 PC (POPC) phospholipids bought from Avanti Polar Lipids. An Accumet chloride ion-sensitive electrode (ISE) was used to monitor fluctuations in Cl^−^ ion concentrations in the Cl^−^/NO_3_^−^ exchange assay (S3.2). All fluorescence-based transport studies were performed with an Agilent Cary Eclipse Fluorescence Spectrophotometer equipped with a temperature controller (maintained at 25°C (298 K)) and a stirrer plate using HPTS, a pH sensitive ratiometric fluorescent probe.

#### Synthesis and characterization of compounds

##### Synthesis of *N,N′,N″,N*‴-((ethane-1,2-diylbis(azanetriyl))tetrakis(ethane-2,1-diyl))tetrakis(4-methylbenzenesulfonamide) (tetrakistosyl sulfonamide)

Using a previously reported method, *N*-tosylaziridine (7.0 g, 5 equiv) was allowed to stir and dissolve in C_7_H_8_ (25 mL, dry) with cooling before the dropwise addition of a solution of CH_3_CN (25 mL, dry) and ethylenediamine (0.4223 mL, 1 equiv) over 0.5 h. The reaction mixture was allowed to stir for 3 h at r.t. before being heated to reflux at 65°C for 48 h. After 48 h, the reaction was allowed to cool, resulting in a white precipitate which was collected via vacuum filtration, washed with CH_3_CN (3 × 5 mL), and dried in *vacuo* resulting in a yield of 5.53 g (92%), which was carried through to the next step.^10^^,^^15^^,^^16^

##### Synthesis of N1,N1,N2,N2-tetrakis(2-(bromo-λ 5-azaneyl)ethyl)ethane-1,2-diamine (tetrakis amine HBr salt)

Using a previously developed synthetic method, the tetrakistosyl sulfonamide (4.0 g) was added to a solution of CH_3_COOH (glacial, 40 mL) and HBr (48%, 60 mL).^10^^,^^16^^,^^17^ The mixture was heated to reflux at 117°C for 48 h and turned from orange to California brick red. After 48 h, the mixture was cooled to r.t. before further cooling in an ice bath. An off-white precipitate formed which was collected via vacuum filtration washed with CH_3_OH (3 × 10 mL) and dried in *vacuo* resulting in the desired bromine salt of the tetrakis amine with a yield of 0.27 g (89%), which was carried through to the next step.

##### Synthesis of N1,N1,N2,N2-tetrakis(2-aminoethyl)-1,2-ethanediamine (tetrakis amine)

Following a previously reported method, the tetrakis amine bromine salt (1.0 g) was suspended in C_2_H_5_OH (100%, 50 mL), and aliquots of NaOH (1 M) was added with stirring until pH 8.^10^^,^^16^^,^^18^ The solvent was evaporated under reduced pressure resulting in a mixture of the desired tetrakis amine (green oil) and sodium bromide salt (white solid). The mixture was further dried in *vacuo* and carried through to the next step without characterization due to instability.

##### Synthesis of 1,1′,1″,1‴-((ethane-1,2-diylbis(azanetriyl))tetrakis(ethane-2,1-diyl))tetrakis(3-(4-(trifluoromethyl)phenyl)urea) (1)

The tetrakis amine (0.2540 g) was added to a solution of CH_3_CN (dry, degassed, 50 mL) under N_2_ (g) and allowed to stir at r.t. for 0.5 h to ensure a homogeneous mixture. The mixture was heated to 30°C (303.15 K), and the 4-(trifluoromethyl)phenyl isocyanate (0.794 mL, 4.4 equiv) was added directly to the reaction via syringe, where a precipitate was observed to form immediately. The reaction was allowed to stir for another 8 h before the solid was filtered and underwent a series of washes with cold CH_3_CN (3 × 10 mL), deionized H_2_O (3 x 10 mL), CH_2_Cl_2_:CH_3_OH (98:2 *v/v*, 1 × 10 mL), and finally CHCl_3_:(CH_3_)_2_CO (70:30 *v/v*, 1 × 10 mL). The off-white solid was collected and dried *in vacuo* with a final yield of 0.1175 g (11%).

^**1**^**H-NMR** (600 MHz, DMSO-*d*_6_): *δ* 9.10 (s, 4H), 7.57 (d, *J* = 8.54 Hz, 8H), 7.50 (d, *J* = 8.56 Hz, 8H), 6.34 (s, 4H), 3.19 (q, *J* = 6.47, 6.28, 6.28 Hz, 8H), 2.63 (s, 4H), 2.61–2.55 (m, 8H). ^**13**^**C-NMR** (151 MHz, DMSO-*d*_6_): *δ* 154.9, 144.3, 125.9, 129.6–120.1 (m), 120.8 (d), 117.2, 53.8, 51.9, 37.4. ^**19**^**F-NMR** (282 MHz, DMSO-*d*_6_): *δ* −62.3. **LR-MS** (ESI^+^): [M+H]^+^ and [M+Na]^+^: 981.6 and 1003.6 *m/z*, (ESI^–^) [M+Br]^–^: 1061.1 *m/z*. **HR-MS** (ESI^+^) calculated for C_42_H_44_F_12_N_10_O_4_ [M+H]^+^: 981.34336 *m/z*, found: 981.34173 *m/z*. **M.P.**: 212.1–218.7°C. **R**_***f***_: 0.0 in CH_2_Cl_2_:CH_3_OH (90:10 *v/v*).

##### Synthesis of 1,1′,1″,1‴-((ethane-1,2-diylbis(azanetriyl))tetrakis(ethane-2,1-diyl))tetrakis(3-(3,5-bis(trifluoromethyl)phenyl)urea) (2)

The tetrakis amine (0.2526 g) was added to a solution of CH_3_CN (dry, degassed, 50 mL) under N_2__(g)_ and allowed to stir at r.t. for 0.5 h to ensure a homogeneous mixture. The reaction was heated to 30°C (303.15 K), and the 3,5-bis(trifluoromethyl)phenyl isocyanate (0.824 mL, 4.4 equiv) was added directly to the reaction via syringe where a precipitate was observed to form immediately. The reaction was allowed to stir for 12 h before the precipitate was filtered off, washed with CH_3_CN (3 × 10 mL), and the filtrate was collected and evaporated under reduced pressure. The solid was washed with CH_2_Cl_2_:CH_3_OH (98:2 *v/v*, 2 × 10 mL), and again the filtrate was evaporated under reduced pressure. The off-white solid then underwent a series of washes; first with (CH_3_CH_2_)_2_O:CH_3_(CH_2_)_4_CH_3_ (60:40 *v/v*, 3 × 10 mL), then freezing CH_3_OH (3 x 10 mL), and finally with deionized H_2_O (excess). The white solid was collected and dried *in vacuo* with a final yield of 0.1628 g (12%).

^**1**^**H-NMR** (600 MHz, DMSO-*d*_6_): *δ* 9.32 (s, 4H), 7.98 (s, 8H), 7.44 (s, 4H), 6.38 (s, 4H), 3.20 (q, *J* = 6.57, 6.27, 6.27 Hz, 8H), 2.64 (s, 4H), 2.62–2.57 (m, 8H). ^**13**^**C-NMR** (126 MHz, DMSO-*d*_6_): *δ* 154.8, 142.5, 130.5 (q), 123.3 (q), 117.0, 113.2, 53.6, 52.2, 37.6. ^**19**^**F-NMR** (282 MHz, DMSO-*d*_6_): *δ* −64.2. **LR-MS** (ESI^+^): [M+H]^+^ and [M+Na]^+^: 1253.3 and 1275.3 *m/z*, (ESI^–^) [M+Br]^–^: 1330.1 *m/z*. **HR-MS** (ESI^+^) calculated for C_46_H_40_F_24_N_10_O_4_ [M+H]^+^: 1253.29235 *m/z*, found: 1253.28695 *m/z*. **M.P.**: 238.8–244.5°C. **R**_***f***_: 0.04 in CH_2_Cl_2_:CH_3_OH (95:5 *v/v*).

##### Synthesis of 1,1′,1″,1‴-((ethane-1,2-diylbis(azanetriyl))tetrakis(ethane-2,1-diyl))tetrakis(3-(4-fluorophenyl)urea) (3)

The tetrakis amine (0.2532 g) was added to a solution of CH_2_Cl_2_ (dry, degassed, 50 mL) under N_2__(g)_ and allowed to stir at r.t. for 0.5 h to ensure a homogeneous mixture. The reaction was heated to 30°C (303.15 K), and the 4-fluorophenyl isocyanate (0.543 mL, 4.4 equiv) was added directly to the reaction via syringe, where a precipitate began to form immediately. The reaction was allowed to stir for 12 h before the white precipitate was filtered, washed with CH_2_Cl_2_ (3 × 10 mL), CH_2_Cl_2_:CH_3_OH (95:5 *v/v*, 3 × 10 mL), deionized H_2_O (3 x 10 mL), and freezing CH_3_OH (3 x 10 mL). The white solid was collected and dried in *vacuo* with a final yield of 0.0085 g (1%).

^**1**^**H-NMR** (400 MHz, DMSO-*d*_6_): *δ* 8.59 (s, 4H), 7.44–7.30 (m, 8H), 7.02 (t, *J* = 8.91, 8.91 Hz, 8H), 6.13 (s, 4H), 3.17 (q, *J* = 6.24, 6.24, 6.17 Hz, 8H), 2.61 (s, 4H), 2.56 (t, *J* = 6.58, 6.58 Hz, 8H). ^**13**^**C-NMR** (151 MHz, DMSO-*d*_6_): *δ* 156.8 (d), 155.4, 136.9, 119.3 (d), 115.1, 115.0, 54.1, 52.1, 37.5. ^**19**^**F-NMR** (282 MHz, DMSO-*d*_6_): *δ* −125.0 (hept). **LR-MS** (ESI^+^): [M+Na]^+^: 803.3 *m/z*, (ESI^–^) [M–H]^–^ and [M+Cl]^–^: 779.5 and 815.5 *m/z*. **HR-MS** (ESI^+^) calculated for C_38_H_44_F_4_N_10_O_4_ [M+H]^+^: 781.35559 *m/z*, found: 781.35456 *m/z*. **M.P.**: 224.0–227.0°C. **R**_***f***_: 0.5 in CH_2_Cl_2_:CH_3_OH (95:5 *v/v*).

##### Synthesis of 1,1′,1″,1‴-((ethane-1,2-diylbis(azanetriyl))tetrakis(ethane-2,1-diyl))tetrakis(3-(perfluorophenyl)urea) (4)

The tetrakis amine (0.2108 g) was added to a solution of CH_3_CN (dry, degassed, 50 mL) under N_2__(g)_ and allowed to stir at r.t. for 0.5 h to ensure a homogeneous mixture. The reaction was heated to 30°C (303.15 K), and the pentafluorophenyl isocyanate (0.52 mL, 4.4 equiv) was added directly to the reaction via a syringe. Precipitate began to form immediately, and the reaction was allowed to stir for 12 h before the precipitate was filtered off, washed with CH_3_CN (3 × 10 mL), and the filtrate was collected and evaporated under reduced pressure. The fine white solid was then washed with (CH_3_CH_2_)_2_O:CH_3_(CH_2_)_4_CH_3_ (60:40 *v/v*, 3 × 10 mL), freezing CH_3_OH (3 x 10 mL), and deionized H_2_O (3 x 10 mL). The white solid was collected and dried *in vacuo* with a final yield of 0.1449 g (15%).

^**1**^**H-NMR** (400 MHz, DMSO-*d*_6_): *δ* 8.40 (s, 6H), 6.53 (s, 7H), 3.15 (q, *J* = 6.25, 6.25, 6.23 Hz, 12H), 2.60–2.50 (m, 12H). ^**13**^**C-NMR** (151 MHz, DMSO-*d*_6_): *δ* 154.5, 142.7 (d), 138.3 (d), 136.7 (d), 114.7, 54.0, 52.3, 38.0. ^**19**^**F-NMR** (282 MHz, DMSO-*d*_6_): *δ* −149.2 (d), −163.3 (t), −166.9 (t). **LR-MS** (ESI^+^): [M+H]^+^ and [M+Na]^+^: 1069.3 and 1091.3 *m/z*, (ESI^–^) [M–H]^–^: 1067.3 *m/z*. **HR-MS** (ESI^+^) calculated for C_38_H_29_F_20_N_10_O_4_ [M+H]^+^: 1069.20484 *m/z*, found: 1069.20285 *m/z*. **M.P.**: 232.2–237.9°C. **R**_***f***_: 0.09 in CH_2_Cl_2_:CH_3_OH (95:5 *v/v*).

##### Synthesis of 1,1′,1″,1‴-((ethane-1,2-diylbis(azanetriyl))tetrakis(ethane-2,1-diyl))tetrakis(3-(4-(trifluoromethyl)phenyl)thiourea) (5)

The tetrakis amine (0.2114 g) was added to a solution of CH_3_CN (dry, degassed, 50 mL) under N_2__(g)_ and allowed to stir at r.t. for 0.5 h to ensure a homogeneous mixture. The reaction was heated to 30°C (303.15 K), and the 4-(trifluoromethyl)phenyl isothiocyanate (0.8107 g, 4.4 equiv) was added directly to the reaction via syringe where a precipitate was observed to form immediately. The reaction was allowed to stir for 8 h before the precipitate was filtered off, washed with CH_3_CN (3 × 10 mL), and the filtrate was collected and evaporated under reduced pressure. The solid was then washed with (CH_3_CH_2_)_2_O:CH_3_(CH_2_)_4_CH_3_ (60:40 *v/v*, 3 × 10 mL), freezing CH_3_OH (1 x 10 mL), (CH_3_CH_2_)_2_O:CH_3_(CH_2_)_4_CH_3_:CH_3_(CH_2_)_3_CH_3_ (50:25:25 *v/v*, 3 x 10 mL), and deionised H_2_O (3 × 10 mL). The off-white solid was collected and dried *in vacuo* with a final yield of 0.2273 g (24%).

^**1**^**H-NMR** (400 MHz, DMSO-*d*_6_): *δ* 9.96 (s, 4H), 7.92 (s, 4H), 7.70 (d, *J* = 8.52 Hz, 8H), 7.62 (d, *J* = 8.57 Hz, 8H), 3.62 (s, 8H), 2.79–2.69 (m, 12H). ^**13**^**C-NMR** (151 MHz, DMSO-*d*_6_): *δ* 180.3, 141.8, 130.0 (dd), 125.0 (d), 121.6, 115.9, 51.9, 51.4, 41.9. ^**19**^**F-NMR** (282 MHz, DMSO-*d*_6_): *δ* −64.0. **LR-MS** (ESI^+^): [M+H]^+^: 1045.4 *m/z*, (ESI^–^) [M+Br]^–^: 1124.9 *m/z*. **HR-MS** (ESI^+^) calculated for C_42_H_44_F_12_N_10_S_4_ [M+H]^+^: 1045.25144 *m/z*, found: 1045.25048 *m/z*. **M.P.**: 204.4–206.8°C. **R**_***f***_: 0.12 in CH_2_Cl_2_:CH_3_OH (90:10 *v/v*).

##### Synthesis of 1,1′,1″,1‴-((ethane-1,2-diylbis(azanetriyl))tetrakis(ethane-2,1-diyl))tetrakis(3-(3,5-bis(trifluoromethyl)phenyl)thiourea) (6)

The tetrakis amine (0.2132 g) was added to a solution of CH_3_CN (dry, degassed, 50 mL) under N_2__(g)_ and allowed to stir at r.t. for 0.5 h to ensure a homogeneous mixture. The reaction was heated to 30°C (303.15 K), and the 3,5-bis(trifluoromethyl)phenyl isothiocyanate (0.735 mL, 4.4 equiv) was added directly to the reaction via syringe where a precipitate was observed to form immediately. The reaction was allowed to stir for 12 h before the white precipitate was filtered off, washed with CH_2_Cl_2_ (3 × 10 mL), deionized H_2_O (3 x 10 mL), (CH_3_)_2_CO (1 x 10 mL), and (CH_3_CH_2_)_2_O:CH_3_(CH_2_)_4_CH_3_:CH_3_(CH_2_)_3_CH_3_ (50:25:25 *v/v*, 3 x 10 mL). The solid was dried *in vacuo* resulting in a final yield of 0.1926 g (16%).

^**1**^**H-NMR** (400 MHz, DMSO-*d*_6_): *δ* 10.18 (s, 4H), 8.21 (s, 8H), 8.04 (s, 4H), 7.64 (s, 4H), 3.76–3.53 (m, 8H), 2.88–2.63 (m, 12H). ^**13**^**C-NMR** (126 MHz, DMSO-*d*_6_): *δ* 180.3, 141.8, 130.0 (dd), 124.3, 121.6, 115.9, 52.0, 51.4, 41.9. ^**19**^**F-NMR** (282 MHz, DMSO-*d*_6_): *δ* −62.7. **LR-MS** (ESI^+^): [M+H]^+^ and [M+Na]^+^: 1317.2 and 1339.2 *m/z*, (ESI^–^) [M+Br]^–^: 1396.58 *m/z*. **HR-MS** (ESI^+^) calculated for C_46_H_40_F_24_N_10_S_4_ [M+H]^+^: 1317.20098 *m/z*, found: 1317.20009 *m/z*. **M.P.**: 199.5–203.4°C. **R**_***f***_: 0.13 in CHCl_3_:CH_3_OH (90:10 *v/v*).

##### Synthesis of 1,1′,1″,1‴-((ethane-1,2-diylbis(azanetriyl))tetrakis(ethane-2,1-diyl))tetrakis(3-(4-fluorophenyl)thiourea) (7)

The tetrakis amine (0.2110 g) was added to a solution of CH_2_Cl_2_ (dry, degassed, 50 mL) under N_2__(g)_ and allowed to stir at r.t. for 0.5 h to ensure a homogeneous mixture. The reaction was heated to 30°C (303.15 K), and the 4-fluorophenyl isothiocyanate (0.6101 g, 4.4 equiv) was added directly to the reaction via a syringe. The reaction was allowed to stir for 8 h before the off-white precipitate was filtered off, washed with CH_2_Cl_2_ (3 × 10 mL), deionized H_2_O (3 x 10 mL), CH_3_OH (3 x 10 mL), and (CH_3_CH_2_)_2_O:CH_3_(CH_2_)_4_CH_3_:CH_3_(CH_2_)_3_CH_3_ (50:25:25 *v/v*, 3 x 10 mL). The solid was dried *in vacuo* resulting in a final yield of 0.0764 g (10%).

^**1**^**H-NMR** (400 MHz, DMSO-*d*_6_): *δ* 9.62 (s, 4H), 7.55 (s, 4H), 7.38 (dd, *J* = 8.74, 4.98 Hz, 8H), 7.15 (t, *J* = 8.67, 8.67 Hz, 8H), 3.59–3.47 (m, 8H), 2.66 (t, *J* = 6.75, 6.75 Hz, 8H), 2.60 (s, 4H). ^**13**^**C-NMR** (126 MHz, DMSO-*d*_6_): *δ* 180.6, 159.0 (d), 135.3, 125.8, 115.3 (d), 52.5, 51.7, 42.0. ^**19**^**F-NMR** (282 MHz, DMSO-*d*_6_): *δ* −120.5. **LR-MS** (ESI^+^): [M+H]^+^ and [M+Na]^+^: 845.4 and 867.3 *m/z*, (ESI^–^) [M−H]^–^ and [M+Cl]^–^: 843.3 and 879.3 *m/z*. **HR-MS** (ESI^+^) calculated for C_38_H_44_F_4_N_10_S_4_ [M+H]^+^: 845.26422 *m/z*, found: 845.26342 *m/z*. **M.P.**: 192.8–194.6°C. **R**_***f***_: 0.14 in CHCl_3_:CH_3_OH (90:10 *v/v*).

##### Synthesis of 1,1′,1″,1‴-((ethane-1,2-diylbis(azanetriyl))tetrakis(ethane-2,1-diyl))tetrakis(3-(perfluorophenyl)thiourea) (8)

The tetrakis amine (0.2559 g) was added to a solution of CH_3_CN (dry, degassed, 50 mL) under N_2__(g)_ and allowed to stir at r.t. for 0.5 h to ensure a homogeneous mixture. The reaction was heated to 30°C (303.15 K), and the pentafluorophenyl isothiocyanate (0.682 mL, 4.4 equiv) was added directly to the reaction via a syringe. The reaction was allowed to stir for 12 h before the precipitate was filtered off, washed with CH_3_CN (3 × 10 mL), and the filtrate was collected and evaporated under reduced pressure. The solid was washed with CH_2_Cl_2_:CH_3_OH (98:2 *v/v*, 3 × 10 mL), and again, the filtrate was evaporated under reduced pressure. The off-white solid then underwent a series of washes; first with (CH_3_CH_2_)_2_O:CH_3_(CH_2_)_4_CH_3_ (60:40 *v/v*, 3 × 10 mL), then freezing CH_3_OH (3 x 10 mL), deionized H_2_O (excess), and finally with CHCl_3_:CH_3_(CH_2_)_4_CH_3_ (80:20 *v/v*, 1 × 10 mL). The white solid was re-dissolved in (CH_3_)_2_CO (3 mL) and loaded onto a preparatory TLC plate which was run in CH_2_Cl_2_:(CH_3_)_2_CO (95:5 *v/v*). The silica containing the product spot at R_*f*_ = 0.23 was collected, stirred for 12 h in (CH_3_)_2_CO (100 mL), filtered, and the filtrate evaporated under reduced pressure. The white solid was then washed with (CH_3_CH_2_)_2_O:CH_3_(CH_2_)_4_CH_3_:CH_3_(CH_2_)_3_CH_3_ (50:25:25 *v/v*, 3 x 10 mL) before being dried *in vacuo* resulting in a final yield of 0.0622 g (5%).

^**1**^**H-NMR** (400 MHz, DMSO-*d*_6_): *δ* 9.33 (s, 4H), 8.08 (s, 4H), 3.55 (s, 8H), 2.93–2.57 (m, 12H). ^**13**^**C-NMR** (126 MHz, DMSO-*d*_6_): *δ* 182.4, 144.0 (d), 140.4, 138.4–135.8 (m), 115.0, 52.3, 52.0, 42.8. ^**19**^**F-NMR** (282 MHz, DMSO-*d*_6_): *δ* −147.3 (d), −159.9 (t), −164.1 (t). **LR-MS** (ESI^+^): [M+H]^+^ and [M+Na]^+^: 1133.2 and 1155.1 *m/z*, (ESI^–^) [M−H]^–^ and [M+Cl]^−^: 1131.7 and 1167.0 *m/z*. **HR-MS** (ESI^+^) calculated for C_38_H_28_F_20_N_10_S_4_ [M+H]^+^: 1133.11347 *m/z*, found: 1133.11392 *m/z*. **M.P.**: 195.4–204.7°C decomposed. **R**_***f***_: 0.23 in CH_2_Cl_2_:CH_3_OH (95:5 *v/v*).

#### 1H-NMR binding studies

A host solution of a constant concentration (0.001 M) was made by dissolving **1**–**8** in DMSO-*d*_6_/0.5% H_2_O. A separate concentrated anionic guest solution (1 M) was made by dissolving the TBA (Cl^−^, NO_3_^−^, H_2_PO_4_^−^, and HP_2_O_7_^3−^) or TEA (HCO_3_^−^) salts into some of the host solution. A more dilute anionic guest solution (0.1 M) was made by diluting some of the conc. guest solution with the host stock solution. An initial ^1^H-NMR spectrum was acquired of the host-receptor (0.5 mL) before aliquots of the guest solutions were incrementally titrated into the host solution. At first, the dilute anionic guest solution was used and once approx. 10 equivs. of the guest was added to the system, the conc. guest solution was used to saturate the system. A guest equivalence of approx. 400–600 equiv of the guest was added to observe the second binding event. The chemical shifts (ppm) of the proton peaks involved in binding were recorded after all spectra had been referenced to the DMSO-*d*_6_ proton peak (2.50 ppm), and anion binding constants (*K*_a_) of the host:guest complexes that formed were derived using the BindFit web applet at www.supramolecular.org.^19^^,^^20^^,^^21^

#### Covariance of fit calculations for anion binding constants

The ^1^H-NMR binding studies of the fluorinated tetrapodal transporters with the 1:1 and 1:2 host:guest covariance of fit (cov_fit_) calculated using Equations 1 and 2 for all anion-binding data that could be fit to either a 1:1 or 1:2 host:guest binding isotherm (Table S1).

(Equation 1) $${cov}_{res}=y-y_{res}$$

The equation used to derive the covariance of the residual data.

Where cov_res_ is the covariance of the residual data, *y* is the tracked proton shift (in ppm), and *y*_res_ is the calculated residual value after fitting the peak shift data to a 1:1 or 1:2 binding model.

(Equation 2) $${cov}_{fit}={cov}_{cal}-{cov}_{res}$$

The equation used to calculate the covariance of fit from the calculated covariance after fitting and residual covariance.

Where cov_fit_ is the overall covariance of fit and cov_cal_ is the calculated covariance after fitting to either the 1:1 or 1:2 binding model.

If the data fits both a 1:1 and 1:2 host:guest binding isotherm, the enhancement factor for the covariance of fit (Fcov_fit_) can be calculated using Equation 3 (Table S1).

(Equation 3) $$F{cov}_{fit}=\frac{1:1\;{cov}_{fit}}{{1:2\;cov}_{fit}}$$

The equation used to calculate the covariance of fit enhancement factor.

Where Fcov_fit_ is the covariance of fit enhancement factor, 1:1 cov_fit_ is the covariance of fit for the data when fitted to a 1:1 host:guest binding model, and 1:2 cov_fit_ is the covariance of fit when the binding data is fit to a 1:2 host:guest binding model.

If 1:2 host:guest binding is observed, the overall binding constant (β_12_) can be calculated using Equation 4 (Table S1).

(Equation 4) $$\beta_{12}=K_{11}\times K_{12}$$

The equation used to calculate the overall binding constant.

Where *β*_12_ is the overall binding constant, *K*_11_ is the binding constant of the first guest binding event, and *K*_12_ is the binding constant of the second binding event.

#### Preparation of POPC vesicles

Large unilamellar POPC vesicles (200 nm) were prepared by first making a stock solution of POPC (1 g, 37.5 mM) in CHCl_3_ (10 mL). A predetermined volume (mL) of the POPC stock solution (37.5 mM) was taken and slowly evaporated under reduced pressure to form an even film of POPC. The initial lipid film was further dried in *vacuo* for at least 12 h before the next step. The lipid film was rehydrated using a 1:1 *v/v* ratio of an assay-specific internal solution, e.g., 4 mL of the POPC stock solution was dried, so 4 mL of the desired internal solution was used to rehydrate the lipid film. The solution was then vortexed for 20 min to ensure complete rehydration. The suspension was then repeatedly frozen in liquid N_2 (l)_ and then thawed in lukewarm H_2_O nine times before allowing the frozen unilamellar vesicle suspension to rest and warm to r.t. over 0.5 h. The suspension was then subject to twenty-five extrusion cycles through a 200 nm polycarbonate membrane. The resulting vesicle suspension was then subject to either dialysis in a sodium phosphate buffer solution (5 mM) or Sephadex G-25 size-exclusion chromatography using the desired assay-specific external solution. The collected vesicle solution was diluted with the desired external solution affording a vesicle stock solution of a known concentration to be used in subsequent assays.

#### The chloride/nitrate exchange assay

Large unilamellar vesicles (200 nm) were prepared with an internal solution of NaCl (489 mM) and a sodium phosphate salt buffer of NaH_2_PO_4_·2H_2_O (5 mM) and Na₂HPO₄ (5 mM) adjusted to pH 7.2. The vesicles were dialyzed using the same sodium phosphate buffer (5 mM), after which the vesicles were resuspended in an external solution of the sodium phosphate buffer (5 mM) and NaNO_3_ (489 mM) adjusted to pH 7.2. The system to be tested was prepared by first adding vesicles (1 mM) into the external solution (5 mL). A stock DMSO solution of the transporter (50 mM) was prepared and diluted to different concentrations as needed. A chloride ion-sensitive electrode (ISE) was used to monitor the transporter-facilitated Cl^−^ efflux when the transporter (10 μL) was added to the assay system at *t* = 0 s. After *t* = 300 s, Triton X-100 (50 μL) was added to lyse the vesicles. At *t* = 420 s, the 100% Cl^−^ efflux value was recorded, and the experiment was stopped.

#### The HPTS transport selectivity assay

Large unilamellar POPC vesicles (200 nm) were prepared to contain an internal solution of HPTS (1 mM) and NaCl (100 mM), buffered to pH 7.0 with HEPES (10 mM). The vesicles were suspended in an external solution of NaCl (100 mM) and HEPES (10 mM), which was also buffered to pH 7.0 after size exclusion chromatography using Sephadex® G-25 run with the external solution. The system to be tested was prepared by first adding vesicles (0.1 mM) into the external solution (2.5 mL). A stock DMSO solution of the transporter (20 mM) was prepared and diluted to different concentrations as needed. At *t* = −30 s, a NaOH (0.5 M, 25 μL) base pulse was added to the system to increase the external pH from 7 to 8. The experiment was allowed to run for approx. 30 s to make sure no vesicle leakage was occurring. The transporters (5 μL) were added to the assay system at varying concentrations at *t* = 0 s which contained untreated vesicles to test anion transport in the presence of membrane-embedded fatty acids. At *t* = 220 s, Triton™ X-100 (50 μL) was added to lyse the vesicles, and at *t* = 280 s, the fractional fluorescence intensity (*I*_f_) of HPTS at 100% chloride efflux was recorded. The transporters (1 mol % with respect to the lipid concentration) were also screened for the ability to facilitate H^+^/Cl^−^ transport by adding the weak acid protonophore CCCP (1 mol %). Similarly, the ability of transporters (1 mol %) to mediated H^+^/Cl^−^ co-transport was analyzed in the absence of membrane-embedded fatty acids through vesicle pre-treatment with BSA (1 mol %).

#### The modified HPTS transport selectivity assay

Large unilamellar vesicles (200 nm) were prepared and contained an internal solution of HPTS (1 mM), HEPES (10 mM), and NaCl (100 mM) buffered to pH 7.0. The vesicles were suspended in an external solution of NaX (100 mM, where X = Cl^−^, Br^−^, NO_3_^−^, ClO_4_^−^, and I^−^) and HEPES (10 mM), which was also buffered to pH 7.0 after size exclusion chromatography using Sephadex® G-25. The system to be tested was prepared by first adding vesicles (0.1 mM) into the external solution (2.5 mL). At *t* = −30 s, the experiment was allowed to run for ∼30 s to ensure no vesicle leakage was occurring. At *t* = 0 s, the transporters (0.5 or 1.0 mol %, with respect to the lipid concentration) were added to the assay system, and the fractional fluorescence intensity of the system was recorded until the recorded data reached an inflexion point. The data was then normalized, giving the change in pH described by Equation 5.

(Equation 5) $$\Delta pH={pH}_{OUT}-{pH}_{IN}$$

The equation used to normalize the HPTS data to give the change in pH.

Where ΔpH is the total change in pH of the system, pH_OUT_ is the extravesicular pH, and pH_IN_ is the intravesicular pH value.

#### Hill analysis

Hill analysis was performed by first plotting the transporter concentration (mol %, with respect to the lipid concentration) against either the percentage Cl^−^ efflux (%, for the Cl^−^/NO_3_^−^ exchange or cationophore coupled transport assays) at *t* = 270 s or the fractional fluorescence intensity (*I*_f_) at *t* = 200 s, for the HPTS transport assay. The data were then fit to the Hill equation (Equation 6) to calculate the transporter concentration, which facilitates 50% of the observed transport (EC_50_, mol %) and the host:guest stoichiometry using the Hill coefficient (*n*).

(Equation 6) $$y=y_{0}+\left(y_{1}-y_{0}\right)\frac{x^{n}}{\left(k^{n}+x^{n}\right)}$$

The Hill equation.

Where *x* is the transporter concentration (mol %), *y* is either the percentage Cl^−^ efflux (%) or fractional fluorescence intensity (*I*_f_) at *t* = 270 or 200 s, *y*_0_ is the value of the minimum observed Cl^−^ efflux (%) or the *I*_f_ value for the DMSO control, *y*_1_ is the value of the maximum observed Cl^−^ efflux (%) or the *I*_f_ value for the transporter, *k* is the EC_50_ (in mol %), and *n* is the Hill coefficient.

#### Initial rate constant calculations

The initial Cl^−^ transport rates (*k*_ini_, s^−1^) facilitated by the transporters were calculated by non-linear curve fitting of the recorded Cl^−^ efflux (%) vs time *t* (s) data using a two-phase exponential decay function (Equation 7).

(Equation 7) $$y=y_{0}+A_{1}e^{\frac{-x}{t_{1}}}+A_{2}e^{\frac{-x}{t_{2}}}$$

A two-phase exponential decay function.

Where *x* is time *t* (s), *t*_1_ and *t*_2_ are time constants, *y* is the percentage of Cl^−^ efflux (%), *y*_0_ is the curve offset, and *A*_1_ and *A*_2_ are the amplitude constants.

The fitted data was then substituted into a second function (Equation 8) to calculate the *k*_ini_ value of each transporter.

(Equation 8) $$k_{ini}=\left(\frac{dy}{dx}\right)x=0=-\frac{A_{1}}{t_{1}}-\frac{A_{2}}{t_{2}}$$

The equation used to calculate *k*_ini_ (s^−1^) of the transporters.

Where *t*_1_ and *t*_2_ and *A*_1_ and *A*_2_ are the time and amplitude constants, respectively, derived from non-linear curve fitting of the Cl^−^ efflux (%) vs time (s) transport data.

#### X-Ray crystallography

Collection of the crystallographic data of **8** and **8·**2NO_3_ was done at The Sydney Pharmacy School, Faculty of Medicine and Health, The University of Sydney, Australia, using an Agilent Supernova X-ray Diffractometer with a CCD Atlas detector at Mo∖Kα wavelength (0.7107 Å) and Cu∖Kα (1.54184 Å), respectively. Each specimen was cooled to −123.15°C (150 K) throughout the collection. A full sphere of data was collected for both samples using 1° ω-scans with the crystal-to-detector distance maintained at 5.3 cm for both crystals. Coverage of reciprocal space was obtained for **8** by positioning the detector arm between 3.65(1)° and 76.32(2)° in 2Θ. The coverage of reciprocal space was obtained for **8·**2NO_3_ by positioning the detector arm between 2.784(1)° and 74.49(1)° in 2Θ. Integration and reduction of the raw reflection data was performed using CrysAlis^Pro^ for both **8** and **8·**2NO_3_. The spherical models of **8** and **8·**2NO_3_ were solved by the dual phase method in SHELX-T, and the models were refined by a full matrix least squared refinement in SHELXL-2015 based on *F*^2^.^32^^,^^33^^,^^34^^,^^35^ Selected refinement details can be found in Table S2. Further information on data collection, bond lengths, and bond angles of each structure can be found in Tables S3–S15. The full crystallographic data can be obtained from the Cambridge Structure Database (CSD), CSD №: 2201398 and CSD №: 32201397, for **8** and **8·**2NO_3_, respectively.

## Data Availability

• Data which has been reported in this work is available from the lead contact upon request. • No original code was used or reported in this work. • Further information needed to reanalyze the data reported in this work is available from the lead contact upon request.
