# Supplementary material for: Fluorinated tetrapodal anion transporters
Source: iScience. 2023 Jan 23;26(2):105988. doi: 10.1016/j.isci.2023.105988 (PMC9932467; doi:10.1016/j.isci.2023.105988)
Supplement: Document S1. Figures S1–S108 and Tables S1–S15 [file mmc1.pdf]

iScience, Volume *26*

Supplemental information

Fluorinated tetrapodal anion transporters

Alexander M. Gilchrist, Xin Wu, Bryson A. Hawkins, David E. Hibbs, and Philip A. Gale

Table of Contents:

S1. General experimental: 2
S2. ^{1}H-, ^{13}C-, and ^{19}F-NMR characterization of compounds **1**–**8**: 3
S3. Transport studies: 15
S3.1 The chloride/nitrate transport exchange assay: 15
S3.2 The HPTS transport selectivity assay - fatty acid H^{+}/Cl^{-} cotransport: 19
S3.3 The modified HPTS transport selectivity assay: selectivity among anions 29
S4. Structure-activity relationships: 33
S4.1 Initial rate constants from the chloride/nitrate exchange assay: 33
S4.2 Initial rate constants from the HPTS transport selectivity assay: 37
S5. ^{1}H-NMR binding studies: 47
S5.1 The calculated covariance of fit, enhancement factors, and overall binding constants: 47
S5.2 Anion binding studies of transporter **1**: 48
S5.3 Anion binding studies of transporter **2**: 52
S5.4 Anion binding studies of transporter **3**: 55
S5.5 Anion binding studies of transporter **4**: 60
S5.6 Anion binding studies of transporter **5**: 64
S5.7 Anion binding studies of transporter **6**: 68
S5.8 Anion binding studies of transporter **7**: 72
S5.9 Anion binding studies of transporter **8**: 76
S6. Single crystal X-Ray diffraction: 79
Crystal structure determination of **8**: 86
Refinement model description of **8**: 86
Crystal structure determination of **8·2NO$_3$**: 95
Refinement model description of **8·2NO$_3$**: 95
S7. References: 96

S1. General experimental:

The synthesis and characterization of precursor compounds *N,N',N'',N'''*-((ethane-1,2-diylbis(azanetriyl))tetrakis(ethane-2,1-diyl))tetrakis(4-methylbenzenesulfonamide), N^1,N^1,N^2,N^2-tetrakis(2-(bromo-λ^5-azaneyl)ethyl)ethane-1,2-diamine, and N^1,N^1,N^2,N^2-tetrakis(2-aminoethyl)-1,2-ethanediamine has been previously reported.[S1-4] The synthesis and characterization of non-fluorinated tetrapodal anion transporters **9** and **10** have been previously reported.[S5]

S2. ^{1}H-, ^{13}C-, and ^{19}F-NMR characterization of compounds 1–8:

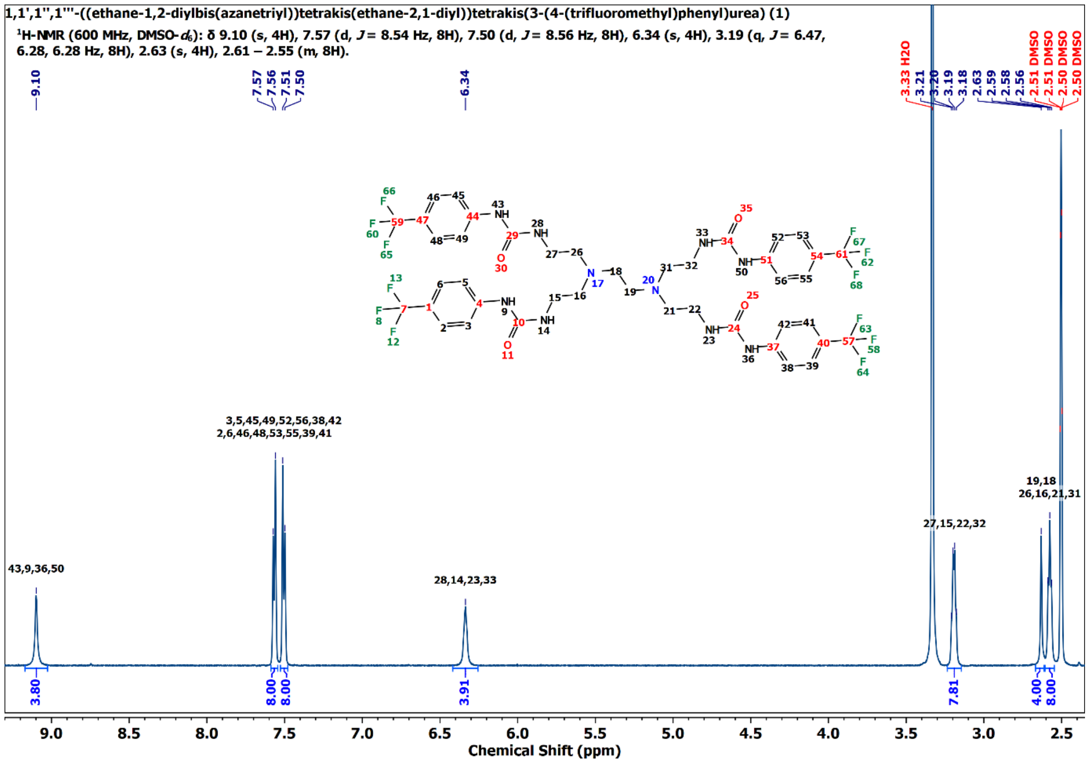

Figure S1. The ^{1}H-NMR (600 MHz) spectrum of transporter **1** in DMSO-d_6 at 298 K, related to the STAR Methods section.

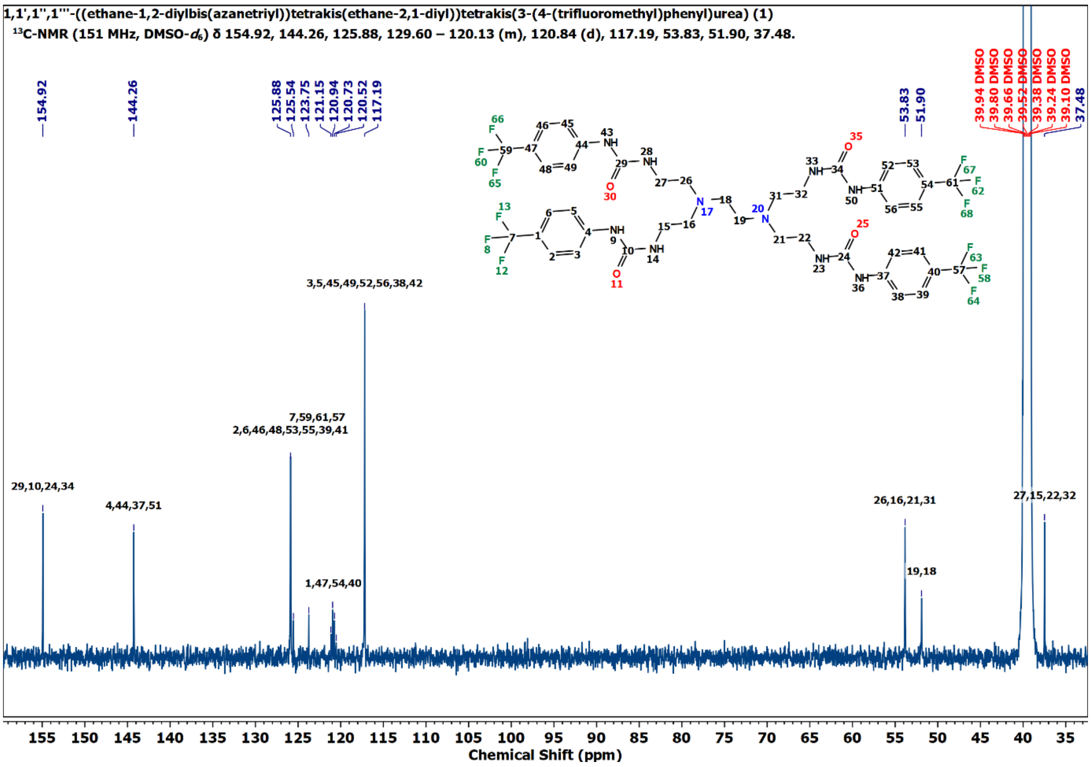

Figure S2. The ^{13}C-NMR (151 MHz) spectrum of transporter **1** in DMSO-d_6 at 298 K, related to the STAR Methods section.

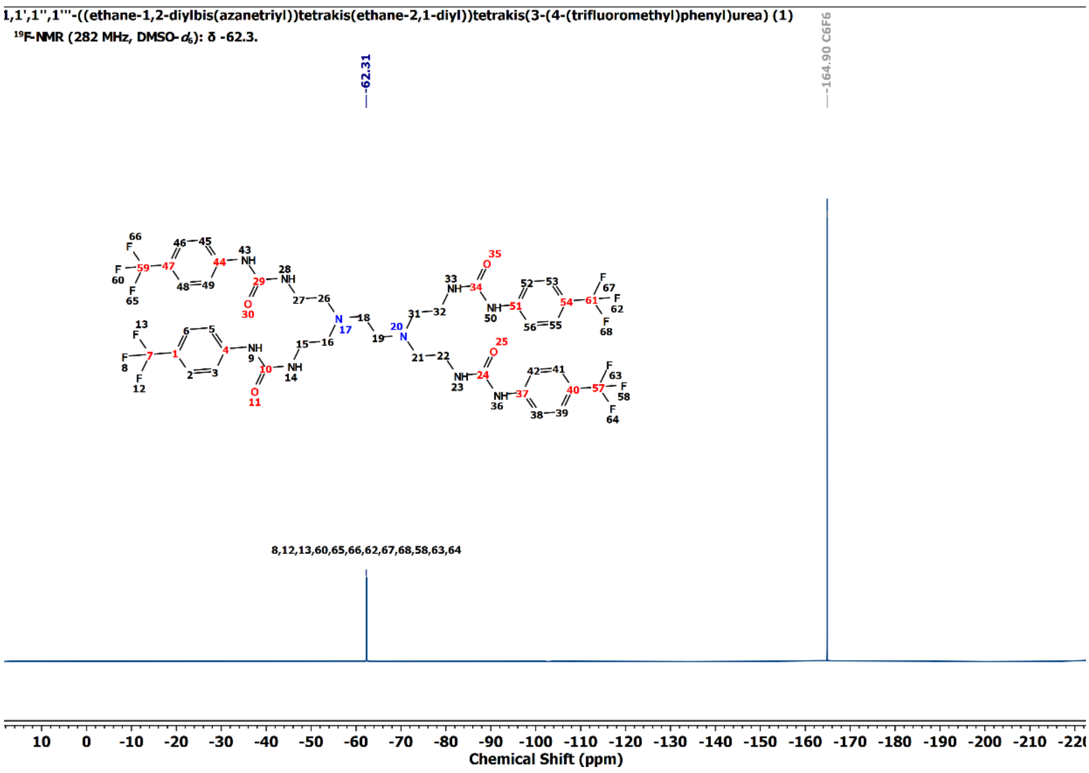

Figure S3. The ^{19}F-NMR (282 MHz) spectrum of transporter **1** in DMSO-d_6 at 298 K, related to the STAR Methods section.

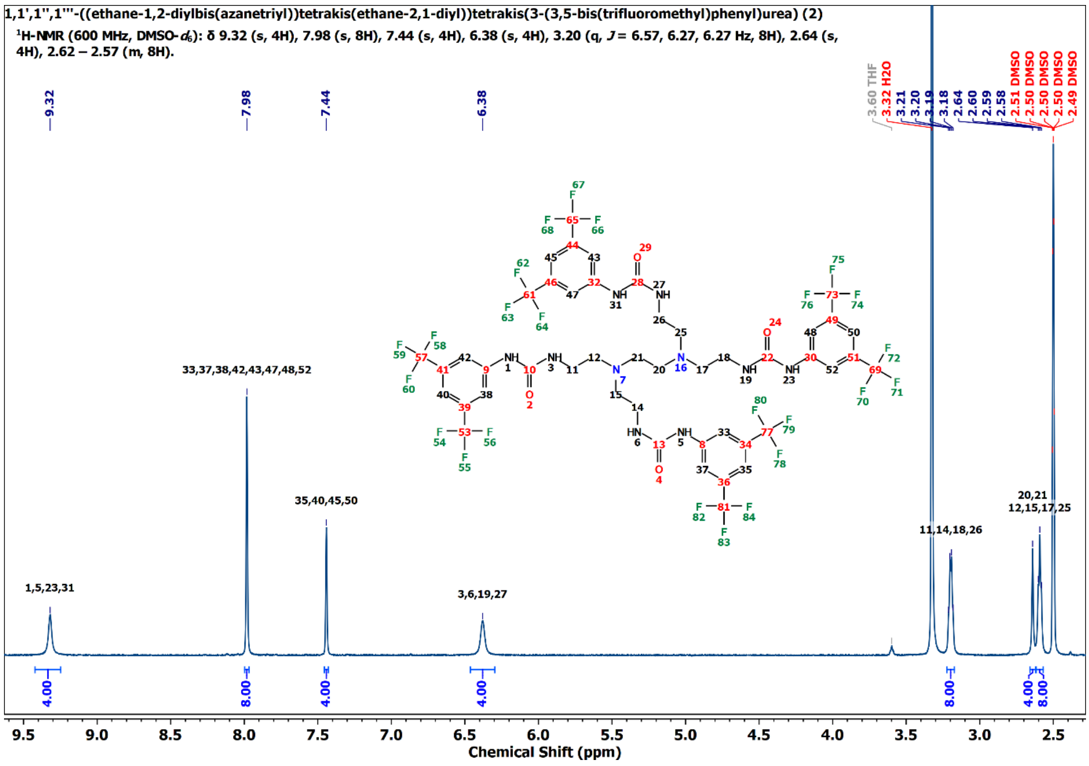

Figure S4. The ^{1}H-NMR (600 MHz) spectrum of transporter **2** in DMSO-d_6 at 298 K, related to the STAR Methods section.

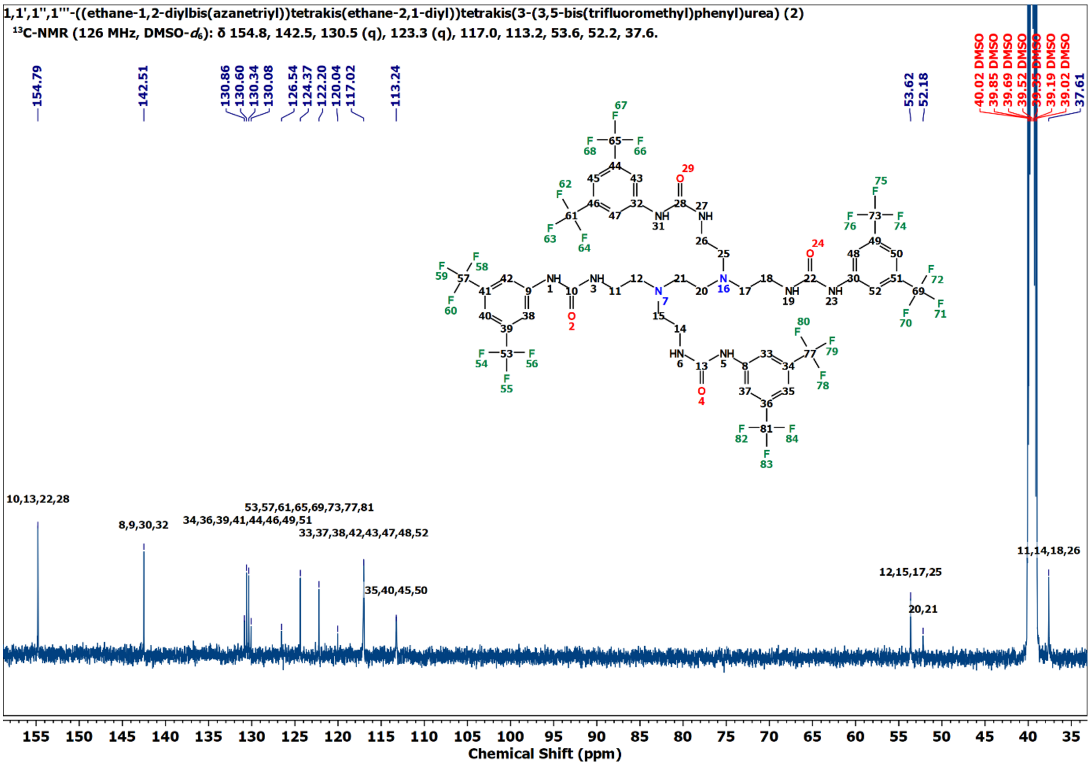

Figure S5. The ^{13}C-NMR (126 MHz) spectrum of transporter **2** in DMSO-d_6 at 298 K, related to the STAR Methods section.

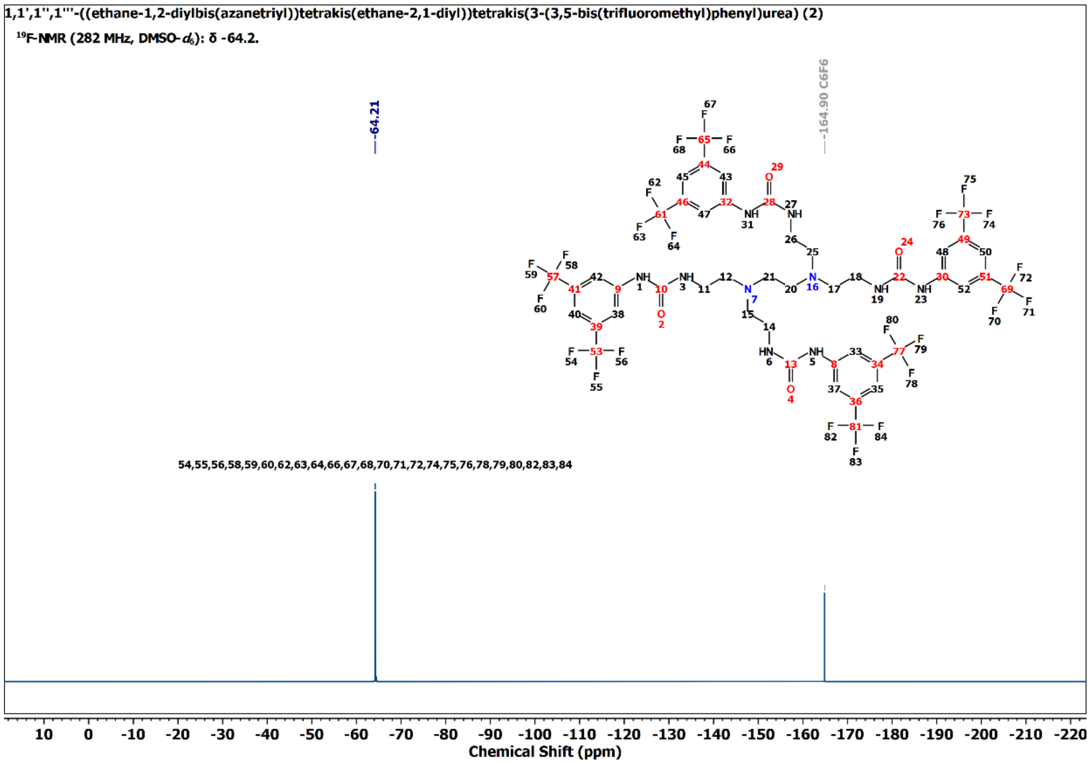

Figure S6. The ^{19}F-NMR (282 MHz) spectrum of transporter **2** in DMSO-d_6 at 298 K, related to the STAR Methods section.

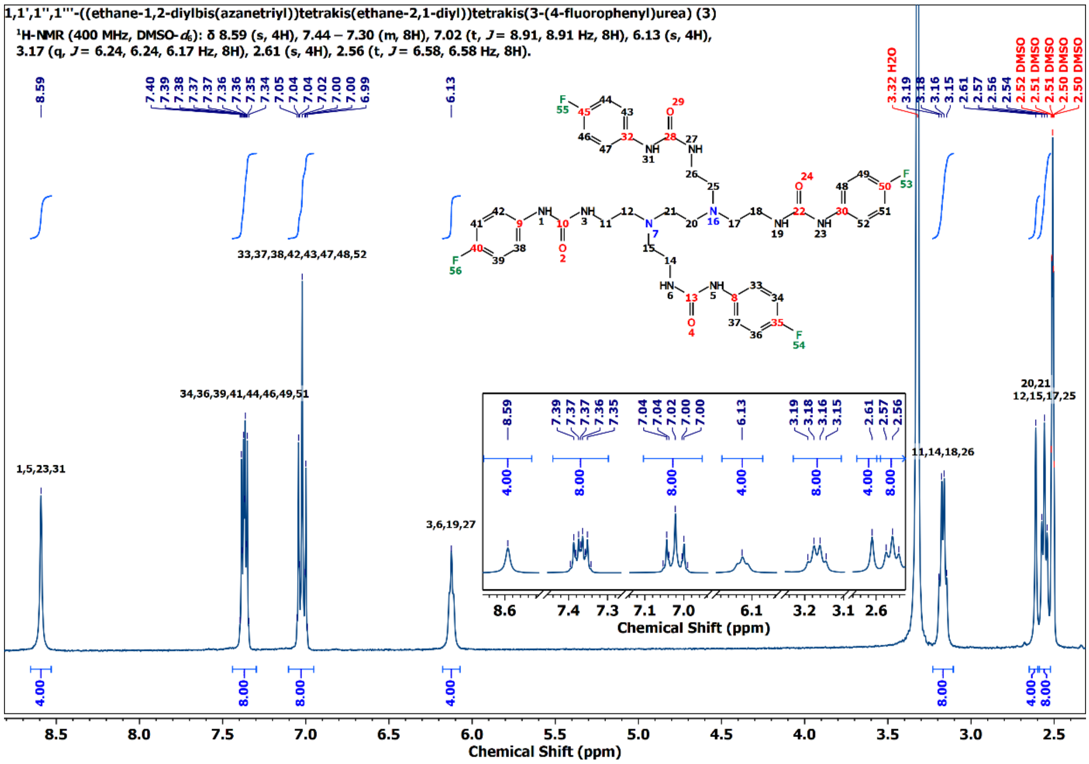

Figure S7. The ^{1}H-NMR (400 MHz) spectrum of transporter **3** in DMSO-d_6 at 298 K, related to the STAR Methods section.

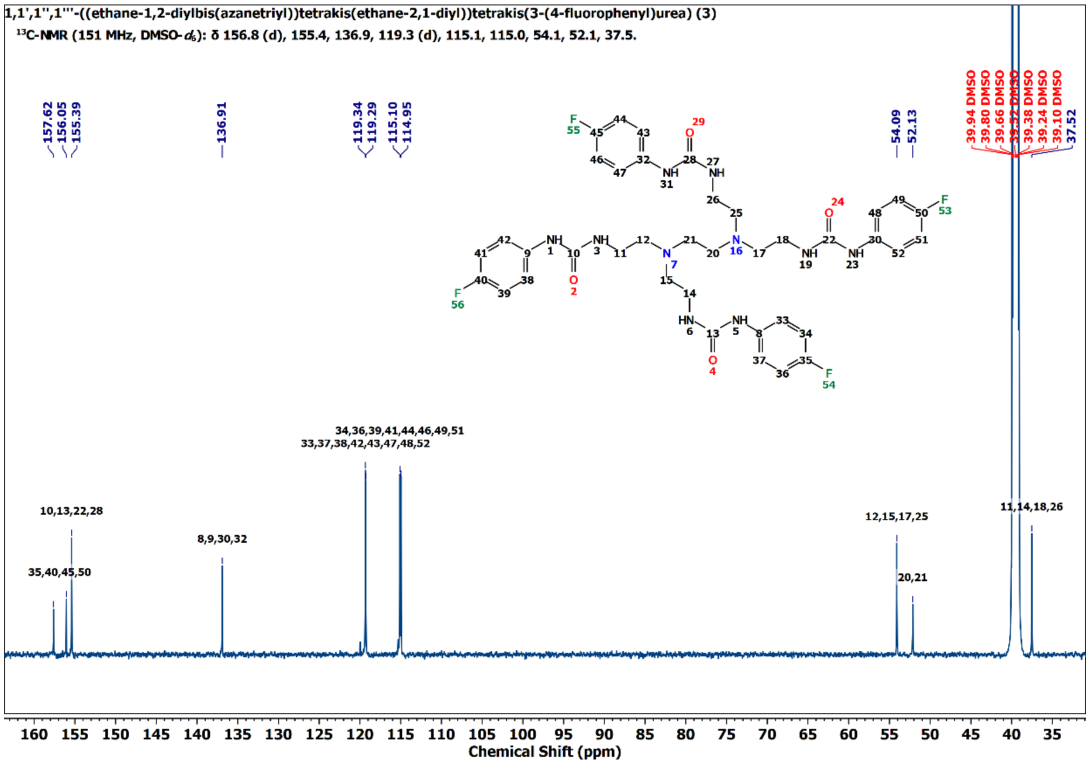

Figure S8. The ^{13}C-NMR (151 MHz) spectrum of transporter **3** in DMSO-d_6 at 298 K, related to the STAR Methods section.

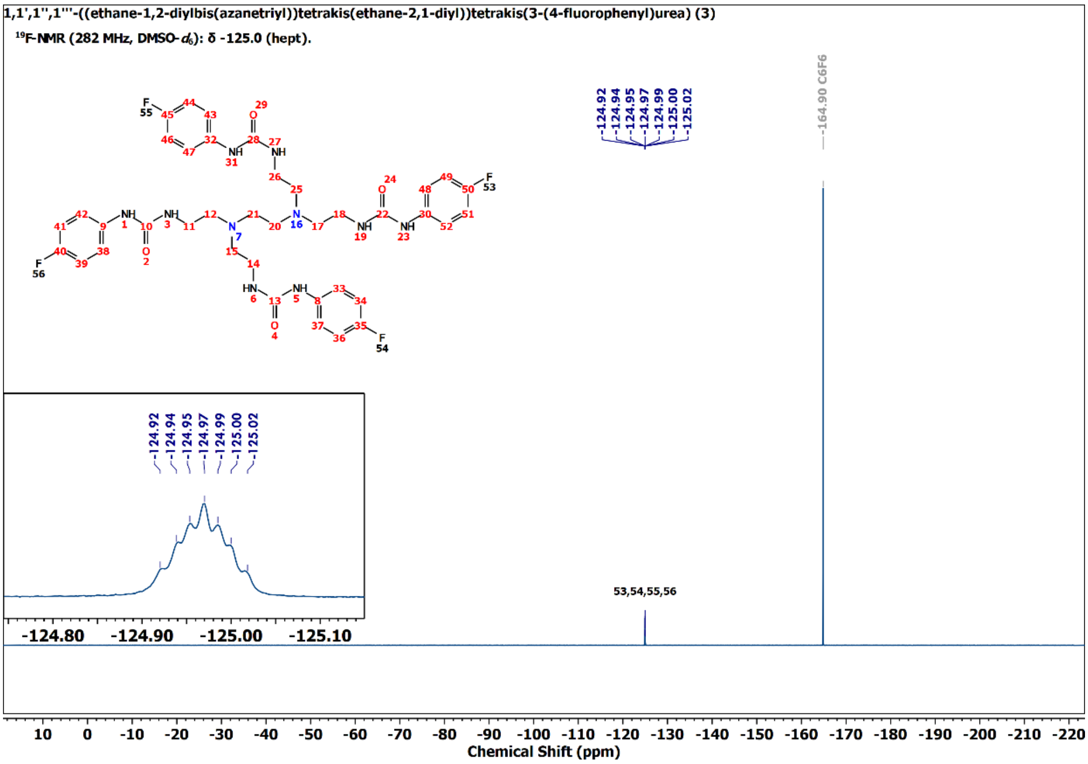

Figure S9. The ^{19}F-NMR (282 MHz) spectrum of transporter **3** in DMSO-d_6 at 298 K, related to the STAR Methods section.

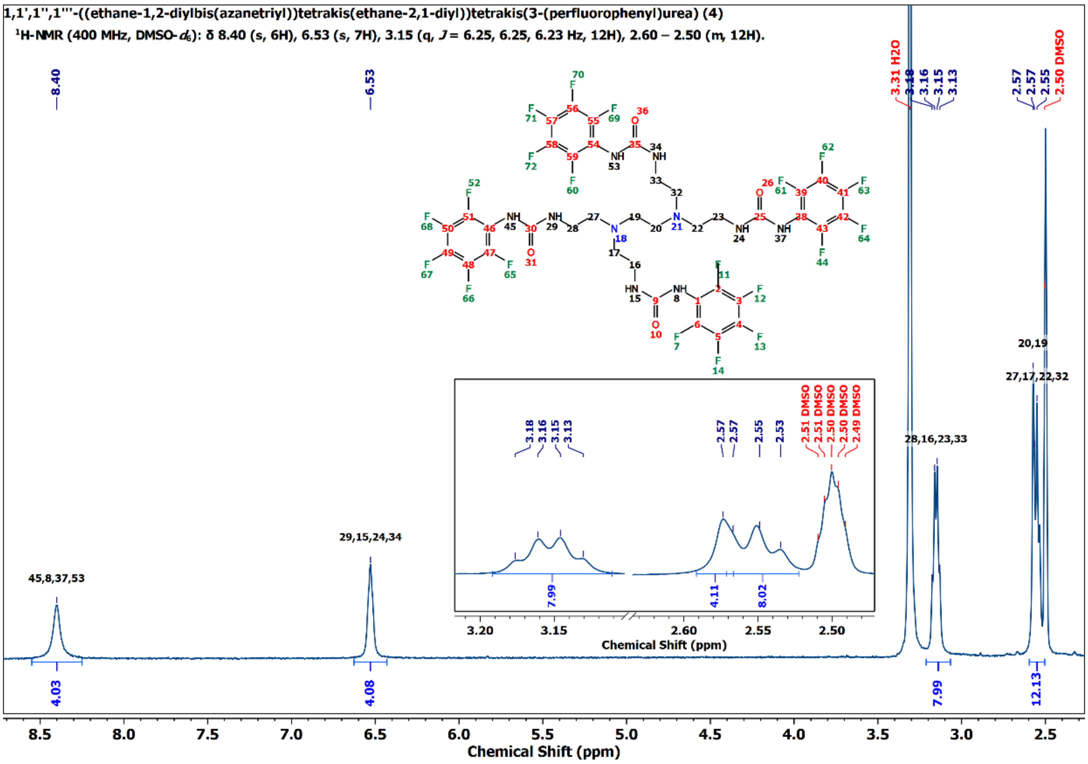

Figure S10. The ^{1}H-NMR (400 MHz) spectrum of transporter **4** in DMSO-d_6 at 298 K, related to the STAR Methods section.

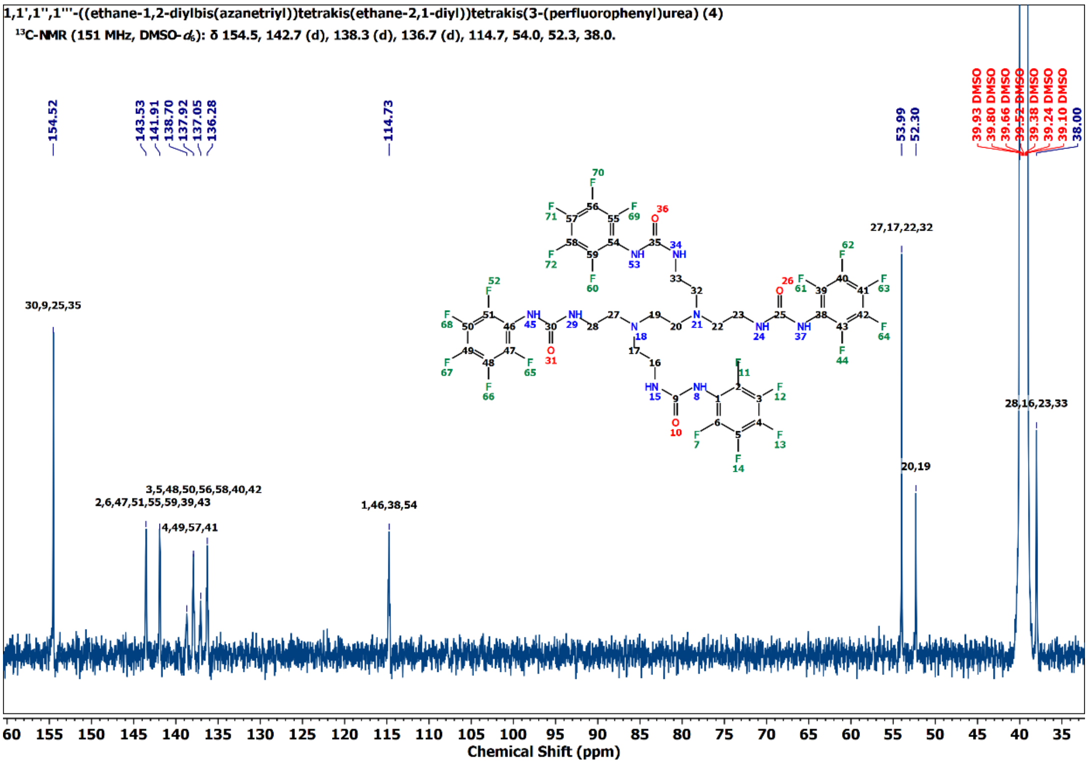

Figure S11. The ^{13}C-NMR (151 MHz) spectrum of transporter **4** in DMSO-d_6 at 298 K, related to the STAR Methods section.

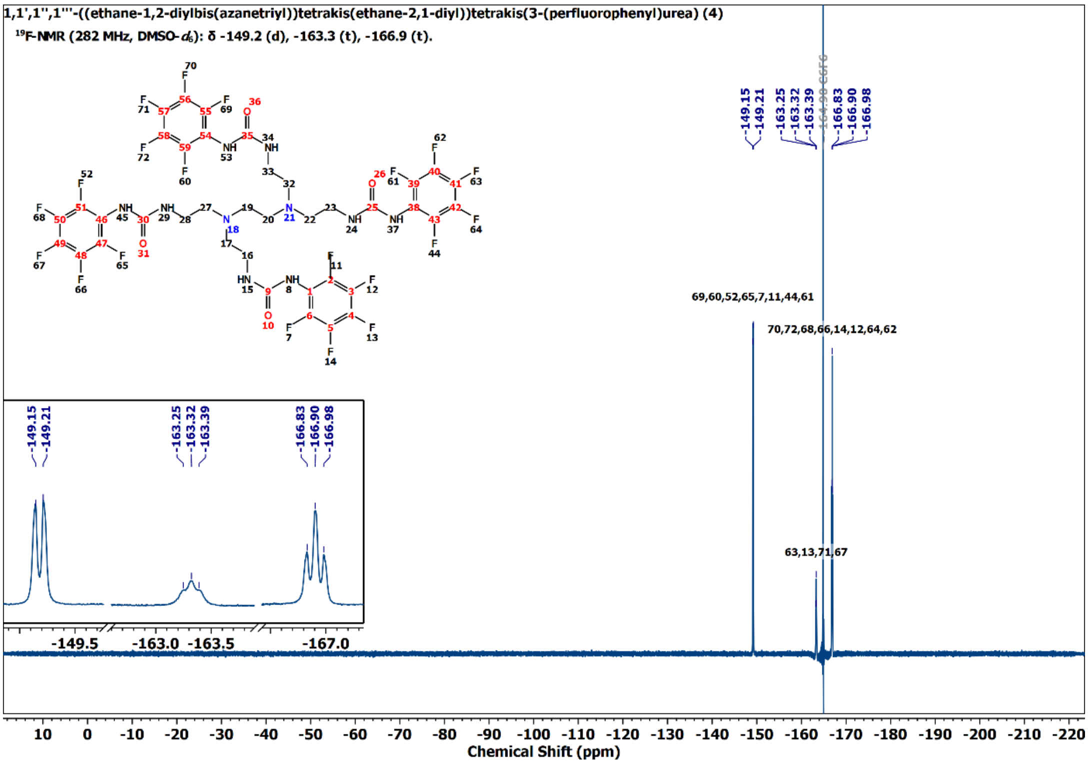

Figure S12. The ^{19}F-NMR (282 MHz) spectrum of transporter **4** in DMSO-d_6 at 298 K, related to the STAR Methods section.

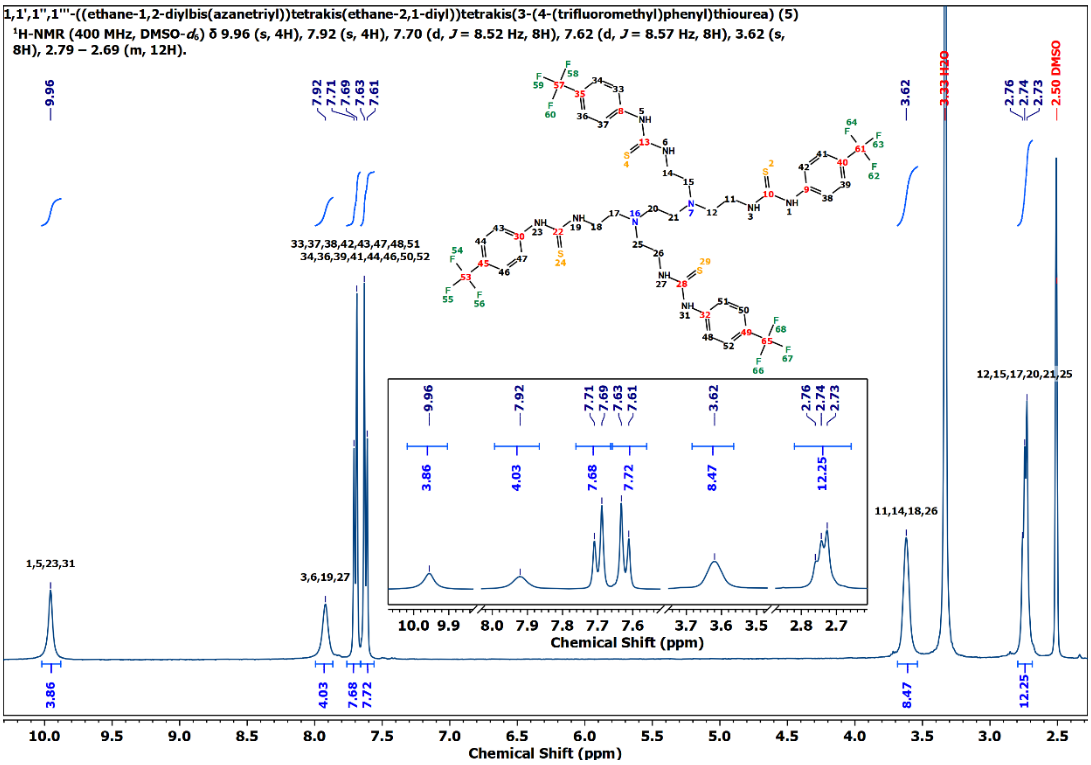

Figure S13. The ¹H-NMR (400 MHz) spectrum of transporter **5** in DMSO-*d*6 at 298 K, related to the STAR Methods section.

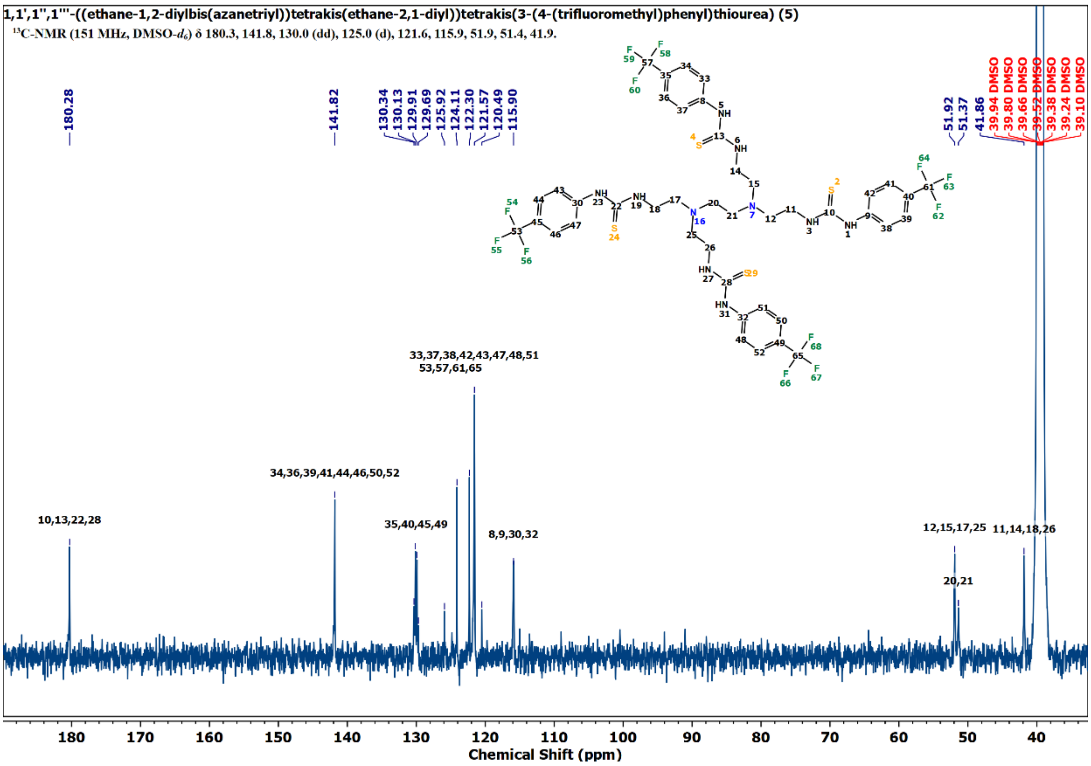

Figure S14. The ¹³C-NMR (151 MHz) spectrum of transporter **5** in DMSO-*d*6 at 298 K, related to the STAR Methods section.

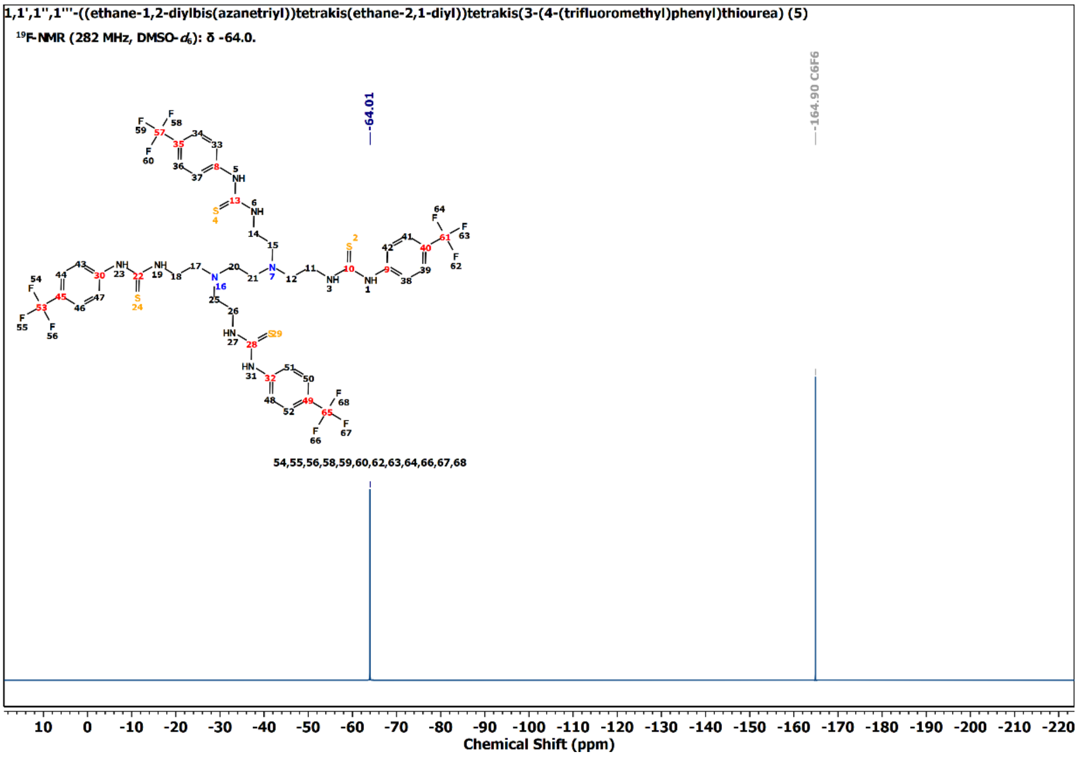

Figure S15. The ^{19}F-NMR (282 MHz) spectrum of transporter **5** in DMSO-d_6 at 298 K, related to the STAR Methods section.

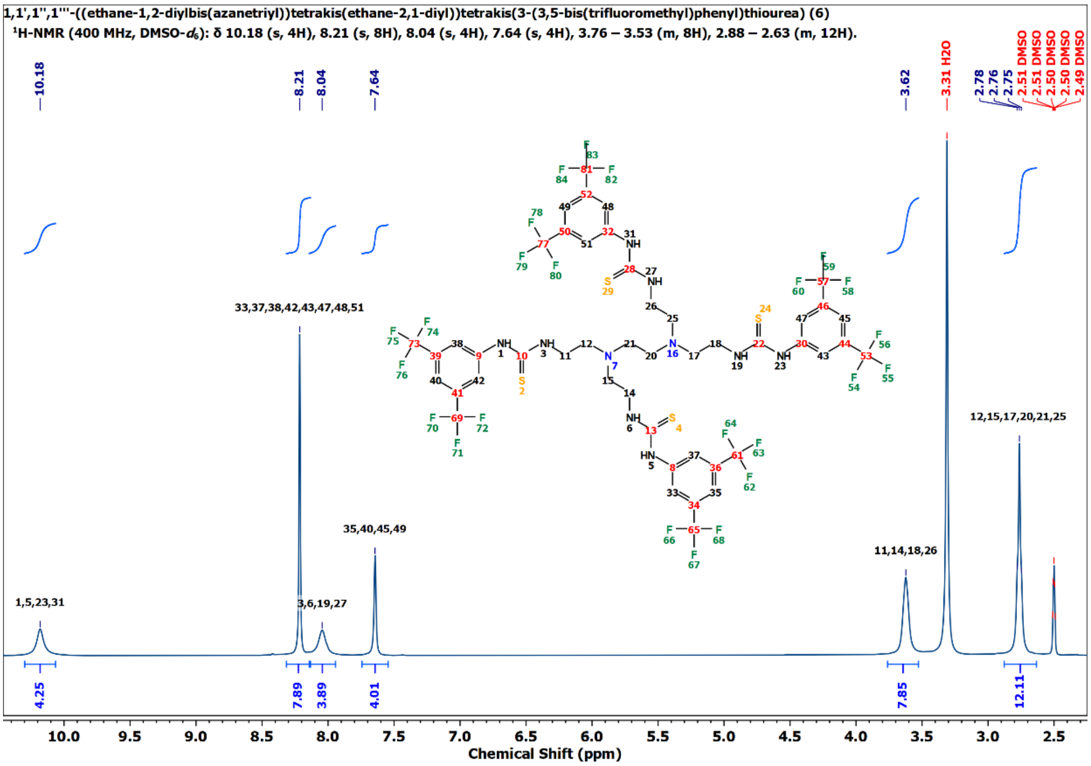

Figure S16. The ^{1}H-NMR (400 MHz) spectrum of transporter **6** in DMSO-d_6 at 298 K, related to the STAR Methods section.

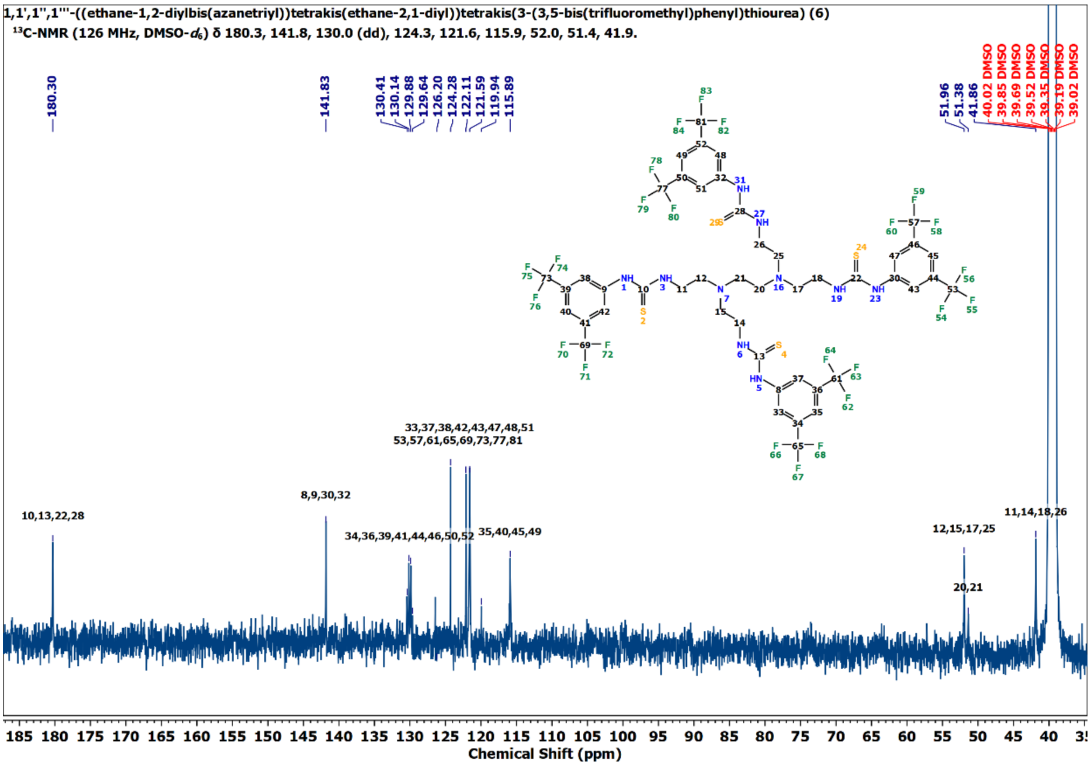

Figure S17. The ^{13}C-NMR (126 MHz) spectrum of transporter **6** in DMSO-d_6 at 298 K, related to the STAR Methods section.

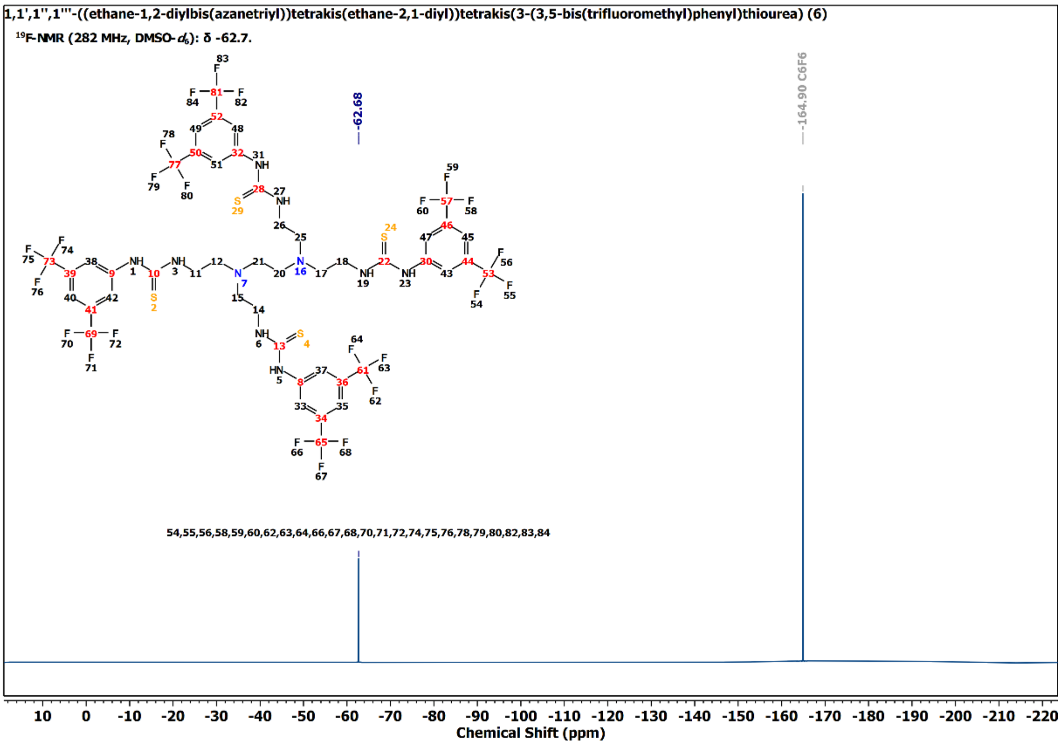

Figure S18. The ^{19}F-NMR (282 MHz) spectrum of transporter **6** in DMSO-d_6 at 298 K, related to the STAR Methods section.

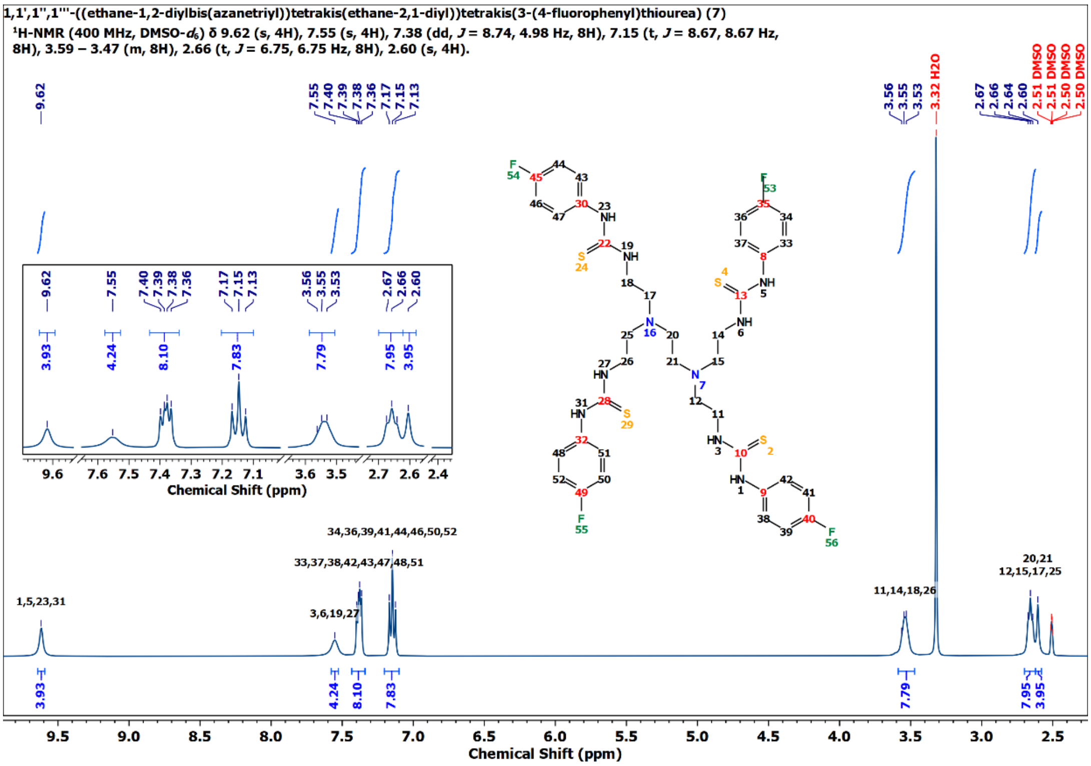

Figure S19. The ^{1}H-NMR (400 MHz) spectrum of transporter **7** in DMSO-*d*$_6$ at 298 K, related to the STAR Methods section.

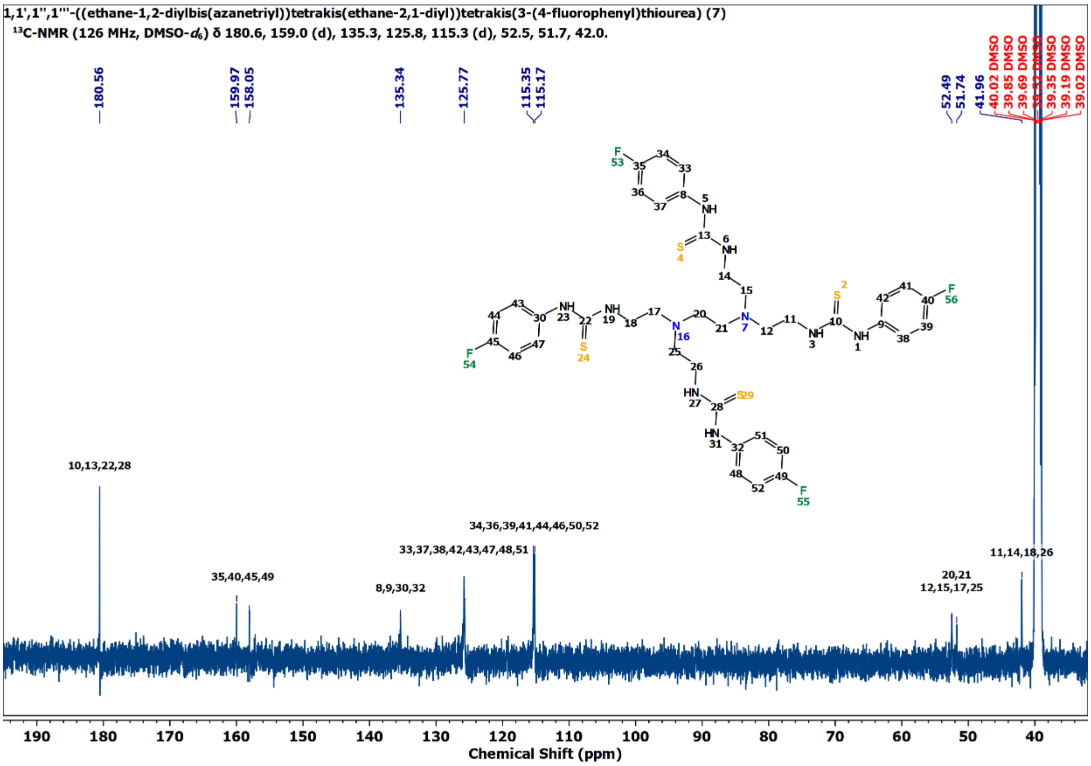

Figure S20. The ^{13}C-NMR (126 MHz) spectrum of transporter **7** in DMSO-*d*$_6$ at 298 K, related to the STAR Methods section.

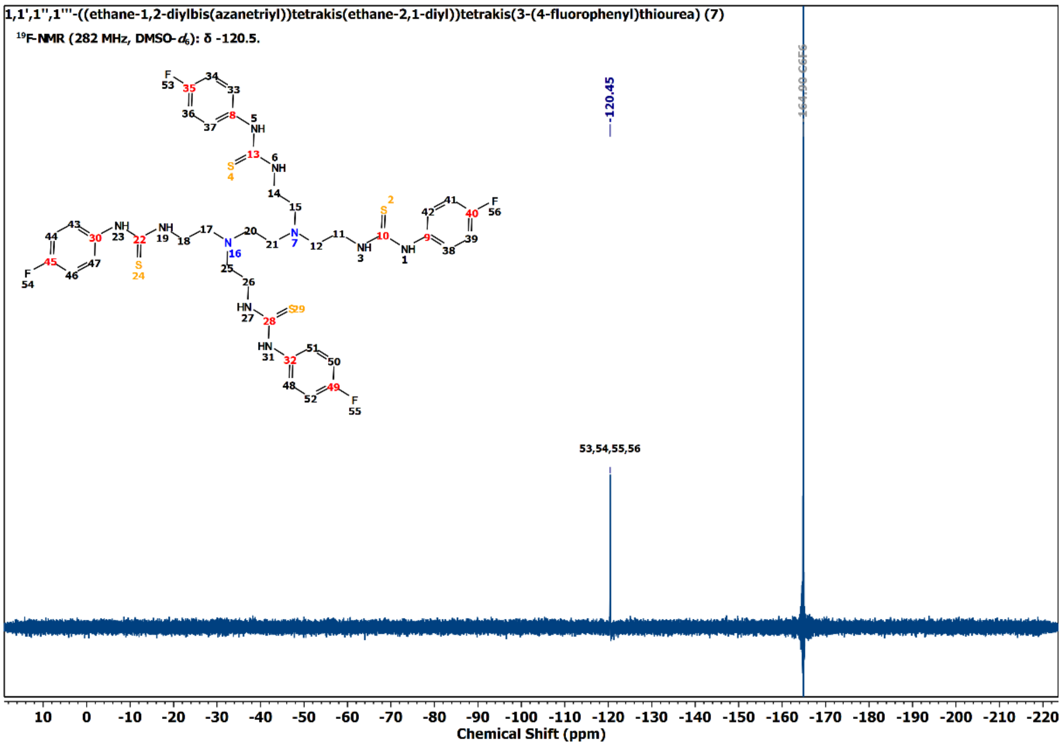

Figure S21. The ^{19}F-NMR (282 MHz) spectrum of transporter **7** in DMSO-d_6 at 298 K, related to the STAR Methods section.

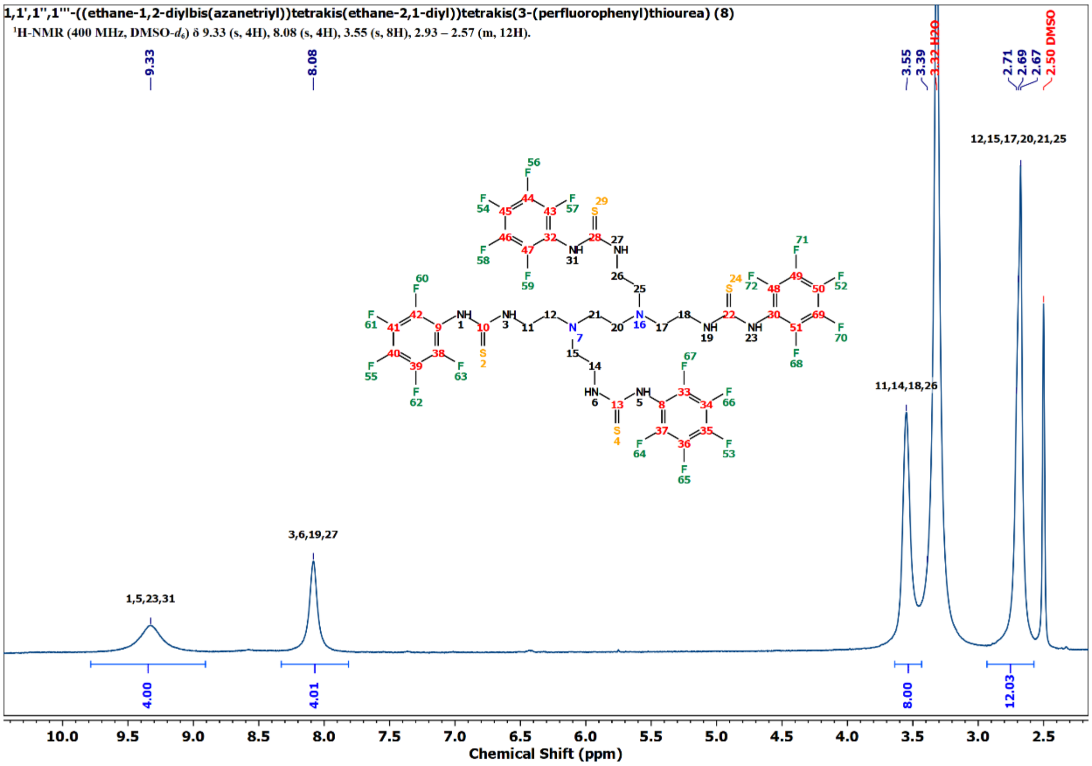

Figure S22. The ^{1}H-NMR (400 MHz) spectrum of transporter **8** in DMSO-d_6 at 298 K, related to the STAR Methods section.

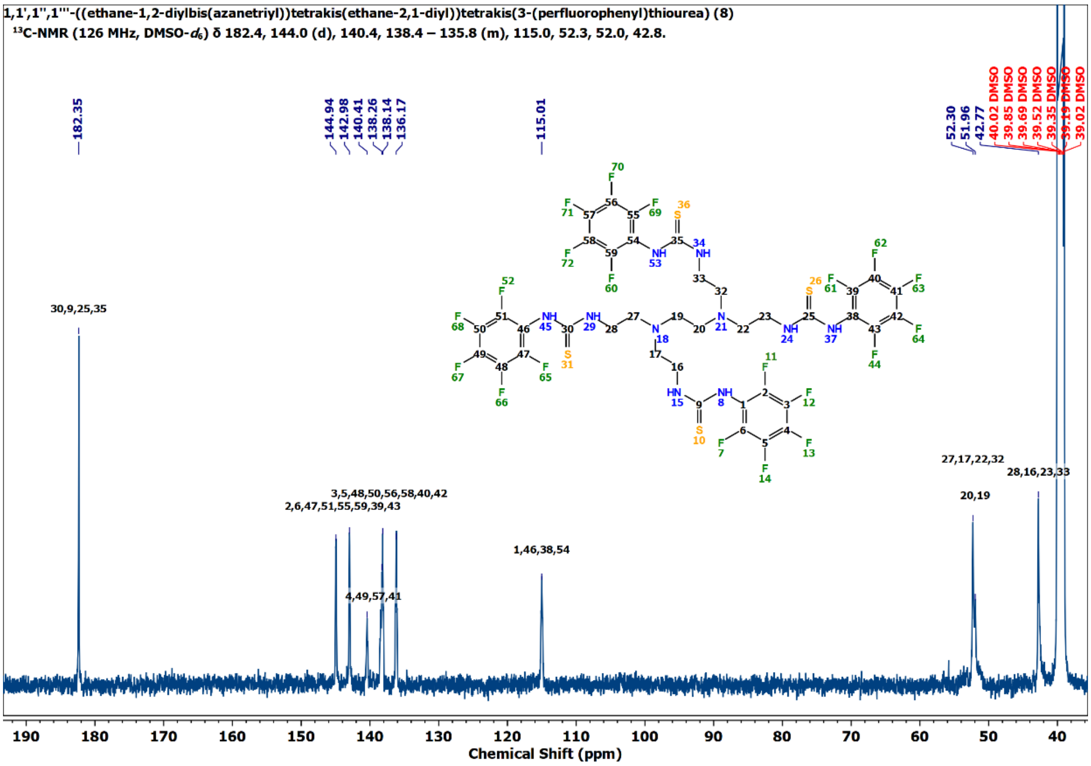

Figure S23. The ^{13}C-NMR (126 MHz) spectrum of transporter **8** in DMSO-d_6 at 298 K, related to the STAR Methods section.

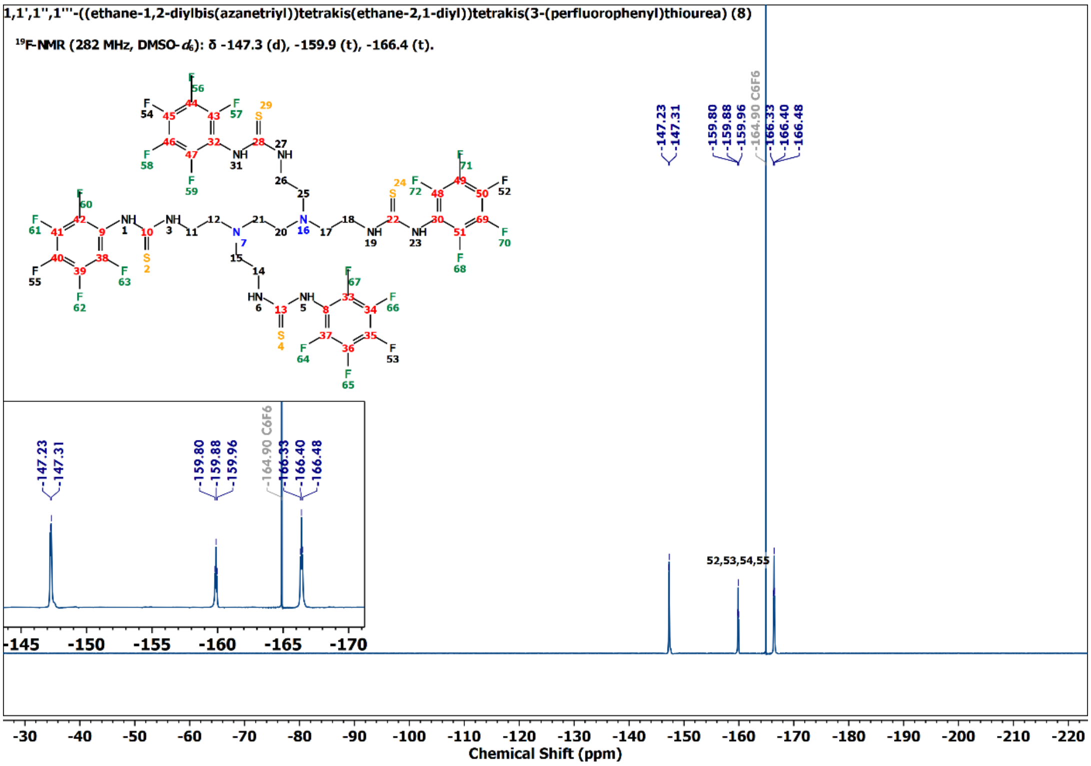

Figure S24. The ^{19}F-NMR (282 MHz) spectrum of transporter **8** in DMSO-d_6 at 298 K, related to the STAR Methods section.

S3. Transport studies:

S3.1 The chloride/nitrate transport exchange assay:

In all cases, the low aqueous solubility prevented any EC_{50} values from being derived due to immediate precipitation and interference with the electrode when added to the assay system. Attempts were also made to test transporters **1**–**8** when they had been pre-incorporated into the lipid bilayer of the POPC vesicles; however, high error, along with very low transport activity, prevented further analysis, related to **Table 4**.

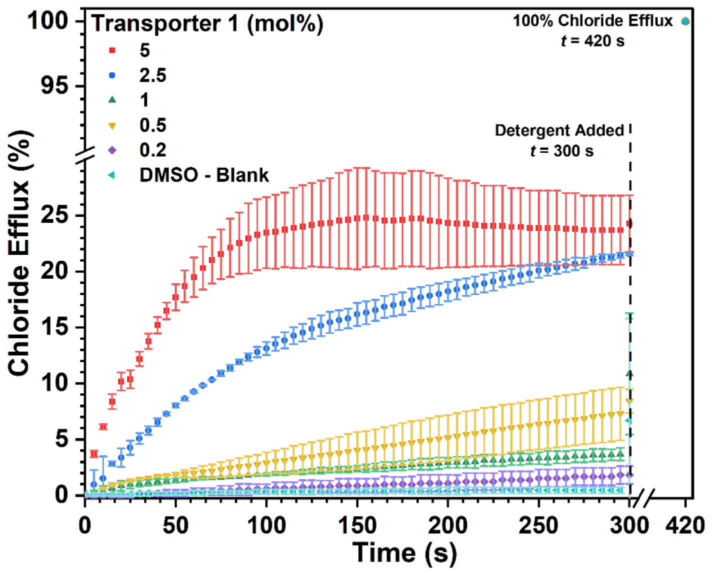

Figure S25. The Cl^{-}/NO_3^{-} exchange elicited by transporter **1** at varying transporter concentrations (mol%) with respect to the lipid concentration, related to **Table 4**. Error bars represent the standard deviation of two repeated experiments.

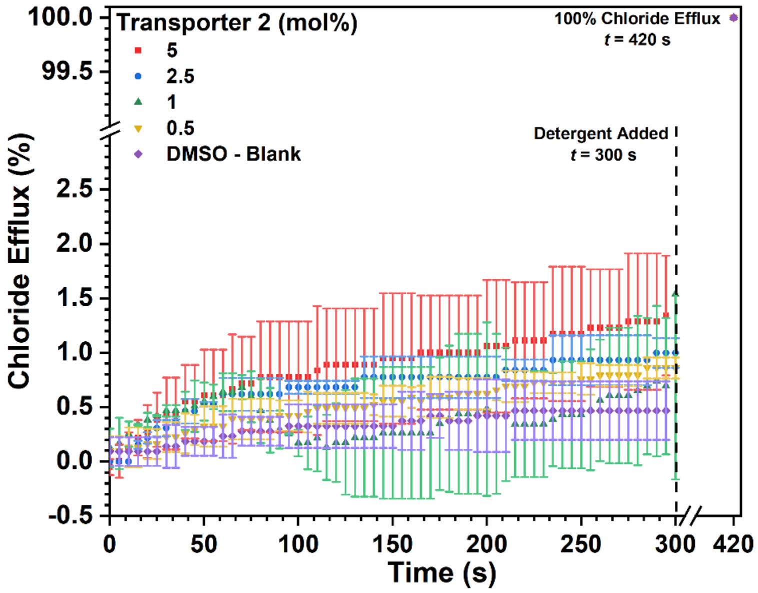

Figure S26. The Cl^{-}/NO_3^{-} exchange elicited by transporter **2** at varying transporter concentrations (mol%) with respect to the lipid concentration, related to **Table 4**. Error bars represent the standard deviation of two repeated experiments.

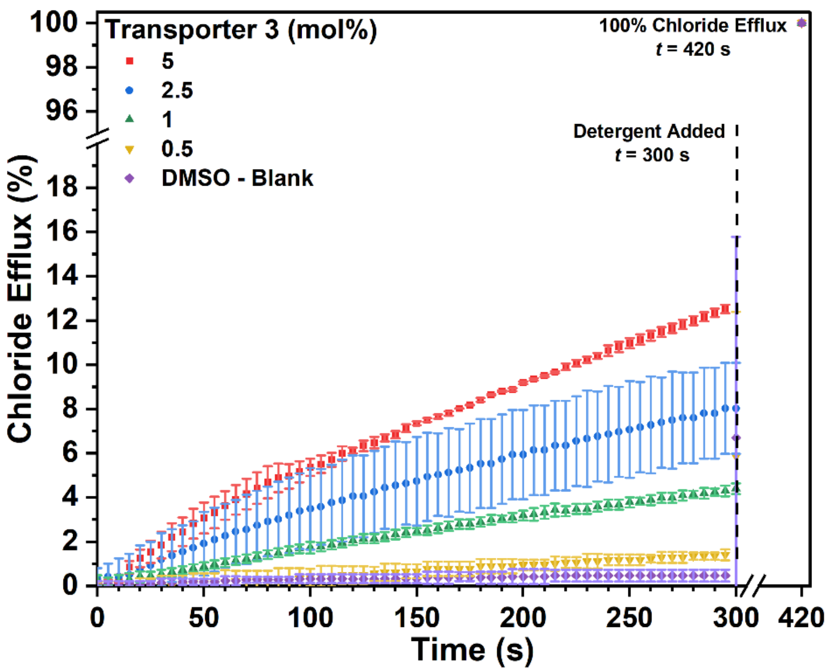

Figure S27. The Cl^-/NO_3^- exchange elicited by transporter **3** at varying transporter concentrations (mol%) with respect to the lipid concentration, related to **Table 4**. Error bars represent the standard deviation of two repeated experiments.

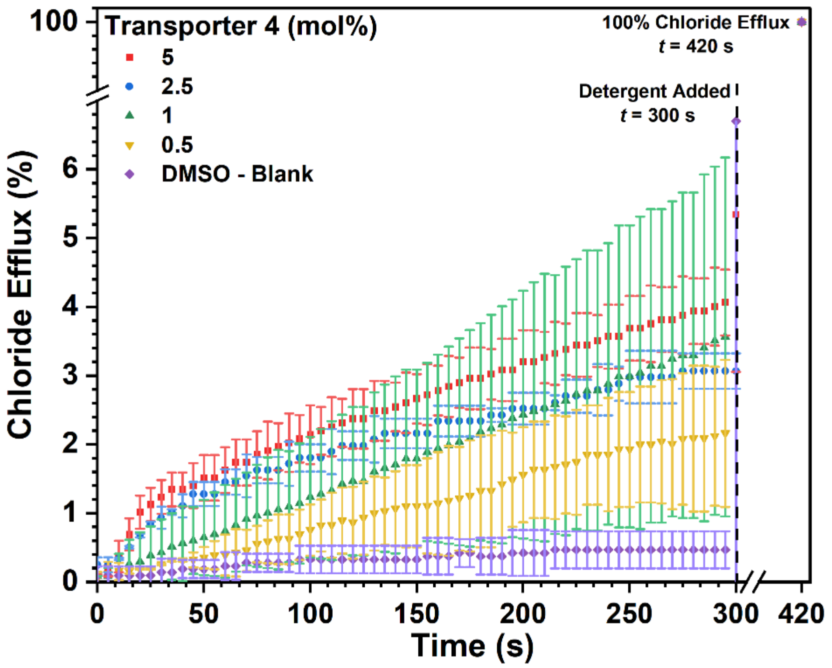

Figure S28. The Cl^-/NO_3^- exchange elicited by transporter **4** at varying transporter concentrations (mol%) with respect to the lipid concentration, related to **Table 4**. Error bars represent the standard deviation of two repeated experiments.

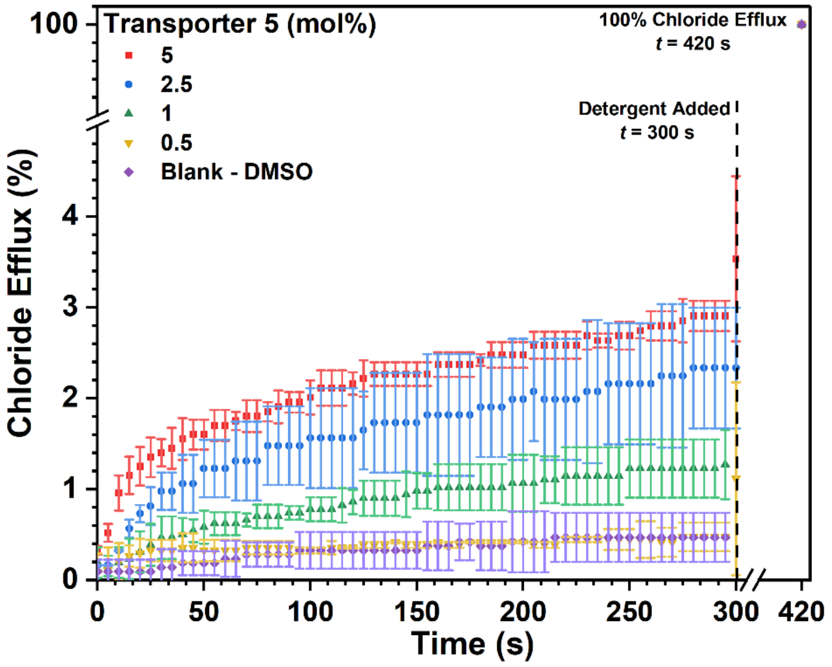

Figure S29. The Cl^-/NO_3^- exchange elicited by transporter **5** at varying transporter concentrations (mol%) with respect to the lipid concentration, related to **Table 4**. Error bars represent the standard deviation of two repeated experiments.

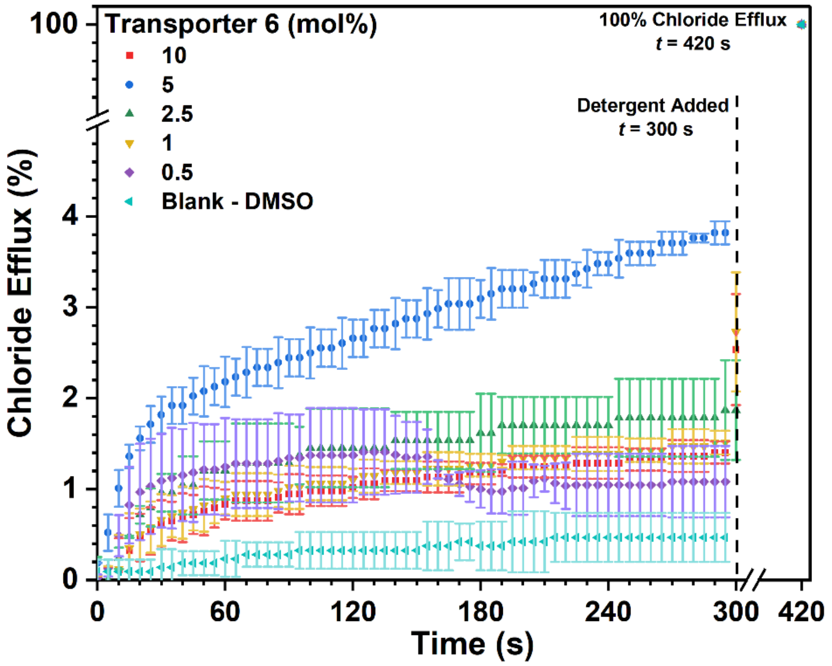

Figure S30. The Cl^-/NO_3^- exchange elicited by transporter **6** at varying transporter concentrations (mol%) with respect to the lipid concentration, related to **Table 4**. Error bars represent the standard deviation of two repeated experiments.

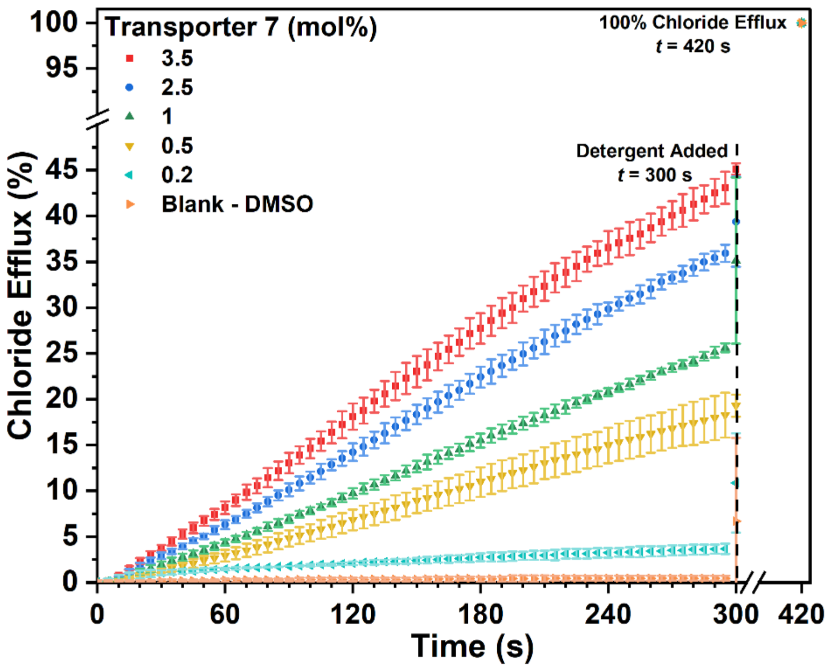

Figure S31. The Cl^-/NO_3^- exchange elicited by transporter **7** at varying transporter concentrations (mol%) with respect to the lipid concentration, related to **Table 4**. Error bars represent the standard deviation of two repeated experiments.

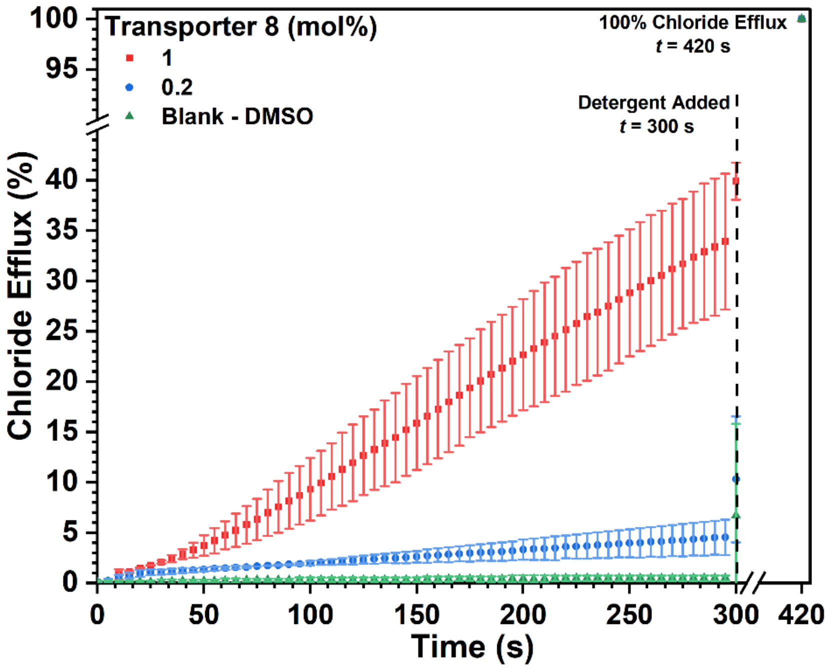

Figure S32. The Cl^-/NO_3^- exchange elicited by transporter **8** at varying transporter concentrations (mol%) with respect to the lipid concentration, related to **Table 4**. Error bars represent the standard deviation of two repeated experiments.

S3.2 The HPTS transport selectivity assay - fatty acid H^+/Cl^- cotransport:

A)

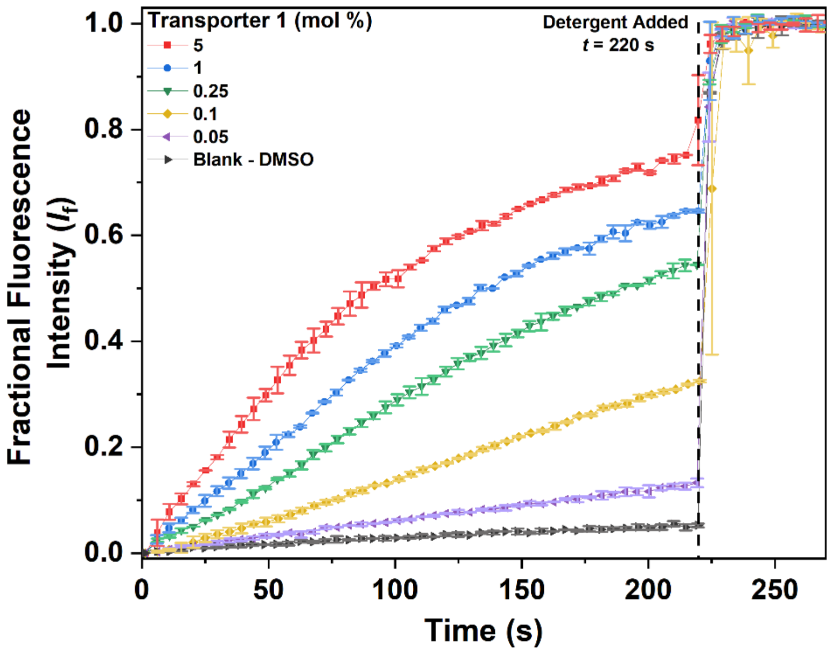

B)

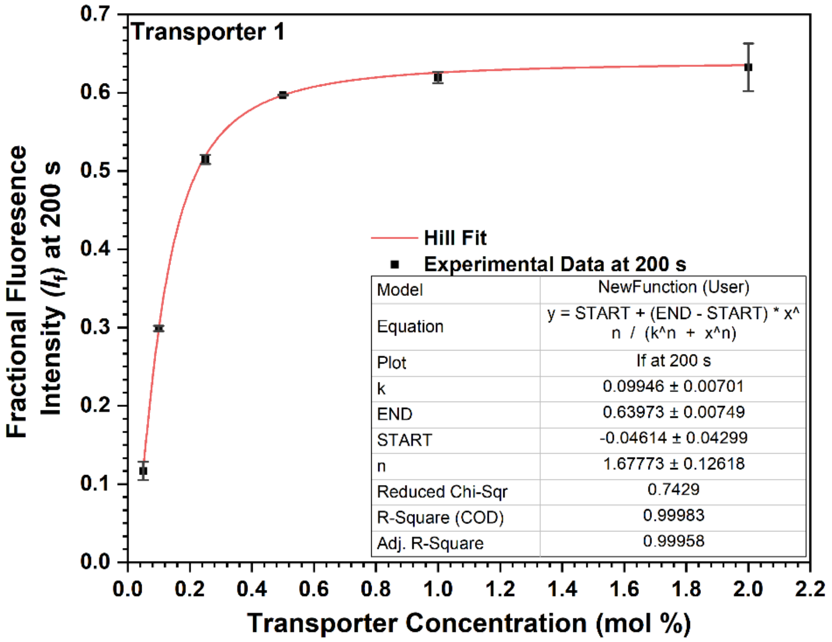

Figure S33. A) The change in fractional fluorescence intensity (I_f) when transporter **1** was added to the assay system at varying concentrations (mol%), related to **Figure 4**, **Table 5**, and the Star Methods section. B) The I_f values were recorded at t = 200 s for each experiment at different transporter concentrations (mol%). All concentrations are expressed in mol% with respect to the lipid concentration, and all error bars represent the standard deviation of two repeated experiments.

A)

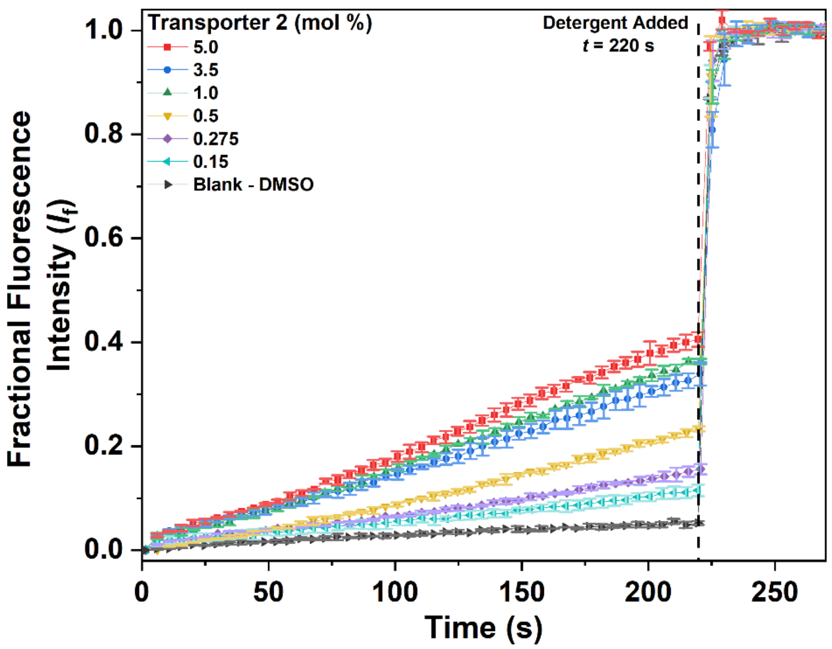

B)

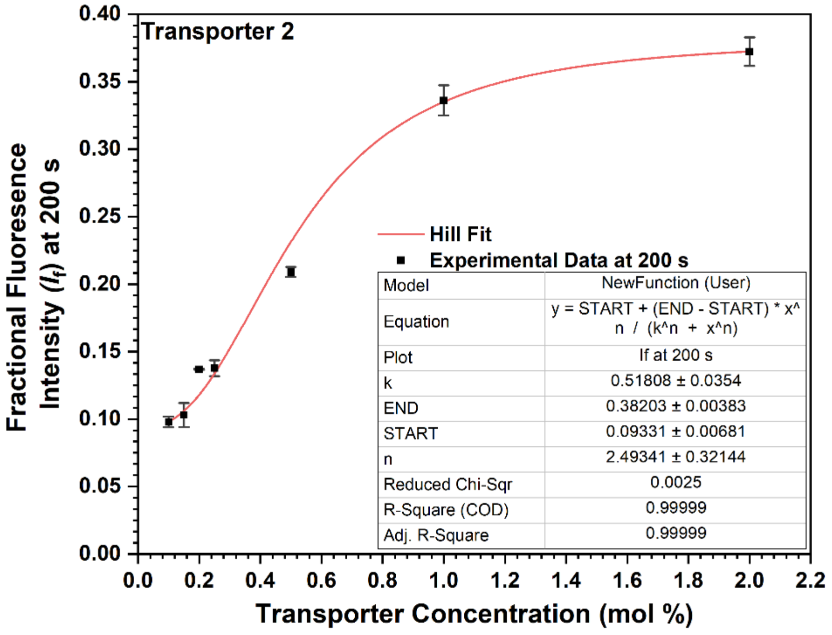

Figure S34. A) The change in fractional fluorescence intensity (I_f) when transporter **2** was added to the assay system at varying concentrations (mol%), related to **Figure 4**, **Table 5**, and the Star Methods section. B) The I_f values were recorded at t = 200 s for each experiment at different transporter concentrations (mol%). All concentrations are expressed in mol% with respect to the lipid concentration, and all error bars represent the standard deviation of two repeated experiments.

A)

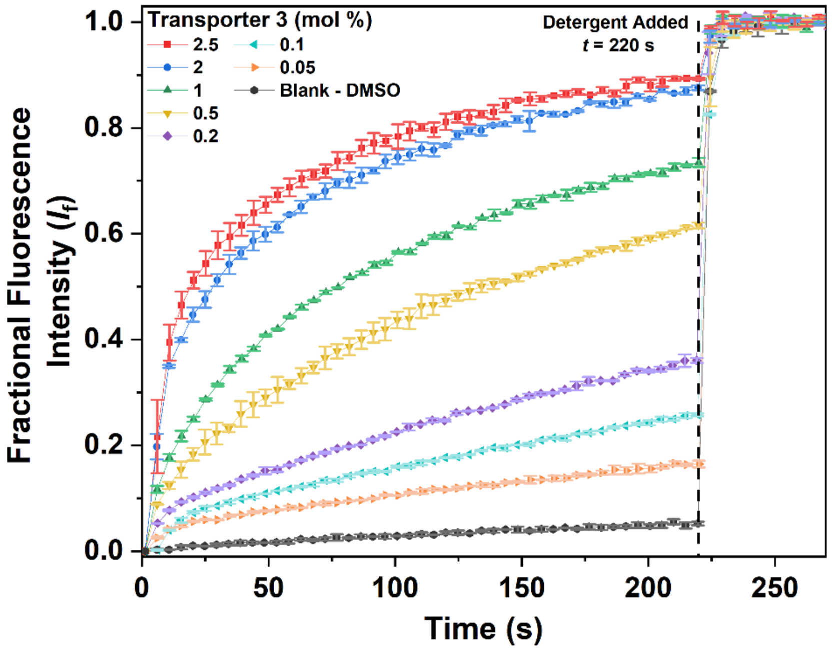

B)

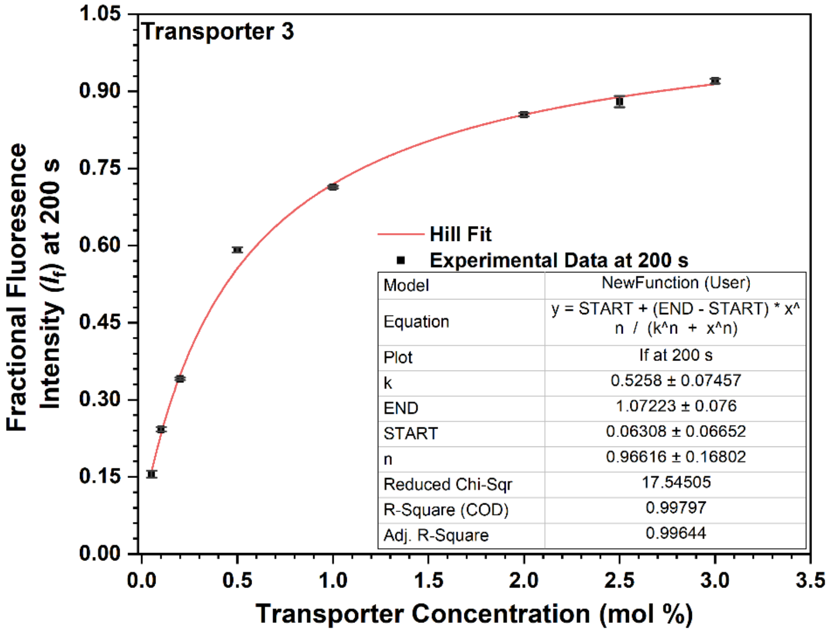

Figure S35. A) The change in fractional fluorescence intensity (I_f) when transporter **3** was added to the assay system at varying concentrations (mol%), related to **Figure 4**, **Table 5**, and the Star Methods section. B) The I_f values were recorded at t = 200 s for each experiment at different transporter concentrations (mol%). All concentrations are expressed in mol% with respect to the lipid concentration, and all error bars represent the standard deviation of two repeated experiments.

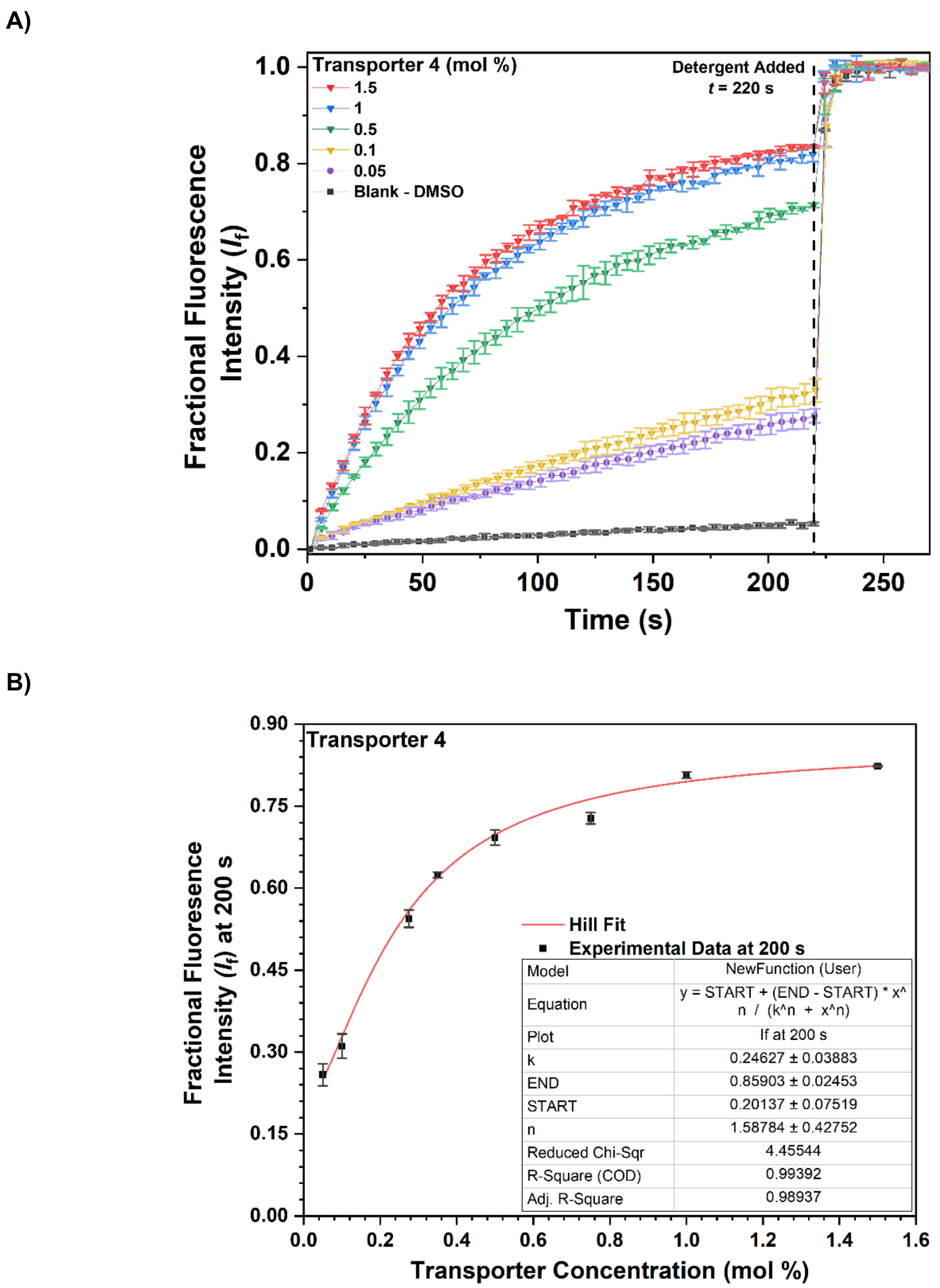

Figure S36. A) The change in fractional fluorescence intensity (I_f) when transporter **4** was added to the assay system at varying concentrations (mol%), related to **Figure 4**, **Table 5**, and the Star Methods section. B) The I_f values were recorded at t = 200 s for each experiment at different transporter concentrations (mol%). All concentrations are expressed in mol% with respect to the lipid concentration, and all error bars represent the standard deviation of two repeated experiments.

A)

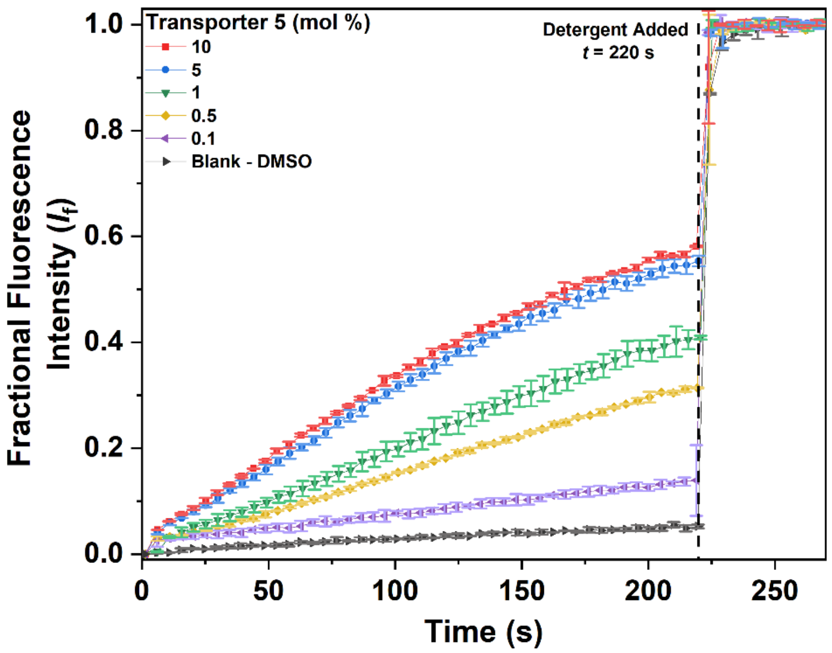

B)

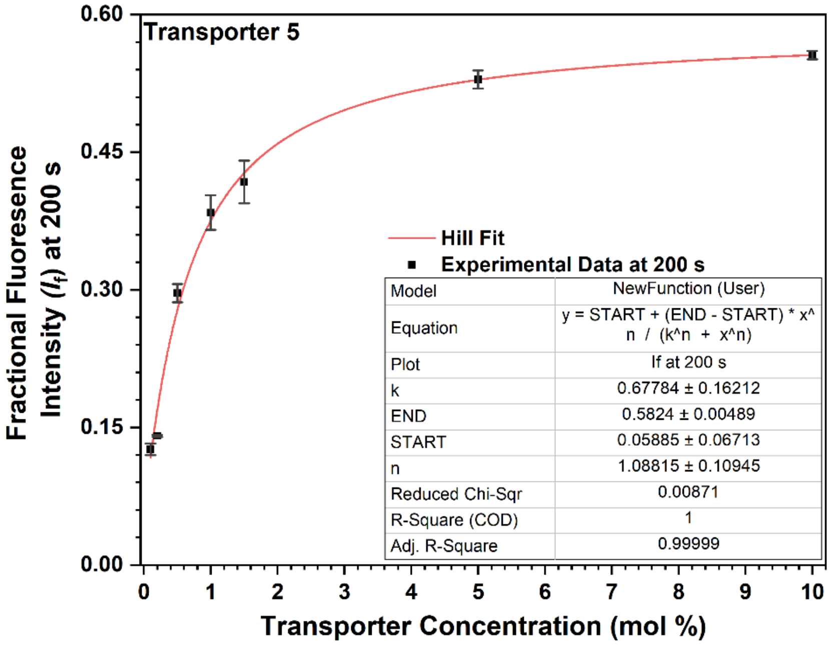

Figure S37. A) The change in fractional fluorescence intensity (I_f) when transporter **5** was added to the assay system at varying concentrations (mol%), related to **Figure 4**, **Table 5**, and the Star Methods section. B) The I_f values were recorded at t = 200 s for each experiment at different transporter concentrations (mol%). All concentrations are expressed in mol% with respect to the lipid concentration, and all error bars represent the standard deviation of two repeated experiments.

A)

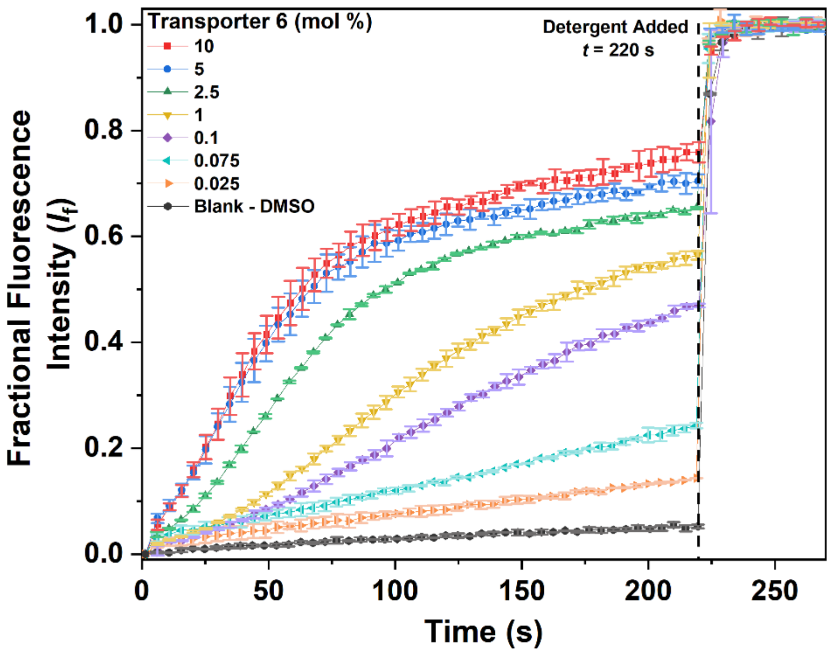

B)

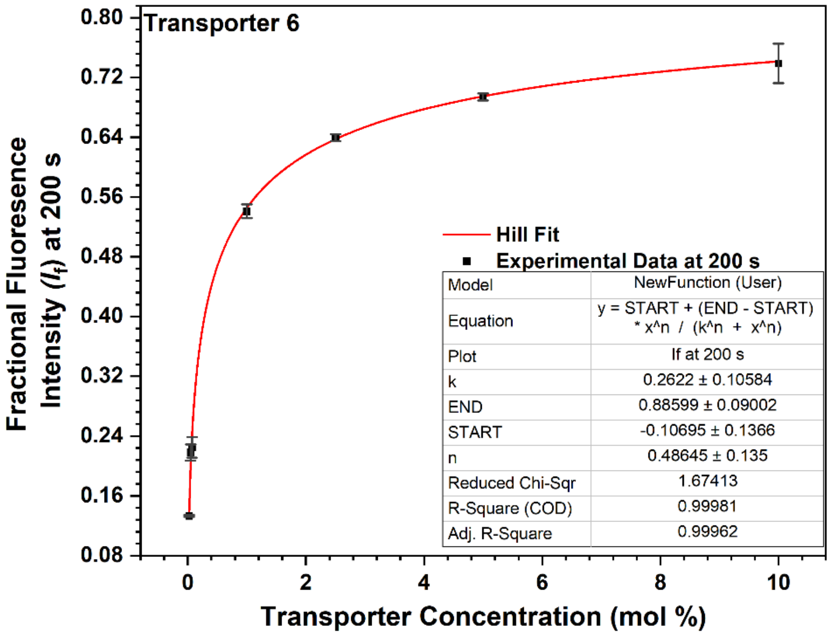

Figure S38. A) The change in fractional fluorescence intensity (I_f) when transporter **6** was added to the assay system at varying concentrations (mol%), related to **Figure 4**, **Table 5**, and the Star Methods section. B) The I_f values were recorded at t = 200 s for each experiment at different transporter concentrations (mol%). All concentrations are expressed in mol% with respect to the lipid concentration, and all error bars represent the standard deviation of two repeated experiments.

A)

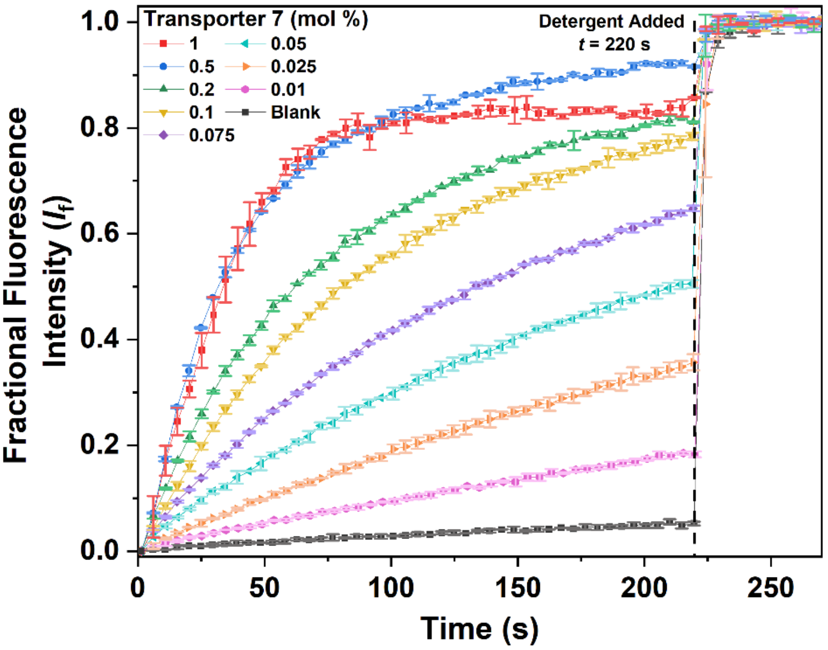

B)

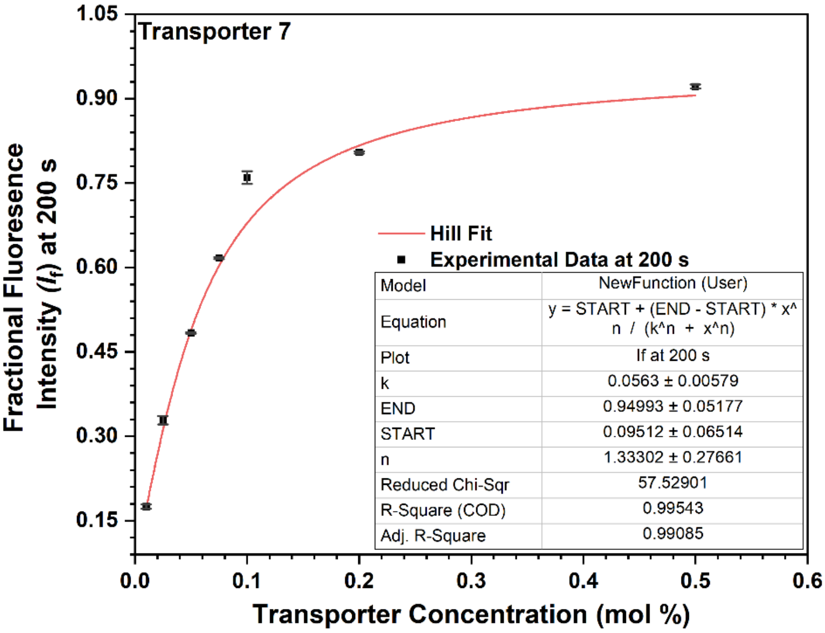

Figure S39. A) The change in fractional fluorescence intensity (I_f) when transporter **7** was added to the assay system at varying concentrations (mol%), related to **Figure 4**, **Table 5**, and the Star Methods section. B) The I_f values were recorded at t = 200 s for each experiment at different transporter concentrations (mol%). All concentrations are expressed in mol% with respect to the lipid concentration, and all error bars represent the standard deviation of two repeated experiments.

A)

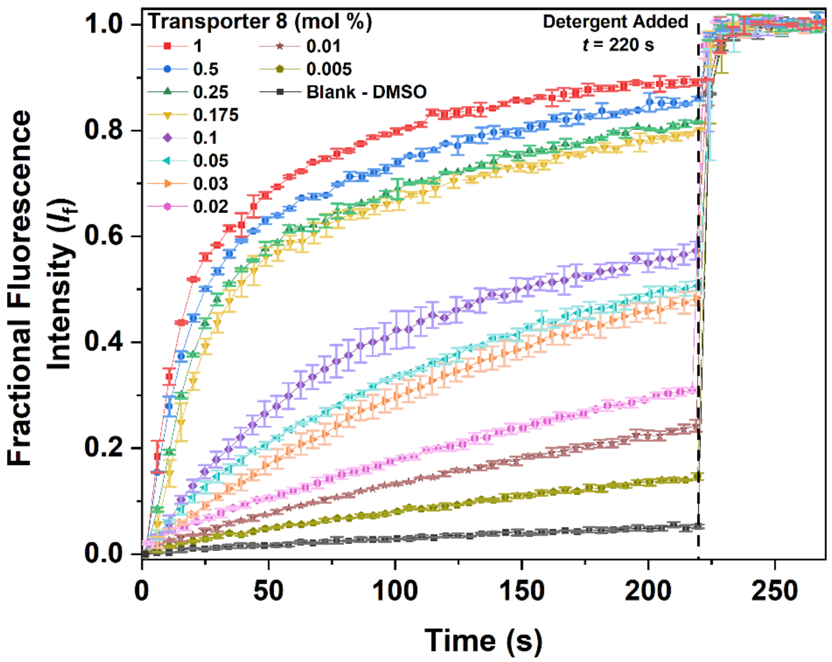

B)

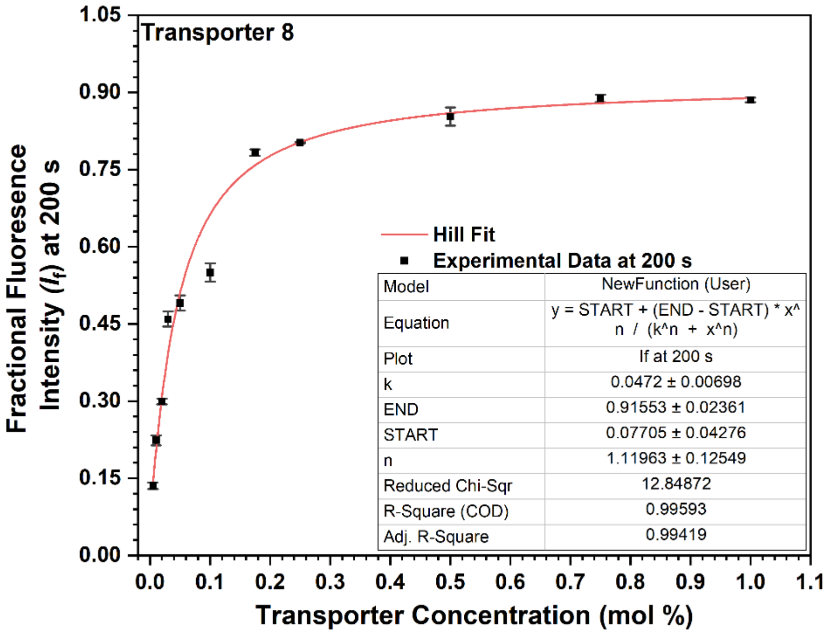

Model	NewFunction (User)
Equation	y = START + (END - START) * x^n / (k^n + x^n)
Plot	If at 200 s
k	0.0472 ± 0.00698
END	0.91553 ± 0.02361
START	0.07705 ± 0.04276
n	1.11963 ± 0.12549
Reduced Chi-Sqr	12.84872
R-Square (COD)	0.99593
Adj. R-Square	0.99419

Figure S40. A) The change in fractional fluorescence intensity (I_f) when transporter **8** was added to the assay system at varying concentrations (mol%), related to **Figure 4**, **Table 5**, and the Star Methods section. B) The I_f values were recorded at t = 200 s for each experiment at different transporter concentrations (mol%). All concentrations are expressed in mol% with respect to the lipid concentration, and all error bars represent the standard deviation of two repeated experiments.

A)

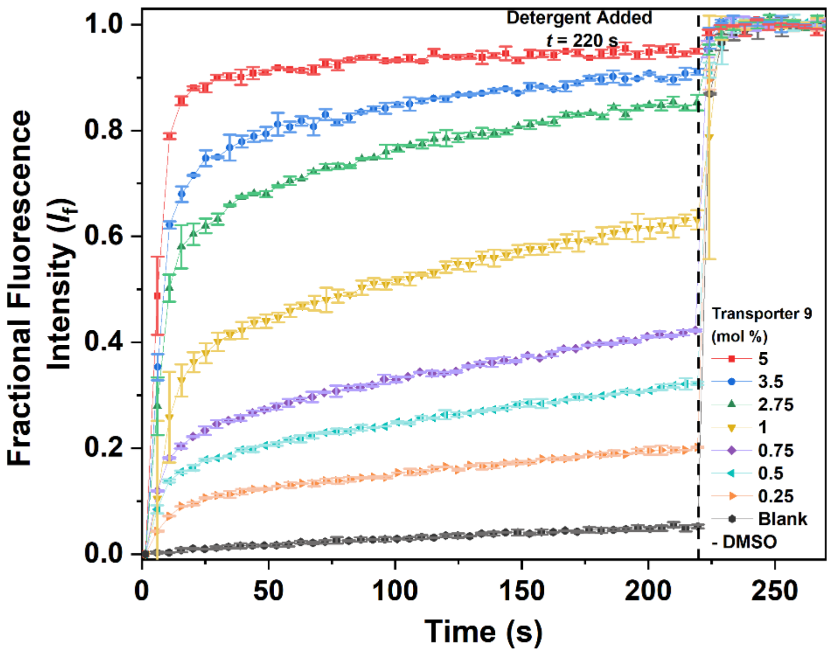

B)

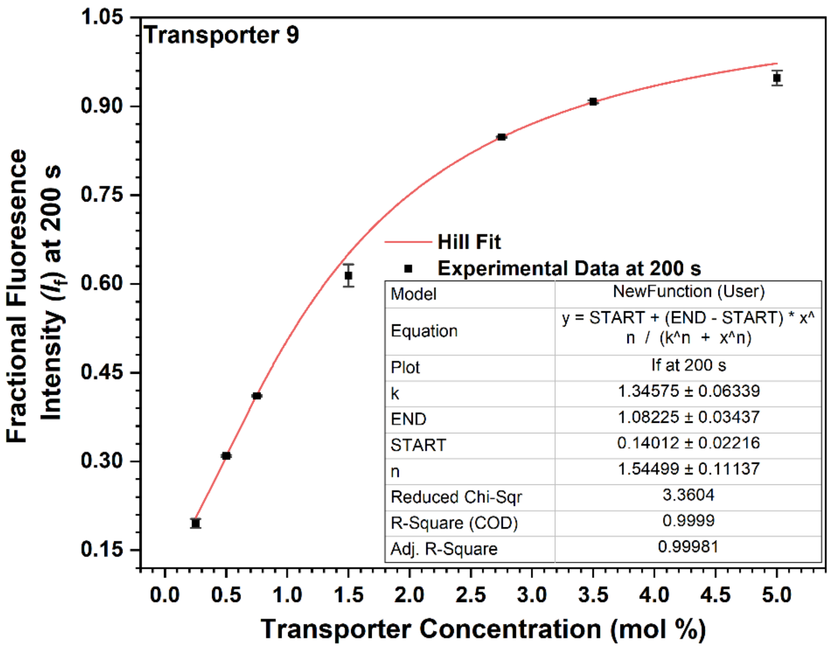

Figure S41. A) The change in fractional fluorescence intensity (I_f) when control transporter **9** was added to the assay system at varying concentrations (mol%), related to **Figure 4**, **Table 5**, and the Star Methods section. B) The I_f values were recorded at t = 200 s for each experiment at different transporter concentrations (mol%). All concentrations are expressed in mol% with respect to the lipid concentration, and all error bars represent the standard deviation of two repeated experiments.

A)

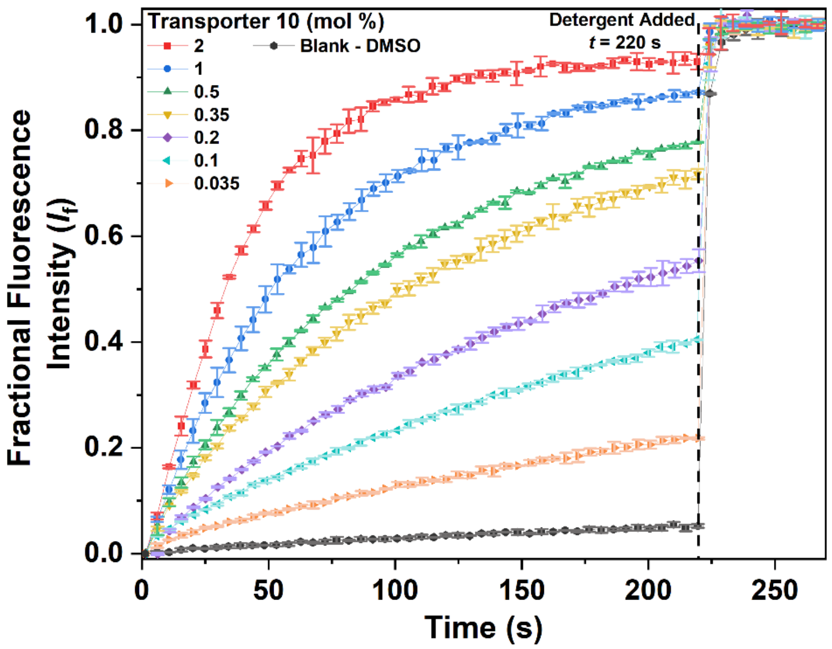

B)

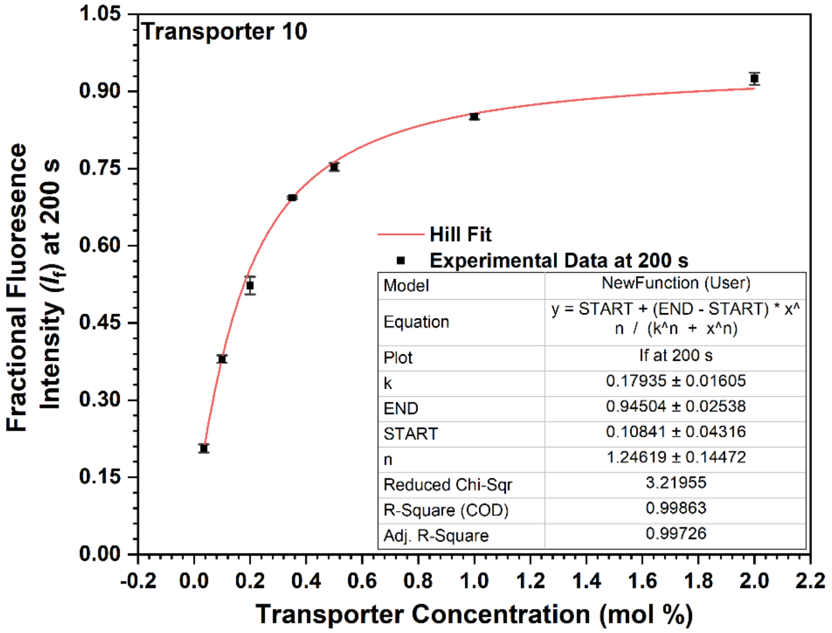

Figure S42. A) The change in fractional fluorescence intensity (I_f) when control transporter **10** was added to the assay system at varying concentrations (mol%), related to **Figure 4**, **Table 5**, and the Star Methods section. B) The I_f values were recorded at t = 200 s for each experiment at different transporter concentrations (mol%). All concentrations are expressed in mol% with respect to the lipid concentration, and all error bars represent the standard deviation of two repeated experiments.

S3.3 The modified HPTS transport selectivity assay: selectivity among anions

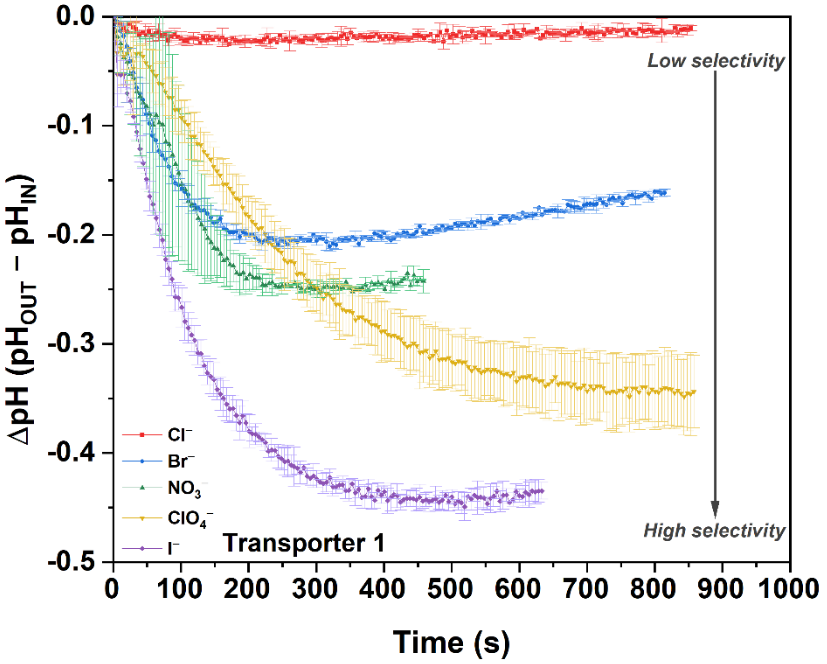

Figure S43. The normalized anion transport selectivity of transporter **1** (1 mol%) with error bars showing the standard deviation of two repeats and where the direction of anion selectivity is represented by a grey arrow, related to **Figure 5** and the Star Methods section. Transporters concentration is shown as mol% values with respect to the lipid concentration.

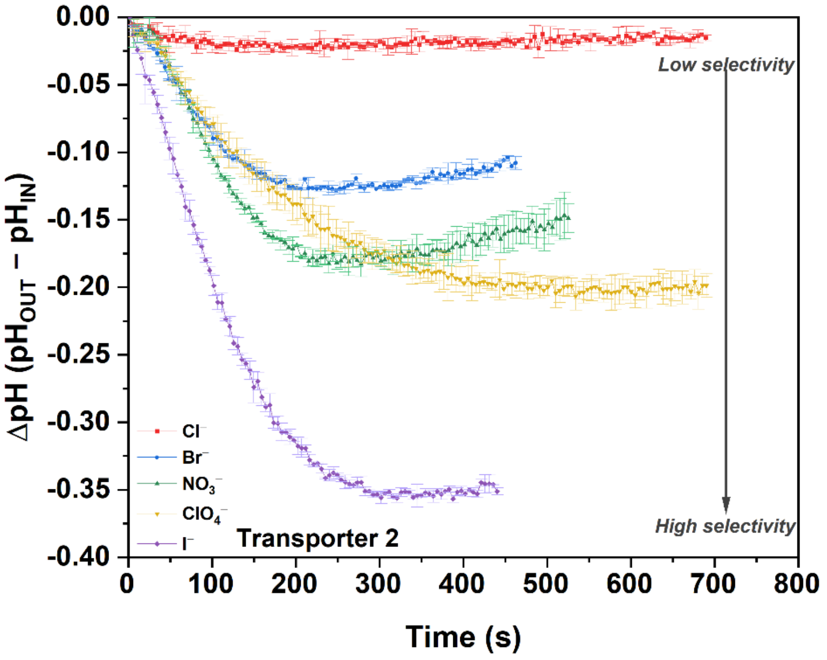

Figure S44. The normalized anion transport selectivity of transporter **2** (1 mol%) with error bars showing the standard deviation of two repeats and where the direction of anion selectivity is represented by a grey arrow, related to **Figure 5** and the Star Methods section. Transporters concentration is shown as mol% values with respect to the lipid concentration.

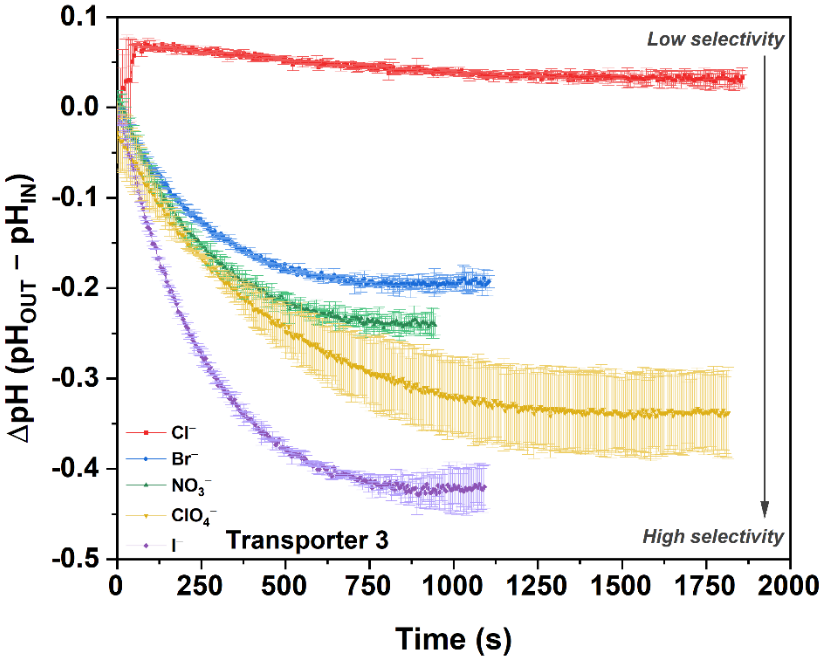

Figure S45. The normalized anion transport selectivity of transporter **3** (1 mol%) with error bars showing the standard deviation of two repeats and where the direction of anion selectivity is represented by a grey arrow, related to **Figure 5** and the Star Methods section. Transporters concentration is shown as mol% values with respect to the lipid concentration.

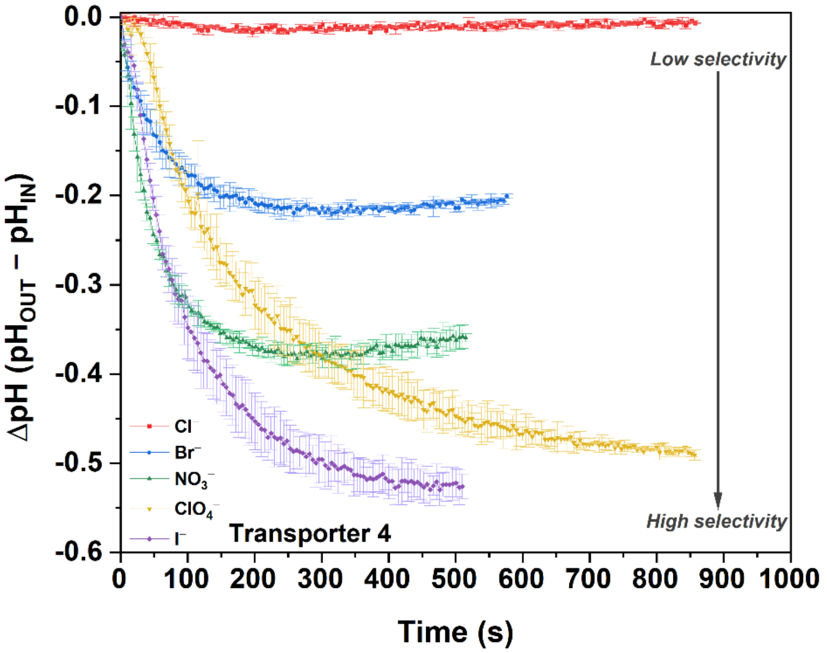

Figure S46. The normalized anion transport selectivity of transporter **4** (1 mol%) with error bars showing the standard deviation of two repeats and where the direction of anion selectivity is represented by a grey arrow, related to **Figure 5** and the Star Methods section. Transporters concentration is shown as mol% values with respect to the lipid concentration.

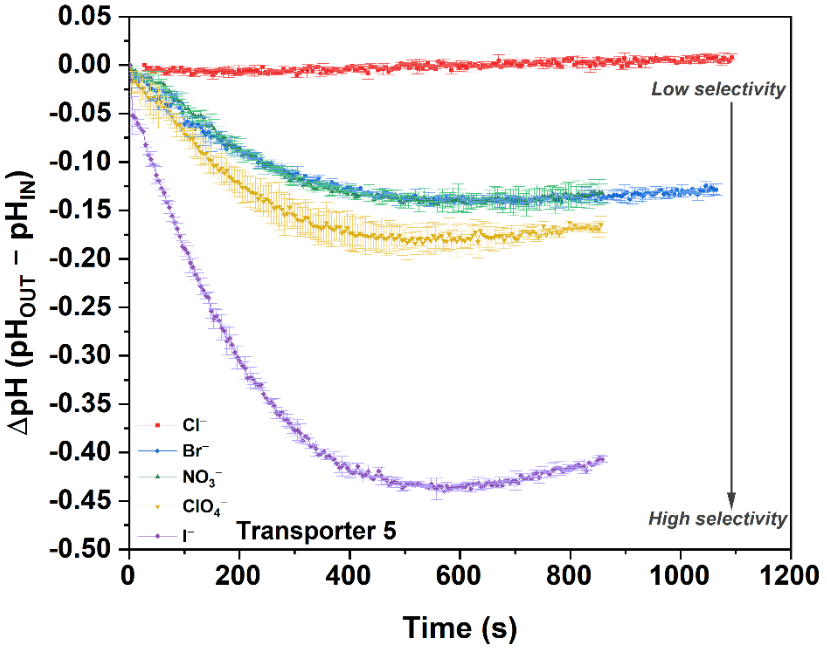

Figure S47. The normalized anion transport selectivity of transporter **5** (1 mol%) with error bars showing the standard deviation of two repeats and where the direction of anion selectivity is represented by a grey arrow, related to **Figure 5** and the Star Methods section. Transporters concentration is shown as mol% values with respect to the lipid concentration.

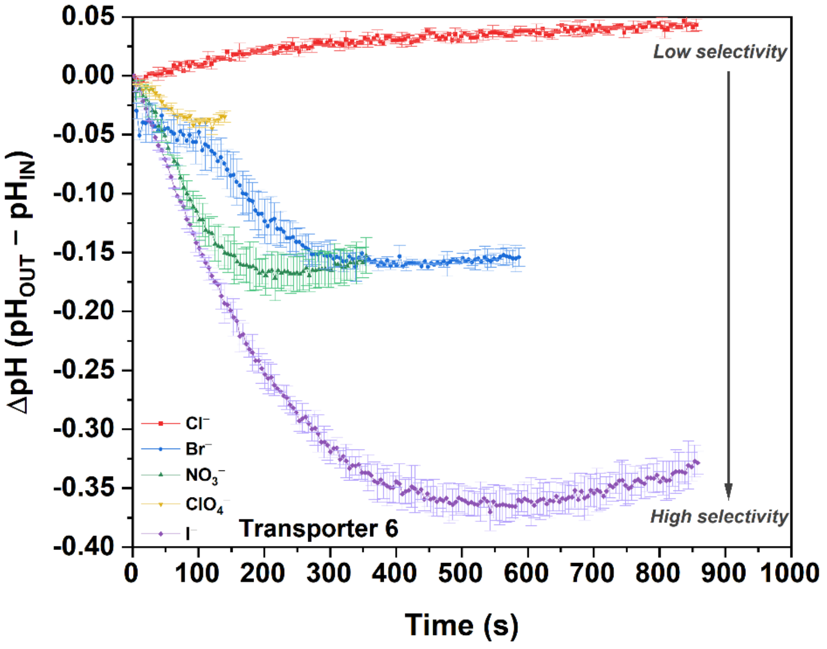

Figure S48. The normalized anion transport selectivity of transporter **6** (0.5 mol%) with error bars showing the standard deviation of two repeats and where the direction of anion selectivity is represented by a grey arrow, related to **Figure 5** and the Star Methods section. Transporters concentration is shown as mol% values with respect to the lipid concentration.

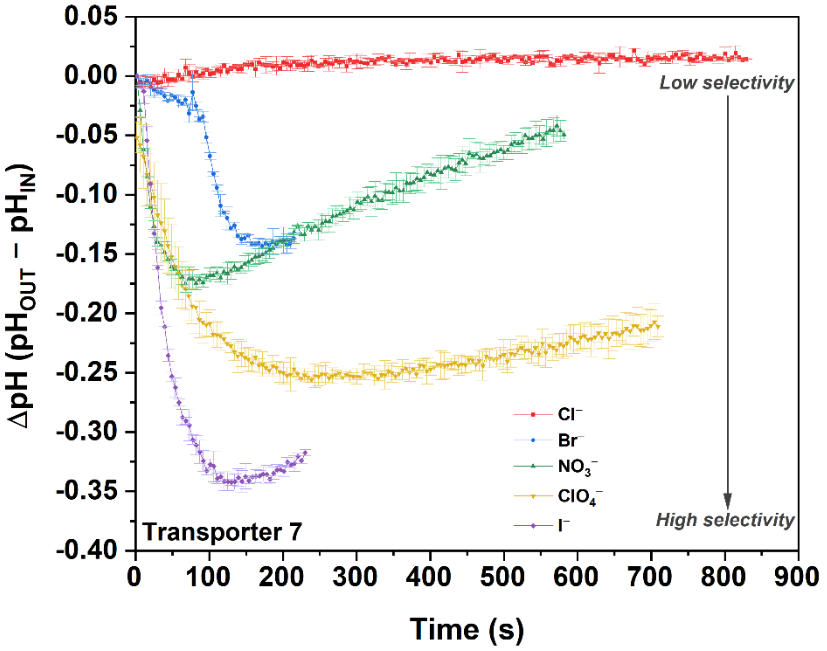

Figure S49. The normalized anion transport selectivity of transporter **7** (1 mol%) with error bars showing the standard deviation of two repeats and where the direction of anion selectivity is represented by a grey arrow, related to **Figure 5** and the Star Methods section. Transporters concentration is shown as mol% values with respect to the lipid concentration.

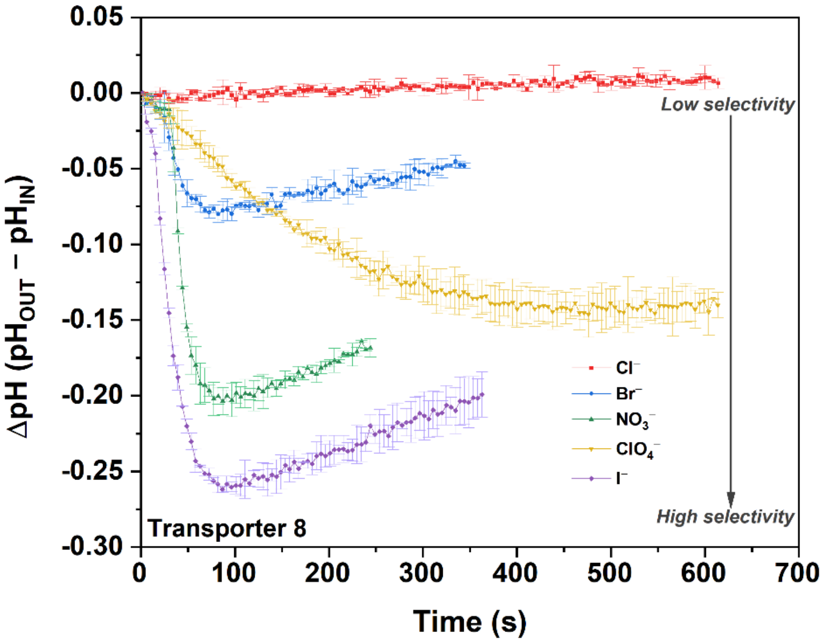

Figure S50. The normalized anion transport selectivity of transporter **8** (0.5 mol%) with error bars showing the standard deviation of two repeats and where the direction of anion selectivity is represented by a grey arrow, related to **Figure 5** and the Star Methods section. Transporters concentration is shown as mol% values with respect to the lipid concentration.

S4. Structure-activity relationships:

S4.1 Initial rate constants from the chloride/nitrate exchange assay:

Due to the relative insolubility of transporters **2**, **4**, **5**, and **6**, no initial rate constant (k_{ini}) values could be calculated accurately using **Equations 7** and **8** due to inherently high levels of recorded error represented as error grey error bars in **Figures S52**, **S54–S56**, related to **Table 4** and the Star Methods section.

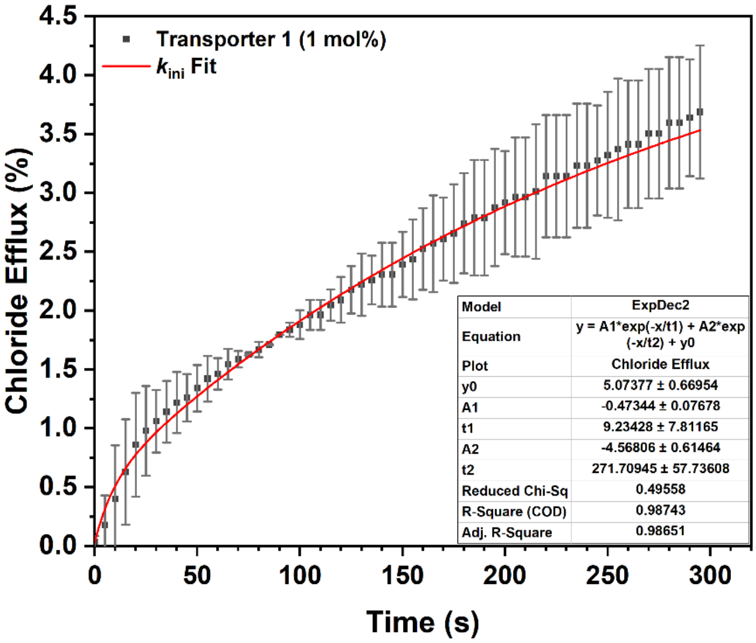

Figure S51. The Cl⁻ efflux (%) data of transporter **1** (1 mol%, black) *vs* time (s), which has undergone non-linear curve fitting (red) using **Equation 7**, related to **Table 4** and the Star Methods section. Error bars (grey) depict the standard deviation of two repeated experiments.

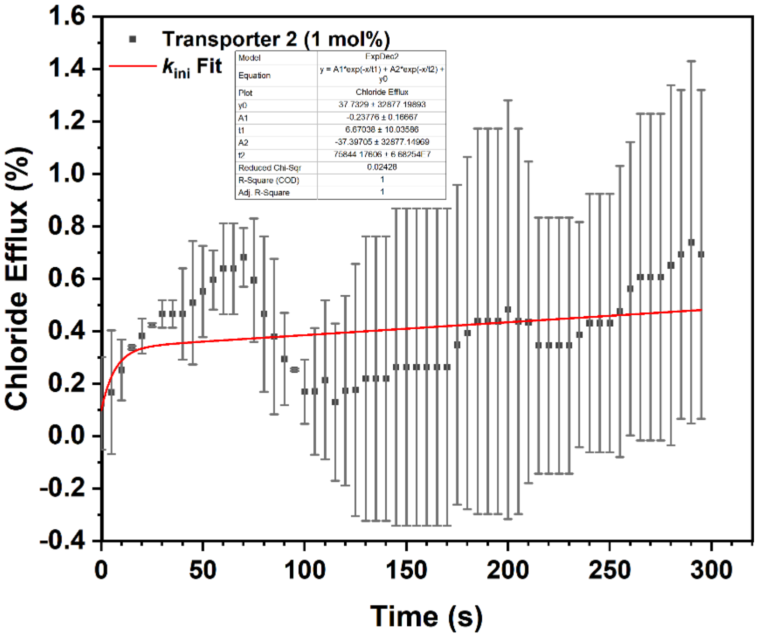

Figure S52. The Cl⁻ efflux (%) data of transporter **2** (1 mol%, black) *vs* time (s), which has undergone non-linear curve fitting (red) using **Equation 7**, related to **Table 4** and the Star Methods section. Error bars (grey) depict the standard deviation of two repeated experiments.

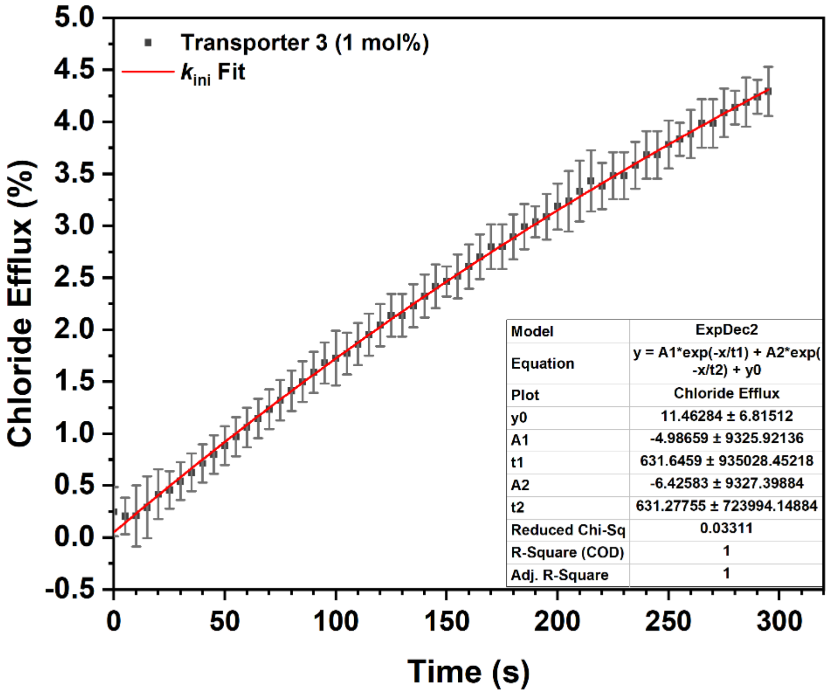

Figure S53. The Cl⁻ efflux (%) data of transporter **3** (1 mol%, black) *vs* time (s), which has undergone non-linear curve fitting (red) using **Equation 7**, related to **Table 4** and the Star Methods section. Error bars (grey) depict the standard deviation of two repeated experiments.

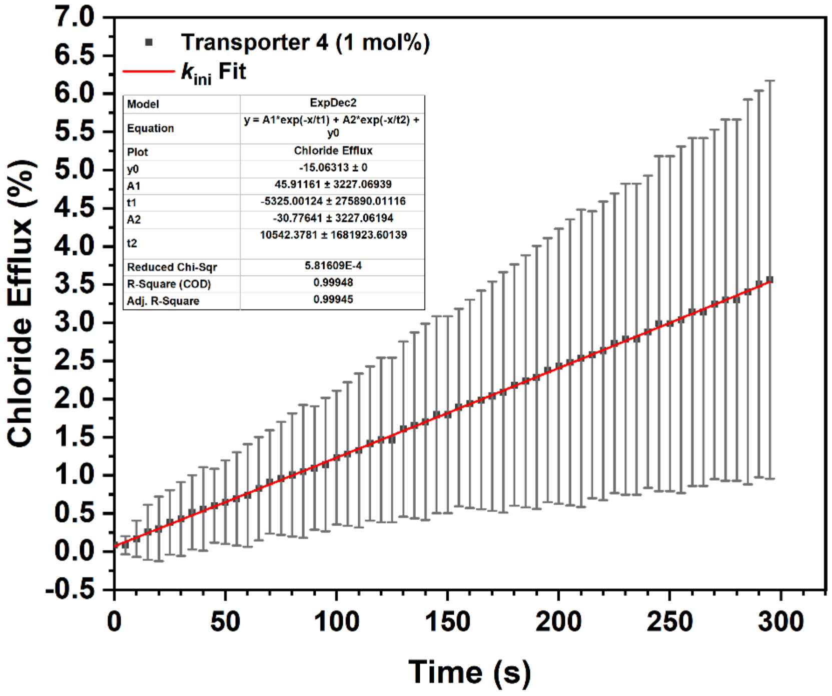

Figure S54. The Cl⁻ efflux (%) data of transporter **4** (1 mol%, black) *vs* time (s), which has undergone non-linear curve fitting (red) using **Equation 7**, related to **Table 4** and the Star Methods section. Error bars (grey) depict the standard deviation of two repeated experiments.

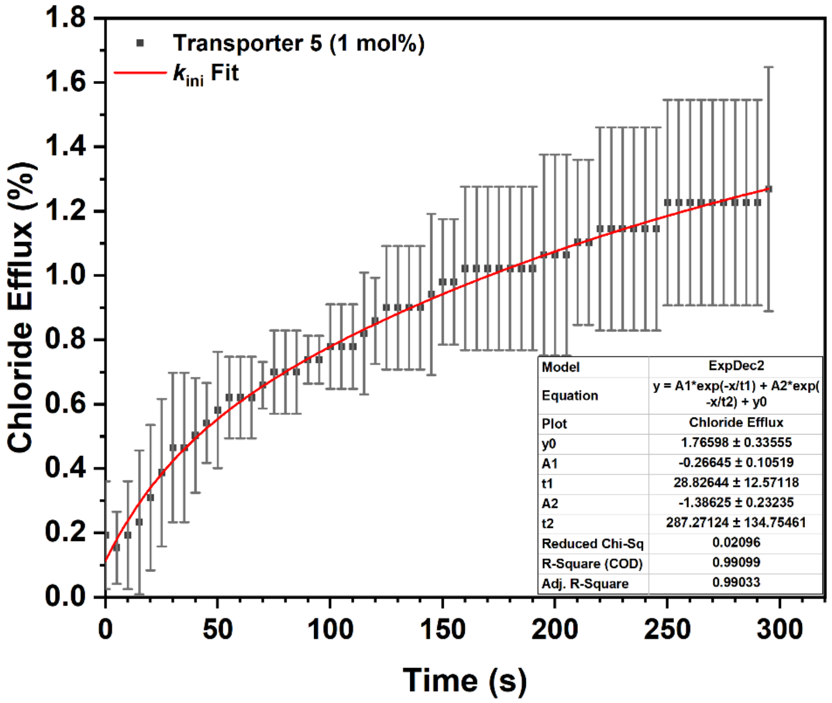

Figure S55. The Cl⁻ efflux (%) data of transporter **5** (1 mol%, black) *vs* time (s), which has undergone non-linear curve fitting (red) using **Equation 7**, related to **Table 4** and the Star Methods section. Error bars (grey) depict the standard deviation of two repeated experiments.

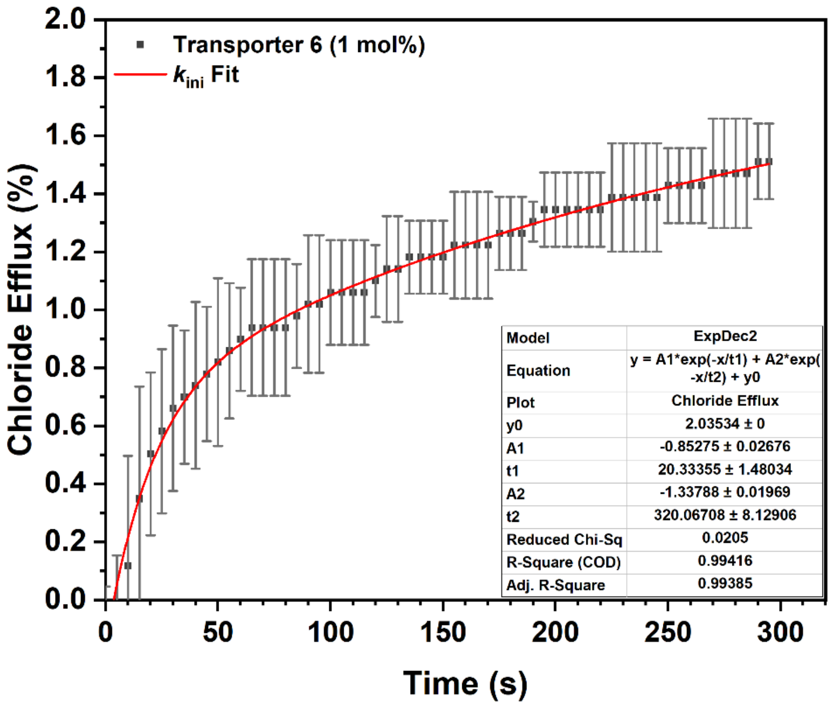

Figure S56. The Cl⁻ efflux (%) data of transporter **6** (1 mol%, black) *vs* time (s), which has undergone non-linear curve fitting (red) using **Equation 7**, related to **Table 4** and the Star Methods section. Error bars (grey) depict the standard deviation of two repeated experiments.

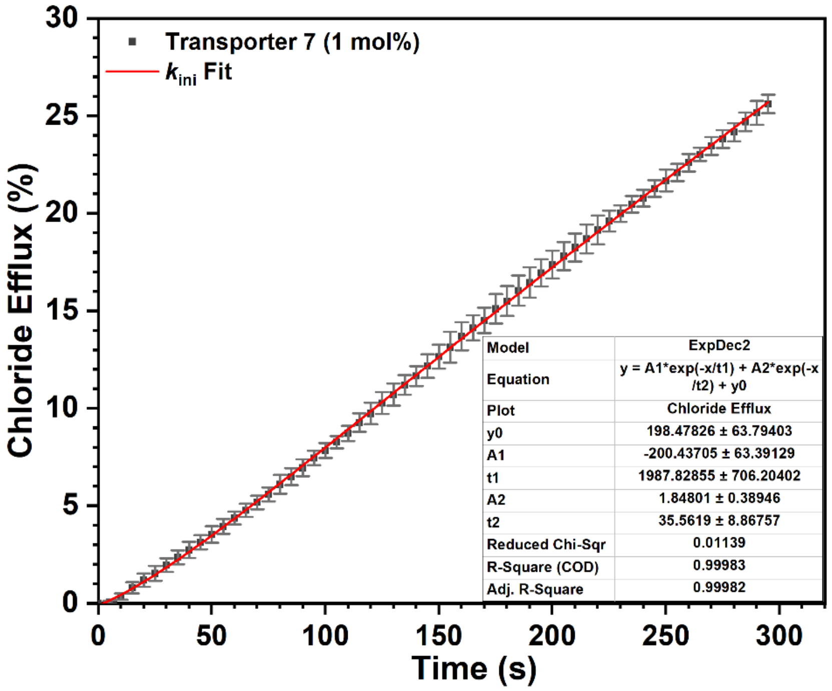

Figure S57. The Cl^- efflux (%) data of transporter **7** (1 mol%, black) *vs* time (s), which has undergone non-linear curve fitting (red) using **Equation 7**, related to **Table 4** and the Star Methods section. Error bars (grey) depict the standard deviation of two repeated experiments.

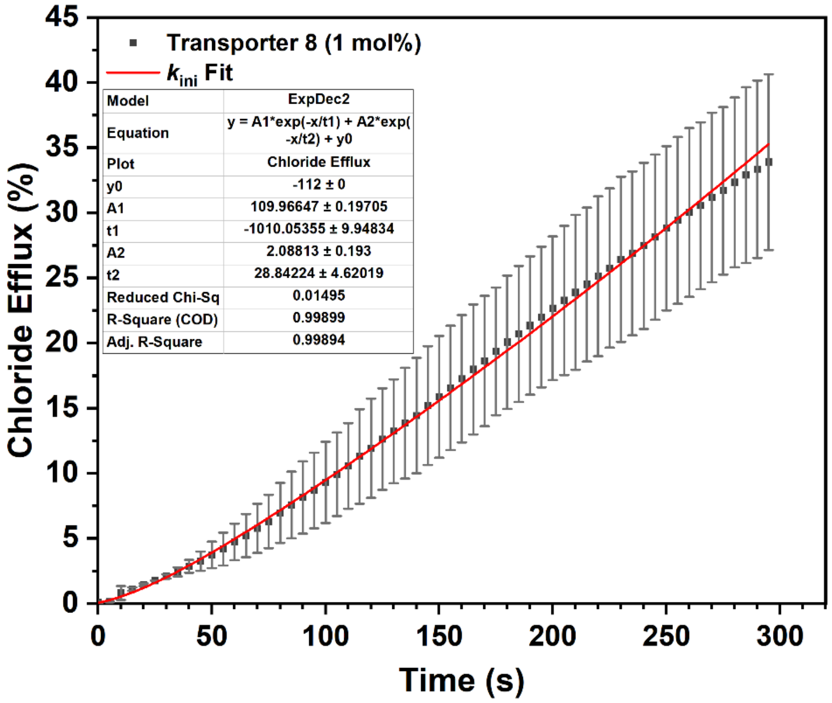

Figure S58. The Cl^- efflux (%) data of transporter **8** (1 mol%, black) *vs* time (s), which has undergone non-linear curve fitting (red) using **Equation 7**, related to **Table 4** and the Star Methods section. Error bars (grey) depict the standard deviation of two repeated experiments.

S4.2 Initial rate constants from the HPTS transport selectivity assay:

All transporter concentrations are represented as a ratio of the transporter concentration with respect to the lipid concentration. Error bars depict the standard deviation of two experiments, and all fitting was done using the two-phase exponential decay function (**Equation 8**) related to **Table 5** and the Star Methods section.

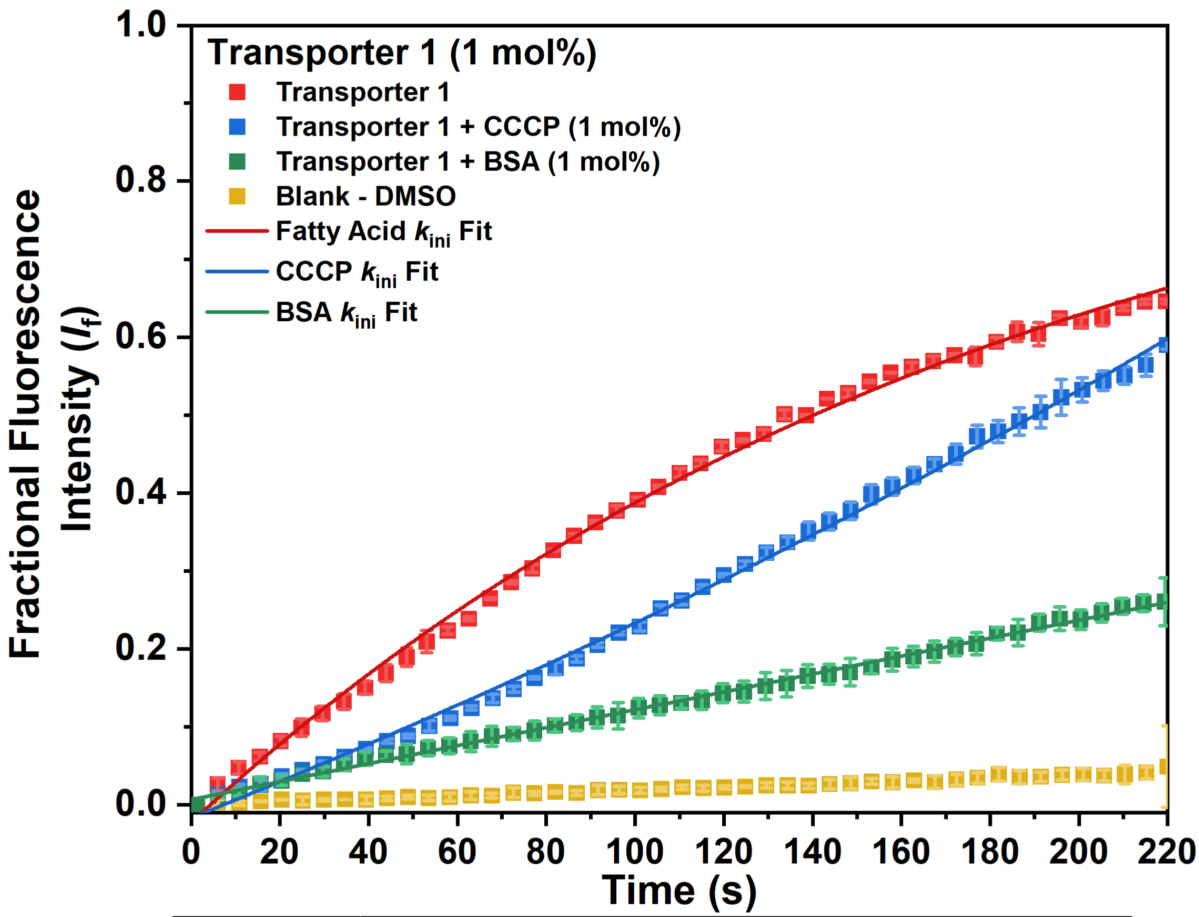

Model	ExpDec2		
Equation	y = A1*exp(-x/t1) + A2*exp(-x/t2) + y0		
Plot	Fractional Fluorescence Intensity	Fractional Fluorescence Intensity	Fractional Fluorescence Intensity
y0	0.97062 ± 0.31312	-1.30871 ± 6.11813	65.54453 ± 242.54922
A1	-0.4895 ± 147699.2454	0.64363 ± 74000.91111	-26.20339 ± 295.00598
t1	187.65821 ± 1229999.8347	-565.07958 ± 2.82032E7	50383.55735 ± 508715.43146
A2	-0.50392 ± 147699.22964	0.64831 ± 74003.16441	-39.33453 ± 437.86137
t2	187.64221 ± 1194803.099	-565.03534 ± 2.79883E7	62069.79826 ± 591753.03
Reduced Chi-Sqr	1.21523E-4	8.68447E-5	1.81233E-5
R-Square (COD)	1	1	1
Adj. R-Square	1	1	1

Figure S59. The fractional fluorescence intensity (I_f) facilitated by transporter **1** (1 mol%) in vesicles with membrane-embedded fatty acids (red), vesicles which have been treated with the weak acid protonophore CCCP (1 mol%, blue), vesicles which have been pre-treated with BSA (1 mol%, green) to remove all fatty acids from vesicle membranes, and the control DMSO experiment (yellow) related to **Table 5**.

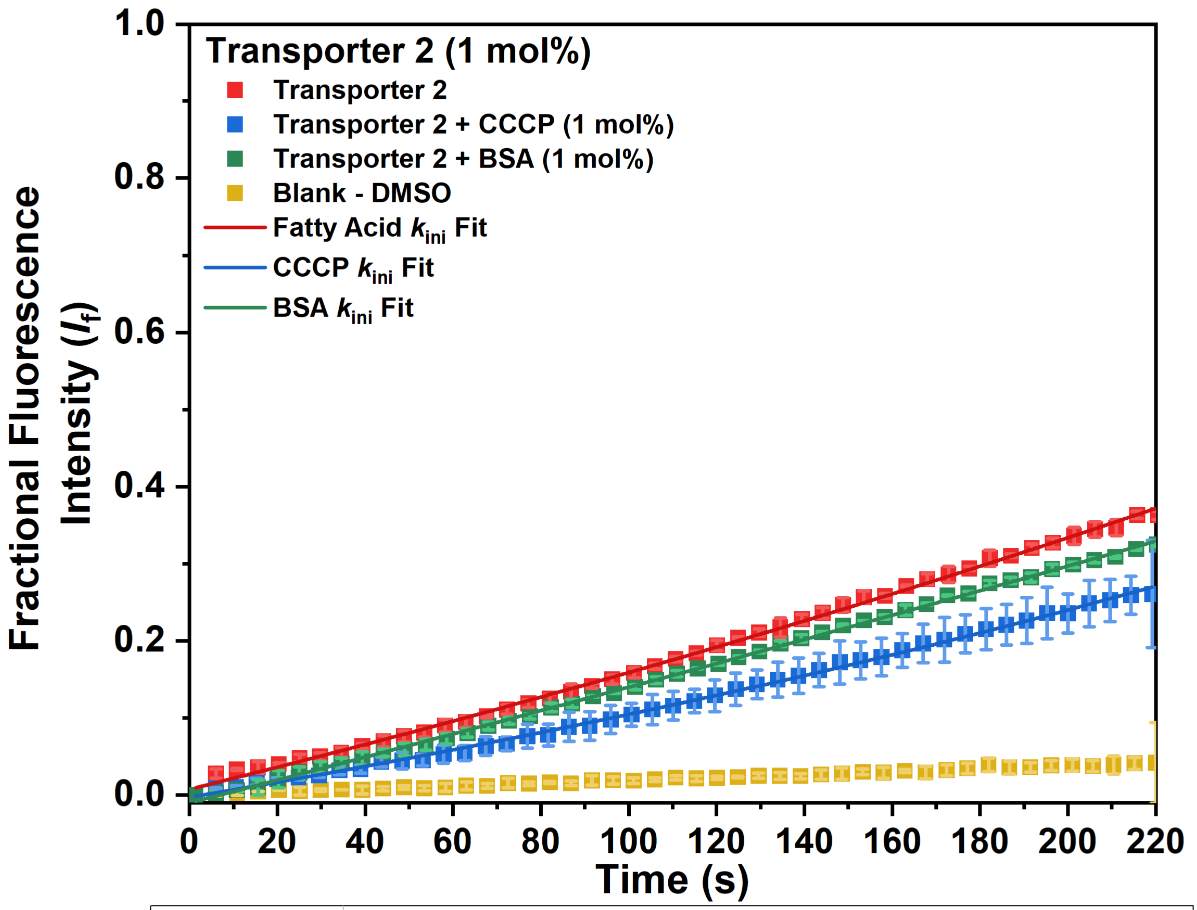

Model	ExpDec2		
Equation	y = A1*exp(-x/t1) + A2*exp(-x/t2) + y0		
Plot	Fractional Fluorescence Intensity	Fractional Fluorescence Intensity	Fractional Fluorescence Intensity
y0	-0.98806 ± 5.68449	-0.41423 ± 1.21977	-3.1952 ± 6.47238
A1	0.4937 ± 31271.34238	0.20449 ± 20855.31259	1.57243 ± 102182.38576
t1	-705.00807 ± 2.02344E7	-430.80389 ± 5276937.64	-2171.28061 ± 1.15861E8
A2	0.50172 ± 31272.48972	0.20682 ± 20855.29422	1.6126 ± 102181.83399
t2	-704.88748 ± 1.98949E7	-430.72311 ± 5213177.54	-2171.20629 ± 1.12959E8
Reduced Chi-Sq	2.38874E-5	1.37278E-5	1.0034E-5
R-Square (COD)	1	1	1
Adj. R-Square	1	1	1

Figure S60. The I_f facilitated by transporter **2** (1 mol%) in vesicles with membrane-embedded fatty acids (red), vesicles which have been treated with CCCP (1 mol%, blue), vesicles which have been pre-treated with BSA (1 mol%, green) to remove all fatty acids from vesicle membranes, and the control DMSO experiment (yellow) related to **Table 5**.

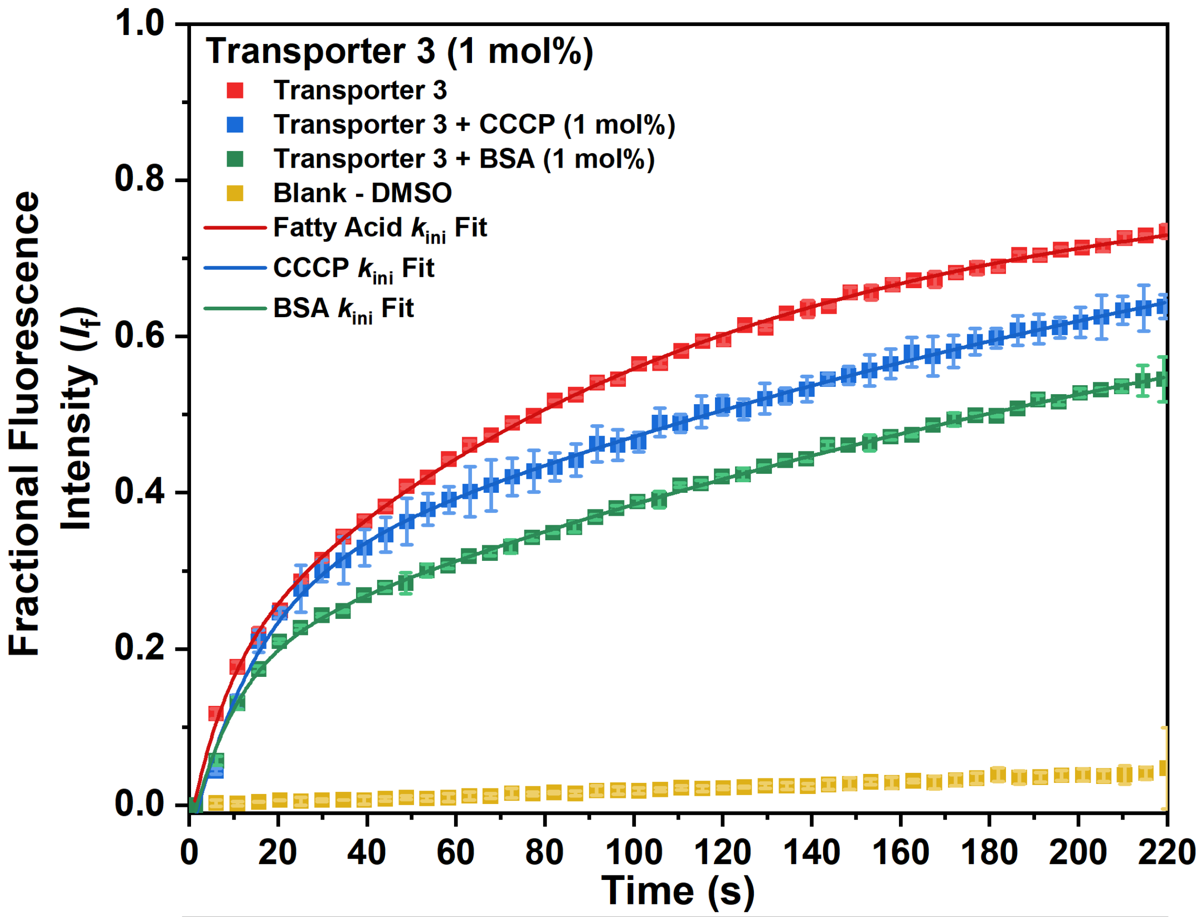

Model	ExpDec2		
Equation	y = A1*exp(-x/t1) + A2*exp(-x/t2) + y0		
Plot	Fractional Fluorescence Intensity	Fractional Fluorescence Intensity	Fractional Fluorescence Intensity
y0	0.81164 ± 0.00701	0.99318 ± 0.1122	0.85803 ± 0.05337
A1	-0.19543 ± 0.00824	-0.31362 ± 0.01412	-0.2238 ± 0.00754
t1	8.39321 ± 0.74794	14.49634 ± 1.22087	10.3715 ± 0.74556
A2	-0.64695 ± 0.00446	-0.7275 ± 0.09875	-0.67366 ± 0.04759
t2	106.44895 ± 3.44056	299.86863 ± 72.98476	282.9378 ± 35.19478
Reduced Chi-S	2.30768E-5	5.64281E-5	2.46898E-5
R-Square (COD	0.99935	0.99782	0.99867
Adj. R-Square	0.99929	0.99762	0.99855

Figure S61. The I_f facilitated by transporter **3** (1 mol%) in vesicles with membrane-embedded fatty acids (red), vesicles which have been treated with CCCP (1 mol%, blue), vesicles which have been pre-treated with BSA (1 mol%, green) to remove all fatty acids from vesicle membranes, and the control DMSO experiment (yellow) related to **Table 5.**

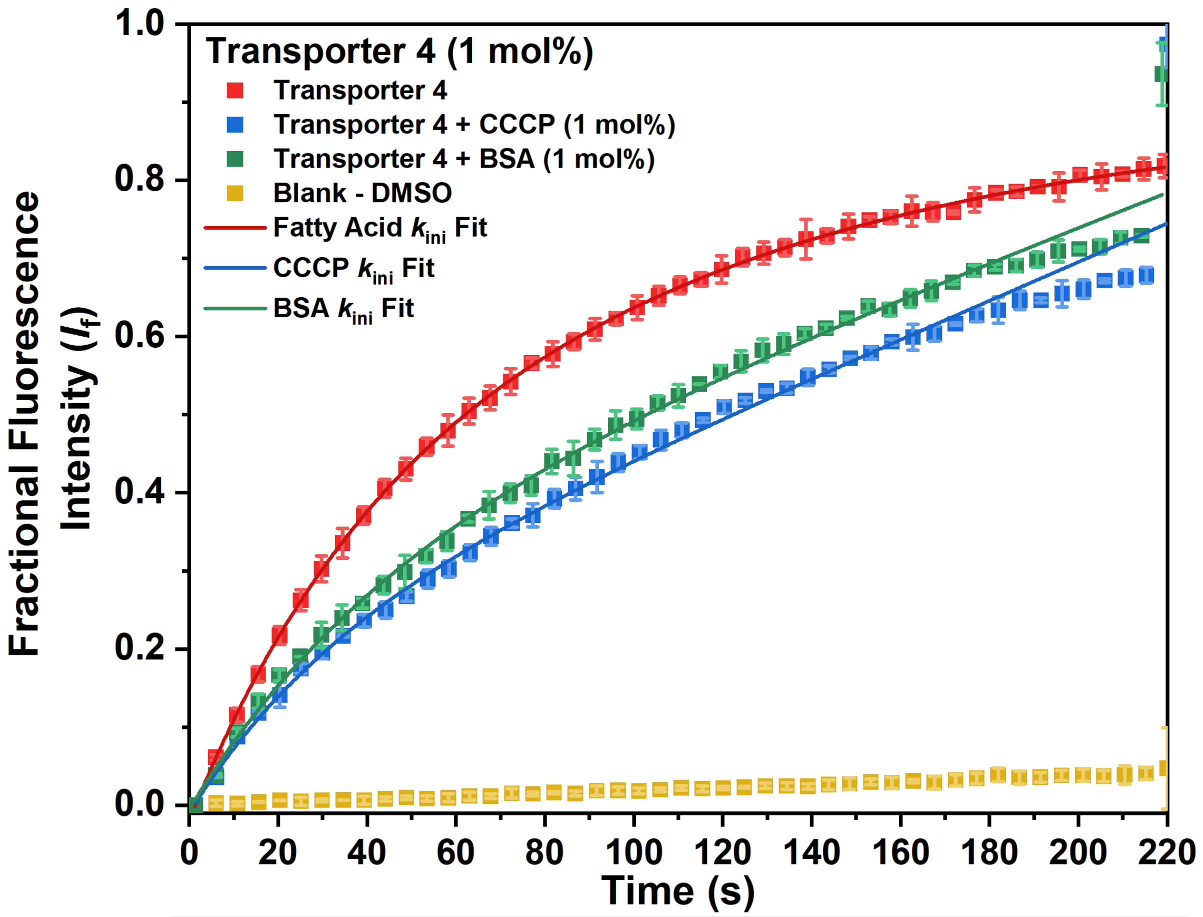

Model	ExpDec2		
Equation	y = A1*exp(-x/t1) + A2*exp(-x/t2) + y0		
Plot	Fractional Fluorescence Intensity	Fractional Fluorescence Intensity	Fractional Fluorescence Intensity
y0	0.89876 ± 0.01971	1347.00757 ± 434967.72	523.7428 ± 255807.16138
A1	-0.31484 ± 0.11511	-0.2125 ± 0.05212	-0.30608 ± 0.10418
t1	36.22206 ± 6.47005	31.83322 ± 15.09361	43.02998 ± 16.46938
A2	-0.6033 ± 0.09759	-1346.80463 ± 434967.70	-523.44165 ± 255807.065
t2	109.50202 ± 20.39008	546392.20529 ± 1.76519	237415.384 ± 1.16123E8
Reduced Chi-Sq	1.03427E-5	0.00155	7.58768E-4
R-Square (COD)	1	1	1
Adj. R-Square	1	1	1

Figure S62. The I_f facilitated by transporter **4** (1 mol%) in vesicles with membrane-embedded fatty acids (red), vesicles which have been treated with CCCP (1 mol%, blue), vesicles which have been pre-treated with BSA (1 mol%, green) to remove all fatty acids from vesicle membranes, and the control DMSO experiment (yellow) related to **Table 5**.

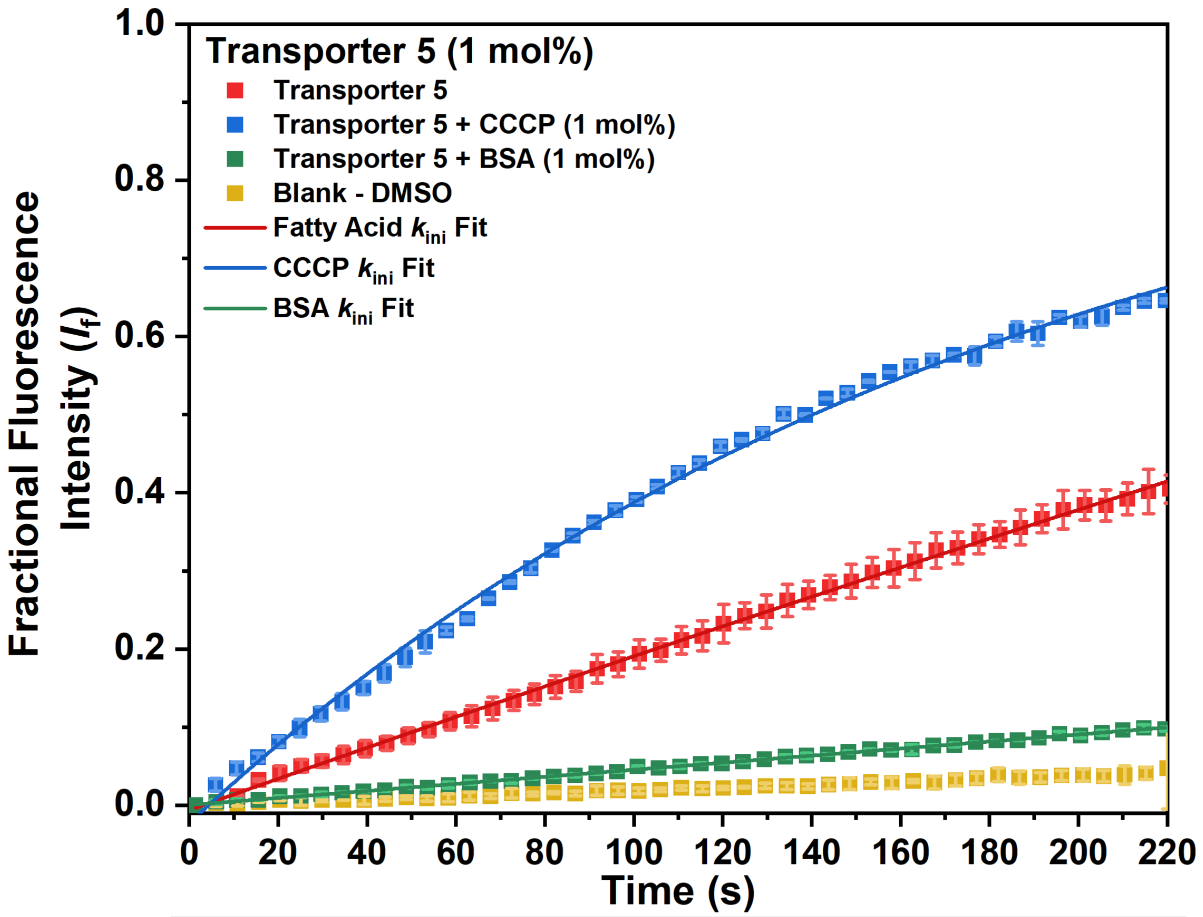

Model	ExpDec2		
Equation	y = A1*exp(-x/t1) + A2*exp(-x/t2) + y0		
Plot	Fractional Fluorescence Intensity	Fractional Fluorescence Intensity	Fractional Fluorescence Intensity
y0	4.01895 ± 105.77215	0.97062 ± 0.31312	127.96966 ± 7850.56653
A1	-1.91031 ± 57850.97596	-0.4895 ± 147699.2454	-59.07514 ± 16460.02457
t1	1993.40228 ± 3.96395E7	187.65821 ± 1229999.834	264953.89589 ± 6.40354
A2	-2.11491 ± 57897.92521	-0.50392 ± 147699.22964	-68.89409 ± 16935.67844
t2	1990.46008 ± 3.55641E7	187.64221 ± 1194803.099	301468.89142 ± 8.55896
Reduced Chi-Sqr	2.24307E-5	1.21523E-4	1.93768E-6
R-Square (COD)	1	1	1
Adj. R-Square	1	1	1

Figure S63. The I_f facilitated by transporter **5** (1 mol%) in vesicles with membrane-embedded fatty acids (red), vesicles which have been treated with CCCP (1 mol%, blue), vesicles which have been pre-treated with BSA (1 mol%, green) to remove all fatty acids from vesicle membranes, and the control DMSO experiment (yellow) related to **Table 5**.

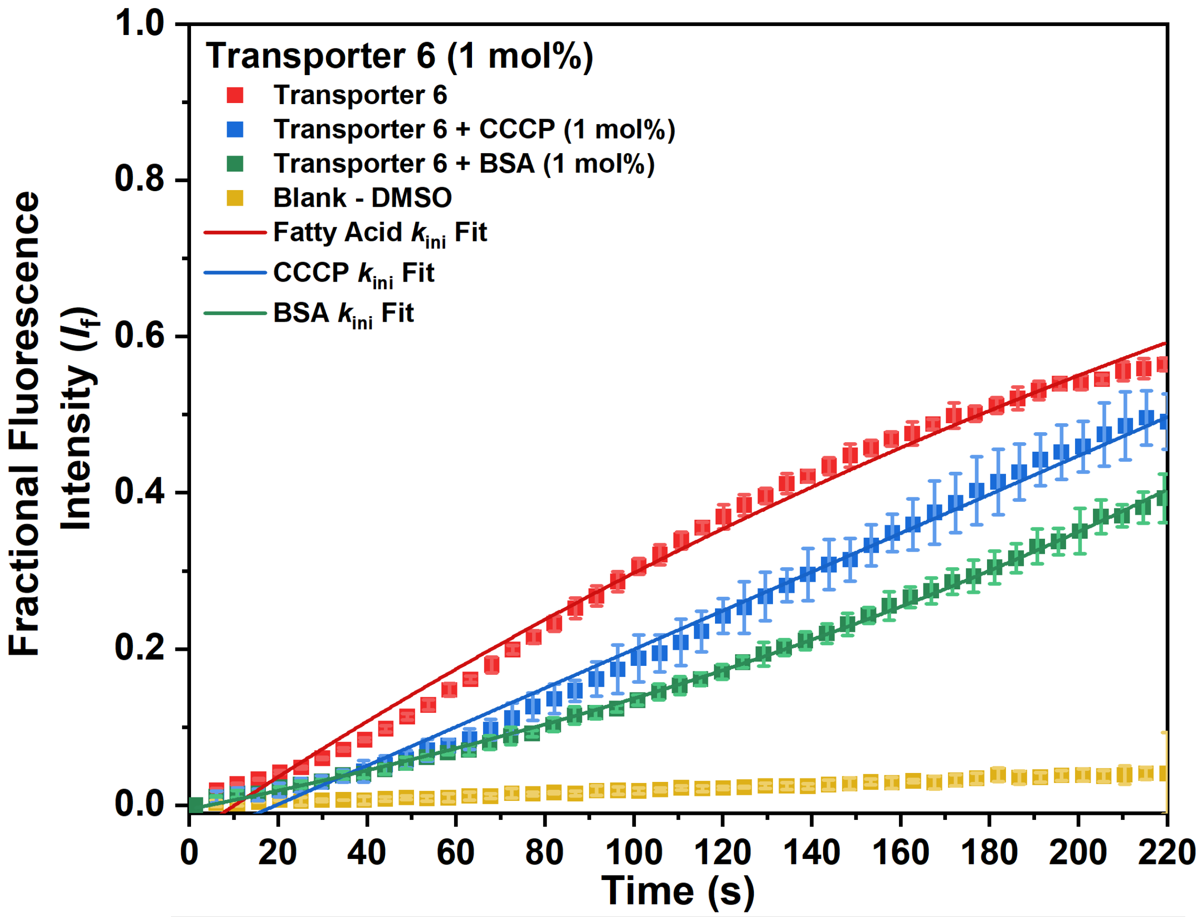

Model	ExpDec2		
Equation	y = A1*exp(-x/t1) + A2*exp(-x/t2) + y0		
Plot	Fractional Fluorescence Intensity	Fractional Fluorescence Intensity	Fractional Fluorescence Intensity
y0	1.31606 ± 1.47606	1270.88188 ± 102217.087	-0.28632 ± 0.08908
A1	-0.64878 ± 195340.17095	-722.25188 ± 158949.893	0.13999 ± 20287.10415
t1	350.66528 ± 7.71486E7	475940.18933 ± 9.55506	-244.82552 ± 3.48048E7
A2	-0.7061 ± 195340.09176	-548.67834 ± 129737.848	0.14123 ± 20287.09552
t2	350.66774 ± 7.08877E7	570655.47883 ± 1.28634	-244.82518 ± 3.45009E7
Reduced Chi-Sq	3.16081E-4	3.13157E-4	2.1109E-5
R-Square (COD)	1	1	1
Adj. R-Square	1	1	1

Figure S64. The I_f facilitated by transporter **6** (1 mol%) in vesicles with membrane-embedded fatty acids (red), vesicles which have been treated with CCCP (1 mol%, blue), vesicles which have been pre-treated with BSA (1 mol%, green) to remove all fatty acids from vesicle membranes, and the control DMSO experiment (yellow) related to **Table 5**.

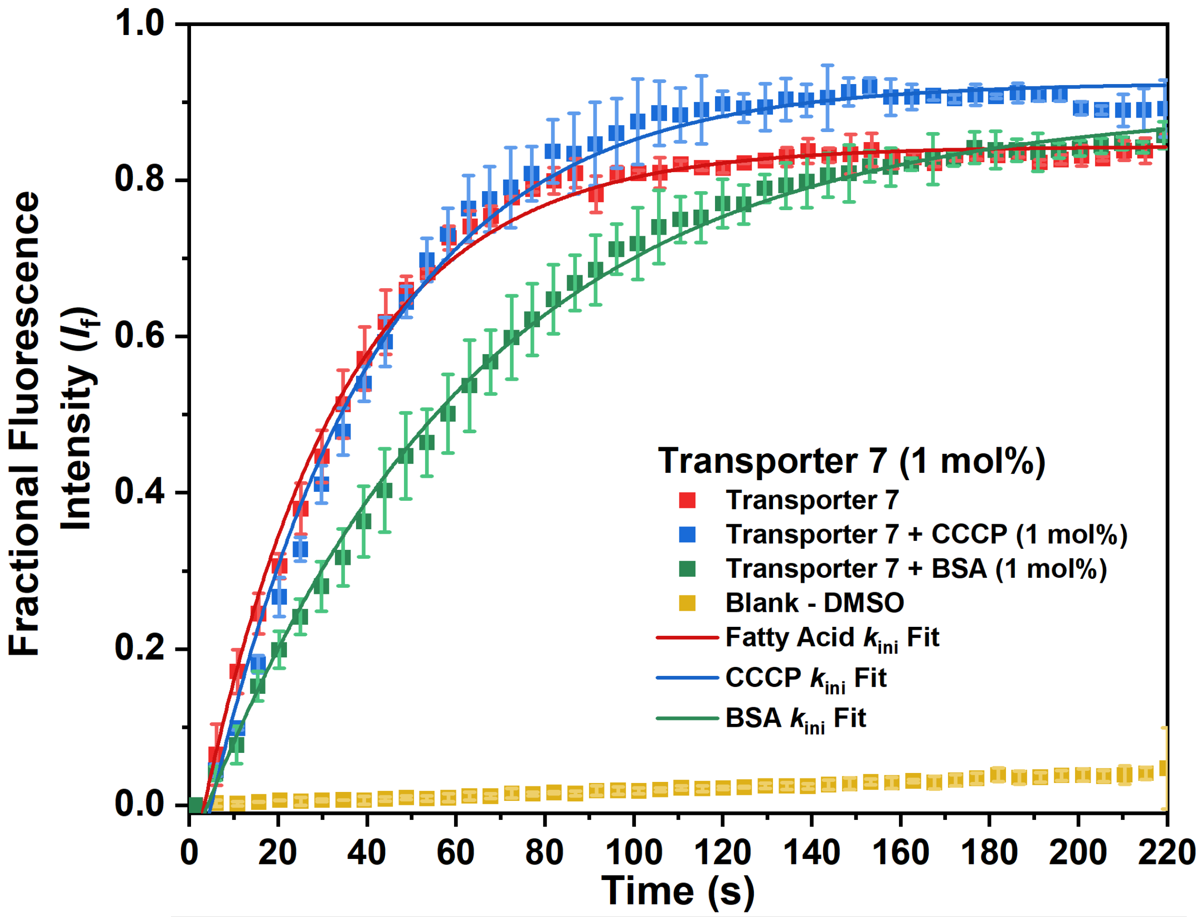

Model	ExpDec2		
Equation	y = A1*exp(-x/t1) + A2*exp(-x/t2) + y0		
Plot	Fractional Fluorescence Intensity	Fractional Fluorescence Intensity	Fractional Fluorescence Intensity
y0	0.84324 ± 0.00636	0.92486 ± 0.01177	0.89395 ± 0.0204
A1	-0.383 ± 68964.36823	-0.51343 ± 149884.7233	-0.46929 ± 74226.27597
t1	31.66713 ± 196827.02538	37.48155 ± 122042.41252	62.62131 ± 1537694.5012
A2	-0.55539 ± 68964.36721	-0.54124 ± 149884.727	-0.48529 ± 74226.27292
t2	31.66672 ± 135732.02104	37.48048 ± 115759.95796	62.62107 ± 1486993.0065
Reduced Chi-Sq	3.79493E-4	7.83561E-4	2.35264E-4
R-Square (COD)	1	1	1
Adj. R-Square	1	1	1

Figure S65. The I_f facilitated by transporter **7** (1 mol%) in vesicles with membrane-embedded fatty acids (red), vesicles which have been treated with CCCP (1 mol%, blue), vesicles which have been pre-treated with BSA (1 mol%, green) to remove all fatty acids from vesicle membranes, and the control DMSO experiment (yellow) related to **Table 5**.

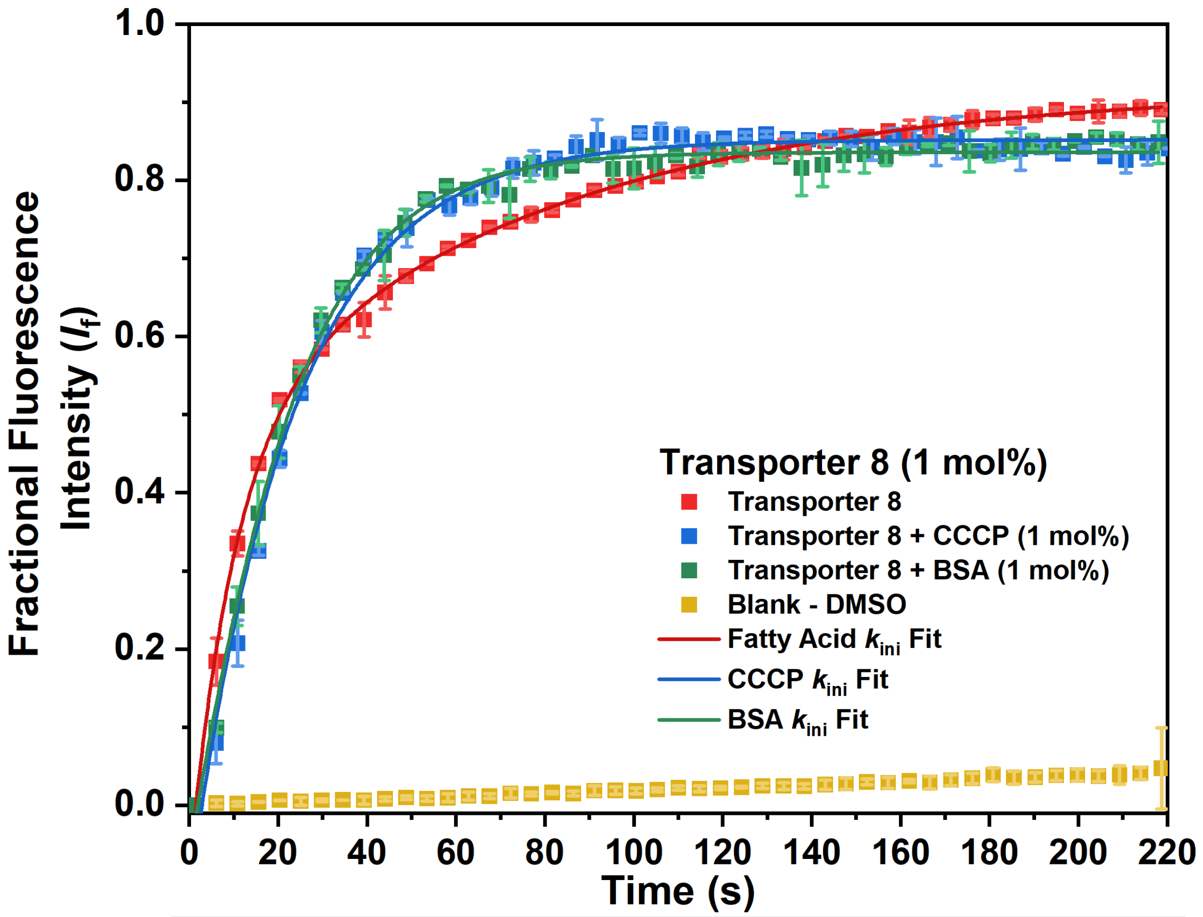

Model	ExpDec2		
Equation	y = A1*exp(-x/t1) + A2*exp(-x/t2) + y0		
Plot	Fractional Fluorescence Intensity	Fractional Fluorescence Intensity	Fractional Fluorescence Intensity
y0	0.91851 ± 0.00539	0.85173 ± 0.00432	0.83595 ± 0.00246
A1	-0.55602 ± 0.01477	-0.54788 ± 27689.85766	0.34569 ± 0.14855
t1	10.17153 ± 0.47136	23.04199 ± 23607.52497	10.17266 ± 0
A2	-0.44769 ± 0.01173	-0.41655 ± 27689.85359	-1.24366 ± 0.13794
t2	75.66679 ± 4.20541	23.04316 ± 31047.90419	18.55779 ± 1.03451
Reduced Chi-Sq	3.77946E-5	3.3773E-4	1.61356E-4
R-Square (COD)	1	1	1
Adj. R-Square	1	1	1

Figure S66. The I_f facilitated by transporter **8** (1 mol%) in vesicles with membrane-embedded fatty acids (red), vesicles which have been treated with CCCP (1 mol%, blue), vesicles which have been pre-treated with BSA (1 mol%, green) to remove all fatty acids from vesicle membranes, and the control DMSO experiment (yellow) related to **Table 5**.

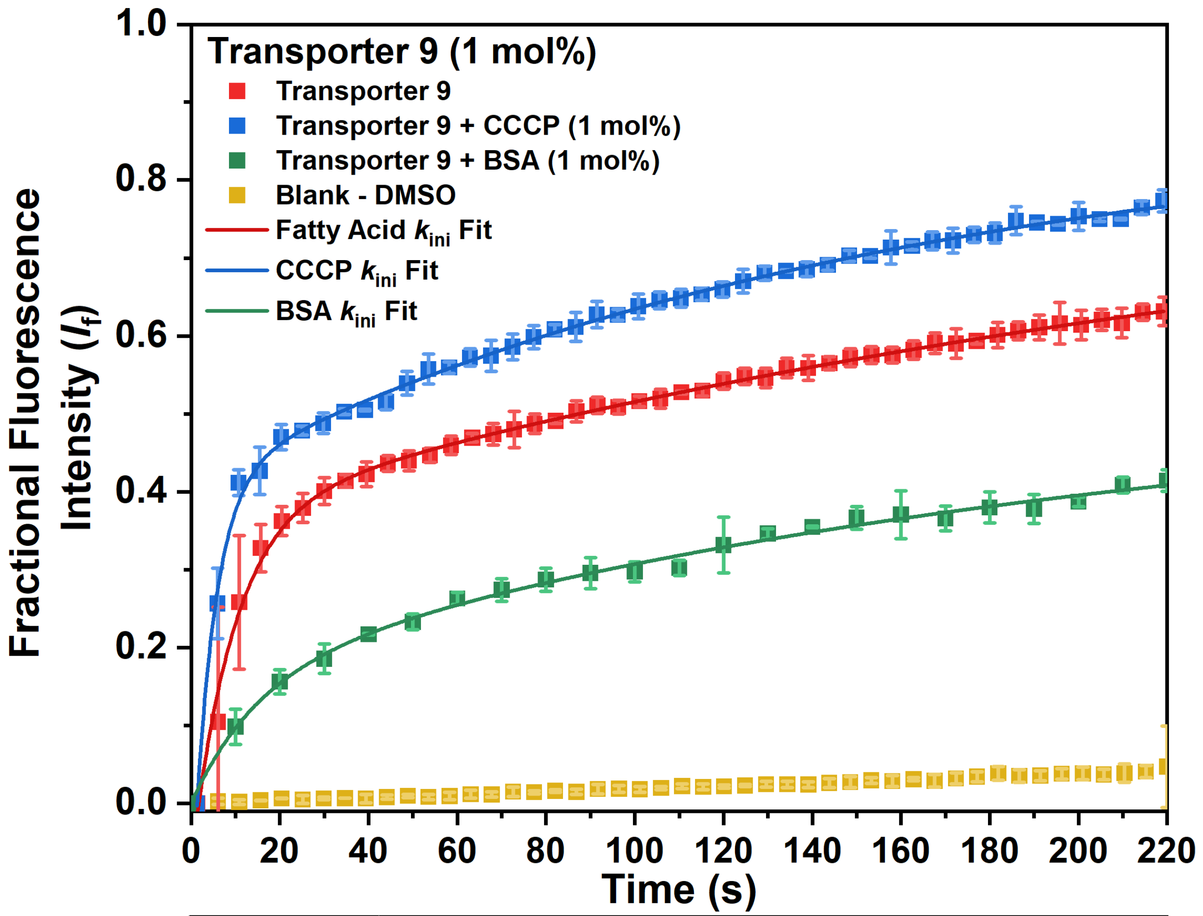

Model	ExpDec2		
Equation	y = A1*exp(-x/t1) + A2*exp(-x/t2) + y0		
Plot	Fractional Fluorescence Intensity	Fractional Fluorescence Intensity	Fractional Fluorescence Intensity
y0	0.84771 ± 0.0795	0.87943 ± 0.01713	0.5302 ± 0.07149
A1	-0.44717 ± 0.01207	-0.55228 ± 0.01223	-0.16126 ± 0.02491
t1	9.5302 ± 0.55612	4.69267 ± 0.2249	15.12788 ± 3.36189
A2	-0.47682 ± 0.0709	-0.46773 ± 0.01303	-0.3682 ± 0.04976
t2	276.65487 ± 72.70219	154.57785 ± 11.63457	198.77882 ± 76.86377
Reduced Chi-Sqr	6.53637E-5	4.67308E-5	5.95975E-5
R-Square (COD)	0.99637	0.99795	0.99555
Adj. R-Square	0.99602	0.99776	0.99456

Figure S67. The I_f facilitated by control transporter **9** (1 mol%) in vesicles with membrane-embedded fatty acids (red), vesicles which have been treated with CCCP (1 mol%, blue), vesicles which have been pre-treated with BSA (1 mol%, green) to remove all fatty acids from vesicle membranes, and the control DMSO experiment (yellow) related to **Table 5**.

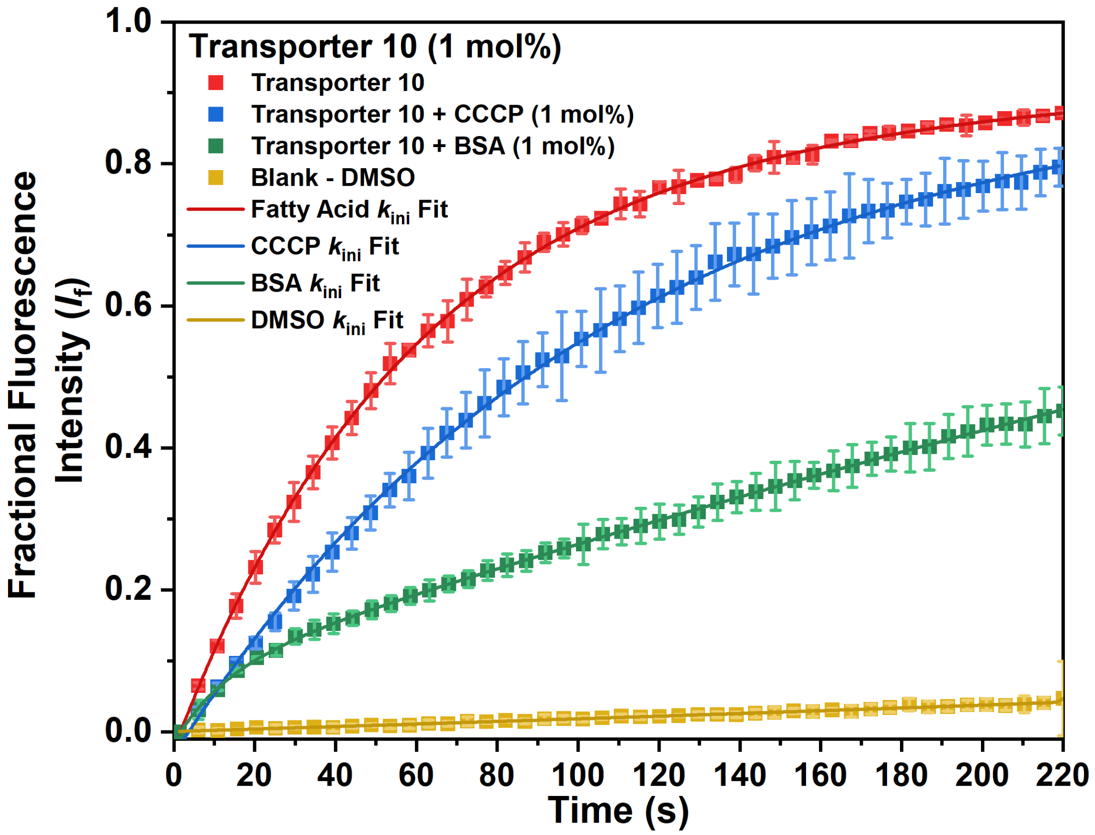

Model	ExpDec2		
Equation	y = A1*exp(-x/t1) + A2*exp(-x/t2) + y0		
Plot	Fatty Acid	CCCP	BSA
y0	5.73504 ± 426.42147	0.91661 ± 0.03621	1.60077 ± 0.2641
A1	-0.87269 ± 0.02408	-0.47444 ± 89612.43098	-0.09732 ± 0.00435
t1	58.83583 ± 1.60079	105.75952 ± 1.71768E7	12.81565 ± 1.20151
A2	-4.88597 ± 426.40377	-0.47403 ± 89612.42812	-1.51808 ± 0.26031
t2	24892.01641 ± 2191356.15147	105.75958 ± 1.71919E7	783.67242 ± 162.06315
Reduced Chi-Sqr	2.00964E-5	5.19353E-5	8.25996E-6
R-Square (COD)	1	1	1
Adj. R-Square	1	1	1

Model	ExpDec2
Equation	y = A1*exp(-x/t1) + A2*exp(-x/t2) + y0
Plot	DMSO
y0	-0.23128 ± 0.19788
A1	5.66412E-105 ± 1.11095E-101
t1	-0.93685 ± 7.82556
A2	0.23211 ± 0.19748
t2	-1346.78291 ± 1060.3550
Reduced Chi-Sq	1.56223E-6
R-Square (COD)	1
Adj. R-Square	1

Figure S68. The I_f facilitated by control transporter **10** (1 mol%) in vesicles with membrane-embedded fatty acids (red), vesicles which have been treated with CCCP (1 mol%, blue), vesicles which have been pre-treated with BSA (1 mol%, green) to remove all fatty acids from vesicle membranes, and the control DMSO experiment (yellow) related to **Table 5**.

S5. ^{1}H-NMR binding studies:

S5.1 The calculated covariance of fit, enhancement factors, and overall binding constants:

Table S1. The 1:1 and 1:2 Host:Guest Model Covariance of Fit (cov_{fit}), the Covariance of Fit Enhancement Factor ($Fcov_{fit}$), and the Overall Binding Constant (β_{12}, M^{-2}) of Transporters **1**–**8** in Relation to the Tested Anions, related to **Table 3** and the Star Methods section.

Transporter	Anion	1:1 cov_{fit} [a]	1:2 cov_{fit} [b]	$Fcov_{fit}$ [c]	β_{12} [M^{-2}] [d]
**1**	Cl^-	5.46×10^{-3}	2.39×10^{-3}	2.28	1.24×10^{4}
**2**	Cl^-	5.41×10^{-3}	1.36×10^{-3}	3.98	1.04×10^{3}
**3**	Cl^-	2.94×10^{-3}	1.72×10^{-3}	1.71	2.26×10^{4}
	NO_3^-	1.64×10^{-4}	[f]	[e]	[e]
**4**	Cl^-	6.77×10^{-6}	5.26×10^{-6}	1.29	1.90×10^{3}
	NO_3^-	8.56×10^{-3}	[f]	[e]	[e]
**5**	Cl^-	3.22×10^{-3}	1.93×10^{-4}	16.72	6.91×10^{2}
	NO_3^-	1.30×10^{-3}	[f]	[e]	[e]
**6**	Cl^-	1.29×10^{-3}	1.51×10^{-4}	8.59	7.12×10^{2}
	NO_3^-	8.49×10^{-4}	[f]	[e]	[e]
**7**	Cl^-	9.36×10^{-4}	1.53×10^{-4}	6.14	2.15×10^{3}
**8**	Cl^-	1.06×10^{-3}	5.96×10^{-5}	17.76	1.42×10^{3}
**9**	Cl^-	1.27×10^{-3} [g]	4.14×10^{-4} [g]	3.06 [g]	1.27×10^{4} [g]
**10**	Cl^-	1.73×10^{-3} [g]	1.51×10^{-3} [g]	1.14 [g]	1.36×10^{4} [g]

[a] The covariance of fit for anion binding data which was fit to 1:1 (1:1 cov_{fit}) and [b] 1:2 (1:2 cov_{fit}) host:guest binding models, was calculated using **Equation 1**–**2**. [c] The enhancement factor for the covariance of fit ($Fcov_{fit}$) was calculated using **Equation 3**. [d] The overall binding constants (β_{12}) were calculated using **Equation 4**. [e] Not determined. [f] The experimental data could not fit a 1:2 binding model. [g] The 1:1 and 1:2 cov_{fit}, $Fcov_{fit}$, and β_{12} values for control transporters **9** and **10** have been previously reported.[5]

S5.2 Anion binding studies of transporter 1:

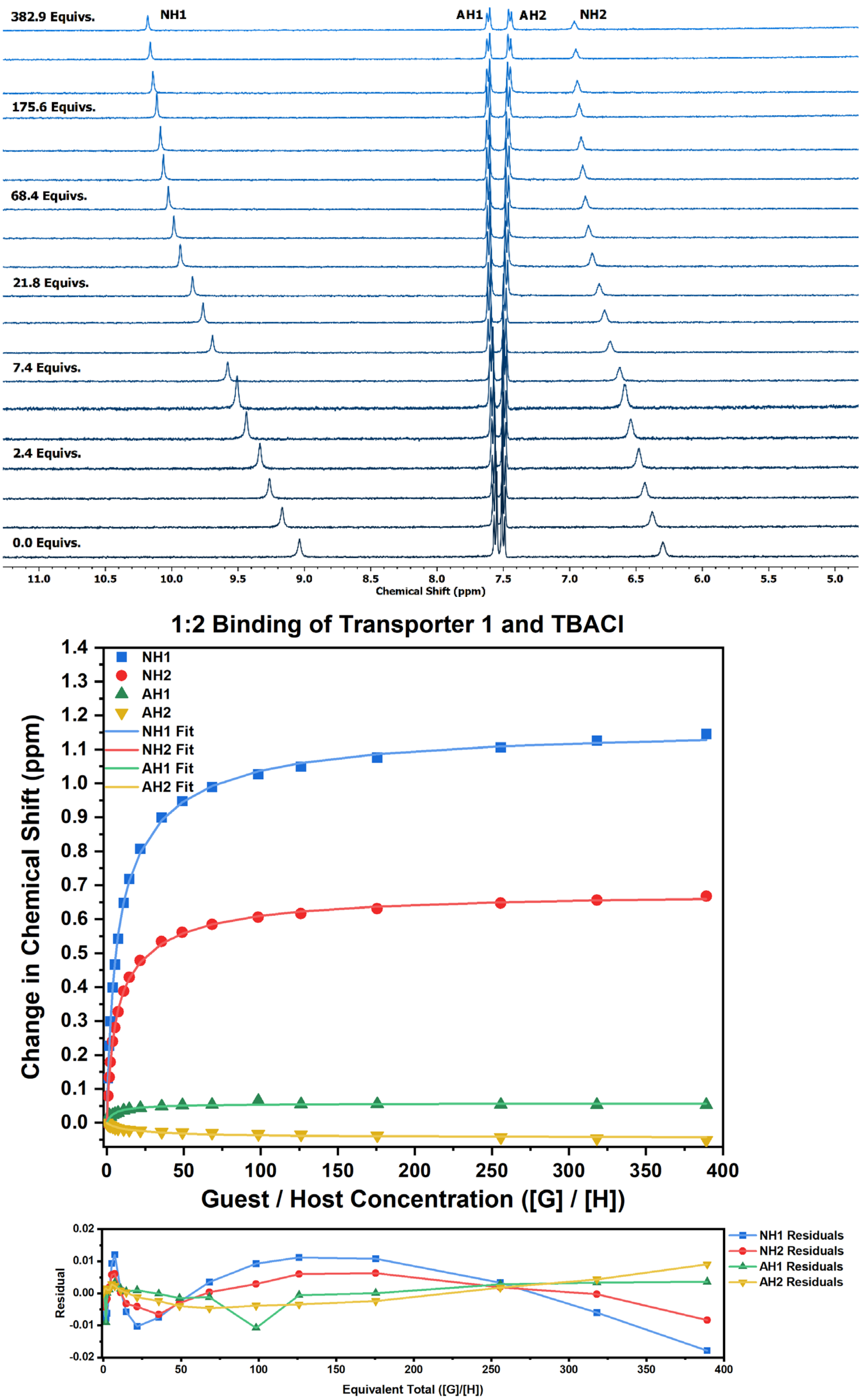

Figure S69. The ^{1}H-NMR stacked spectrum of transporter **1** (1 mM) in DMSO-d_6/0.5% H_2O at 298 K (top) when titrated with TBACl (0–389.2 equivs.), related to **Table 3**. The fitted binding data of **1** and the residual error obtained from fitting to the 1:2 binding model (bottom) K_{11} = 371 (± 3.16 %), K_{12} = 33.4 (± 3.10 %).

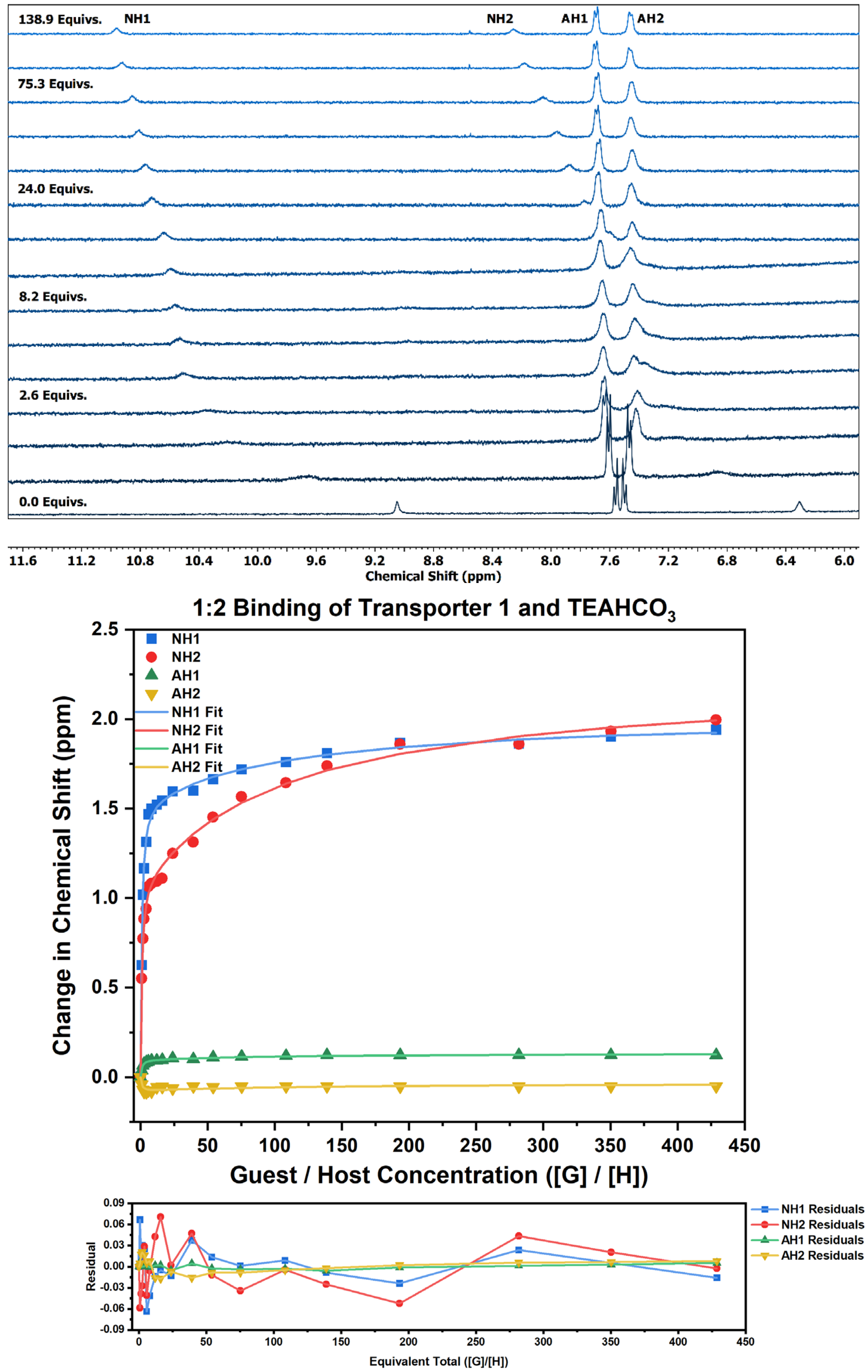

Figure S70. The ^{1}H-NMR stacked spectrum of transporter **1** (1 mM) in DMSO-*d*6/0.5% H_2O at 298 K (top) when titrated with $TEAHCO_3$ (0–428.7 equivs.), related to **Table 3**. Minimal peak shifts occurred after the addition of ~138.9 equivs. of the guest, and the subsequent spectrums were removed for clarity.

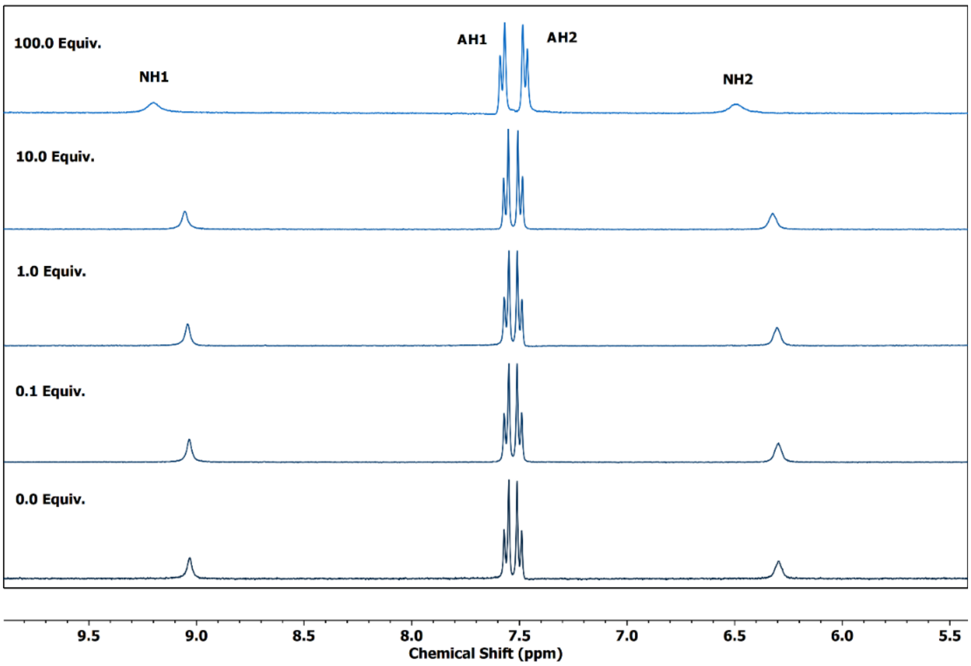

Figure S71. The ^{1}H-NMR stacked spectrum of transporter **1** (1 mM) in DMSO-d_6/0.5% H_2O at 298 K when titrated with $TBANO_3$ (0–100.0 equivs.), related to **Table 3**.

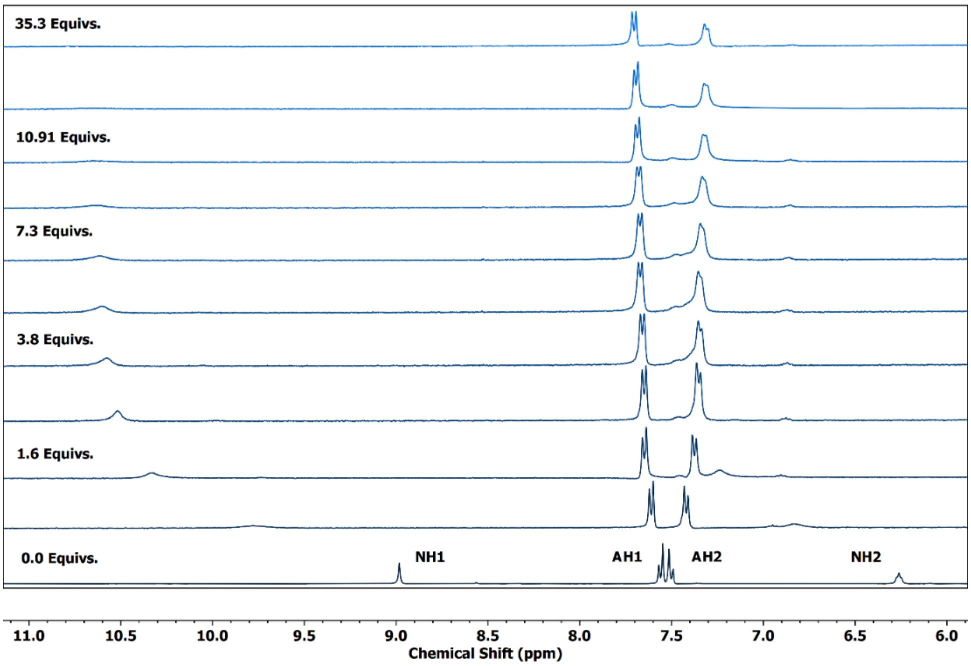

Figure S72. The ^{1}H-NMR stacked spectrum of transporter **1** (1 mM) in DMSO-d_6/0.5% H_2O at 298 K when titrated with $TBAH_2PO_4$ (0–35.3 equivs.), related to **Table 3**.

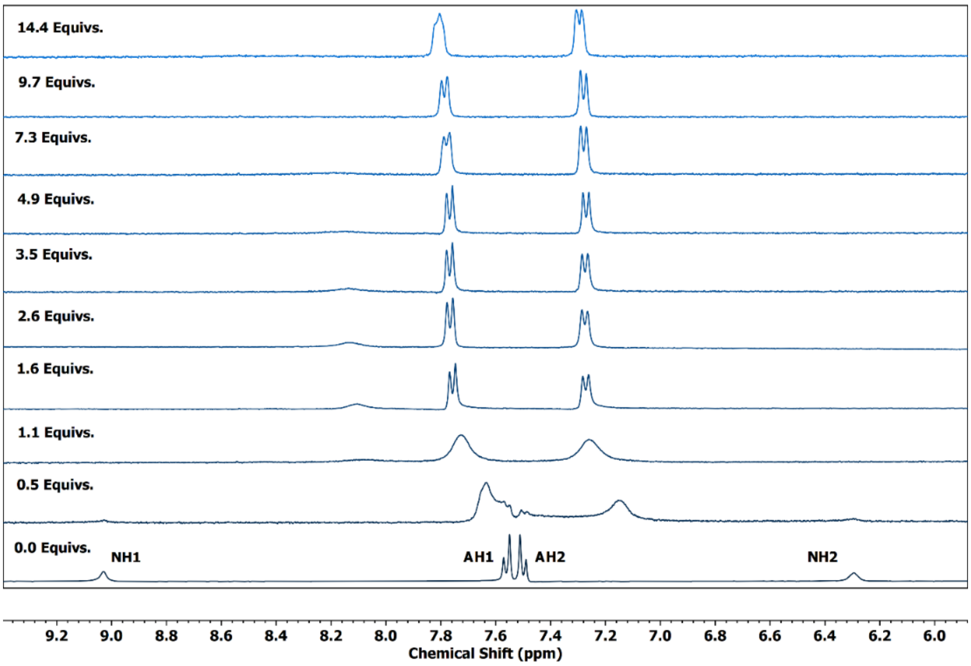

Figure S73. The ^{1}H-NMR stacked spectrum of transporter **1** (1 mM) in DMSO-d_6/0.5% H_2O at 298 K when titrated with $(TBA)_3HP_2O_7$ (0–35.3 equivs.) related to **Table 3**.

S5.3 Anion binding studies of transporter 2:

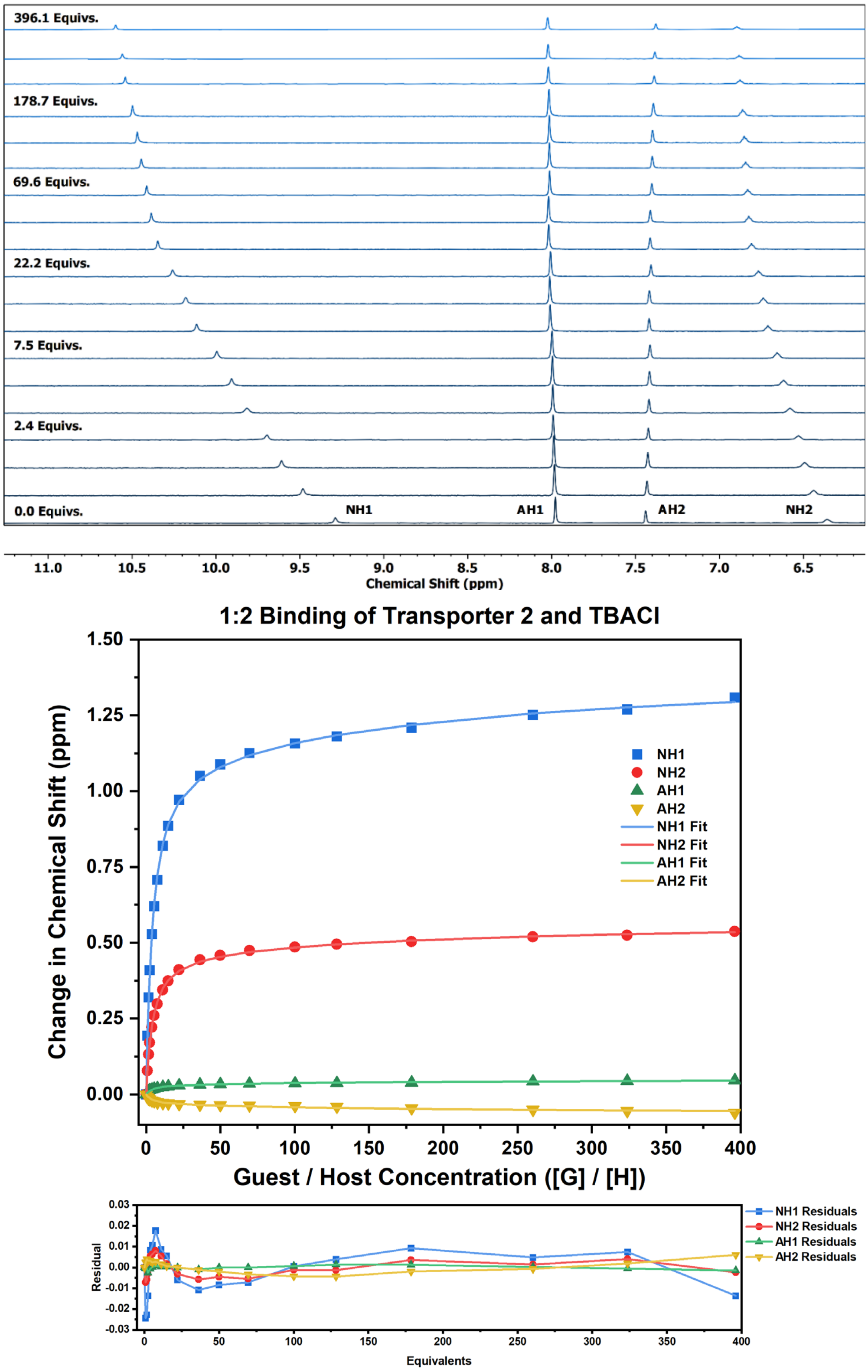

Figure S74. The ^{1}H-NMR stacked spectrum of transporter **2** (1 mM) in DMSO-d_6/0.5% H_2O at 298 K (top) when titrated with TBACl (0–396.1 equivs.), related to **Table 3**. The fitted binding data of **2** and the residual error obtained from fitting to the 1:2 binding model (bottom) K_{11} = 271 (± 1.87 %), K_{12} = 3.84 (± 5.39 %).

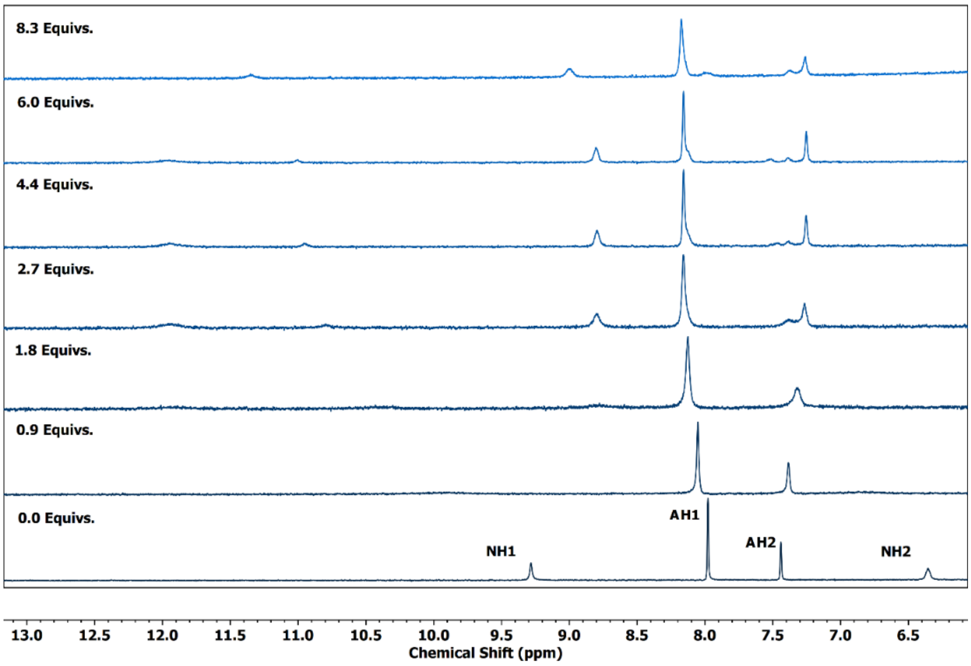

Figure S75. The ^{1}H-NMR stacked spectrum of transporter **2** (1 mM) in DMSO-d_6/0.5% H_2O at 298 K when titrated with $TEAHCO_3$ (0–8.3 equivs.) related to **Table 3**.

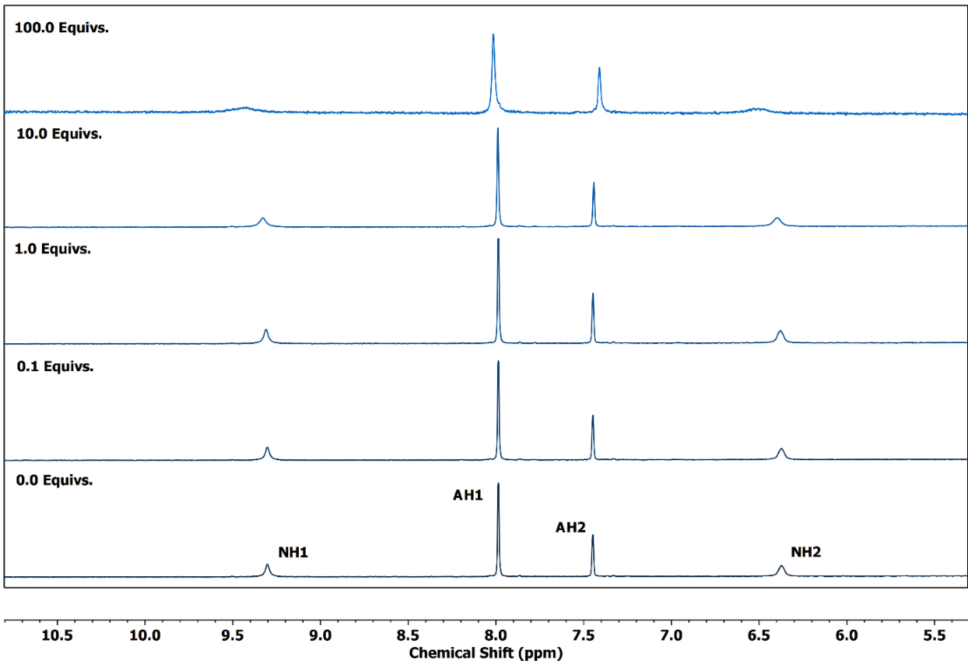

Figure S76. The ^{1}H-NMR stacked spectrum of transporter **2** (1 mM) in DMSO-d_6/0.5% H_2O at 298 K when titrated with $TBANO_3$ (0–100.0 equivs.), related to **Table 3**.

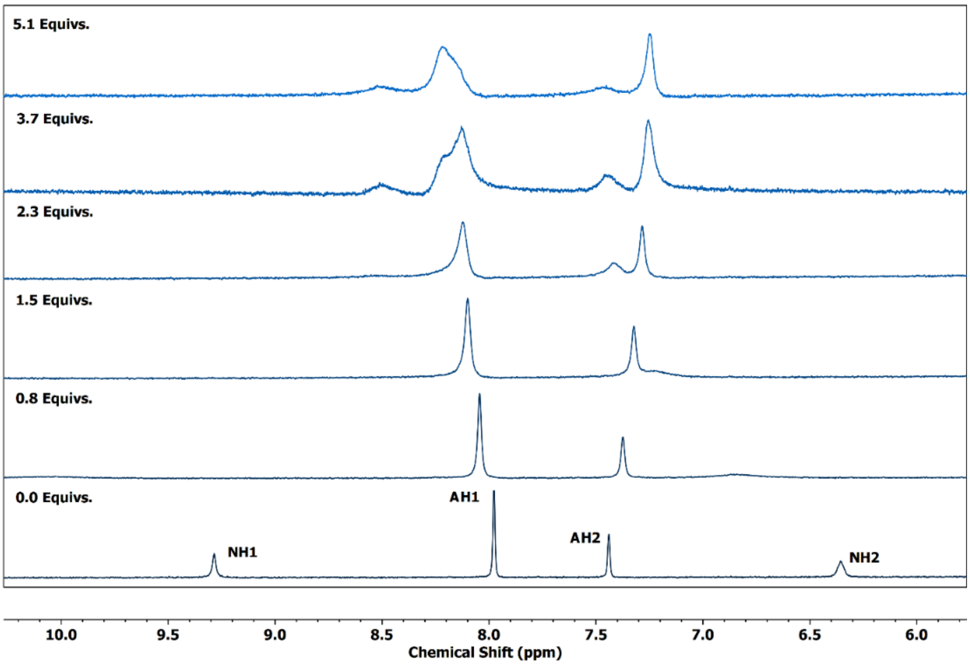

Figure S77. The ^{1}H-NMR stacked spectrum of transporter **2** (1 mM) in DMSO-d_6/0.5% H_2O at 298 K when titrated with $TBAH_2PO_4$ (0–5.1 equivs.), related to **Table 3**.

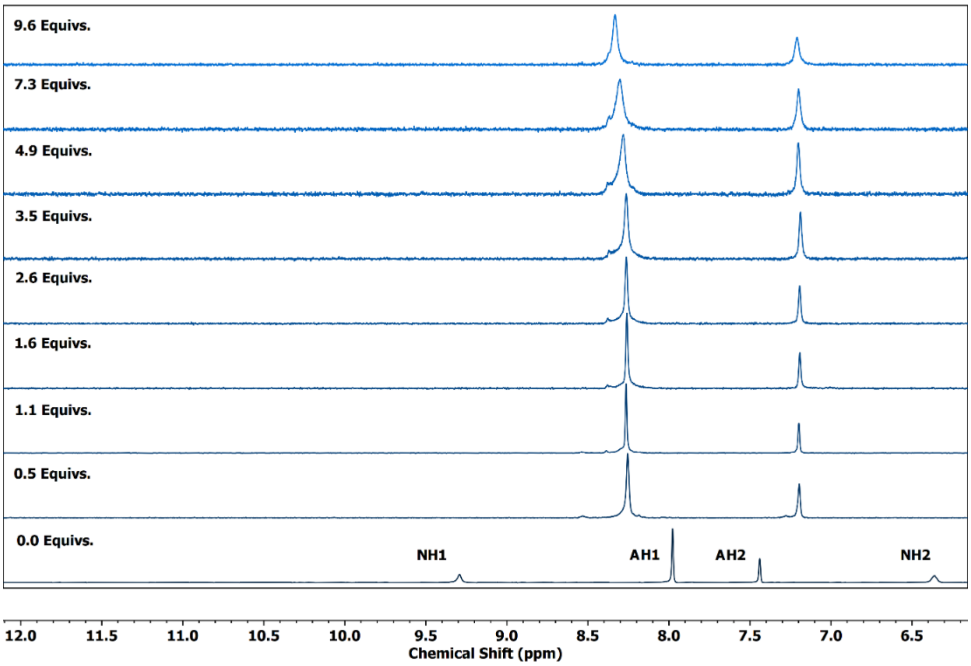

Figure S78. The ^{1}H-NMR stacked spectrum of transporter **2** (1 mM) in DMSO-d_6/0.5% H_2O at 298 K when titrated with $(TBA)_3HP_2O_7$ (0–9.6 equivs.) related to **Table 3**.

S5.4 Anion binding studies of transporter 3:

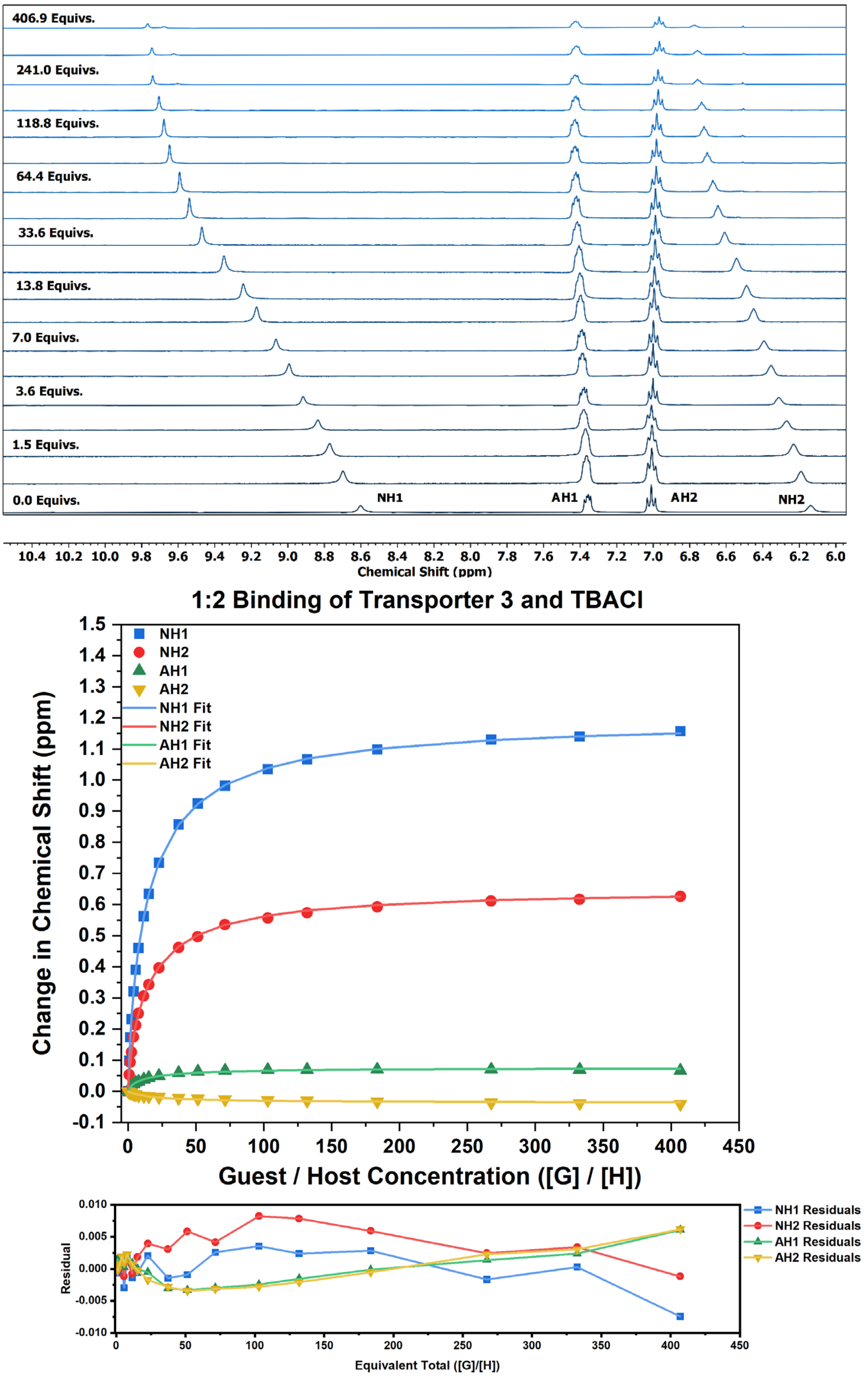

Figure S79. The ^{1}H-NMR stacked spectrum of transporter **3** (1 mM) in DMSO-d_6/0.5% H_2O at 298 K (top) when titrated with TBACl (0–406.9 equivs.), related to **Table 3**. The fitted binding data of **3** and the residual error obtained from fitting to the 1:2 binding model (bottom) K_{11} = 507 (± 4.3 %), K_{12} = 44.5 (± 3.07 %).

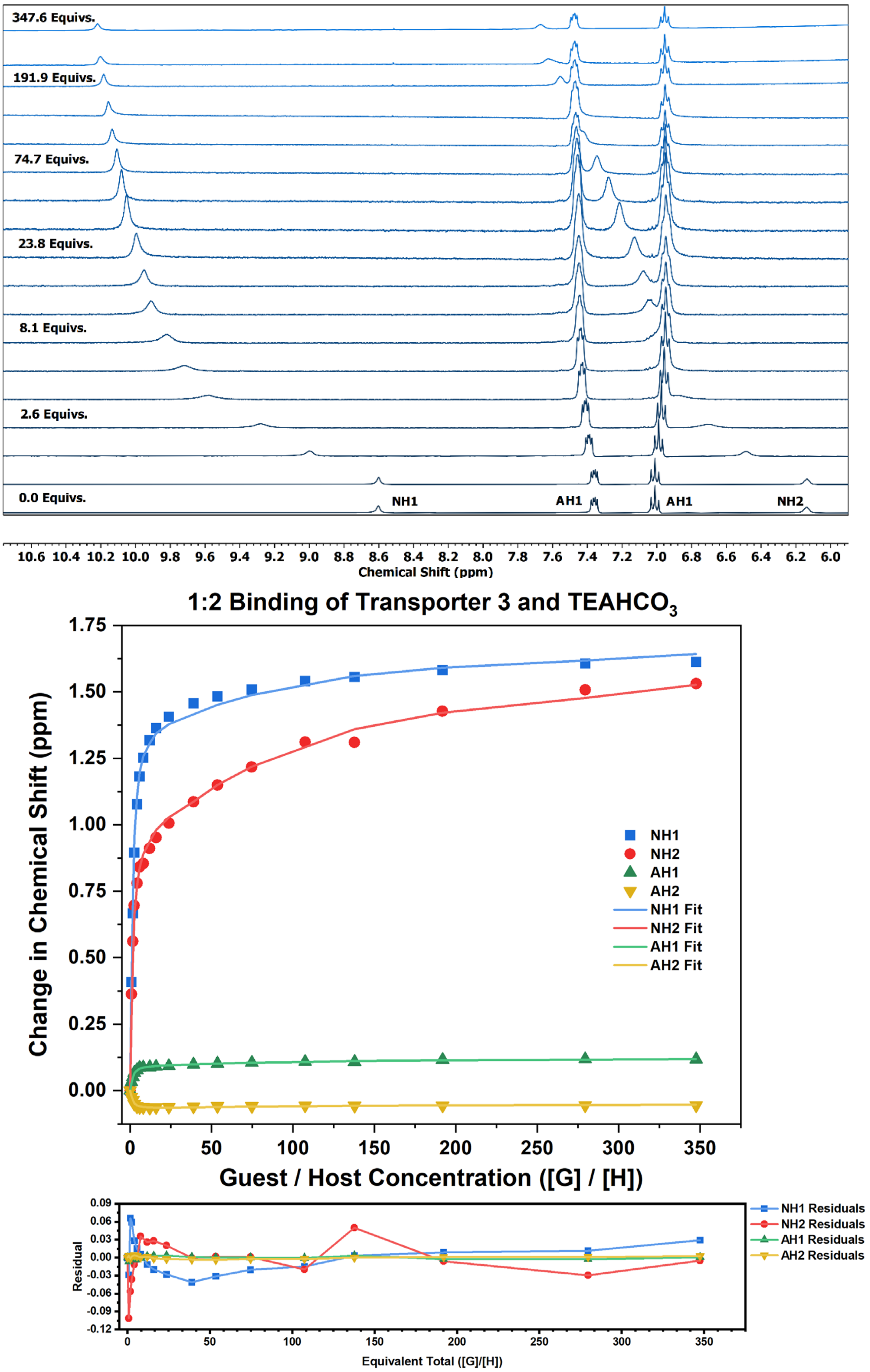

Figure S80. The ^{1}H-NMR stacked spectrum of transporter **3** (1 mM) in DMSO-d_6/0.5% H_2O at 298 K (top) when titrated with $TEAHCO_3$ (0–347.6 equivs.) related to **Table 3**.

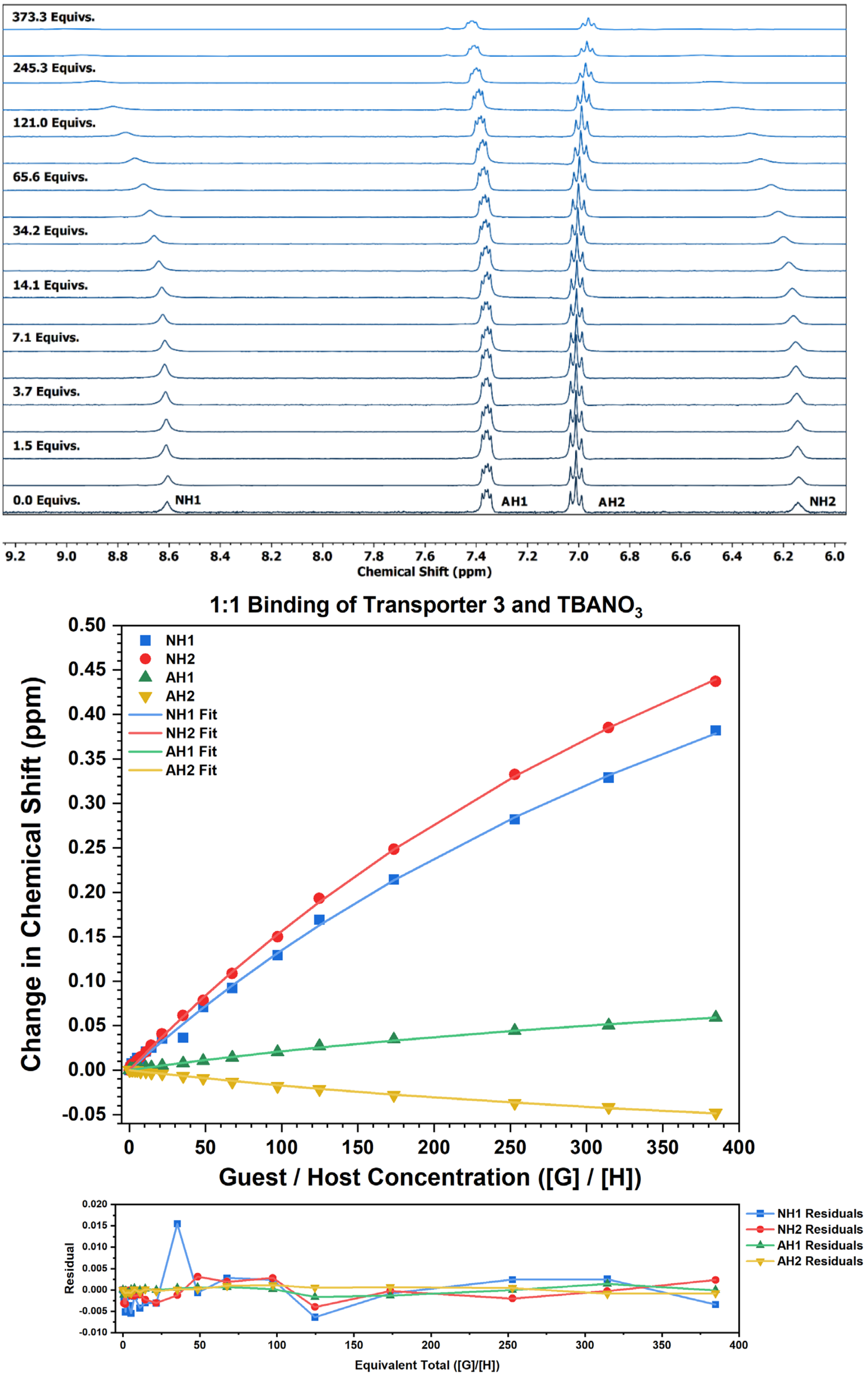

Figure S81. The ^{1}H-NMR stacked spectrum of transporter **3** (1 mM) in DMSO-d_6/0.5% H_2O at 298 K (top) when titrated with $TBANO_3$ (0–373.3 equivs.) related to **Table 3**. The fitted binding data of **3** and the residual error obtained from fitting to the 1:1 binding model (bottom) K_a = 1.33 (± 1.1 %).

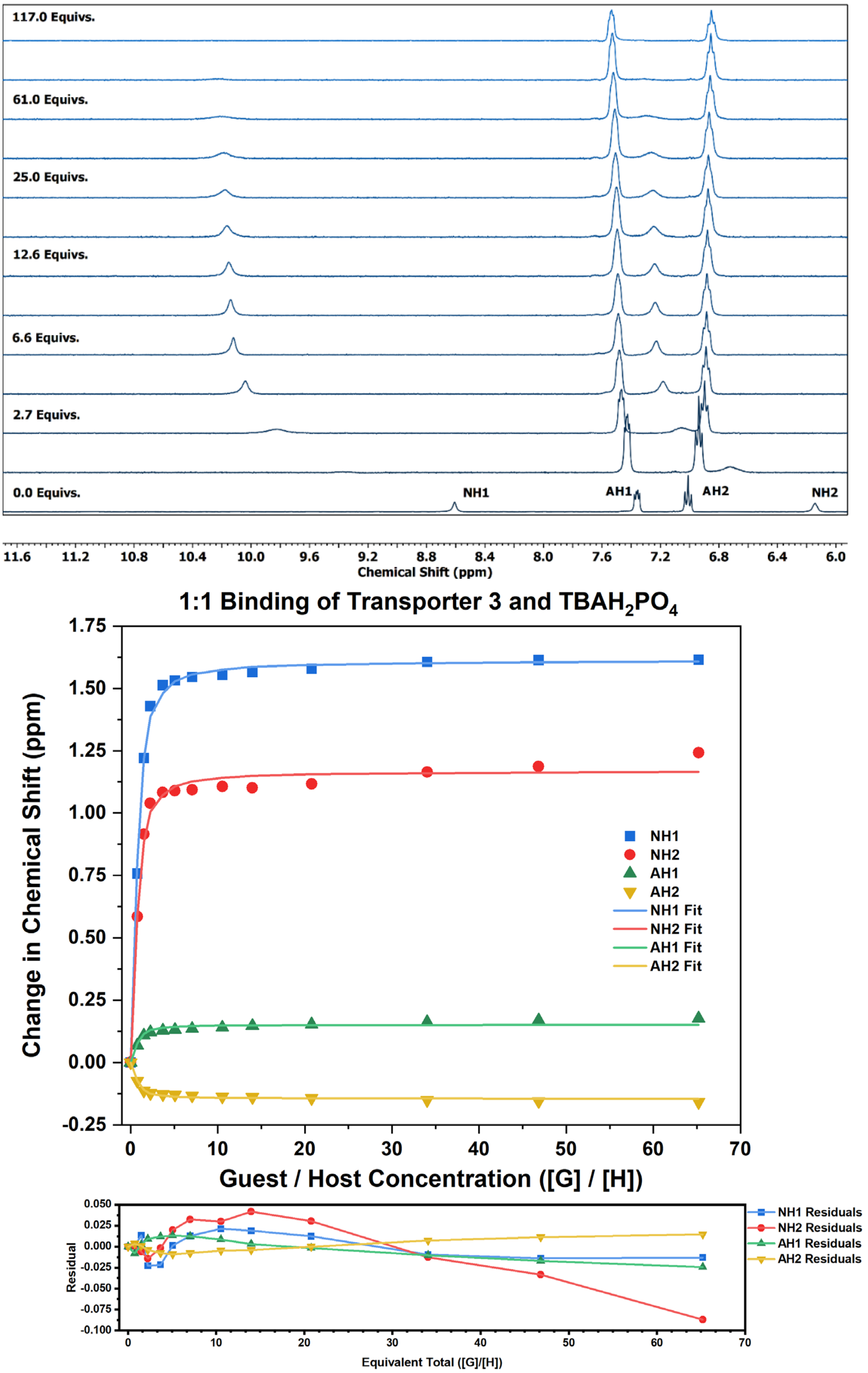

Figure S82. The ^{1}H-NMR stacked spectrum of transporter **3** (1 mM) in DMSO-d_6/0.5% H_2O at 298 K (top) when titrated with $TBAH_2PO_4$ (0–117.0 equivs.), related to **Table 3**. Complete deprotonation of the NH protons was observed after ~61.0 equivs. of the guest had been added, and no other peaks could be reliably tracked.

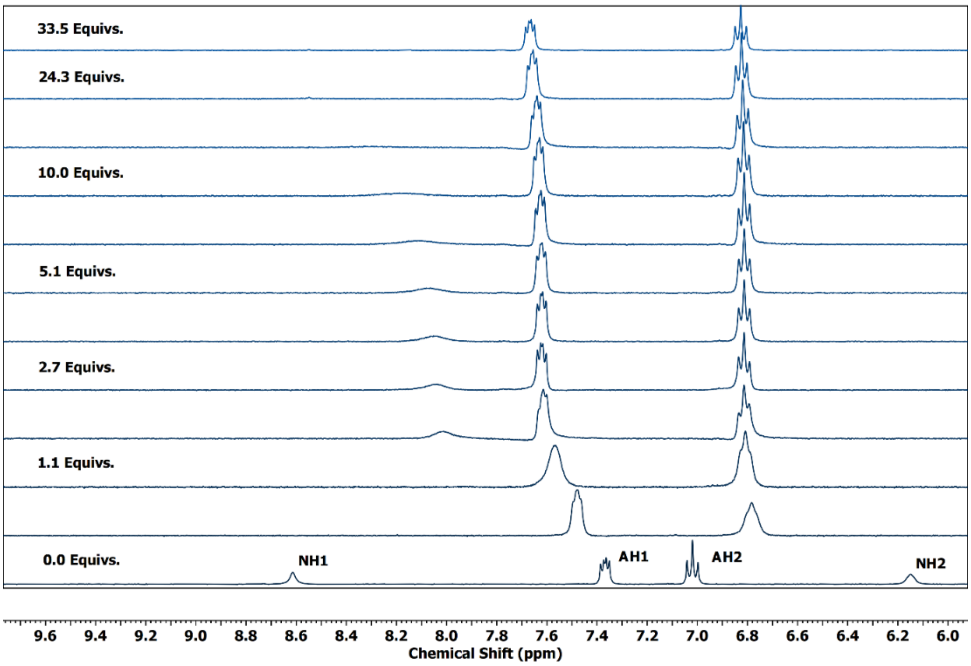

Figure S83. The ^{1}H-NMR stacked spectrum of transporter **3** (1 mM) in DMSO-d_6/0.5% H_2O at 298 K when titrated with $(TBA)_3HP_2O_7$ (0–33.5 equivs.) related to **Table 3**.

S5.5 Anion binding studies of transporter 4:

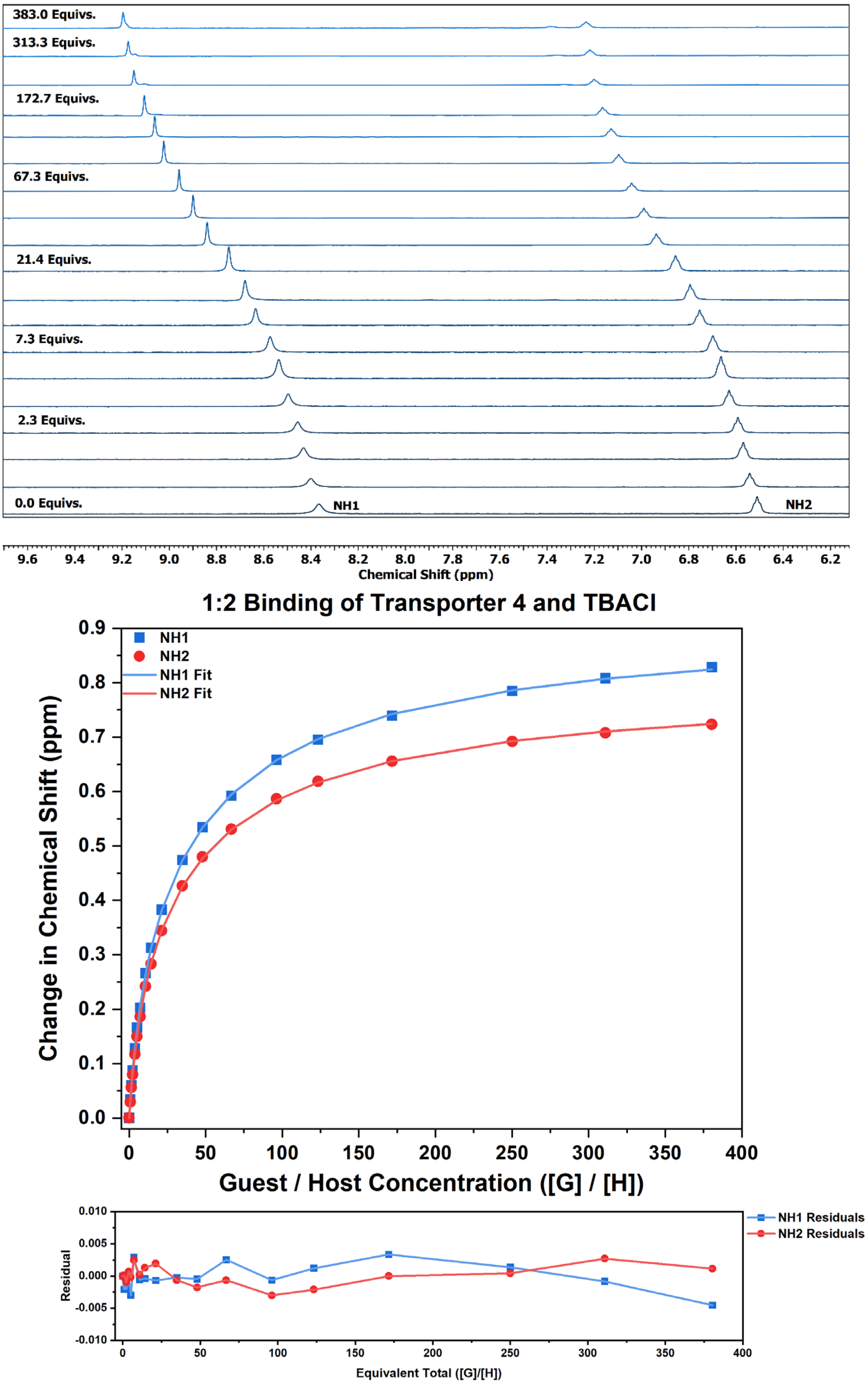

Figure S84. The ^{1}H-NMR stacked spectrum of transporter **4** (1 mM) in DMSO-d_6/0.5% H_2O at 298 K (top) when titrated with TBACl (0–383.0 equivs.), related to **Table 3**. The fitted binding data of **4** and the residual error obtained from fitting to the 1:2 binding model (bottom) K_{11} = 135 (± 1.35 %), K_{12} = 14.1 (± 0.95 %).

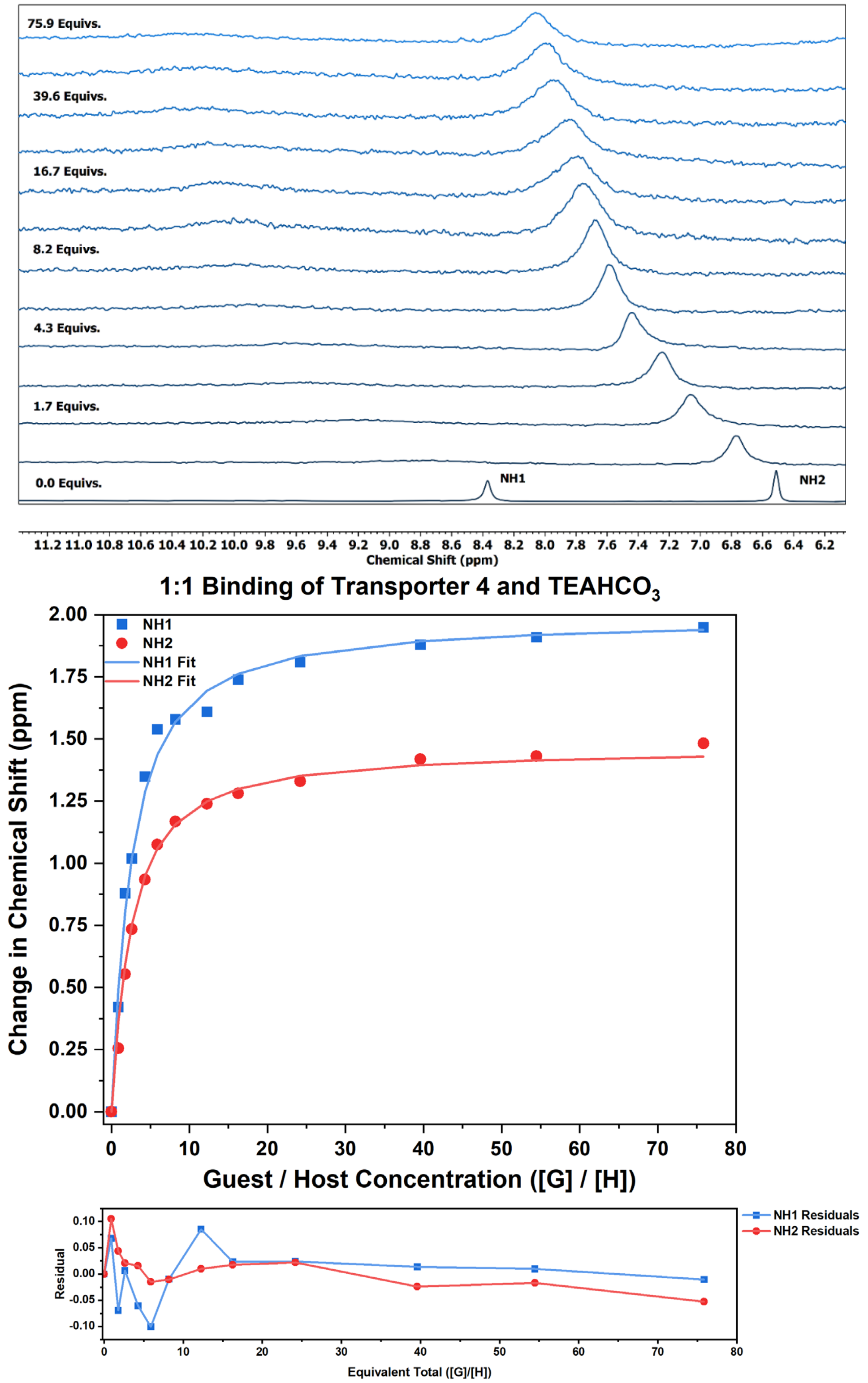

Figure S85. The ^{1}H-NMR stacked spectrum of transporter **4** (1 mM) in DMSO-d_6/0.5% H_2O at 298 K (top) when titrated with $TEAHCO_3$ (0–75.9 equivs.) related to **Table 3**.

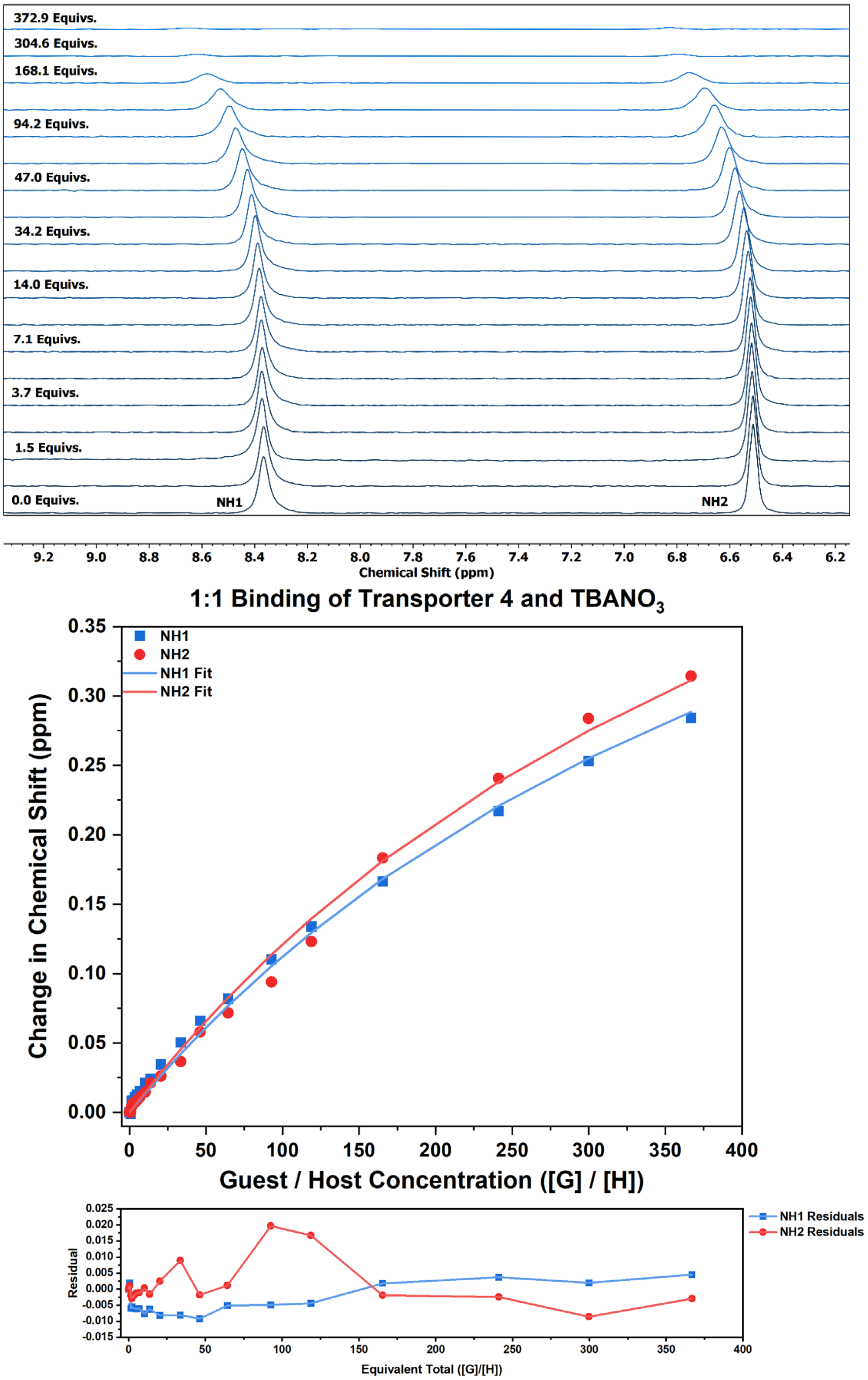

Figure S86. The ^{1}H-NMR stacked spectrum of transporter **4** (1 mM) in DMSO-d_6/0.5% H_2O at 298 K (top) when titrated with $TBANO_3$ (0–372.9 equivs.) related to **Table 3**. The fitted binding data of **4** and the residual error obtained from fitting to the 1:1 binding model (bottom) K_a = 2.40 (± 3.99 %).

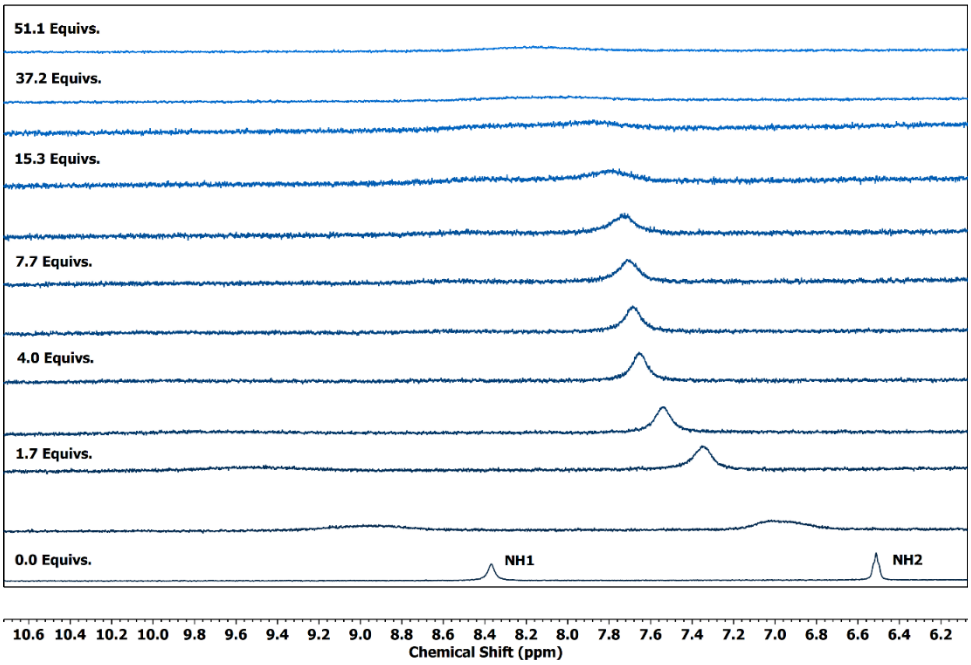

Figure S87. The ^{1}H-NMR stacked spectrum of transporter **4** (1 mM) in DMSO-d_6/0.5% H_2O at 298 K when titrated with $TBAH_2PO_4$ (0–51.1 equivs.), related to **Table 3**.

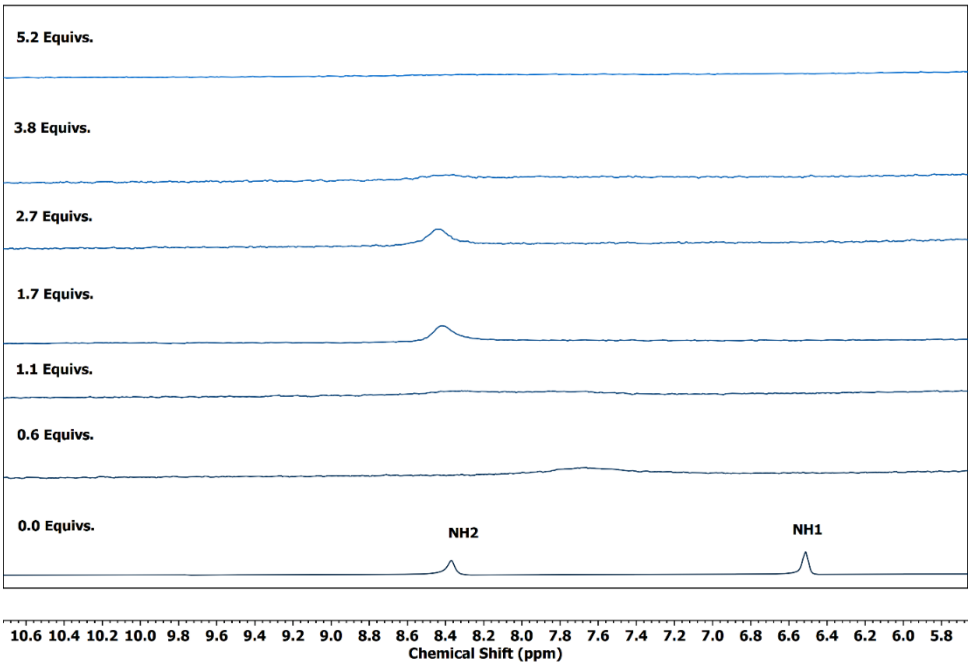

Figure S88. The ^{1}H-NMR stacked spectrum of transporter **4** (1 mM) in DMSO-d_6/0.5% H_2O at 298 K when titrated with $(TBA)_3HP_2O_7$ (0–5.2 equivs.), related to **Table 3**.

S5.6 Anion binding studies of transporter 5:

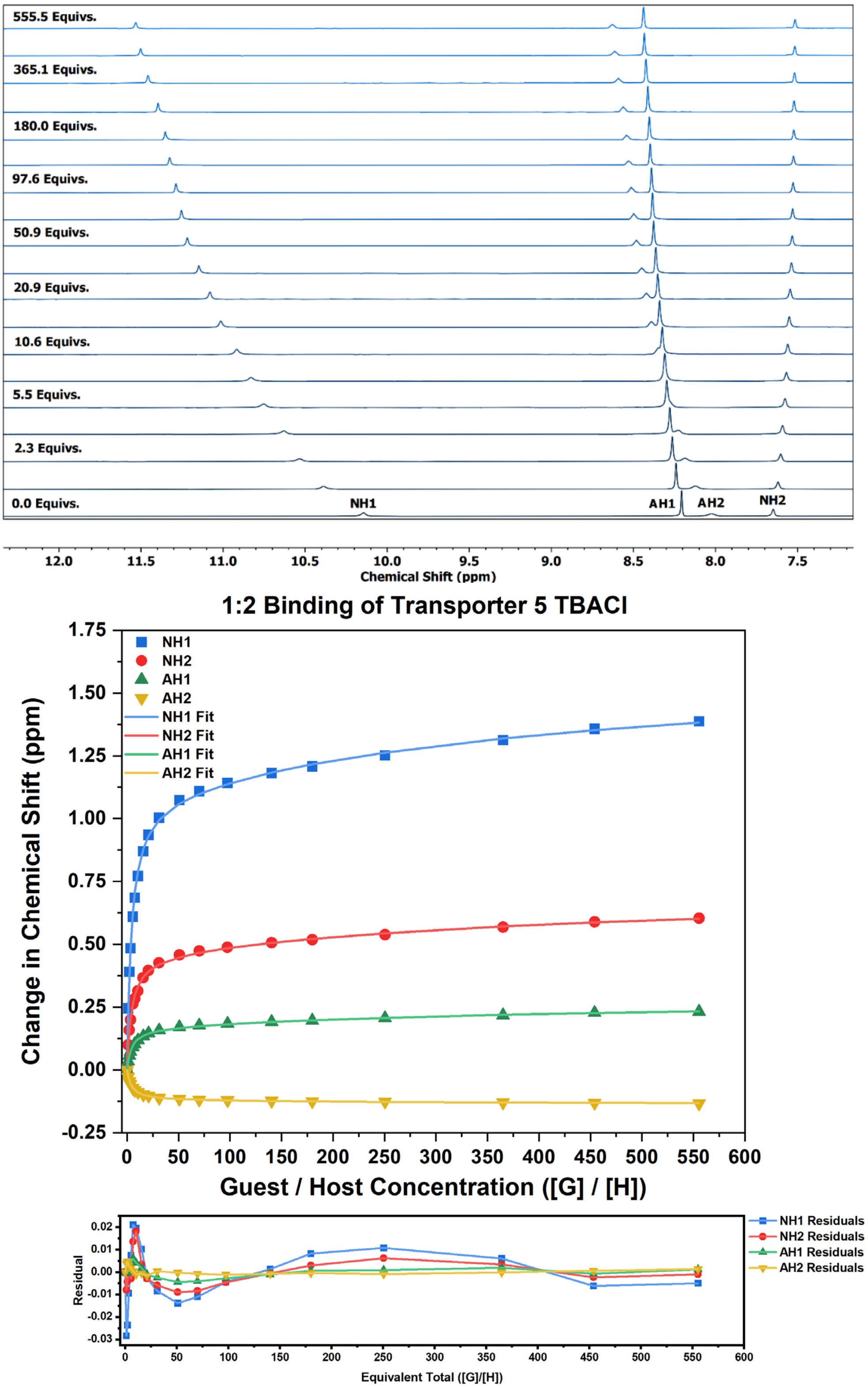

Figure S89. The ^{1}H-NMR stacked spectrum of transporter **5** (1 mM) in DMSO-d_6/0.5% H_2O at 298 K (top) when titrated with TBACl (0–555.5 equivs.), related to **Table 3**. The fitted binding data of **5** and the residual error obtained from fitting to the 1:2 binding model (bottom) K_{11} = 352 (± 2.73 %), K_{12} = 1.96 (± 4.49 %).

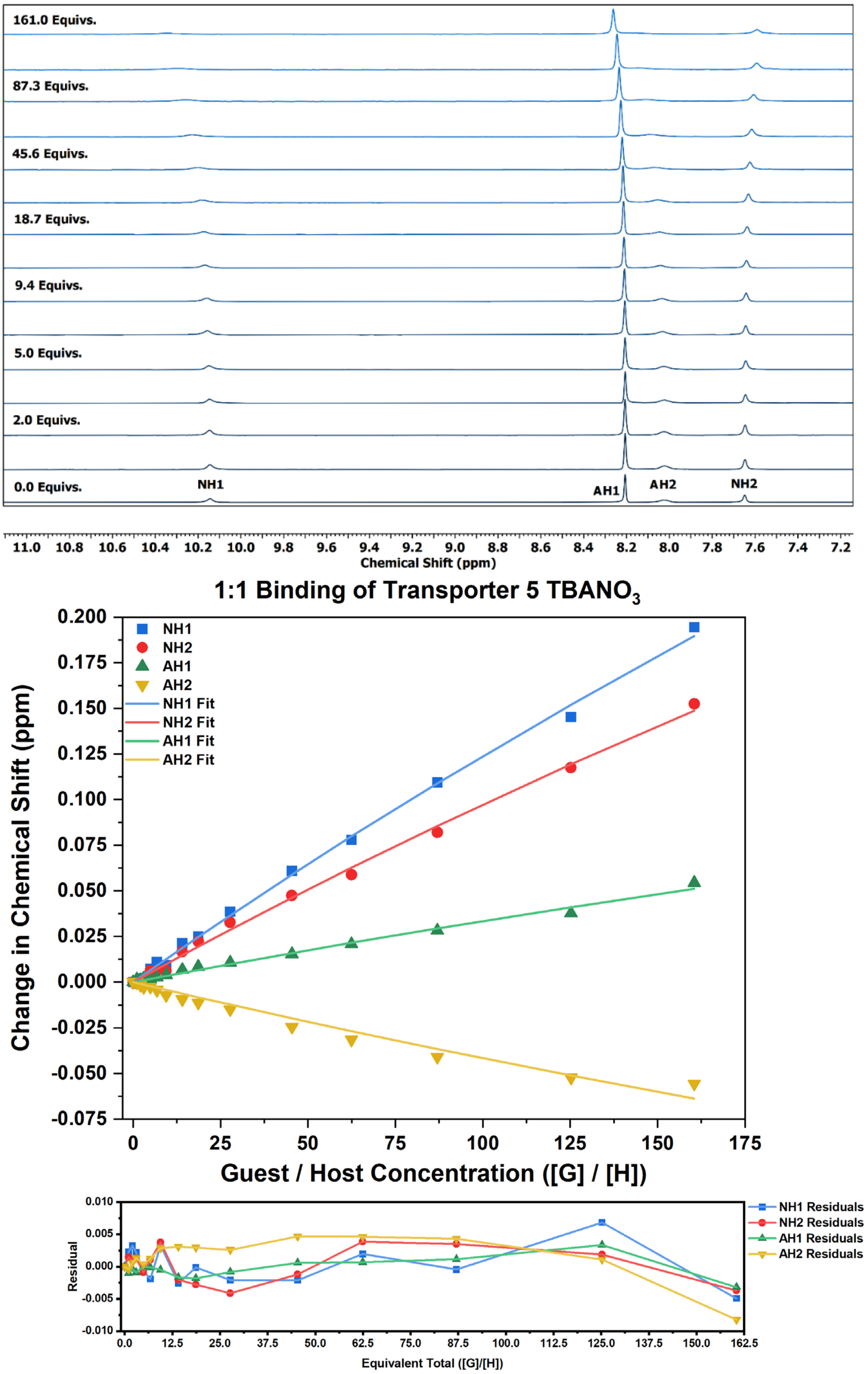

Figure S90. The ^{1}H-NMR stacked spectrum of transporter **5** (1 mM) in DMSO-d_6/0.5% H_2O at 298 K (top) when titrated with $TBANO_3$ (0–161.0 equivs.) related to **Table 3**. The fitted binding data of **5** and the residual error obtained from fitting to the 1:1 binding model (bottom) K_a = 1.51 (± 4.09 %).

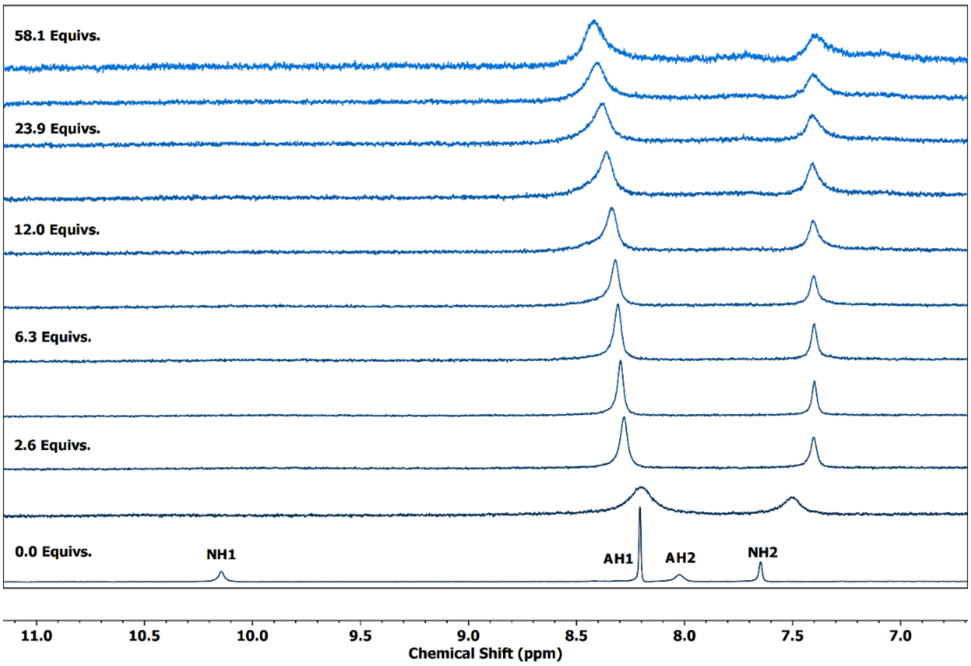

Figure S91. The ^{1}H-NMR stacked spectrum of transporter **5** (1 mM) in DMSO-d_6/0.5% H_2O at 298 K when titrated with $TEAHCO_3$ (0–58.1 equivs.) related to **Table 3**.

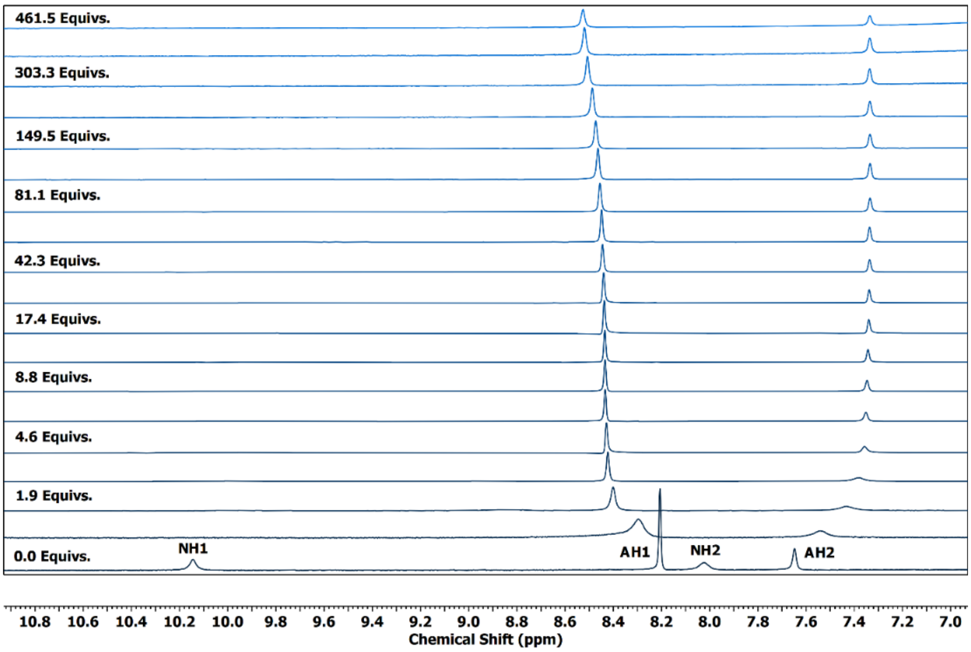

Figure S92. The ^{1}H-NMR stacked spectrum of transporter **5** (1 mM) in DMSO-d_6/0.5% H_2O at 298 K when titrated with $TBAH_2PO_4$ (0–461.5 equivs.) related to **Table 3**.

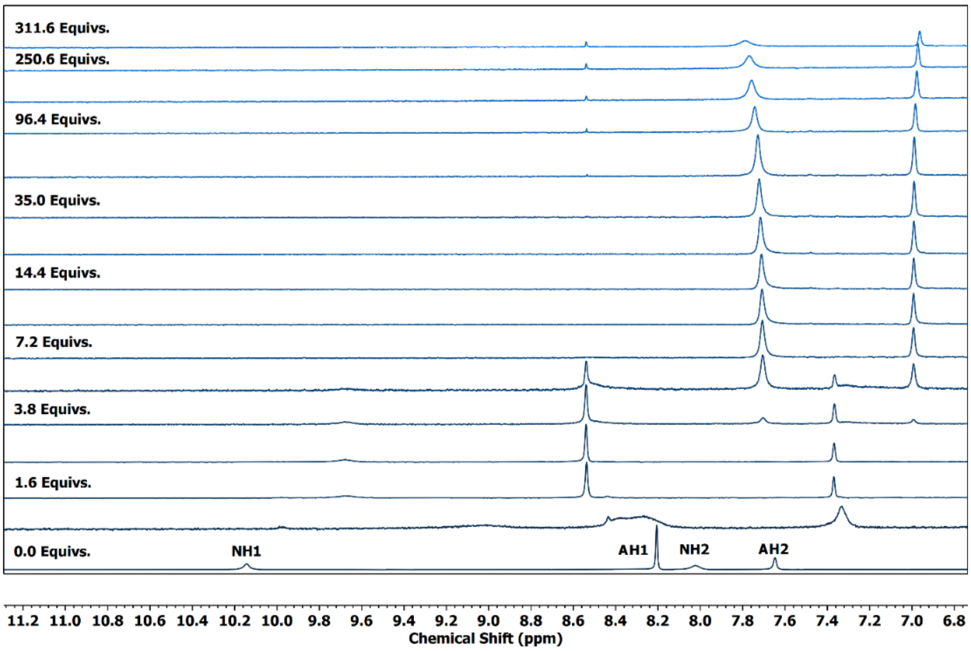

Figure S93. The ^{1}H-NMR stacked spectrum of transporter **5** (1 mM) in DMSO-d_6/0.5% H_2O at 298 K when titrated with $(TBA)_3HP_2O_7$ (0–311.6 equivs.), related to **Table 3**. Immediate deprotonation and complex binding were observed after the first addition of the guest, indicated by the broadened peaks and the emergence of new peaks. Also, significant peak shifts were observed after ~5 equivs. of the guest had been added.

S5.7 Anion binding studies of transporter 6:

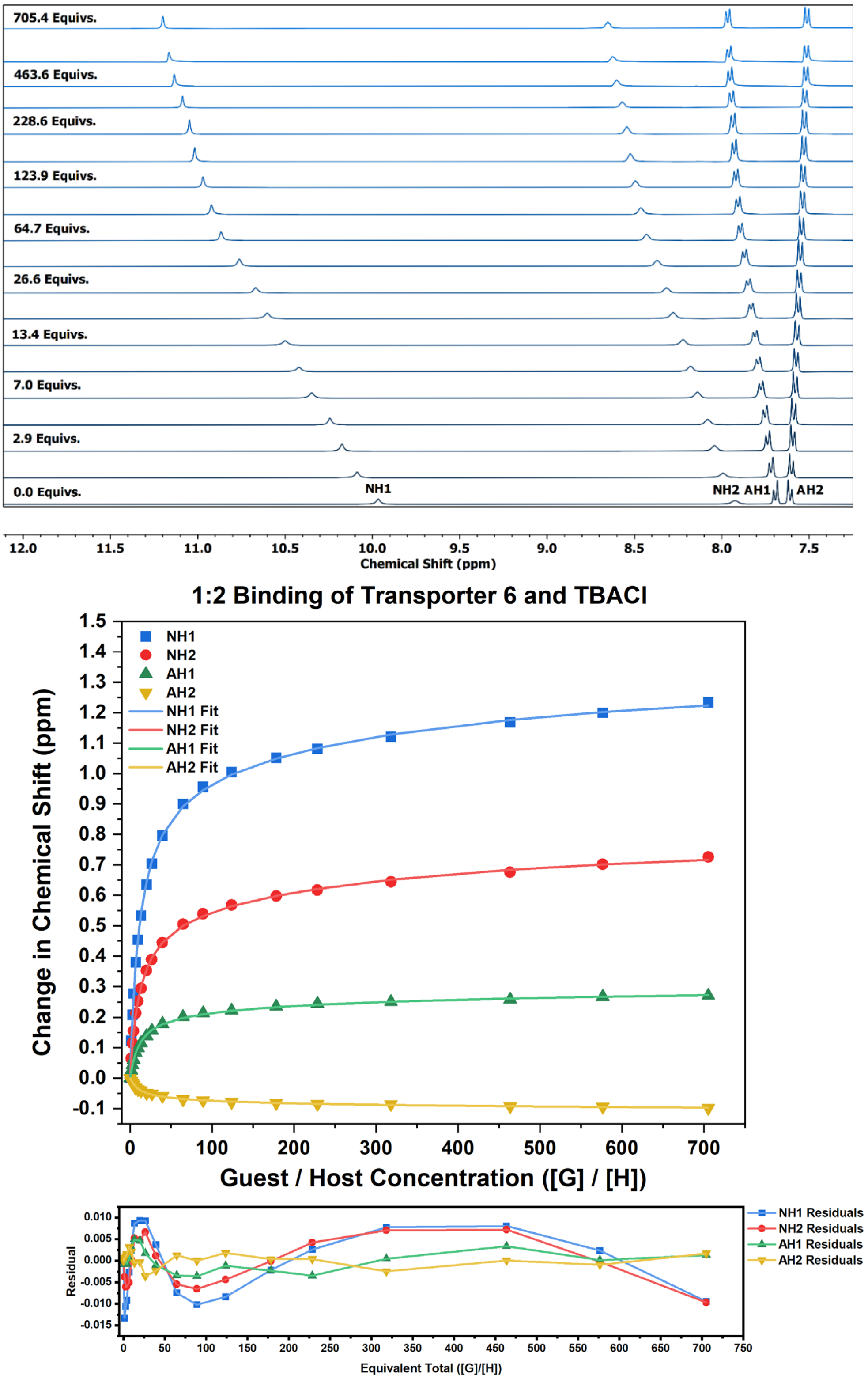

Figure S94. The ^{1}H-NMR stacked spectrum of transporter **6** (1 mM) in DMSO-d_6/0.5% H_2O at 298 K (top) when titrated with TBACl (0–705.4 equivs.), related to **Table 3**. The fitted binding data of **6** and the residual error obtained from fitting to the 1:2 binding model (bottom) K_{11} = 170 (± 2.56 %), K_{12} = 4.18 (± 5.03 %).

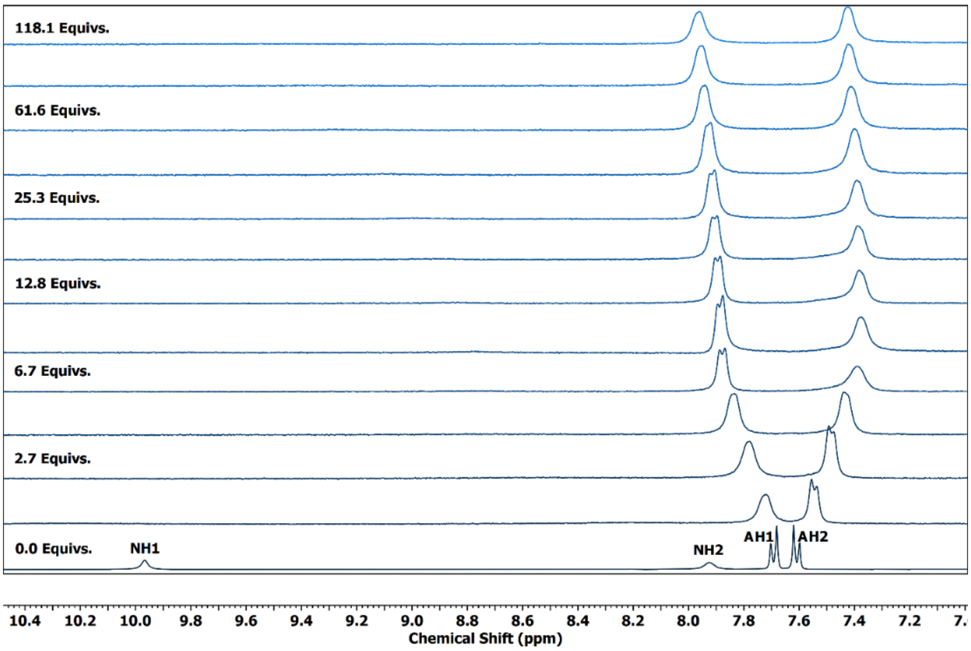

Figure S95. The ^{1}H-NMR stacked spectrum of transporter **6** (1 mM) in DMSO-d_6/0.5% H_2O at 298 K when titrated with $TBAHCO_3$ (0–118.1 equivs.), related to **Table 3**.

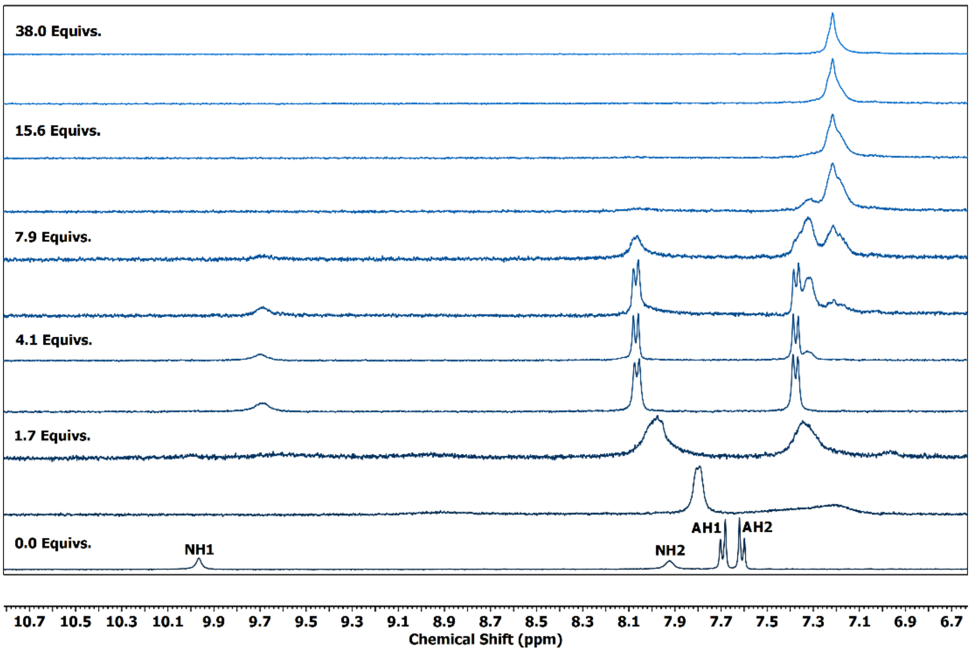

Figure S96. The ^{1}H-NMR stacked spectrum of transporter **6** (1 mM) in DMSO-d_6/0.5% H_2O at 298 K when titrated with $(TBA)_3HP_2O_7$ (0–38.0 equivs.), related to **Table 3**.

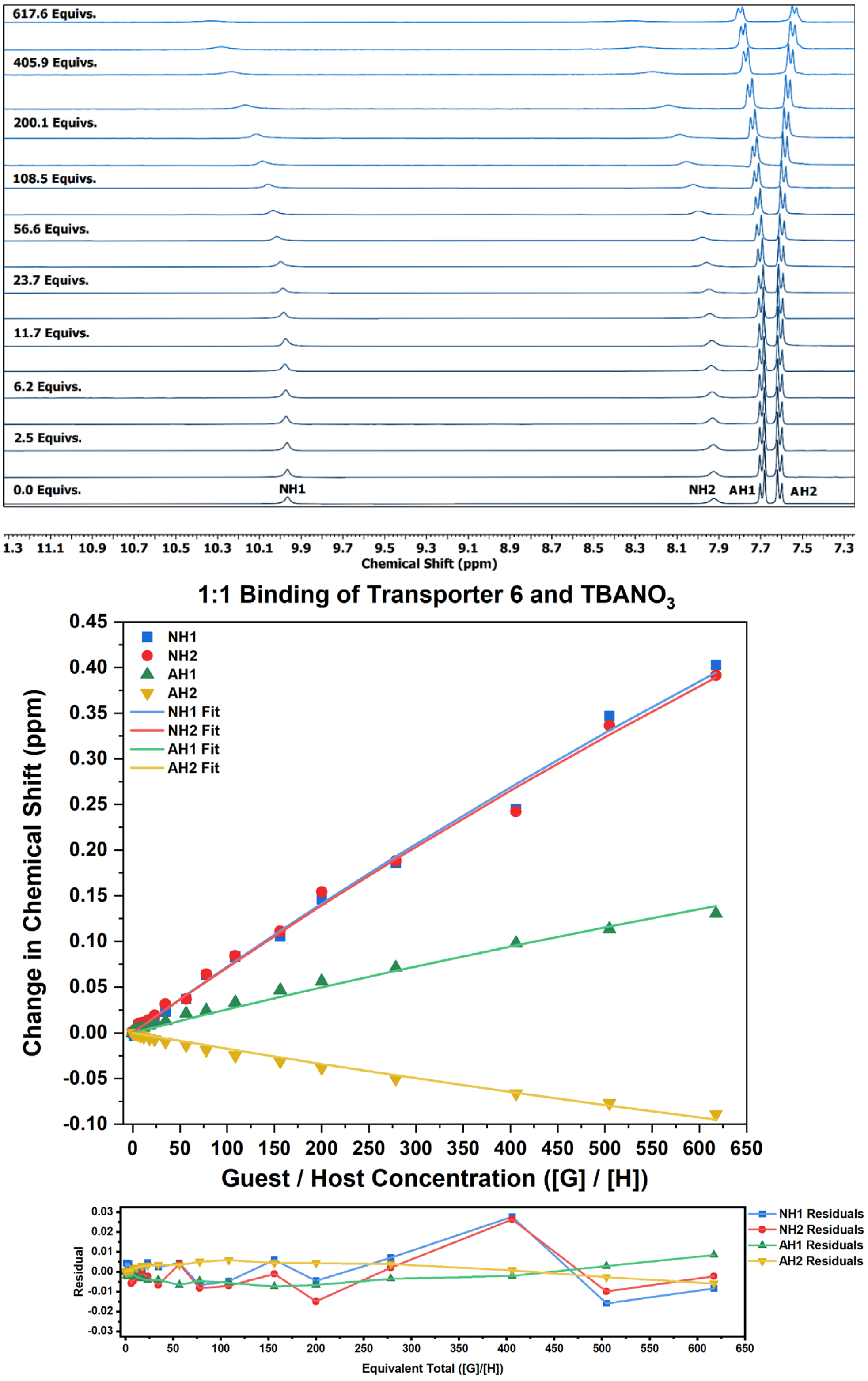

Figure S97. The ^{1}H-NMR stacked spectrum of transporter **6** (1 mM) in DMSO-d_6/0.5% H_2O at 298 K (top) when titrated with $TBANO_3$ (0–617.6 equivs.) related to **Table 3**. The fitted binding data of **6** and the residual error obtained from fitting to the 1:1 binding model (bottom) K_a = 1.64 (± 5.19 %).

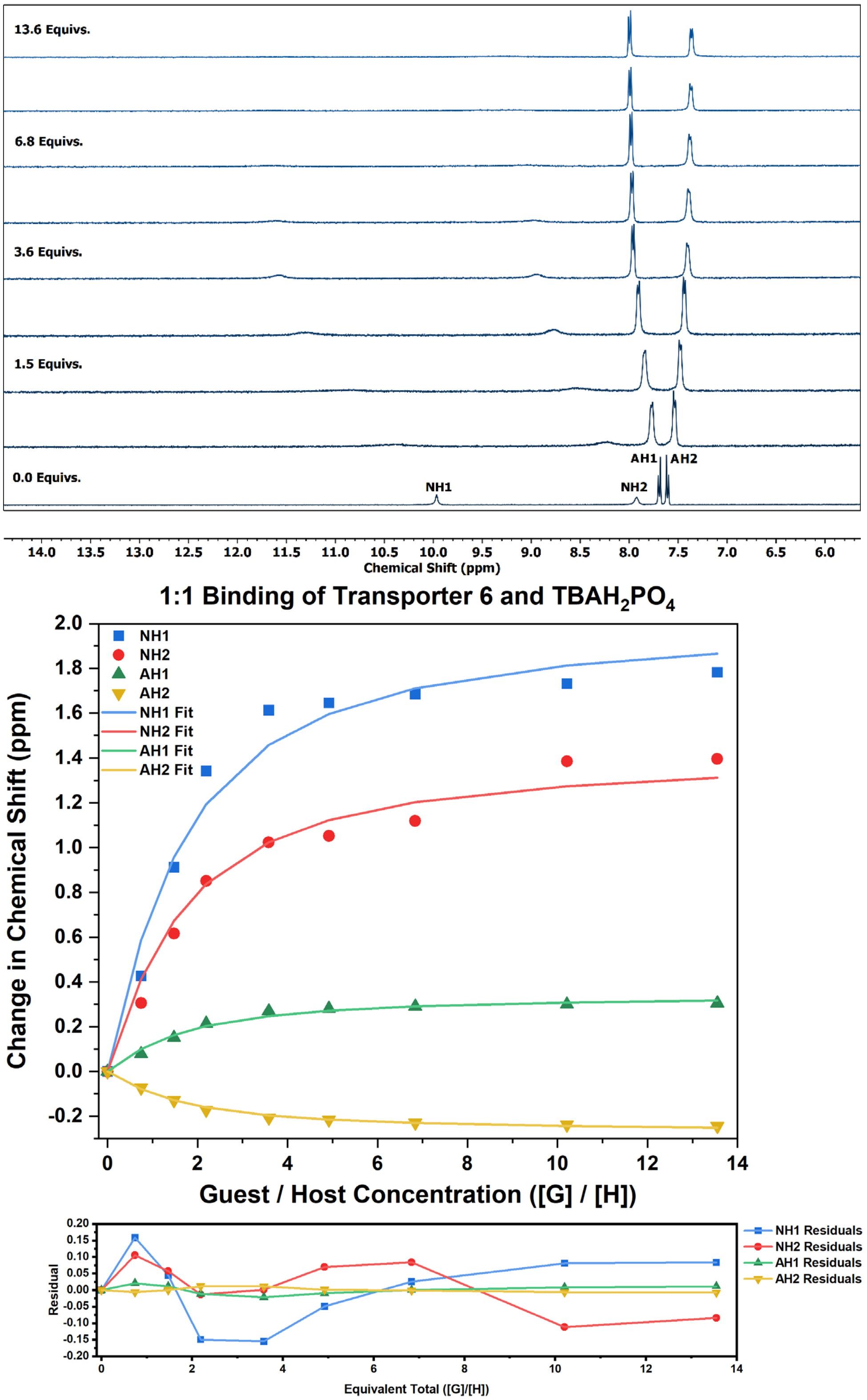

Figure S98. The ^{1}H-NMR stacked spectrum of transporter **6** (1 mM) in DMSO-d_6/0.5% H_2O at 298 K (top) when titrated with $TBAH_2PO_4$ (0–13.6 equivs.) related to **Table 3**.

S5.8 Anion binding studies of transporter 7:

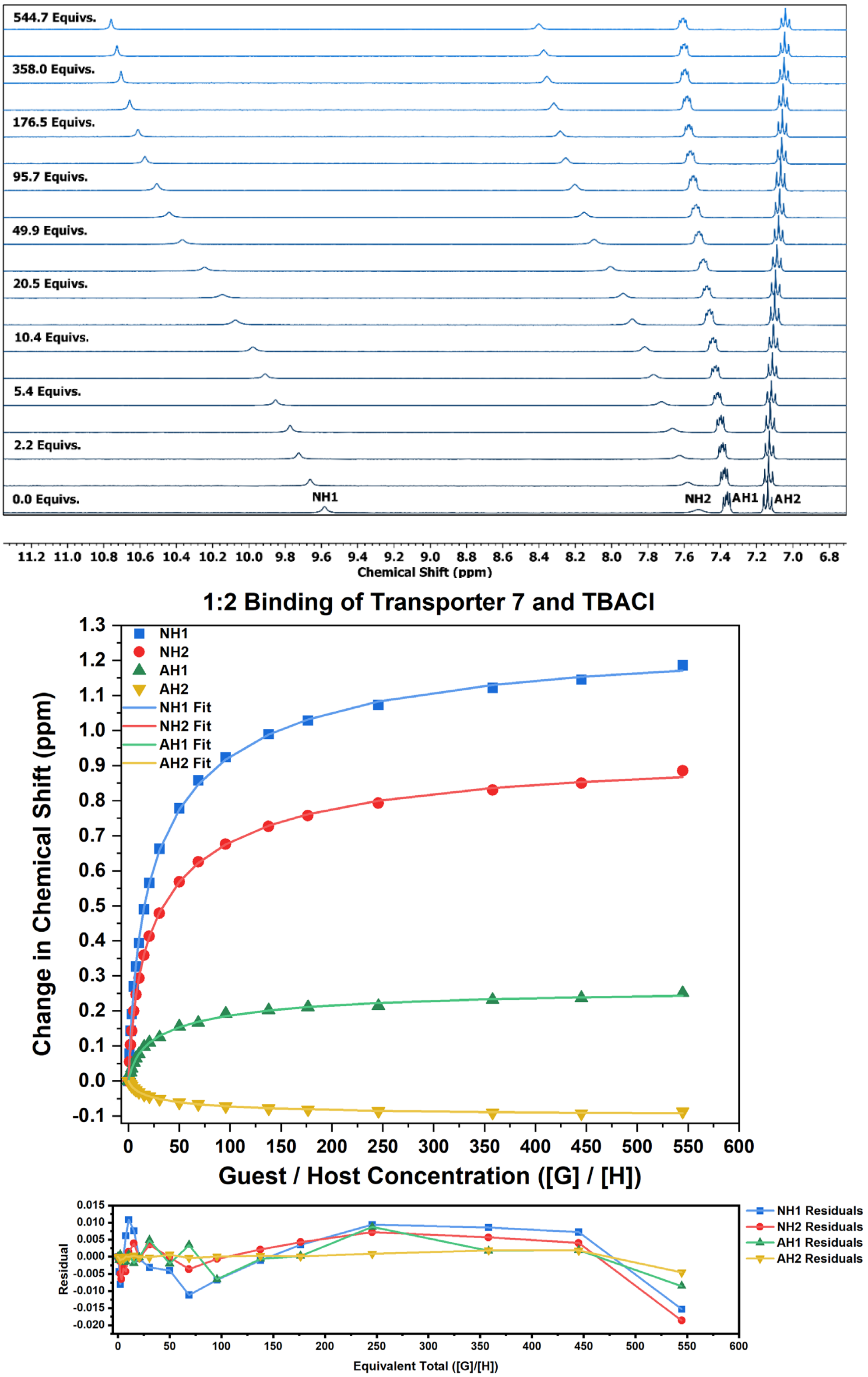

Figure S99. The ^{1}H-NMR stacked spectrum of transporter **7** (1 mM) in DMSO-d_6/0.5% H_2O at 298 K (top) when titrated with TBACl (0–544.7 equivs.), related to **Table 3**. The fitted binding data of **7** and the residual error obtained from fitting to the 1:2 binding model (bottom) K_{11} = 160 (± 2.52 %), K_{12} = 13.4 (± 2.12 %).

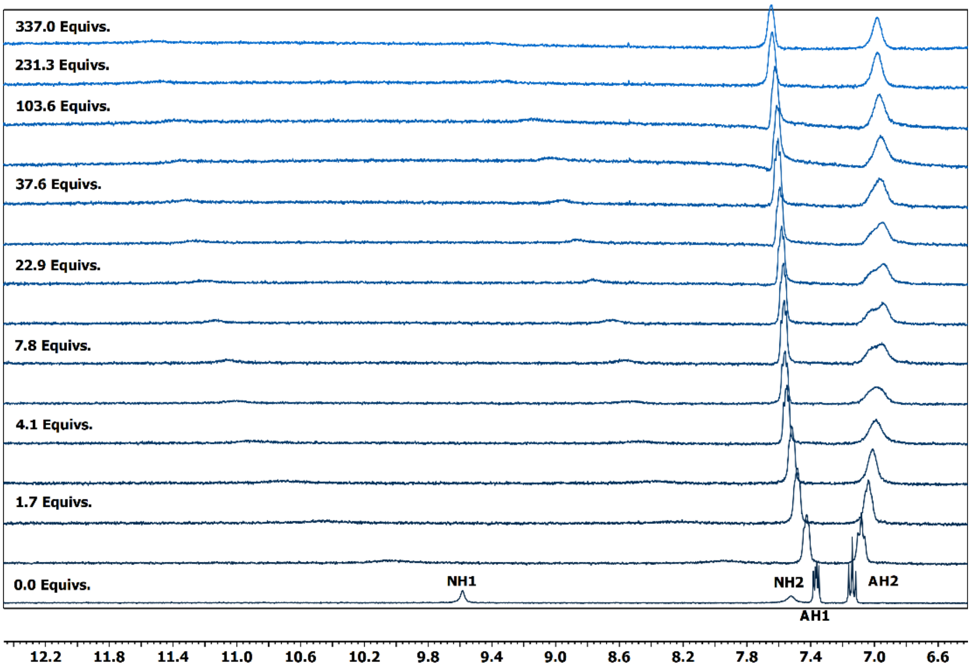

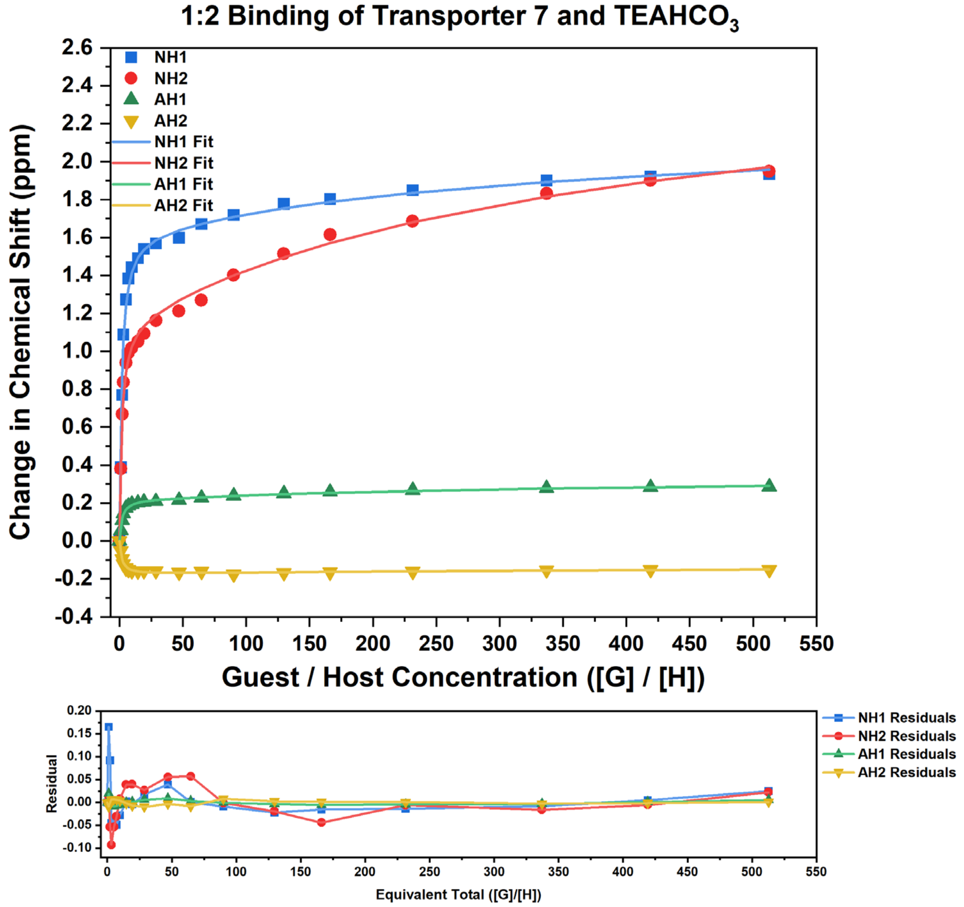

Figure S100. The ^{1}H-NMR stacked spectrum of transporter **7** (1 mM) in DMSO-d_6/0.5% H_2O at 298 K (top) when titrated with $TEAHCO_3$ (0–337.0 equivs.), related to **Table 3**. Note that due to minimal peak shifts and very broad NH proton peaks, spectra acquired after the addition of 337.0 equivs. of the guest have been removed for clarity in the stacked spectrum (top).

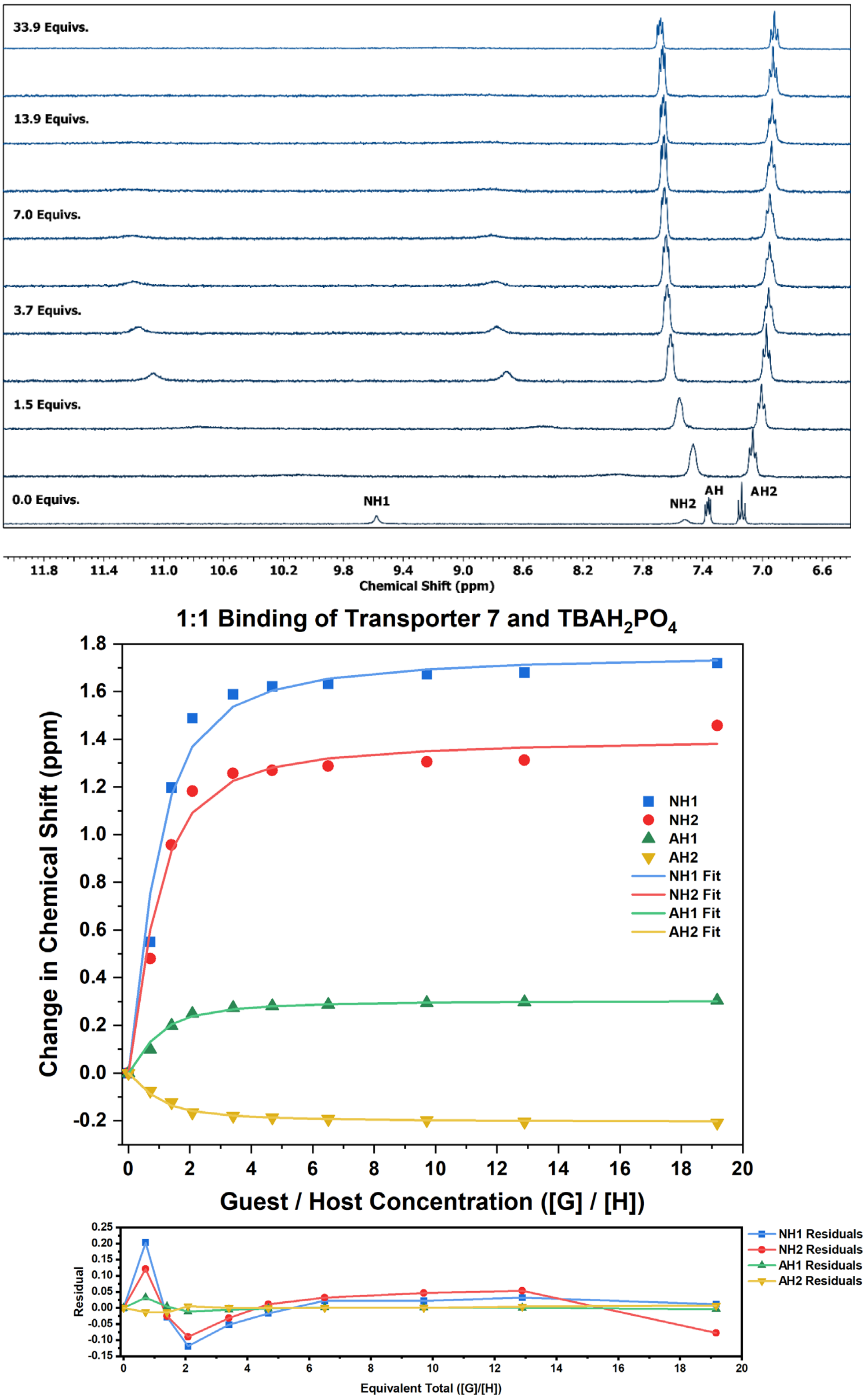

Figure S101. The ^{1}H-NMR stacked spectrum of transporter **7** (1 mM) in DMSO-d_6/0.5% H_2O at 298 K (top) when titrated with $TBAH_2PO_4$ (0–33.9 equivs.), related to **Table 3**. After 19.1 equivs. of the guest had been added, the NH proton peaks could no longer be tracked.

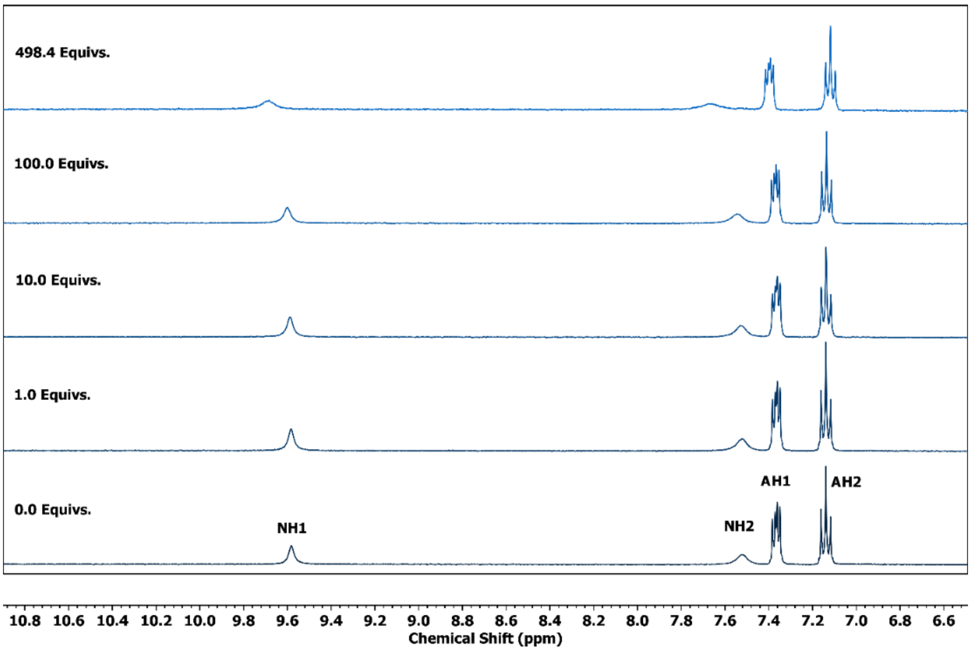

Figure S102. The ^{1}H-NMR stacked spectrum of transporter **7** (1 mM) in DMSO-d_6/0.5% H_2O at 298 K when titrated with $TBANO_3$ (0–498.4 equivs.), related to **Table 3**.

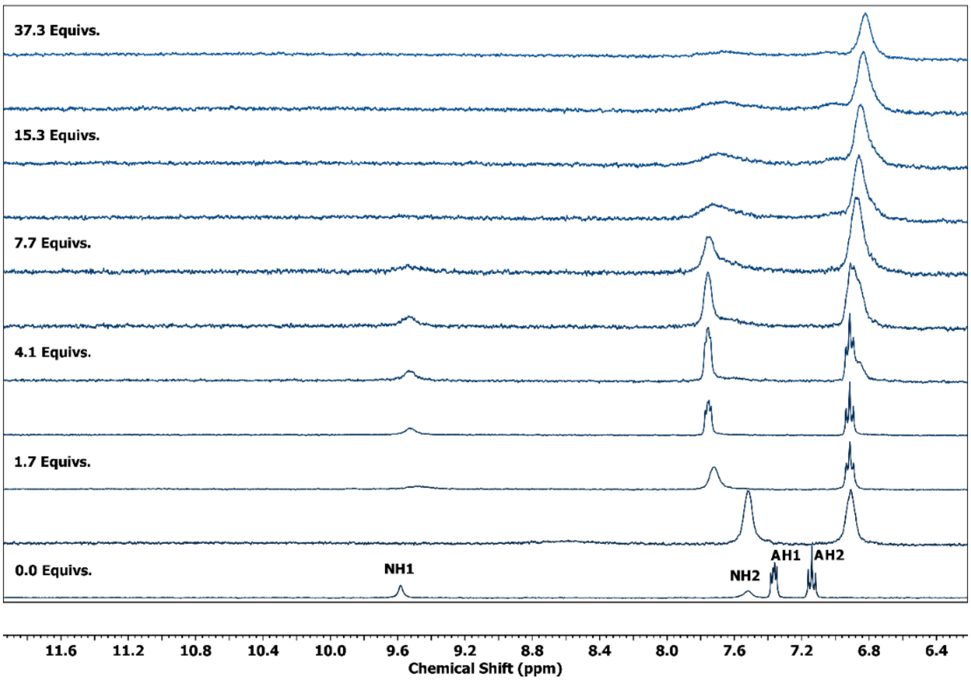

Figure S103. The ^{1}H-NMR stacked spectrum of transporter **7** (1 mM) in DMSO-d_6/0.5% H_2O at 298 K when titrated with $(TBA)_3HP_2O_7$ (0–37.3 equivs.) related to **Table 3**.

S5.9 Anion binding studies of transporter 8:

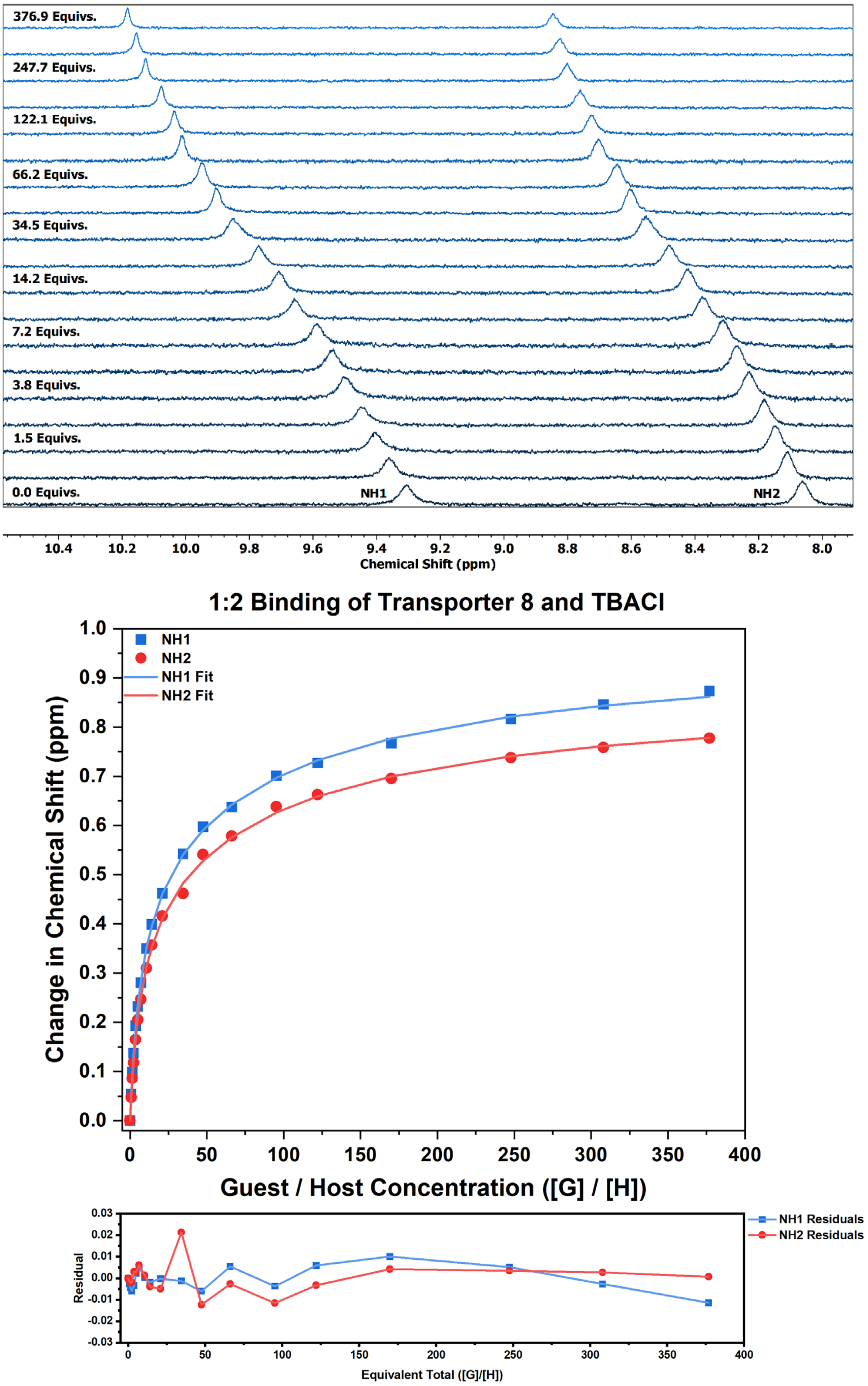

Figure S104. The ^{1}H-NMR stacked spectrum of transporter **8** (1 mM) in DMSO-d_6/0.5% H_2O at 298 K (top) when titrated with TBACl (0–376.9 equivs.). The fitted binding data of **8** and the residual error obtained from fitting to the 1:2 binding model (bottom) K_{11} = 155 (± 4.26 %), K_{12} = 9.20 (± 4.11 %) related to **Table 3**.

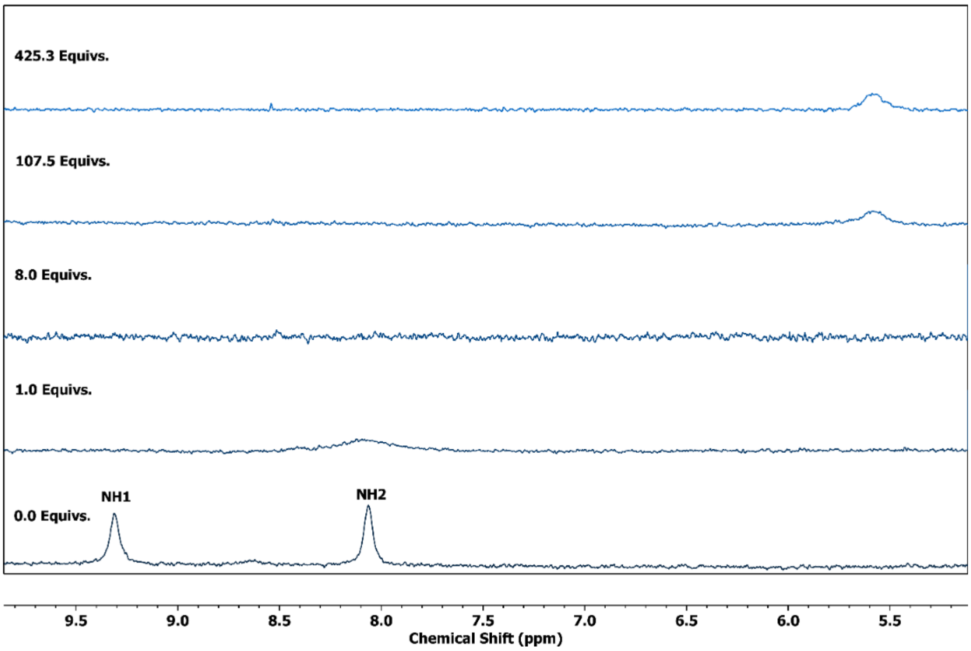

Figure S105. The ^{1}H-NMR stacked spectrum of transporter **8** (1 mM) in DMSO-d_6/0.5% H_2O at 298 K when titrated with $TBAHCO_3$ (0–425.3 equivs.), related to **Table 3**.

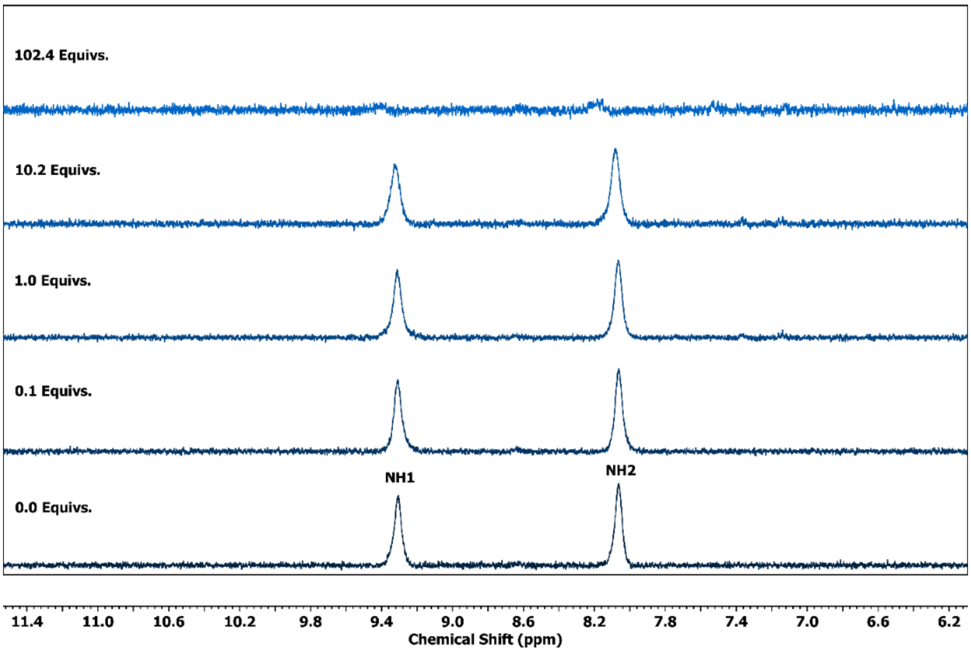

Figure S106. The ^{1}H-NMR stacked spectrum of transport**er 8** (1 mM) in DMSO-d_6/0.5% H_2O at 298 K when titrated with $TBANO_3$ (0–102.4 equivs.), related to **Table 3**.

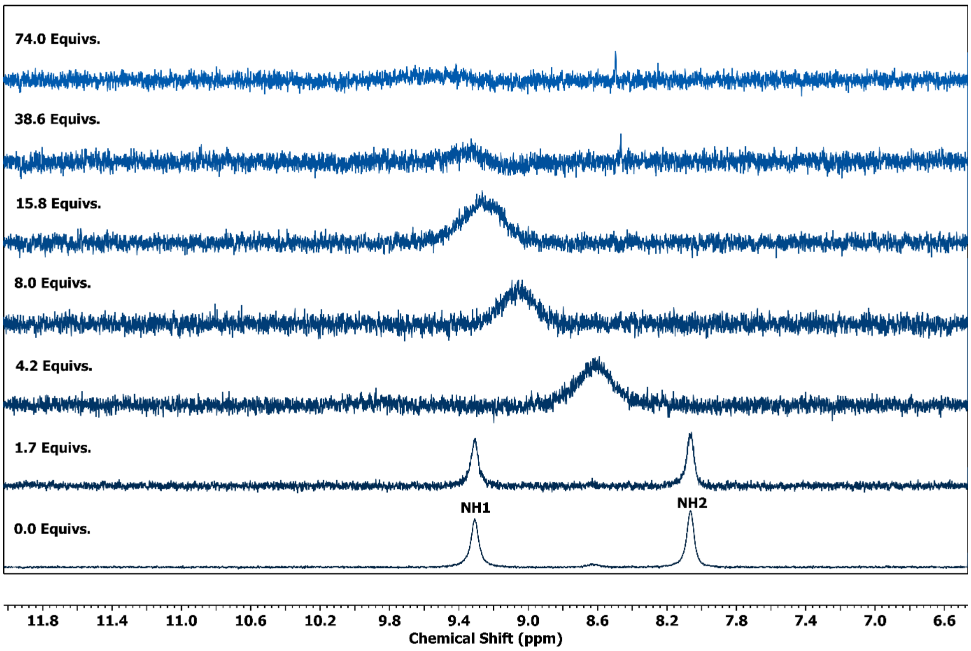

Figure S107. The ^{1}H-NMR stacked spectrum of transporter **8** (1 mM) in DMSO-d_6/0.5% H_2O at 298 K when titrated with $TBAH_2PO_4$ (0–74.0 equivs.), related to **Table 3**.

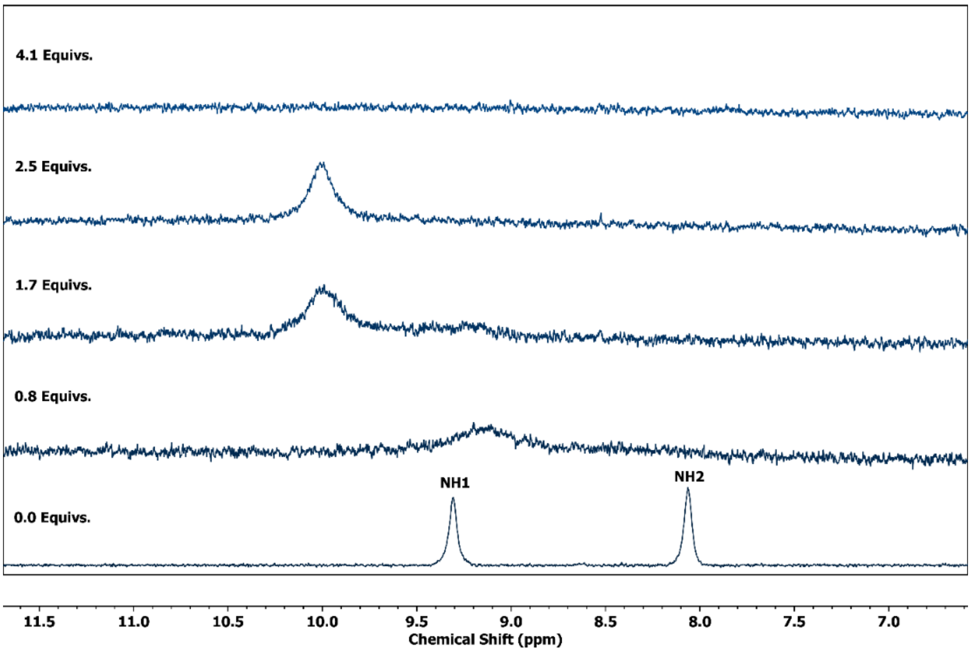

Figure S108. The ^{1}H-NMR stacked spectrum of transporter **8** (1 mM) in DMSO-d_6/0.5% H_2O at 298 K when titrated with $(TBA)_3HP_2O_7$ (0–4.1 equivs.), related to **Table 3**.

S6. Single crystal X-Ray diffraction:

Table S2. Selected Crystallographic Information for **8** and **8·2NO$_3$** related to **Figure 3** and the Star Methods Section.

Transporter	8	8·2NO$_3$
Formula	$C_{38}H_{28}F_{20}N_{10}S_4$	$C_{38}H_{28}F_{20}N_{10}S_4\cdot2[NO_3^-]$
CCDC Deposition №.	2201398	2201397
Molecular Mass (gmol^{-1})	1132.92	1256.93
Crystal Size (mm)	0.14 × 0.24 × 0.36	0.21 × 0.30 × 0.48
Temperature (K)	150	150(1)
Wavelength (Å)	0.7107	1.54184
Crystal System	Triclinic	Triclinic
Space Group	P $\bar{1}$	P $\bar{1}$
a (Å)	8.4103 (4)	13.5201 (4)
b (Å)	11.5621 (5)	13.5649 (4)
c (Å)	11.8311 (6)	14.5206 (4)
α (°)	101.535 (4)	109.335 (3)
β (°)	101.482 (4)	117.651 (3)
γ (°)	92.384 (4)	97.173 (3)
Volume (Å^3)	110.69 (9)	2098.65 (16)
Z	4	4
Refinement Method	Full-matrix least-squares on F^2	Full-matrix least-squares on F^2
№. of Reflections Collected	8135	20916
№. Unique	5071	7998
R_{int}	0.025	0.062
Completeness (%)	98.9	94.7
№. Reflections Used	5071	7998
ρ_c (gcm^{-1})	1.703	1.378
F(000)	566.0	910.0
μ (mm^{-1})	0.35	1.93
**IAM Refinement**		
Final $R1_{all}$, $wR2_{all}$	4.43, 10.79	6.94, 20.91
Goodness of Fit	1.031	1.046
Residual Density (*e* Å^{-3})	0.41, 0.33	−0.54, 0.44

Table S3. Crystal data and structure refinement for **8** and **8·2NO$_3$**, related to **Figure 3** and the Star Methods Section.

Structure	8	8·2NO$_3$
Empirical Formula	$C_{4.75}H_{3.5}F_{2.5}N_{1.25}S_{0.5}$	$C_{11.67}H_{16.67}F_{3.33}N_{2.33}OS_{0.67}$
Formula Weight (gmol^{-1})	141.62	290.31
Temperature (K)	150.00(10)	150.0(4)
Crystal System	Triclinic	Triclinic
Space Group	P $\bar{1}$	P $\bar{1}$
a (Å)	8.4103(4)	13.5201(4)
b (Å)	11.5621(5)	13.5649(4)
c (Å)	11.8311(6)	14.5206(4)
α (°)	101.535(4)	109.335(3)
β (°)	101.482(4)	117.651(3)
γ (°)	92.384(4)	97.173(3)
Volume (Å^3)	1100.69(9)	2098.64(13)
Z	8	6
ρ_{calc} (gcm^{-3})	1.709	1.378
μ (mm^{-1})	0.346	1.927
F(000)	570.0	910.0
Crystal Size (mm^{-3})	0.363 × 0.239 × 0.136	0.48 × 0.3 × 0.21
Radiation	Mo Kα (λ = 0.71073)	Cu Kα (λ = 1.54184)
2Θ Range For Data Collection (°)	5.476 to 59.116	7.326 to 148.922
Index Ranges	−10 ≤ h ≤ 11, −12 ≤ k ≤ 15, −15 ≤ l ≤ 12,	−16 ≤ h ≤ 16, −16 ≤ k ≤ 16, −18 ≤ l ≤ 18
№. of Reflections Collected	8135	20916
№. of Independent Reflections	5071 [R_{int} = 0.0250, R_{sigma} = 0.0484]	7998 [R_{int} = 0.0622, R_{sigma} = 0.0470]
Data/Restraints/Parameters	5071/0/325	7998/0/518
Goodness-of-fit on F^2	1.031	1.046
Final R indexes [I>2α (I)]	R_1 = 0.0443, wR_2 = 0.0961	R_1 = 0.0694, wR_2 = 0.1951
Final R Indexes [all data]	R_1 = 0.0592, wR_2 = 0.1079	R_1 = 0.0770, wR_2 = 0.2091
Largest diff. peak/hole (*e* Å^{-3})	0.42/−0.33	0.44/−0.54

Table S4. Fractional Atomic Coordinates (×10^4) and Equivalent Isotropic Displacement Parameters (Å^2×10^3) for **8**. U_{eq} is defined as 1/3 of the trace of the orthogonalized U_{IJ} tensor, related to **Figure 3, A** and the Star Methods Section.

Atom	x	y	z	U(eq)
S1	322.6(6)	3715.1(5)	1040.3(4)	27.17(14)
S2	6277.3(7)	5128.8(5)	4208.8(4)	28.08(14)
F6	1275.8(18)	2379.0(12)	4278.8(11)	40.8(3)
F1	8541.4(18)	3402.4(12)	5397.7(11)	40.6(3)
F10	2971.4(18)	1715.9(13)	655.3(12)	41.9(3)
F5	6512.3(19)	1900.4(13)	1331.8(11)	45.6(4)
F8	291.5(19)	−1374.6(12)	1760.1(14)	48.2(4)
F7	78.2(19)	100.0(13)	3814.5(13)	45.9(4)
F9	1755(2)	−556.7(13)	195.9(12)	47.4(4)
F2	7360(2)	1406.7(14)	5868.3(14)	54.8(4)
F4	5455(2)	−153.2(13)	1801.3(15)	60.3(5)
F3	5917(2)	−421.7(14)	4078.2(18)	67.2(5)
N3	5142(2)	6211.3(15)	1340.2(14)	25.4(4)
N4	2852(2)	5166.0(15)	2360.5(15)	27.3(4)
N2	8214(2)	5617.9(16)	2789.9(15)	28.4(4)
N1	8132(2)	3731.7(16)	3040.0(16)	30.1(4)
N5	2754(2)	3312.7(16)	2710.6(16)	32.9(5)
C7	7594(2)	4836.4(19)	3305.3(17)	25.4(4)
C16	1426(3)	1668.2(19)	3267.0(18)	28.4(5)
C13	2062(2)	4105.7(18)	2083.2(17)	23.2(4)
C6	7514(3)	2719(2)	3344.8(19)	28.5(5)
C15	2155(3)	2118.5(19)	2476.7(18)	27.3(5)
C9	6727(3)	6892.2(19)	1602.0(18)	28.2(5)
C5	7717(3)	2566(2)	4498.2(19)	30.9(5)
C12	2386(3)	6154.0(19)	1803(2)	29.9(5)
C20	2261(3)	1338(2)	1446.0(19)	29.7(5)
C19	1646(3)	172(2)	1204.3(19)	32.1(5)
C8	7736(3)	6811.5(19)	2796.3(19)	30.0(5)
C11	3906(3)	6911.8(19)	1785.0(19)	28.6(5)
C10	4637(3)	5589.9(18)	101.0(17)	29.6(5)
C17	804(3)	503(2)	3040(2)	31.9(5)
C18	905(3)	−245.9(19)	1998(2)	32.4(5)
C1	6734(3)	1783(2)	2456(2)	34.1(5)
C4	7139(3)	1532(2)	4742(2)	38.6(6)
C2	6189(3)	738(2)	2693(2)	42.3(6)
C3	6394(3)	611(2)	3841(3)	44.2(6)

Table S5. Anisotropic Displacement Parameters (Å^2×10^3) for **8**. The Anisotropic Displacement Factor Exponent Takes the Form: $-2\pi^2[h^2a^{*2}U_{11}+2hka^*b^*U_{12}+\ldots]$, related to **Figure 3**, **A** and the Star Methods Section.

Atom	U_{11}	U_{22}	U_{33}	U_{23}	U_{13}	U_{12}
S1	23.4(3)	31.8(3)	25.0(3)	7.9(2)	0.9(2)	−1.2(2)
S2	26.3(3)	33.6(3)	23.4(3)	4.0(2)	6.1(2)	−1.3(2)
F6	50.7(9)	41.0(8)	29.2(7)	3.6(6)	8.3(6)	6.5(7)
F1	42.3(8)	45.9(9)	31.1(7)	9.0(6)	1.4(6)	1.4(7)
F10	47.0(10)	46.1(9)	40.5(7)	18.3(6)	19.1(7)	1.8(7)
F5	49.3(9)	50.0(9)	32.5(7)	−0.5(6)	5.5(7)	7.1(7)
F8	53.2(10)	25.7(8)	64.5(10)	10.3(7)	10.7(8)	−5.5(7)
F7	50.6(9)	46.6(9)	53.8(9)	26.0(7)	26.5(7)	6.9(7)
F9	60.8(10)	39. 2(8)	38.6(8)	−1.8(6)	12.0(7)	6.6(7)
F2	69.7(11)	57.7(10)	54.4(9)	33.0(8)	29.5(8)	20.9(9)
F4	54.7(10)	33.8(9)	80.2(12)	−12.3(8)	12.3(9)	−5.0(7)
F3	78.5(13)	32.3(9)	107.4(14)	22.7(9)	50.8(11)	3.7(8)
N3	28.4(9)	22.0(9)	24.4(9)	2.1(7)	5.7(7)	0.8(7)
N4	25.6(9)	26.1(10)	27. 3(9)	6.3(7)	−1.3(8)	−0.4(7)
N2	23.7(9)	31.3(10)	31.6(9)	8.2(8)	7.9(8)	−0.3(7)
N1	27.6(10)	33.1(11)	34.6(10)	10.1(8)	15.4(8)	2.6(8)
N5	32.5(10)	28.5(10)	31.8(10)	10.4(8)	−9.6(8)	−5.1(8)
C7	20.0(10)	31.6(12)	22.0(10)	4.3(8)	1.0(8)	−2.1(8)
C16	30.5(11)	31.3(12)	22.1(10)	5.9(8)	1.9(9)	5.2(9)
C13	23.4(10)	26.4(11)	21.0(9)	5.9(8)	6.6(8)	1.6(8)
C6	24.2(11)	30.0(12)	33.0(11)	7.4(9)	9.3(9)	3.4(9)
C15	25.2(11)	26.4(11)	28.3(11)	9.8(8)	−2.6(9)	0.7(9)
C9	34.3(12)	21.7(11)	31.0(11)	6.4(8)	12.4(9)	−2.3(9)
C5	27.5(11)	32.1(13)	34.5(12)	8.5(9)	7.5(10)	4.9(9)
C12	28.7(11)	24.5(12)	36.8(12)	9.0(9)	5.2(10)	3.8(9)
C20	28.8(11)	33.5(13)	29.7(11)	14.0(9)	5.8(9)	2.3(9)
C19	34.2(12)	31.7(13)	28.8(11)	3.4(9)	4.8(10)	6.9(0)
C8	29.0(11)	27.0(12)	32.6(11)	5.1(9)	6.7(9)	−7.1(9)
C11	31.4(12)	23.2(11)	31.5(11)	5.1(8)	8.2(9)	2.4(9)
C10	38.3(13)	26.8(12)	22.1(10)	4.6(8)	2.5(9)	5.4(10)
C17	29.7(12)	35.7(13)	35.8(12)	17.7(10)	8.9(10)	5.2(10)
C18	32.2(12)	22.9(12)	41.8(13)	10.0(9)	4.1(10)	−0.1(9)
C1	28.7(12)	36.5(14)	36.0(12)	4.3(10)	7.0(10)	5.3(10)
C4	40.2(14)	39.6(15)	46.9(14)	21.0(11)	21.0(12)	14.0(11)
C2	33.8(13)	30.0(14)	60.1(16)	−2.3(11)	15.0(12)	2.6(10)
C3	40.5(15)	27.9(14)	73.2(19)	17.2(12)	26.5(14)	5.4(11)

Table S6. Bond Lengths for **8**, related to **Figure 3**, **A** and the Star Methods Section.

Atom	Atom	Length (Å)	Atom	Atom	Length (Å)
S1	C13	1.694(2)	N1	C7	1.371(3)
S2	C7	1.686(2)	N1	C6	1.401(3)
F6	C16	1.342(2)	N5	C13	1.369(3)
F1	C5	1.337(3)	N5	C15	1.404(3)
F10	C20	1.337(3)	C16	C15	1.384(3)
F5	C1	1.341(3)	C16	C17	1.379(3)
F8	C18	1.337(3)	C6	C5	1.389(3)
F7	C17	1.342(3)	C6	C1	1.386(3)
F9	C19	1.338(3)	C15	C20	1.386(3)
F2	C4	1.346(3)	C9	C8	1.519(3)
F4	C2	1.345(3)	C5	C4	1.377(3)
F3	C3	1.344(3)	C12	C11	1.525(3)
N3	C9	1.464(3)	C20	C19	1.376(3)
N3	C11	1.460(3)	C19	C18	1.376(3)
N3	C10	1.467(3)	C10	C10[1]	1.513(4)
N4	C13	1.318(3)	C17	C18	1.378(3)
N4	C12	1.459(3)	C1	C2	1.374(4)
N2	C7	1.330(3)	C4	C3	1.373(4)
N2	C8	1.453(3)	C2	C3	1.373(4)

[1] 1−X, 1−Y, 1−Z

Table S7. Bond Angles for **8**, related to **Figure 3**, **A** and the Star Methods Section.

Atom	Atom	Atom	Angle (°)	Atom	Atom	Atom	Angle (°)
C9	N3	C10	113.06(17)	F10	C20	C15	119.8(2)
C11	N3	C9	112.11(16)	F10	C20	C19	118.5(2)
C11	N3	C10	114.12(17)	C19	C20	C15	121.7(2)
C13	N4	C12	125.32(17)	F9	C19	C20	120.3(2)
C7	N2	C8	126.00(19)	F9	C19	C18	119.7(2)
C7	N1	C6	124.77(19)	C18	C19	C20	120.0(2)
C13	N5	C15	122.83(17)	N2	C8	C9	110.92(17)
N2	C7	S2	125.04(17)	N3	C11	C12	112.78(17)
N2	C7	N1	113.54(19)	N3	C10	C10[1]	110.8(2)
N1	C7	S2	121.42(16)	F7	C17	C16	120.6(2)
F6	C16	C1S	120.0(2)	F7	C17	C18	119.8(2)
F6	C16	C17	117.9(2)	Cl8	C17	C16	119.5(2)
C17	C16	C15	122.1(2)	F8	C18	C19	120.2(2)
N4	C13	S1	124.12(15)	F8	C18	C17	120.2(2)
N4	C13	N5	114. 78(18)	C19	C18	C17	119.6(2)
N5	C13	S1	121.10(16)	F5	C1	C6	118.9(2)
C5	C6	N1	123.6(2)	F5	C1	C2	119.2(2)
C1	C6	N1	119.1(2)	C2	C1	C6	121.9(2)
C1	C6	C5	117.2(2)	F2	C4	C5	119.7(2)
C16	C15	N5	121.4(2)	F2	C4	C3	119.8(2)
C16	C15	C20	117.0(2)	C3	C4	C5	120.4(2)
C20	C15	N5	121.6(2)	F4	C2	C1	119.9(2)
N3	C9	C8	111.90(17)	F4	C2	C3	120.4(2)
F1	C5	C6	120.7(2)	C3	C2	C1	119.7(2)
F1	C5	C4	118.2(2)	F3	C3	C4	120.3(3)
C4	C5	C6	121.1(2)	F3	C3	C2	120.1(2)
N4	C12	C11	109.80(18)	C4	C3	C2	119.7(2)

[1] 1−X, 1−Y, 1−Z

Table S8. Torsion Angles for **8**, related to **Figure 3**, **A** and the Star Methods Section.

A	B	C	D	Angle (°)	A	B	C	D	Angle (°)
F6	C16	C15	N5	0.3(3)	C13	N5	C15	C20	68.3(3)
F6	C16	C15	C20	−179.61(19)	C6	N1	C7	S2	8.7(3)
F6	C16	C17	F7	−0.2(3)	C6	N1	C7	N2	−171.53(18)
F6	C16	C17	C18	178.6(2)	C6	C5	C4	F2	179.7(2)
F1	C5	C4	F2	2.5(3)	C6	C5	C4	C3	2.2(4)
F1	C5	C4	C3	−175.0(2)	C6	C1	C2	F4	−179.2(2)
F10	C20	C19	F9	-0.3(3)	C6	C1	C2	C3	1.0(4)
F10	C20	C19	C18	−179.9(2)	C15	N5	C13	S1	5.0(3)
F5	C1	C2	F4	0.5(4)	C15	N5	C13	N4	−175.1(2)
F5	C1	C2	C3	−179.4(2)	C15	C16	C17	F7	−178.7(2)
F7	C17	C18	F8	−0.5(3)	C15	C16	C17	C18	0.1(3)
F7	C17	C18	C19	179.8(2)	C15	C20	C19	F9	179.4(2)
F9	C19	C18	F8	−0.2(3)	C15	C20	C19	C18	−0.2(3)
F9	C19	C18	C17	179.5(2)	C9	N3	C11	C12	−165.52(17)
F2	C4	C3	F3	−1.5(4)	C9	N3	C10	C10^{1}	84.6(3)
F2	C4	C3	C2	−179.2(2)	C5	C6	C1	F5	179.8(2)
F4	C2	C3	F3	2.5(4)	C5	C6	C1	C2	−0.5(4)
F4	C2	C3	C4	−179.7(2)	C5	C4	C3	F3	176.1(2)
N3	C9	C8	N2	67.4(2)	C5	C4	C3	C3	−1.7(4)
N4	C12	C11	N3	48.7(2)	C12	N4	C13	S1	−1.6(3)
N1	C6	C5	F1	−0.2(3)	C12	N4	C13	N5	178.5(2)
N1	C6	C5	C4	−177.3(2)	C20	C19	C18	F8	179.3(2)
N1	C6	C1	F5	−3.7(3)	C20	C19	C18	C17	−0.9(3)
N1	C6	C1	C2	176.0(2)	C8	N2	C7	S2	−7.6(3)
N5	C15	C20	F10	1.0(3)	C8	N2	C7	N1	172.59(18)
N5	C15	C20	C19	−178.7(2)	C11	N3	C9	C8	88.5(2)
C7	N2	C8	C9	−105.9(2)	C11	N3	C10	C10^{1}	−145.7(2)
C7	N1	C6	C5	−68.0(3)	C10	N3	C9	C8	−140.74(19)
C7	N1	C6	C1	115.8(2)	C10	N3	C11	C12	64.3(2)
C16	C15	C20	F10	−0.2(3)	C17	C16	C15	N5	178.8(2)
C16	C15	C20	C19	1.2(3)	C17	C16	C15	C20	−1.1(3)
C16	C17	C18	F8	−179.3(2)	C1	C6	C5	F1	176.1(2)
C16	C17	C18	C19	1.0(3)	C1	C6	C5	C4	−1.1(3)
C13	N4	C12	C11	−147.4(2)	C1	C2	C3	F4	−177.6(2)
C13	N5	C15	C16	−111.6(2)	C1	C2	C3	C4	0.2(4)

Table S9. Hydrogen Atom Coordinates (A × 10^4) and Isotropic Displacement Parameters (A^2 × 10^3) for **8**, related to **Figure 3**, **A** and the Star Methods Section.

Atom	x	y	z	U(eq)
H4	3731.94	5288.31	2928.29	33
H2	8985.3	5391.01	2409.97	34
H1	8922.89	3652.55	2649.85	36
H5	3611.59	3562.31	3285.05	39
H9A	6558.72	7732.76	1591.81	34
H9B	7329.3	6590.53	980.33	34
H12A	1679.69	6642.5	2245.77	36
H12B	1764.11	5847.55	985.61	36
H8A	8721.84	7372.48	2992.87	36
H8B	7094.56	7039.11	3410.6	36
H11A	3592.57	7490.13	1283.22	34
H11B	4376.68	7362.7	2595.66	34
H10A	4995.31	6084.83	−409.93	36
H10B	3434.29	5460.16	−116.96	36

Crystal structure determination of 8:

Crystal Data for $C_{4.75}H_{3.5}F_{2.5}N_{1.25}S_{0.5}$ (M = 141.62 gmol^{-1}): triclinic, space group P-1 (no. 2), a = 8.4103(4) Å, b = 11.5621(5) Å, c = 11.8311(6) Å, α = 101.535(4)°, β = 101.482(4)°, γ = 92.384(4)°, V = 1100.69(9) Å^3, Z = 8, T = 150.00(10) K, μ(Mo Kα) = 0.346 mm^{-1}, $Dcalc$ = 1.709 gcm^{-3}, 8135 reflections measured (5.476° ≤ 2Θ ≤ 59.116°), 5071 unique (R_{int} = 0.0250, R_{sigma} = 0.0484) which were used in all calculations. The final R_1 was 0.0443 (I > 2σ(I)), and wR_2 was 0.1079 (all data).

Refinement model description of 8:

Number of restraints - 0, number of constraints - unknown.
Details:
Fixed Uiso
At 1.2 times of:
All C(H,H) groups, All N(H) groups.
2.a Secondary CH_2 refined with riding coordinates:
C9(H9A,H9B), C12(H12A,H12B), C8(H8A,H8B), C11(H11A,H11B), C10(H10A,H10B).
2.b Aromatic/amide H refined with riding coordinates:
N4(H4), N2(H2), N1(H1), N5(H5).

Table S10. Fractional Atomic Coordinates (×10^4) and Equivalent Isotropic Displacement Parameters (Å^2×10^3) for **8·2NO$_3$.** U_{eq} is defined as 1/3 of the trace of the orthogonalized U_{IJ} tensor, related to **Figure 3**, **B** and the Star Methods Section.

Atom	x	y	z	U(eq)
S2	2163.0(5)	2100.3(5)	6265.2(5)	46.3(2)
S1	8637.4(6)	7695.8(5)	8132.4(6)	51.3(2)
F1	10873.2(15)	7038.1(14)	10042.3(13)	59.4(4)
F10	4332.2(17)	284.0(14)	6240.7(14)	60.6(4)
F2	12961.2(14)	7515.9(14)	10200.6(14)	63.1(5)
F6	4368.0(16)	2953.3(12)	9319.6(15)	59.5(4)
F8	3955.2(18)	−675.0(14)	8907.5(16)	64.7(5)
F3	13051.4(17)	6675.1(16)	8291.1(17)	66.7(5)
F7	4098.9(17)	1439.8(15)	10071.2(15)	64.1(5)
F9	4176.1(19)	−1207.1(13)	7051.7(17)	70.1(5)
F5	8937.3(16)	4881.1(17)	6022.6(14)	75.4(6)
F4	11041(2)	5320(2)	6200.7(15)	77.7(6)
O1*	6566.0(18)	3818.8(15)	7689(2)	60.3(6)
O2*	6742.7(19)	2209.1(15)	7410(2)	59.7(5)
N1'	−351.5(18)	1114.2(15)	7357.0(15)	39.1(4)
N3	4750.1(17)	5425.7(15)	6224.1(15)	37.7(4)
N4	3877.7(19)	3599.1(17)	6540.8(18)	44.5(5)
O3*	7968(2)	3433.5(18)	7453(2)	69.9(6)
N1*	7098.4(19)	3145.6(17)	7520.8(19)	47.8(5)
N2	7255.2(18)	6095.9(16)	8113.8(16)	41.2(4)
N5	4488(2)	2467.4(18)	7373(2)	49.7(5)
N1	8753.1(19)	5703.3(17)	7935.9(19)	47.7(5)
C10	4480(2)	5106(2)	5042.6(18)	40.5(5)
C14'	1504(2)	2866.0(18)	8609.2(19)	42.3(5)
C7	8185(2)	6448.9(19)	8064.5(18)	41.1(5)
C5'	−1544(2)	762(2)	7214(2)	42.7(5)
C13	3563(2)	2761.3(19)	6746.1(19)	42.6(5)
C9	5537(2)	6581.5(18)	7004(2)	41.8(5)
C1	9842(2)	5966.6(19)	8021(2)	42.6(5)
C15	4297(2)	1922(2)	8736(2)	46.1(6)
C2	10889(2)	6633.6(19)	9082(2)	42.5(5)
C1'	371(2)	392.2(19)	7708(2)	43.4(6)
C14	4357(2)	1662(2)	7760(2)	44.5(5)
C12	3058(2)	4034(2)	5845(2)	47.1(6)
C13'	265(2)	2320.9(18)	8275.9(19)	41.1(5)
C16	4174(2)	1155(2)	9133(2)	48.7(6)
C8	6468(2)	6709.2(19)	8182.4(19)	43.6(5)
C18	4309(2)	596(2)	7205(2)	47.3(6)
C6'	−2351(2)	−416(2)	6350(2)	44.6(6)
C2'	642(2)	331(2)	8821(2)	46.0(6)

C15'	1841(2)	4132.6(19)	9223(2)	46.6(6)
C3	11961(2)	6881.6(19)	9171(2)	45.8(6)
C11	3680(2)	5248(2)	6258(2)	44.0(5)
C9'	−492(2)	997(2)	6219.5(19)	45.0(6)
C4	12010(2)	6452(2)	8199(2)	48.3(6)
C17	4210(3)	−178(2)	7607(2)	52.1(6)
C6	9921(2)	5536(2)	7063(2)	49.9(6)
C01C	4109(2)	94(2)	8551(2)	49.9(6)
C16'	3095(3)	4746(2)	9636(3)	57.7(7)
C5	10985(3)	5757(2)	7138(2)	53.0(6)
C10'	−965(3)	1789(2)	5763(2)	53.4(7)
C7'	−3488(3)	−611(2)	6343(2)	54.8(7)
C8'	−4425(3)	−1720(2)	5398(2)	51.8(6)
C3'	1461(3)	−350(2)	9091(2)	53.3(6)
C11'	−1106(3)	1466(2)	4575(2)	57.2(7)
C4'	1885(3)	−333(2)	10268(3)	66.2(9)
C12'	−1307(4)	2341(3)	4154(3)	80.8(11)

Table S11. Anisotropic Displacement Parameters (Å^2×10^3) for **8·2NO$_3$**. The Anisotropic Displacement Factor Exponent Takes the Form: $-2\pi^2[h^2a^{*2}U_{11}+2hka^*b^*U_{12}+\ldots]$, related to **Figure 3**, **B** and the Star Methods Section.

Atom	U_{11}	U_{22}	U_{33}	U_{23}	U_{13}	U_{12}
S2	36.6(3)	48.0(3)	43.8(3)	7.0(2)	26.1(3)	1.7(3)
S1	50.3(4)	29.9(3)	66.7(4)	10.2(3)	35.2(3)	9.0(3)
F1	55.9(9)	63.7(10)	45.9(8)	3.4(7)	33.4(7)	15.6(8)
F10	69.7(11)	52.7(9)	59.1(9)	11.3(7)	44.3(8)	16.8(8)
F2	40.6(8)	52.1(9)	58.6(9)	-2.9(7)	20.1(7)	6.3(7)
F6	65.7(10)	35.4(7)	72.1(10)	6.7(7)	45.5(9)	14.7(7)
F8	68.7(11)	50.9(9)	77.5(11)	30.9(8)	40.7(9)	16.8(8)
F3	57.4(10)	71.4(11)	87.8(12)	30.2(9)	54.2(10)	22.2(9)
F7	71.6(11)	62.7(10)	63.6(9)	18.0(8)	48.2(9)	18.4(9)
F9	89.2(14)	37.5(8)	86.6(12)	15.7(8)	56.6(11)	25.5(9)
F5	54.8(10)	82.4(13)	43.2(8)	-2.3(8)	15.8(8)	15.6(9)
F4	82.6(13)	104.5(15)	55.2(9)	23.6(9)	50.0(10)	37.9(12)
O1*	47.7(10)	34.2(9)	93.3(14)	12.5(9)	44.9(11)	12.1(8)
O2*	53.6(11)	37.5(9)	88.5(14)	22.5(9)	43.0(11)	12.6(9)
N1'	40.1(10)	30.3(9)	39.4(9)	1.0(7)	27.5(8)	4.3(8)
N3	38.1(10)	32.0(9)	36.7(9)	5.0(7)	23.1(8)	8.8(8)
N4	36.6(10)	40.2(10)	53.8(11)	12.5(8)	29.1(9)	7.3(9)
O3*	67.6(14)	50.7(11)	121.3(19)	37.4(12)	71.4(15)	25.4(11)
N1*	39.3(11)	38.3(11)	58.5(12)	10.3(9)	29.1(9)	11.1(9)
N2	39.7(10)	33.6(9)	42.0(9)	7.5(7)	23.1(8)	8.8(8)
N5	41.4(11)	41.5(11)	66.9(13)	18.5(9)	34.6(10)	8.2(9)
N1	40.2(11)	31.8(10)	62.7(12)	9.5(9)	30.4(10)	8.1(8)
C10	39.6(12)	39.6(11)	36.4(10)	8.3(8)	22.4(9)	12.2(10)
C14'	44.6(12)	30.5(11)	40.3(10)	1.1(8)	26.0(10)	6.0(10)
C7	38.8(11)	33.2(11)	36.9(10)	2.4(8)	20.3(9)	6.7(9)
C5'	41.3(12)	39.2(12)	44.4(11)	5.9(9)	30.3(10)	9.7(10)
C13	42.0(12)	35.9(11)	40.5(11)	1.9(9)	27.1(10)	6.7(10)
C9	45.1(12)	29.6(10)	43.1(11)	5.3(8)	25.9(10)	10.8(10)
C1	42.7(12)	31.4(10)	48.8(12)	9.3(9)	27.0(10)	11.5(10)
C15	42.9(12)	33.0(11)	54.8(13)	6.9(10)	30.0(11)	9.3(10)
C2	44.7(12)	35.0(11)	41.6(11)	5.7(9)	26.6(10)	12.5(10)
C1'	42.5(12)	31.7(10)	50.2(12)	2.2(9)	32.7(10)	7.4(10)
C14	36.5(11)	35.8(11)	53.9(13)	10.6(10)	26.5(10)	7.4(10)
C12	40.1(12)	47.3(13)	47.7(12)	12.0(10)	26.4(10)	10.1(11)
C13'	48.1(13)	28.7(10)	39.3(10)	1.5(8)	28.5(10)	8.4(10)
C16	45.1(13)	43.0(13)	54.9(13)	12.9(10)	31.8(11)	9.9(11)
C8	44.4(12)	35.7(11)	38.9(11)	1.7(9)	25.4(10)	7.4(10)
C18	43.3(13)	40.0(12)	52.1(13)	8.0(10)	30.2(11)	11.1(11)
C6'	43.7(13)	40.5(12)	42.0(11)	5.8(9)	27.8(10)	6.9(11)
C2'	49.7(14)	34.5(11)	47.0(12)	5.6(9)	30.5(11)	10.0(10)

C15'	51.5(14)	29.8(11)	45.2(11)	6.3(9)	24.2(10)	10.3(10)
C3	42.7(13)	32.5(11)	48.3(12)	5.0(9)	24.1(10)	8.9(10)
C11	43.3(12)	43.9(12)	46.8(12)	13.2(10)	29.9(10)	16.3(11)
C9'	47.0(13)	38.3(12)	39.2(11)	1.0(9)	28.9(10)	4.6(10)
C4	45.7(13)	47.0(14)	61.5(14)	21.0(11)	36.9(12)	18.5(12)
C17	52.1(15)	32.7(11)	62.3(15)	8.1(10)	33.4(13)	12.6(11)
C6	47.4(14)	47.0(13)	40.2(11)	7.8(10)	21.0(11)	13.7(12)
C01C	46.3(14)	39.2(12)	58.8(14)	17.4(11)	28.4(12)	10.3(11)
C16'	54.7(16)	31.5(11)	64.2(16)	9.4(11)	26.2(13)	3.8(11)
C5	56.2(16)	59.8(16)	47.1(13)	17.3(11)	34.4(12)	22.2(14)
C10'	58.8(16)	48.0(14)	46.3(12)	10.2(10)	31.7(12)	12.7(13)
C7'	47.0(14)	50.1(14)	53.3(13)	2.2(11)	33.9(12)	3.6(12)
C8'	50.9(14)	44.4(13)	53.2(13)	11.3(11)	32.9(12)	3.8(12)
C3'	51.9(15)	41.0(13)	67.4(16)	15.3(11)	38.7(13)	14.0(12)
C11'	54.4(16)	52.8(15)	43.2(12)	6.9(11)	24.1(12)	4.5(13)
C4'	75(2)	42.1(13)	59.3(15)	11.5(12)	26.8(15)	21.4(14)
C12'	99(3)	65(2)	60.2(18)	23.1(15)	36.8(19)	12(2)
C8	44.4(12)	35.7(11)	38.9(11)	1.7(9)	25.4(10)	7.4(10)
C3'	51.9(15)	41.0(13)	67.4(16)	15.3(11)	38.7(13)	14.0(12)
C11'	54.4(16)	52.8(15)	43.2(12)	6.9(11)	24.1(12)	4.5(13)
C4'	75(2)	42.1(13)	59.3(15)	11.5(12)	26.8(15)	21.4(14)
C12'	99(3)	65(2)	60.2(18)	23.1(15)	36.8(19)	12(2)

Table S12. Bond Lengths for **8·2NO$_3$**, related to **Figure 3**, **B** and the Star Methods Section.

Atom	Atom	Length (Å)	Atom	Atom	Length (Å)
S2	C13	1.676(3)	N1	C1	1.407(3)
S1	C7	1.679(3)	C10	C10[1]	1.521(5)
F1	C2	1.330(3)	C14'	C13'	1.513(4)
F10	C18	1.339(3)	C14'	C15'	1.539(3)
F2	C3	1.336(3)	C5'	C6'	1.512(3)
F6	C15	1.338(3)	C9	C8	1.515(3)
F8	C01C	1.343(3)	C1	C2	1.389(3)
F3	C4	1.336(3)	C1	C6	1.380(4)
F7	C16	1.344(3)	C15	C14	1.386(4)
F9	C17	1.343(3)	C15	C16	1.372(4)
F5	C6	1.338(3)	C2	C3	1.380(4)
F4	C5	1.336(3)	C1'	C2'	1.517(4)
O1*	N1*	1.262(3)	C14	C18	1.379(3)
O2*	N1*	1.229(3)	C12	C11	1.519(4)
N1'	C5'	1.517(3)	C16	C01C	1.374(4)
N1'	C1'	1.510(3)	C18	C17	1.380(4)
N1'	C13'	1.528(3)	C6'	C7'	1.523(4)
N1'	C9'	1.522(3)	C2'	C3'	1.524(4)
N3	C10	1.467(3)	C15'	C16'	1.516(4)
N3	C9	1.470(3)	C3	C4	1.374(4)
N3	C11	1.463(3)	C9'	C10'	1.501(4)
N4	C13	1.335(4)	C4	C5	1.383(4)
N4	C12	1.455(4)	C17	C01C	1.373(4)
O3*	N1*	1.250(3)	C6	C5	1.376(4)
N2	C7	1.331(3)	C10'	C11'	1.541(4)
N2	C8	1.449(3)	C7'	C8'	1.515(3)
N5	C13	1.363(3)	C3'	C4'	1.520(4)
N5	C14	1.412(4)	C11'	C12'	1.504(5)
N1	C7	1.360(3)	-	-	-

[1]1−X,1−Y,1−Z

Table S13. Bond Angles for **8·2NO$_3$**, related to **Figure 3**, **B** and the Star Methods Section.

Atom	Atom	Atom	Angle (°)	Atom	Atom	Atom	Angle (°)
C5'	N1'	C13'	105.53(16)	C18	C14	C15	117.2(3)
C5'	N1'	C9'	111.47(18)	N4	C12	C11	109.4(2)
C1'	N1'	C5'	112.1(2)	C14'	C13'	N1'	116.15(18)
C1'	N1'	C13'	111.69(18)	F7	C16	C15	120.0(2)
C1'	N1'	C9'	104.86(17)	F7	C16	C01C	120.6(3)
C9'	N1'	C13'	111.31(19)	C15	C16	C01C	119.4(3)
C10	N3	C9	111.46(19)	N2	C8	C9	112.05(18)
C11	N3	C10	112.33(18)	F10	C18	C14	120.1(3)
C11	N3	C9	110.27(17)	F10	C18	C17	118.4(2)
C13	N4	C12	124.9(2)	C14	C18	C17	121.4(3)
O2*	N1*	O1*	119.4(2)	C5'	C6'	C7'	109.10(19)
O2*	N1*	O3*	121.0(2)	C1'	C2'	C3'	109.7(2)
O3*	N1*	O1*	119.7(2)	C16'	C15'	C14'	113.0(2)
C7	N2	C8	124.9(2)	F2	C3	C2	120.7(2)
C13	N5	C14	122.6(2)	F2	C3	C4	119.3(2)
C7	N1	C1	123.1(2)	C4	C3	C2	120.0(2)
N3	C10	C10[1]	111.1(2)	N3	C11	C12	112.68(19)
C13'	C14'	C15'	109.5(2)	C10'	C9'	N1'	117.72(19)
N2	C7	S1	124.20(19)	F3	C4	C3	119.9(2)
N2	C7	N1	114.4(2)	F3	C4	C5	120.4(2)
N1	C7	S1	121.4(2)	C3	C4	C5	119.7(3)
C6'	C5'	N1'	117.06(18)	F9	C17	C18	119.9(3)
N4	C13	S2	123.9(2)	F9	C17	C01C	120.1(3)
N4	C13	N5	113.9(2)	C01C	C17	C18	119.9(2)
N5	C13	S2	122.2(2)	F5	C6	C1	119.4(3)
N3	C9	C8	111.6(2)	F5	C6	C5	118.5(2)
C2	C1	N1	121.3(2)	C5	C6	C1	122.1(2)
C6	C1	N1	121.4(2)	F8	C01C	C16	120.2(3)
C6	C1	C2	117.2(2)	F8	C01C	C17	120.0(2)
F6	C15	C14	119.3(3)	C17	C01C	C16	119.9(3)
F6	C15	C16	118.5(2)	F4	C5	C4	119.4(3)
C16	C15	C14	122.1(2)	F4	C5	C6	121.0(3)
F1	C2	C1	119.9(2)	C6	C5	C4	119.5(2)
F1	C2	C3	118.6(2)	C9'	C10'	C11'	108.5(2)
C3	C2	C1	121.5(2)	C8'	C7'	C6'	113.4(2)
N1'	C1'	C2'	116.92(18)	C4'	C3'	C2'	112.4(2)
C15	C14	N5	121.0(2)	C12'	C11'	C10'	113.3(3)
C18	C14	N5	121.8(2)	-	-	-	-

[1] 1−X, 1−Y, 1−Z

Table S14. Torsion Angles for **$8\cdot 2NO_3$**, related to **Figure 3**, **B** and the Star Methods Section.

A	B	C	D	Angle (°)	A	B	C	D	Angle (°)
F1	C2	C3	F2	−0.1(4)	C1	C2	C3	F2	−179.3(2)
F1	C2	C3	C4	178.3(2)	C1	C2	C3	C4	−1.0(4)
F10	C18	C17	F9	2.1(4)	C1	C6	C5	F4	179.2(3)
F10	C18	C17	C01C	−175.9(3)	C1	C6	C5	C4	−1.5(5)
F2	C3	C4	F3	−0.3(4)	C15	C14	C18	F10	178.3(2)
F2	C3	C4	C5	177.3(3)	C15	C14	C18	C17	−0.2(4)
F6	C15	C14	N5	−0.1(4)	C15	C16	C01C	F8	−178.1(2)
F6	C15	C14	C18	178.4(2)	C15	C16	C01C	C17	2.3(4)
F6	C15	C16	F7	1.5(4)	C2	C1	C6	F5	179.5(2)
F6	C15	C16	C01C	−179.5(3)	C2	C1	C6	C5	−0.5(4)
F3	C4	C5	F4	−0.8(4)	C2	C3	C4	F3	−178.7(2)
F3	C4	C5	C6	179.9(3)	C2	C3	C4	C5	−1.1(4)
F7	C16	C01C	F8	0.9(4)	C1'	N1'	C5'	C6'	−57.8(3)
F7	C16	C01C	C17	−178.7(3)	C1'	N1'	C13'	C14'	56.1(3)
F9	C17	C01C	F8	−1.2(4)	C1'	N1'	C9'	C10'	−168.1(2)
F9	C17	C01C	C16	178.4(3)	C1'	C2'	C3'	C4'	173.3(2)
F5	C6	C5	F4	−0.8(4)	C14	N5	C13	S2	−6.4(3)
F5	C6	C5	C4	178.5(3)	C14	N5	C13	N4	174.8(2)
N1'	C5'	C6'	C7'	179.3(2)	C14	C15	C16	F7	−178.8(2)
N1'	C1'	C2'	C3'	−175.2(2)	C14	C15	C16	C01C	0.2(4)
N1'	C9'	C10'	C11'	−176.3(2)	C14	C18	C17	F9	−179.5(3)
N3	C9	C8	N2	63.3(3)	C14	C18	C17	C01C	2.6(4)
N4	C12	C11	N3	56.9(3)	C12	N4	C13	S2	−2.1(3)
N5	C14	C18	F10	−3.2(4)	C12	N4	C13	N5	176.7(2)
N5	C14	C18	C17	178.4(2)	C13'	N1'	C5'	C6'	-179.6(2)
N1	C1	C2	F1	−0.1(4)	C13'	N1'	C1'	C2'	60.1(3)
N1	C1	C2	C3	179.1(2)	C13'	N1'	C9'	C10'	−47.2(3)
N1	C1	C6	F5	2.1(4)	C13'	C14'	C15'	C16'	177.4(2)
N1	C1	C6	C5	−177.9(3)	C16	C15	C14	N5	−179.8(2)
C10	N3	C9	C8	-139.4(2)	C16	C15	C14	C18	−1.2(4)
C10	N3	C11	C12	75.9(3)	C8	N2	C7	S1	2.8(3)
C7	N2	C8	C9	81.9(3)	C8	N2	C7	N1	−176.3(2)
C7	N1	C1	C2	72.2(4)	C18	C17	C01C	F8	176.8(2)
C7	N1	C1	C6	−110.6(3)	C18	C17	C01C	C16	−3.6(4)
C5'	N1'	C1'	C2'	−58.1(2)	C15'	C14'	C13'	N1'	162.3(2)
C5'	N1'	C13'	C14'	178.2(2)	C3	C4	C5	F4	−178.4(3)
C5'	N1'	C9'	C10'	70.3(3)	C3	C4	C5	C6	2.3(4)
C5'	C6'	C7'	C8'	172.7(3)	C11	N3	C10	C10[1]	−159.2(3)
C13	N4	C12	C11	152.4(2)	C11	N3	C9	C8	95.1(2)
C13	N5	C14	C15	−79.7(3)	C9'	N1'	C5'	C6'	59.4(3)
C13	N5	C14	C18	101.8(3)	C9'	N1'	C1'	C2'	−179.2(2)
C9	N3	C10	C10[1]	76.4(3)	C9'	N1'	C13'	C14'	−60.7(3)

C9	N3	C11	C12	−159.1(2)	C9'	C10'	C11'	C12'	−166.9(3)
C1	N1	C7	S1	9.6(3)	C6	C1	C2	F1	−177.5(2)
C1	N1	C7	N2	−171.2(2)	C6	C1	C2	C3	1.8(4)

[1] 1−X, 1−Y, 1−Z

Table S15. Hydrogen Atom Coordinates (A × 10[4]) and Isotropic Displacement Parameters (A[2] × 10[3]) for **8·2NO$_3$**, related to **Figure 3**, **B** and the Star Methods Section.

Atom	*x*	*y*	*z*	U(eq)
H4	4637.84	3911.5	6849.65	53
H2	7107.2	5440.91	8104.09	49
H5	5195.67	2792.7	7542.01	60
H1	8424.2	5024.25	7792.59	57
H10C	4292.81	5704.68	4817.25	49
H10D	3775.11	4428.16	4497.1	49
H14A	1538.71	2653.07	7906.52	51
H14B	2071.27	2613.67	9132.57	51
H5'A	−1400.71	873.83	7980.61	51
H5'B	−1970.06	1265.33	6985.08	51
H9A	5059.77	7058.22	7098.06	50
H9B	5930.69	6831.69	6652.72	50
H1'A	1132.01	662.61	7783.09	52
H1'B	−54.48	−369.45	7069.18	52
H12D	2362.99	3958.32	5916.97	57
H12E	2776.82	3608.85	5018.59	57
H13A	−231.96	2758.9	7990.37	49
H13B	300.16	2358.84	8983.8	49
H8A	6938.63	7503.91	8700.46	52
H8B	6072.93	6443.89	8524.94	52
H6'A	−1951.89	−940.35	6564.93	54
H6'B	−2536.25	−543.77	5566.43	54
H2'A	−102.73	−15.92	8736.74	55
H2'B	1027.22	1087.48	9467.38	55
H15A	1283	4376.88	8682.39	56
H15B	1756.78	4331.1	9897.01	56
H11C	3127	5532.24	5764.77	53
H11D	3895.15	5675.28	7062.95	53
H9'A	292.67	1071.61	6316.17	54
H9'B	−1022.62	235.31	5619.66	54
H16A	3653.94	4518.7	10183.6	87
H16B	3262.22	5545.47	10021.74	87
H16C	3179.19	4569.38	8970.57	87
H10A	−410.83	2556.32	6308.75	64
H10B	−1739.71	1752.8	5678.63	64
H7'A	−3299.16	−565.11	7104.88	66
H7'B	−3814.33	−14.17	6241.02	66

H8'A	−4697.98	−1733.34	4638.63	78
H8'B	−5094.42	−1832.47	5494.55	78
H8'C	−4086.98	−2313.31	5449.65	78
H3'A	1035.64	−1126.21	8482.72	64
H3'B	2155.67	−54.66	9072.86	64
H11A	−384.08	1322.5	4631.78	69
H11B	−1782.49	769.9	3997.44	69
H4'A	2217.62	435.71	10861.24	99
H4'B	2493.74	−687.25	10452.44	99
H4'C	1215.53	−736.37	10247.99	99
H12A	−1974.05	2539.77	4166.74	121
H12B	−1484.85	2053.53	3360.04	121
H12C	−592.48	2998.09	4660.8	121

Crystal structure determination of 8·2NO$_3$:

Crystal Data for $C_{11.666667}H_{16.666667}F_{3.333333}N_{2.333333}OS_{0.666667}$ (M = 290.31 gmol^{-1}): triclinic, space group P-1 (no. 2), a = 13.5201(4) Å, b = 13.5649(4) Å, c = 14.5206(4) Å, α = 109.335(3)°, β = 117.651(3)°, γ = 97.173(3)°, V = 2098.64(13) Å^3, Z = 6, T = 150.0(4) K, μ(Cu Kα) = 1.927 mm^{-1}, $Dcalc$ = 1.378 gcm^{-3}, 20916 reflections measured (7.326° ≤ 2Θ ≤ 148.922°), 7998 unique (R_{int} = 0.0622, R_{sigma} = 0.0470) which were used in all calculations. The final R_1 was 0.0694 (I > 2σ(I)), and wR_2 was 0.2091 (all data).

Refinement model description of 8·2NO$_3$:

Number of restraints - 0, number of constraints - unknown.
Details:
1. Fixed Uiso
 At 1.2 times of:
 All C(H,H) groups, All N(H) groups
 At 1.5 times of:
 All C(H,H,H) groups
2.a Secondary CH$_2$ refined with riding coordinates:
 C10(H10C,H10D), C14'(H14A,H14B), C5'(H5'A,H5'B), C9(H9A,H9B), C1'(H1'A,H1'B), C12(H12D,H12E), C13'(H13A,H13B), C8(H8A,H8B), C6'(H6'A,H6'B), C2'(H2'A,H2'B), C15'(H15A,H15B), C11(H11C,H11D), C9'(H9'A,H9'B), C10'(H10A,H10B), C7'(H7'A, H7'B), C3'(H3'A,H3'B), C11'(H11A,H11B)
2.b Aromatic/amide H refined with riding coordinates:
 N4(H4), N2(H2), N5(H5), N1(H1)
2.c Idealized Me refined as a rotating group:
 C16'(H16A,H16B,H16C), C8'(H8'A,H8'B,H8'C), C4'(H4'A,H4'B,H4'C), C12'(H12A, H12B,H12C)

S7. References:

[S1] Avval, M.M., Murthy, S.V., and Shashikanth, S. (2014). Synthesis and antimicrobial activity evaluation of poly ethylene imine (PEI) dendrimer modified with 1,3,4-oxadiazole derivatives. Res. J. Pharm. Biol. Chem. Sci. *5*, 441-447.

[S2] Raymond, K.N., Corneillie, T.M., and Xu, J. (2014) Luminescent macrocyclic lanthanide complexes. United States patent 8729258, and granted 20-05-2014.

[S3] Keypour, H., Khanmohammadi, H., Wainwright, K.P., and Taylor, M.R. (2005). Synthesis, crystal structures and ab initio studies of some heptaaza manganese (II) macrocyclic Schiff-base complexes with two 2-aminoethyl pendant arms. Inorg. Chim. Acta *358*, 247-256. https://doi.org/10.1016/j.ica.2004.02.040.

[S4] Wagnon, B.K., and Jackels, S.C. (1989). Synthesis, characterization, and aqueous proton relaxation enhancement of a manganese(II) heptaaza macrocyclic complex having pendant arms. Inorganic Chemistry *28*, 1923-1927. https://doi.org/10.1021/ic00309a030.

[S5] Gilchrist, A.M., Chen, L., Wu, X., Lewis, W., Howe, E.N.W., Macreadie, L.K., and Gale, P.A. (2020). Tetrapodal Anion Transporters. Molecules *25*, 5179. https://doi.org/10.3390/molecules25215179.
